# Cascade Iodine-Catalyzed Synthesis of Nitrogenated Aromatics from 2‑Pyrones

**DOI:** 10.1021/acs.joc.5c03172

**Published:** 2026-03-31

**Authors:** Bruna B. Souza, Tadeu L. G. Cabral, Catarina B. Varriano, Claudio F. Tormena, Julio C. Pastre

**Affiliations:** Institute of Chemistry, 28132 Universidade Estadual de Campinas (UNICAMP), Campinas, SP 13083-970, Brazil

## Abstract

Herein, we report an improved strategy for the synthesis of nitrogen-containing aromatic compounds featuring a nitrogen atom directly attached to a benzene ring. The method relies on a thermal one-pot Diels–Alder/decarboxylation/iodine-catalyzed aromatization cascade reaction starting from 2-pyrones, enabling access to novel C–3-nitrogenated phthalimides and related aromatics in yields of up to 95%. Iodine, a simple and readily available catalyst, was employed to promote the transformation while effectively suppressing the competing second Diels–Alder reaction. Subsequent derivatization demonstrated the synthetic utility of this approach through the preparation of pomalidomide, an immunomodulatory drug.

## Graphical abstract

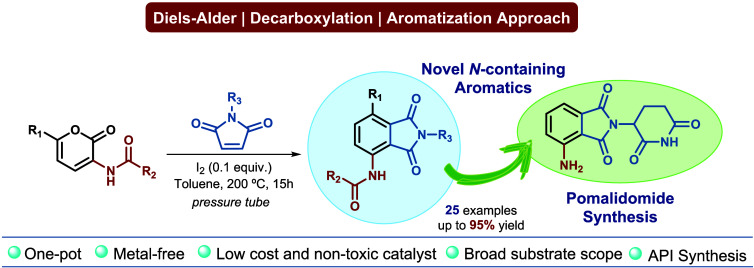

## 1 Introduction

Nitrogenated aromatic compounds are of great significance and constitute important building blocks in various fields. Nitrogen atoms also play a fundamental role in medicinal chemistry since it is estimated that over 84% of the approved drugs have at least one nitrogen atom in their scaffolds and over 60% are composed by some sort of nitrogen heterocycle.^1,2^ Therefore, nitrogen-containing aromatics remain attractive targets for the synthetic organic community.

In this scenario, phthalimides arise as a fascinating class of biologically active *N*-heterocycles, widely found in natural products and pharmaceutical compounds.^3–5^ Their broad-ranging biological activities, including anti-inflammatory,^6^ anticonvulsant,^7^ antimicrobial,^6^ antifungal,^8^ antitumor,^9^ antibacterial,^10^ and antimalarial,^11^ underscore their pharmacological relevance. Furthermore, phthalimides have garnered attention in materials science, particularly in the development of polymers and other materials.^12–14^

Among the phthalimide scaffolds, lenalidomide (Revlimid) and pomalidomide (Pomalyst) stand out due to their high efficacy in treating multiple myeloma, while apremilast (Otezla) is known for the treatment of plaque psoriasis (Scheme 1a).^4^ These compounds feature a nitrogen substituent at the C–3 position, highlighting the demand for new C–3-functionalized nitrogen aromatics with improved therapeutic potential. Traditionally, their synthesis relied on different strategies, such as—but not limited to—a multistep process involving a nitration and a reduction step and, in some cases, followed by alkylation or acylation/benzoylation reactions (Scheme 1b).^15^ Furthermore, besides the poor step economy,^15^ the insertion of a nitro group is usually associated with harsh conditions and hazardous reagents, as the classical electrophilic nitration approach requires the use of a toxic and explosive mixture of strong mineral acids.^16^

**Scheme 1:**
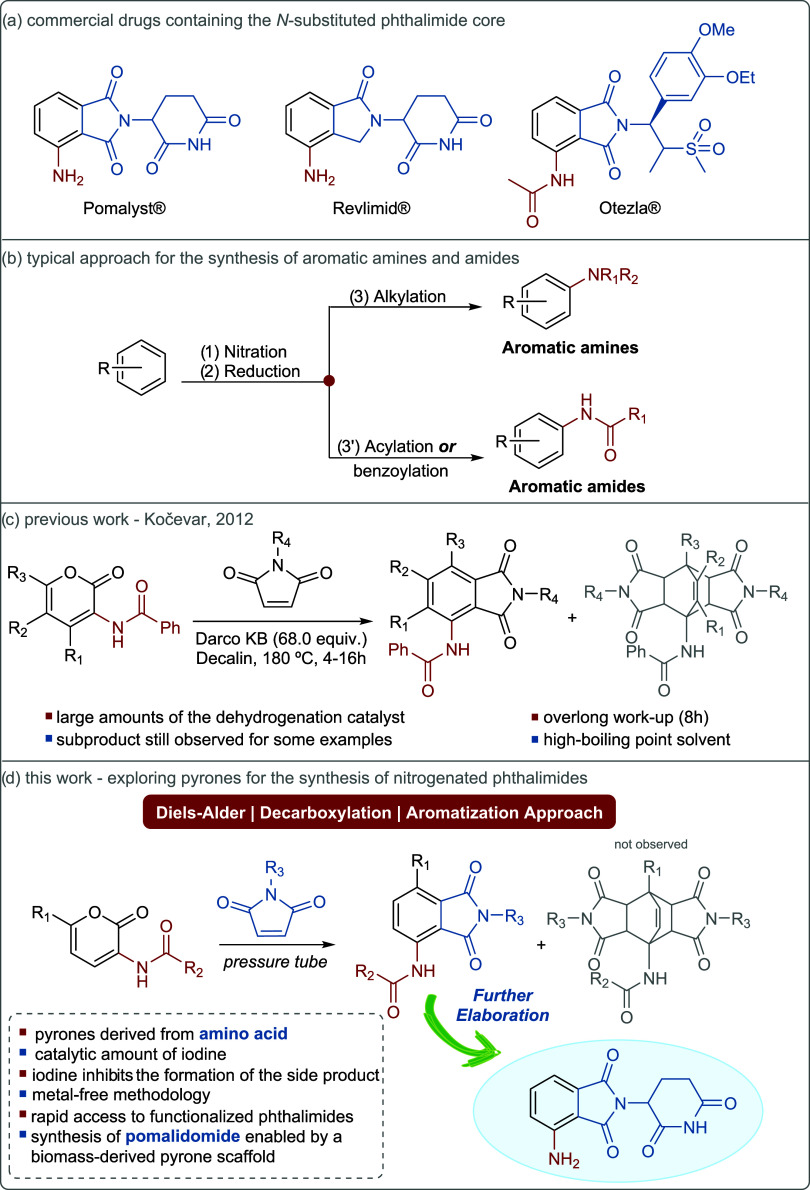
Nitrogenated 2-Pyrones as Valuable Compounds in the Synthesis of Aromatics via DA Reaction: (a) commercial drugs featuring the N-substituted phthalimide core; (b) typical approaches to aromatic amines and amides; (c) previous work (Koc̆evar, 2012); (d) this work, exploring pyrones toward nitrogenated phthalimides.

In this context, 2-pyrones emerge as an important building block for the synthesis of high-added value chemicals.^17^ 2-Pyrones are cyclic dienes capable of undergoing Diels–Alder (DA) reactions, although generally less readily than most conjugated cyclic dienes due to their aromatic character.^18^ To access new aromatics, these pyrones can be sequentially submitted to Diels–Alder, decarboxylation, and aromatization reactions. Recently, a wide variety of DA of 2-pyrones have been reported, especially involving coumalic acid and methyl coumalate, two bioderived platform molecules.^19–27^

Meanwhile, Diels–Alder reactions involving nitrogenated 2-pyrones remain far less explored and exhibit notable limitations, particularly the favored formation of the double cycloaddition products, which has only been reported for nitrogenated pyrones.^28–33^ This behavior likely arises from the generation of a highly reactive diene upon decarboxylation of the first DA adduct. Thus, this approach warrants further investigation as a convenient method for accessing novel nitrogen-containing aromatic building blocks including phthalimides. For instance, Kočevar and co-workers^28^ reported the selective synthesis of some phthalimides through the DA reaction of nitrogenated 2-pyrones (Scheme 1c), representing an important contribution to the development of this transformation. However, the reaction proceeded in decalin, which is a high-boiling point solvent, and required a large amount of the catalyst: over 68 equiv of Darco KB, a high-surface activated carbon, were employed. Another example, also studied by Kočevar, explored the DA reaction of fused pyran-2-ones, although the use of precious metals, such as Rh/C, was necessary.^34^

Thereupon, in this work, we developed a new metal-free methodology to produce high-value-added chemicals from nitrogenated 2-pyrones, via a cascade reaction involving Diels–Alder reaction, decarboxylation, and aromatization steps (Scheme 1d). These nitrogenated pyrones can be easily synthesized through *N*-acetylglycine or hippuric acid, both derived from glycine, the simplest stable amino acid which occurs in many proteins and is particularly abundant in silk fibroin, gelatin, and sugar cane.^35^ The aromatization step is mediated by iodine, a mild-reagent,^36^ in catalytic amounts, also efficiently preventing the formation of a competing side product. Different *N*-substituted maleimides as well as some other classical dienophiles were explored. In addition, two examples from chitin-derived pyrones were reported and three Pomalyst analogues were also shown. Remarkably, this methodology also allowed the concise three-step synthesis of the immunomodulatory drug Pomalyst, underscoring its potential for the rapid access to pharmaceutically relevant compounds.

## 2 Results and Discussion

We commenced our study by computationally analyzing the reactivity of three nitrogen-containing pyrones as dienes, 3-acetamido-2-pyrone (3A2P), 3-acetamido-6-methyl-2-pyrone (3A6M2P), and 3-benzamido-6-phenyl-2-pyrone (3B6P2P), toward *N*-ethylmaleimide (NEM) and ethyl vinyl ether (EVE) (Scheme 2). These calculations were used as a guiding tool to rationally select both the diene and the dienophile prior to experimental optimization. Moreover, the selection of these pyrones was motivated by their structural relevance. Notably, 3A2P and 3A6M2P are derived from chitin, the second most abundant biopolymer on Earth, which is found in insect and arthropod exoskeletons, crustacean shells, cell walls, fungi, yeast and organisms in the lower plant and animal kingdoms.^37–41^ Electronically different dienophiles were also evaluated in order to determine if the Diels–Alder reaction could proceed via both normal and inverse electron demand, although the calculations indicated that the normal electron-demand pathway is preferred (see Supporting Information). The observed reactivity can be rationalized based on the HOMO–LUMO energy gaps obtained from Density Functional Theory (DFT) calculations. Among the dienes, 3B6P2P is theoretically predicted to be the most reactive (−3.54 eV), followed by 3A6M2P (−3.69 eV) and 3A2P (−3.91 eV) when *N*-ethylmaleimide is employed as the dienophile. Owing to its electron deficiency, NEM affords a reduced HOMO–LUMO gap, and it was therefore identified as the most suitable partner in the normal demand regime. Guided by these findings, we initiated our exploratory study with the reaction between 3-benzamido-6-phenyl-2-pyrone (**1a**) and *N*-ethylmaleimide (Table 1), systematically evaluating parameters such as solvent, reaction time, temperature, and the number of dienophile equivalents (see Tables S1 and S2 for the full range of conditions tested).

**Scheme 2:**
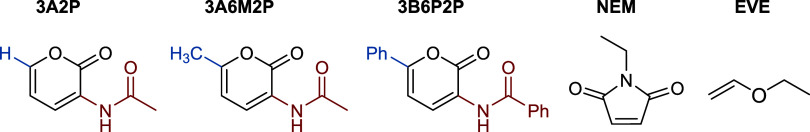
Dienes and Dienophiles Used in Computational Studies for HOMO-LUMO Energy Gap Determination

**Table 1:**
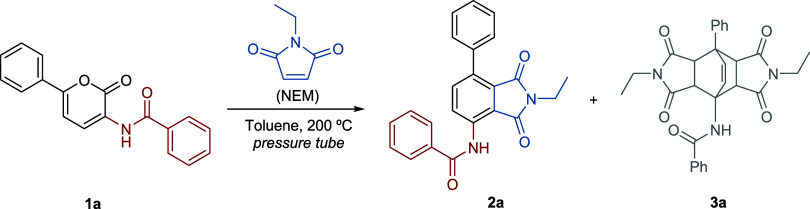
Screening of Reaction Parameters for the Diels-Alder reaction/Decarboxylation/Aromatization Steps

**entry**	**equiv NEM**	**equiv**_**2**_	**time (h)**	**yield 2a** (%)^a^	**yield 3a** (%)^a^
1	3.0	none	15	32	38
2	3.0	1.0	4	83	-
3	3.0	0.5	4	84	-
4	3.0	0.25	4	85	-
5	3.0	0.1	4	82	-
6	3.0	0.05	4	53	22
7	2.0	0.1	4	60	-
8	**2.0**	**0.1**	**15**	**92 (84** ^b^ **)**	-
9	1.0	0.1	15	50	-
10^c^	2.0	0.1	2	76	-
11^c^	2.0	0.1	4	77	-
12^c^	3.0	0.1	2	83	-
13^d^	2.0	0.1	15	77	-
14^e^	2.0	0.1	15	69	-

a Yields and conversion were determined by ^1^H NMR analysis using 1,3,5-Trimethoxybenzene as an internal standard.

b Isolated yield.

c Reaction performed under microwave irradiation.

d Reaction performed at 175 °C.

e Reaction performed at 150 °C.

Inspired by previous works involving the DA reaction of 2-pyrones,^19,21^ we initiated our experiments in toluene at 200 °C, employing 3 equiv of *N*-ethylmaleimide (entry 1). Two products were observed in almost equimolar amounts: the desired aromatic compound (**2a**) and an adduct resulting from two consecutive DA reactions (**3a**). The adduct from the first Diels–Alder could not be isolated since the related reactions occur in cascade. Attempts to suppress the double cycloaddition with bases, Lewis and Brønsted acids, and dehydrogenation catalysts were ineffective (see Table S1), as either the conversion of the starting material remained low or the formation of **2a** still competed heavily with the undesired **3a**. We next examined iodine as an aromatization catalyst. Molecular iodine is inexpensive, widely available, air- and moisture-stable, and nontoxic.^36,42–44^ To our delight, 1.0 equiv of iodine enhanced the conversion of the starting material and, gratifyingly, provided exclusively the aromatized product **2a** in 85% yield after 4 h at 200 °C (entry 2). Furthermore, catalytic amounts were equally efficient down to 0.1 equiv (entries 3 to 5), although further reduction to 0.05 equiv resulted in a mixture of both products (entry 6). Optimization of the dienophile stoichiometry showed that two equivalents of NEM required extended reaction times (15 h), while one equivalent gave poor conversion (entries 7–9). Microwave conditions were also evaluated (entries 10–12) in an attempt to reduce the reaction time; however, although the results were comparable, albeit slightly inferior, a reaction time of 4 h was still required. Therefore, we decided to proceed with conventional heating, as it allows multiple reactions to be carried out simultaneously. Finally, lowering the temperature to 175 or 150 °C (entries 13 and 14) resulted in lower yields. As a result, entry 8 was selected as the standard condition.

To obtain further insights into the experimental results, especially regarding the generation of the side product (**3a**), we computationally analyzed the energy profile of these cascade reactions (Figure 1). First, we calculated the energy barrier for the first DA reaction, which resulted in a Δ*G* ^‡^ value of 39.1 kcal·mol^–1^, whereas the subsequent decarboxylation step required 33.1 kcal·mol^–1^, thereby justifying the need for a high temperature to promote both transformations. Furthermore, considering that the Gibbs activation free energies of each step are in a difference of only 6 kcal·mol^–1^, it is understandable why the respective DA adduct (intermediate **1**) could not be isolated owing to the fact that once formed the decarboxylation should readily take place. Concerning the undesired second DA reaction, this step also presented a high energy barrier (Δ*G* ^‡^ = 39.2 kcal·mol^–1^). We initially considered that both products could be formed, and iodine could help catalyze the retro-Diels–Alder reaction (rDA); however, the Δ*G* ^‡^ value for this rDA step was 53.0 kcal·mol^–1^ and, therefore, the last cycloaddition step is likely not reversible under the applied reaction conditions. Hence, **3a** (product) is not formed in the presence of iodine, which conveniently prevents its formation even at catalytic amounts. This finding explicitly rules out the reversibility of the second Diels–Alder reaction and is fully consistent with the experimental results (Figure S1).

**Figure 1:**
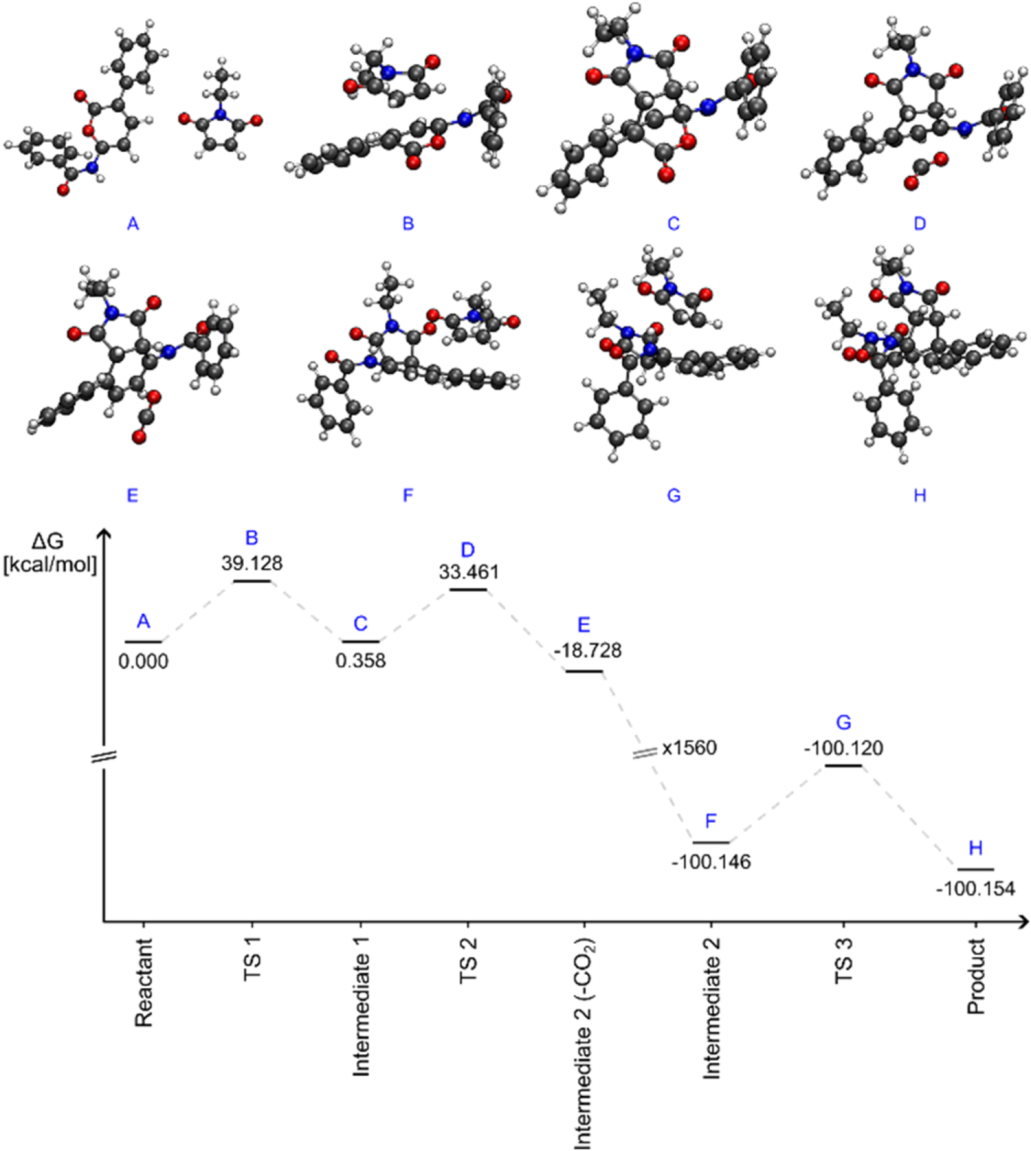
Reaction energy profile for the DA and decarboxylation step as well as for the competitive second cycloaddition.

With the optimal conditions established and an understanding of the energy profile of the reaction, we next explored the substrate scope (Scheme 3) of this iodine-mediated aromatization. To our knowledge, there are no Diels–Alder synthetic methodologies of pyrones involving an iodine-catalyzed aromatization. To this end, we initially investigated eight pyrones bearing distinct C–6 substituents, yielding the products **2a–h**. The synthesis of the pyrones is discussed in the Supporting Information. When phenyl and 2-pyridyl substituted 2-pyrones were evaluated, aromatics **2a** and **2d** were obtained in 84 and 91% yield, respectively. Compound **2a** was also synthesized on a 1 mmol scale to assess the scalability of the reaction under the optimized conditions. Other heteroaromatic substituents, such as 2-furyl and 2-thienyl, yielded the respective aromatics **2b** and **2c** in 72 and 86%. When a *tert*-butyl substituent was employed, the reaction unexpectedly led to the formation of two products (**2e** and **2e′**), one of which was the corresponding dealkylated aromatic. This finding is discussed in more detail in the Supporting Information, where we demonstrated that dealkylation occurs due to the presence of hydroiodic acid (HI) in the reaction medium. Generally, when a *N*-acetyl group was the substituent in C–3 positions (**2f–h**), it resulted in slightly lower yields than when C–3 had a *N*-benzoyl group, as the pyrones starting materials may undergo hydrolysis in the presence of HI, whereas the benzoylamide moiety remains largely unaffected. Aromatics **2g** and **2h** are both synthesized from pyrones that can be obtained from chitin,^45^ thus this method could be further applied for the synthesis of novel building blocks derived from the chitin biomass, thereby contributing to the reduction of dependence on fossil-based resources. Indeed, this is the first example of an aromatic compound synthesized from chitin-derived pyrones.

**Scheme 3:**
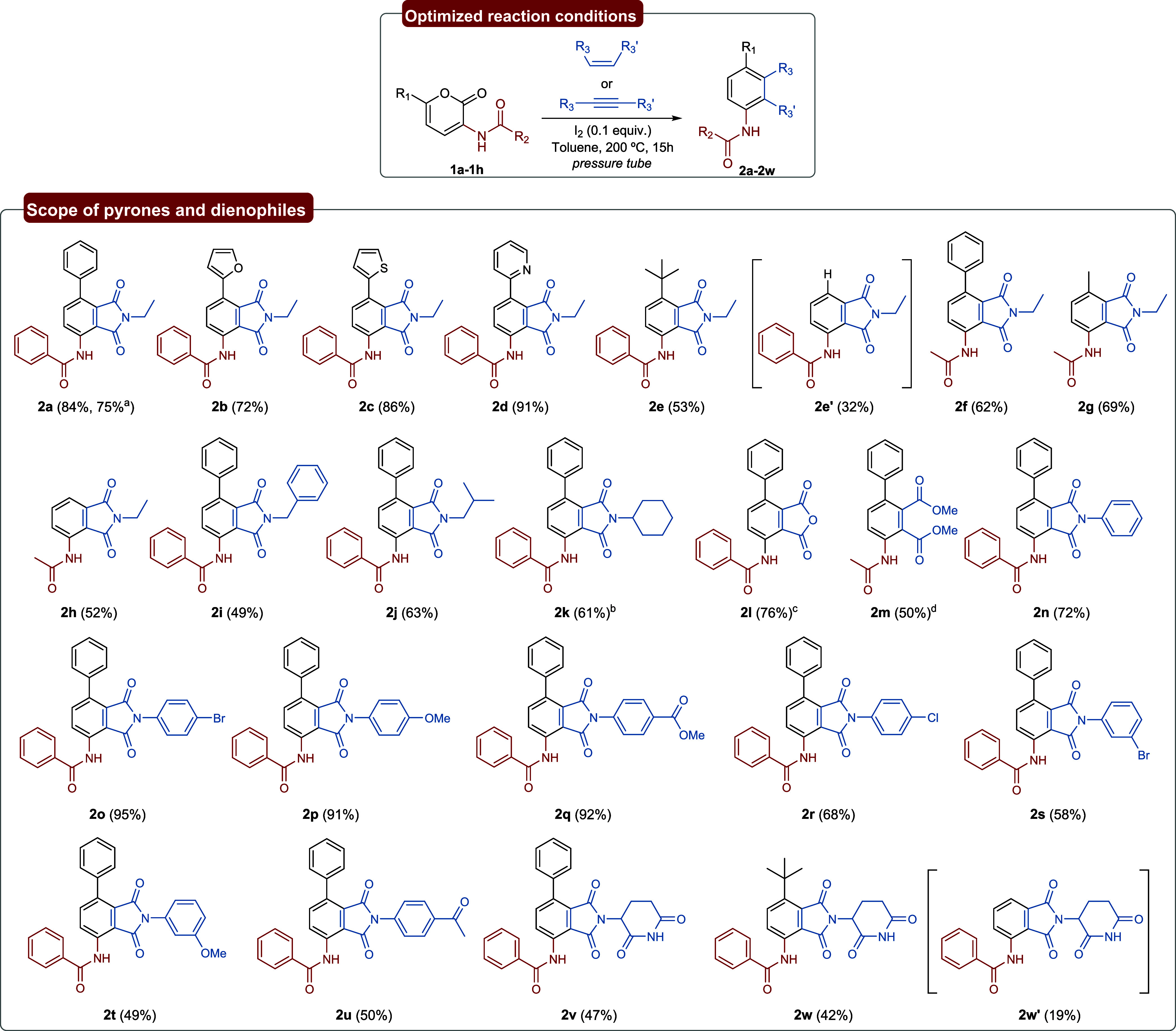
Scope of Aromatic Compounds Obtained from Pyrones **1a–h**

a One mmol scale.
b 1.5 equiv of *N*-cyclohexylmaleimide.
c 0.2 equiv of I_2_.
d 4.0 equiv of dimethyl acetylenedicarboxylate.

Next, this methodology was further extended to a variety of *N*-substituted maleimides, as well as other classical dienophiles (for dienophiles that did not afford the product, see the Supporting Information). Overall, *N*-alkyl maleimides resulted in compounds **2i–k** in lower yields when comparing to *N*-aryl maleimides, likely because the latter are more electron-deficient, which lowers the HOMO–LUMO gap and thereby enhances the reaction efficiency. A wide range of *N*-aryl maleimides (**2n–2u**) successfully afforded the desired product, although substituents in the meta position led to inferior yields (**2s** and **2t**). Maleic anhydride and dimethyl acetylenedicarboxylate resulted in the corresponding aromatics in 76 and 50% yields (**2l** and **2m**), respectively. It should be emphasized that iodine is not required for the formation of compound **2m**, as the competing secondary Diels–Alder reaction is not accessible with this dienophile. However, the catalyst plays a beneficial role in improving the efficiency of the process, as an experiment performed under identical conditions in the absence of iodine resulted in a 38% yield of **2m** (determined by ^1^H NMR analysis). Lastly, three Pomalyst analogues were synthesized from pyrones **1a** and **1e**, yielding the aromatics **2v** and **2w**, besides the respective dealkylated product **2w′**. Moreover, compounds **2a–2m** and **2u–2w** exhibited high fluorescence when subjected to 254 and 365 nm UV light sources. Fluorescence of phthalimides, especially those with amino-substituents in the ring, has been widely reported. This observed characteristic is interesting since fluorescent phthalimide derivatives are known for their biological applications, such as fluorescent markers and fluorophore probes.^46–48^

Afterward, subsequent modifications were investigated to demonstrate the versatility of these aromatic products (Scheme 4). Deacetylation of the chitin-derived compound **2h** in acidic medium provided the corresponding 3-amino derivative **4** in 81% yield. Likewise, deprotection of the benzoylamide moiety of **2e** was also performed under acidic conditions, affording compound **5** in 77% yield. Compound **4** was also accessed in 38% yield through a one-pot, five-step protocol starting from **1e**, in which dealkylation and subsequent hydrolysis were achieved in the presence of hydroiodic acid. Interestingly, compounds **4** and **5** exhibited fluorescence both in solution and in the solid state when subjected to UV light at 365 nm. Finally, the synthesis of Pomalyst, compound **7**, was accomplished through a three-step protocol starting from the chitin-derived pyrone **1h**. Initially, the corresponding aromatic intermediate was obtained through the optimized reaction between maleic anhydride and pyrone **1h**, which was then readily reacted with the corresponding amine, via nucleophilic attack at the carbonyl carbon of the maleic anhydride-derived portion, to afford compound **6** in 32% in two steps. Subsequent hydrolysis of **6** under acidic conditions provided the drug **7** in 45% yield.

**Scheme 4:**
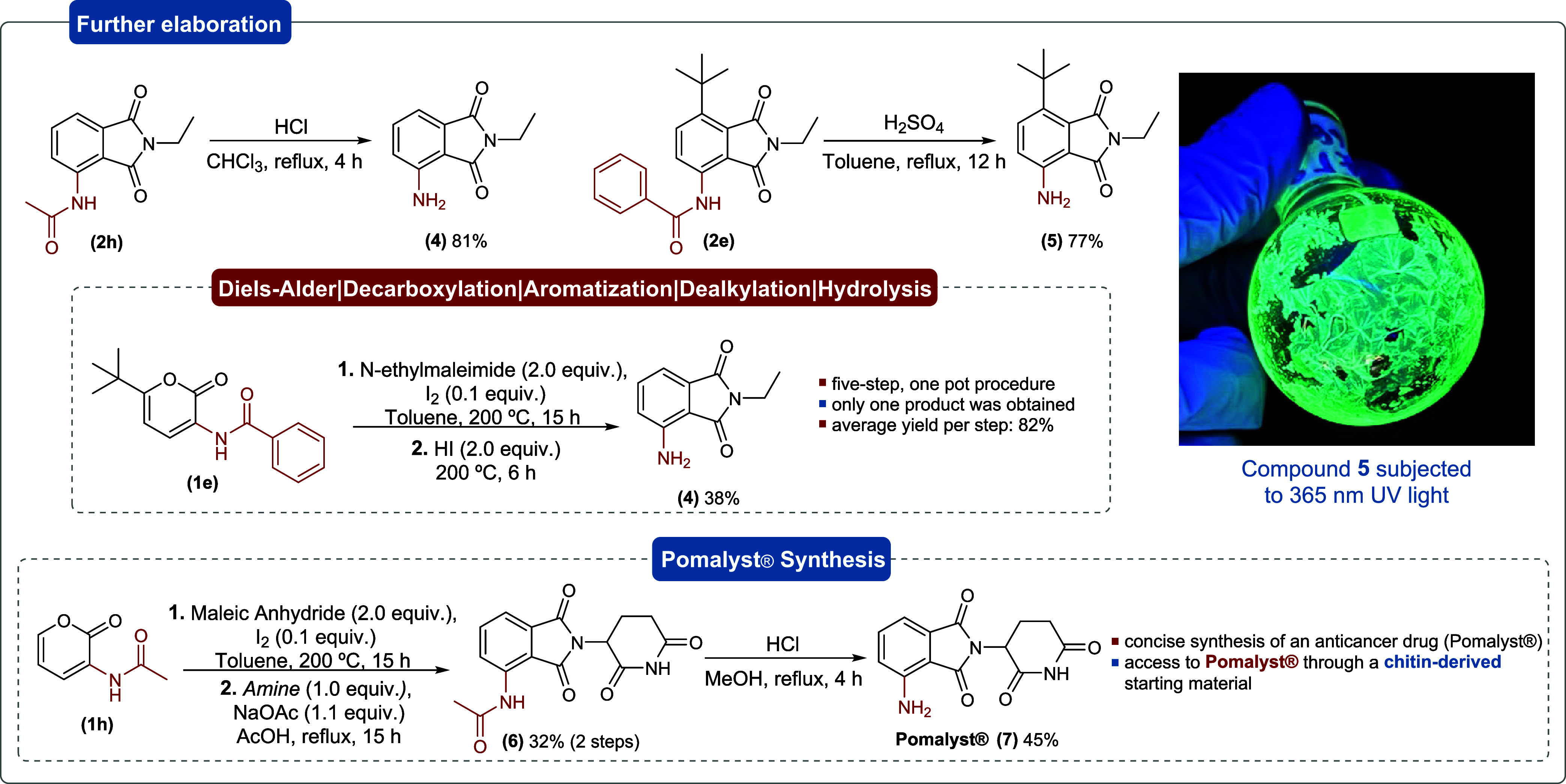
Subsequent Derivatizations to Yield New 3-Aminophthalimides

Regarding the aromatization mechanism and the role of iodine (Scheme 5), we proposed that after the first DA reaction, the decarboxylation should readily proceed, providing the intermediate **III** in the reaction medium. **III** should then promptly react with iodine via iodoiranium species to produce **V**, hampering the second cycloaddition reaction. Subsequently, elimination of a proton would lead to **VI**, and, upon removal of the second hydrogen, it would yield the aromatic **VII**. Meanwhile, as already reported in the literature,^49^ the HI generated during the aromatization process reacts with the excess of *N*-ethylmaleimide, forming the corresponding succinimide and, therefore, regenerating iodine in the reaction medium. Indeed, *N*-ethylsuccinimide was detected in the crude reaction mixture via ^1^H NMR analysis. The addition of an organic base was evaluated in an attempt to neutralize the hydroiodic acid and thereby enable the reaction to proceed with a lower dienophile stoichiometry. However, the presence of a base negatively affected both the conversion of the starting material and the overall yield (see Table S1, entry 20). As this side reaction is unavoidable, an excess of the dienophile is required to ensure full conversion of the pyrone, although it also enables the reaction to proceed under catalytic amounts of iodine.

**Scheme 5:**
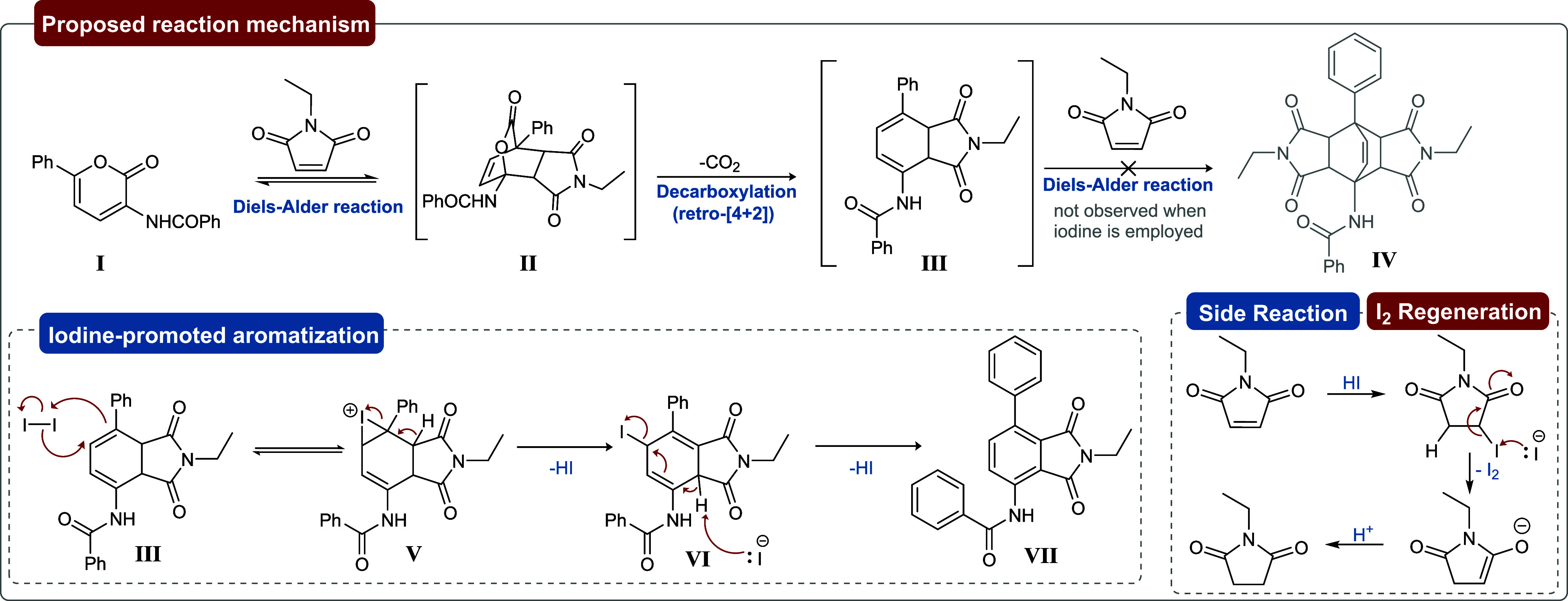
Proposed Reaction Mechanism for the Iodine-Catalyzed Aromatization

## 3 Conclusions

In summary, we have demonstrated the application of iodine as a mild aromatization catalyst for the synthesis of *N*-containing aromatics in which the nitrogen atom is directly attached to the benzene ring, starting from 2-pyrones. This one-pot, metal-free protocol afforded 25 pharmaceutically relevant aromatics, 22 of them novel compounds, in up to 95% yield, the majority of which exhibited fluorescence. The use of iodine, in addition to its role in the aromatization step, improved selectivity, thus guaranteeing higher yields. Additionally, the computational studies were essential for understanding and rationalizing the observed reactivity, and the temperature requirements, indicating that the first Diels–Alder reaction is the most energy demanding step of the process. Finally, the developed methodology was effective in overcoming key limitations concerning the DA reaction of nitrogenated pyrones, such as the use of precious metals and high-boiling point solvents.

## Supplementary Material



## Data Availability

The data underlying this study are available in the published article, in its Supporting Information, and openly available in REDU at 10.25824/redu/CI2IKZ.

## Notes

Experimental details, characterization data, DFT calculations, and NMR spectra for all new compounds (PDF)
