# Supplementary material for: Cascade Iodine-Catalyzed Synthesis of Nitrogenated Aromatics from 2‑Pyrones
Source: J Org Chem. 2026 Mar 31;91(14):5006–14. doi: 10.1021/acs.joc.5c03172 (PMC13077698; doi:10.1021/acs.joc.5c03172)
Supplement: Supplementary file 1 [file jo5c03172_si_001.pdf]

SUPPORTING INFORMATION

Cascade Iodine-Catalyzed Synthesis of Nitrogenated Aromatics from 2-Pyrones

Bruna B. Souza,[a] Tadeu L. G. Cabral,[a] Catarina B. Varriano,[a] Claudio F. Tormena,[a] Julio C. Pastre [a,*]

[a] Institute of Chemistry, Universidade Estadual de Campinas (UNICAMP), 13083-970, Campinas, SP, Brazil
* jpastre@unicamp.br

Table of Contents

General Information……………………………………………………………………..S7

Optimization Study for the Diels-Alder and Aromatization Reaction…………..……………….S8

Study of the reversibility of the second Diels-Alder cycloaddition………………………...S10

Dienophiles tested that did not afford product...S10

Study of the formation of the dealkylated compound 2e’...S11

Procedure A: Synthesis of Pyrones (**1a**–**1g**)...S14

Procedure B: Synthesis of Pyrone **1h**...S14

Procedure C: Synthesis of Aromatic Compounds (**2a**–**2w**)...S14

Procedure C’: Synthesis of Aromatic **2a** – 1 mmol scale...S15

Procedure D: Synthesis of Diels-Alder Adduct **3a**...S15

Procedure E: Synthesis of Compound **4**...S16

Procedure E’: Synthesis of Compound **4** starting from **1e**...S16

Procedure F: Synthesis of Compound **5**..S16

Procedure G: Synthesis of Compound **6**...S17

Procedure H: Synthesis of Compound **7**...S17

Structural Characterization..S18

Computational Studies...S37

HOMO-LUMO Energy gap..S38

Reaction Pathways and Mechanisms...S43

^{1}H and ^{13}C NMR Spectra...S64

References..S140

List of Figures

Figure S1. Evaluation of the second Diels-Alder reaction..**S9**

Figure S2. Dienophiles that failed to give the product..**S10**

Figure S3. Reaction steps (the first Diels-Alder reaction, decarboxylation, and the second Diels-Alder reaction) that were computationally investigated..**S42**

Figure S4. Reaction pathway involving the first Diels-Alder reaction, decarboxylation, and the second Diels-Alder reaction. The pathway includes the isolated reactants (Isol React), the reactant complex (Reac Complex), three transition states (TS1, TS2, TS3), two intermediates (Intermediate 1 and Intermediate 2), and the final product (Product). Energies were computed at the ωB97X-D4/def2-TZVP level with CPCM (toluene). Reported values (ΔG) correspond to Gibbs free energies in kcal/mol..**S43**

Figure S5. Optimized structures of: **A)** and **B)** the isolated reactants, **C)** the reactant complex, **D)** the transition state of the first Diels–Alder reaction, **E)** the first intermediate, **F)** the transition state of the decarboxylation, **G)** the decarboxylation intermediate, **H)** the second intermediate (reactant complex for the second Diels–Alder), **I)** the transition state of the second Diels–Alder reaction, and **J)** the final product..**S44**

Figure S6. Calculated Intrinsic Reaction Coordinate (IRC) profiles for: **A)** the first Diels–Alder reaction, **B)** decarboxylation, and **C)** the second Diels–Alder reaction. All energy values are reported relative to the first point of IRC Path in the first Diels–Alder reaction.......................**S44**

Figure S7. ^{1}H NMR Spectrum (600 MHz, $CDCl_3$) for Pyrone **1a**...**S64**

Figure S8. ^{13}C{^{1}H} NMR Spectrum (150 MHz, $CDCl_3$) for Pyrone **1a**.................................**S65**

Figure S9. ^{1}H NMR Spectrum (500 MHz, $CDCl_3$) for Pyrone **1b**...**S66**

Figure S10. ^{13}C{^{1}H} NMR Spectrum (125 MHz, $CDCl_3$) for Pyrone **1b**...............................**S67**

Figure S11. ^{1}H NMR Spectrum (500 MHz, $CDCl_3$) for Pyrone **1c**...**S68**

Figure S12. ^{13}C{^{1}H} NMR Spectrum (125 MHz, $CDCl_3$) for Pyrone **1c**...............................**S69**

Figure S13. ^{1}H NMR Spectrum (500 MHz, $CDCl_3$) for Pyrone **1d**...**S70**

Figure S14. ^{13}C{^{1}H} NMR Spectrum (125 MHz, $CDCl_3$) for Pyrone **1d**...............................**S71**

Figure S15. ^{1}H NMR Spectrum (600 MHz, $CDCl_3$) for Pyrone **1e**...**S72**

Figure S16. ^{13}C{^{1}H} NMR Spectrum (150 MHz, $CDCl_3$) for Pyrone **1e**...............................**S73**

Figure S17. ^{1}H NMR Spectrum (600 MHz, $CDCl_3$) for Pyrone **1f**..**S74**

Figure S18. ^{13}C{^{1}H} NMR Spectrum (150 MHz, $CDCl_3$) for Pyrone **1f**................................**S75**

Figure S19. ^{1}H NMR Spectrum (500 MHz, $CDCl_3$) for Pyrone **1g**...**S76**

Figure S20. ^{13}C{^{1}H} NMR Spectrum (125 MHz, $CDCl_3$) for Pyrone **1g**...............................**S77**

Figure S21. ^{1}H NMR Spectrum (500 MHz, $CDCl_3$) for Pyrone **1h**..**S78**
Figure S22. $^{13}C\{^{1}H\}$ NMR Spectrum (125 MHz, $CDCl_3$) for Pyrone **1h**................................**S79**
Figure S23. ^{1}H NMR Spectrum (500 MHz, $CDCl_3$) for Aromatic **2a**....................................**S80**
Figure S24. $^{13}C\{^{1}H\}$ NMR Spectrum (125 MHz, $CDCl_3$) for Aromatic **2a**............................**S81**
Figure S25. ^{1}H NMR Spectrum (500 MHz, $CDCl_3$) for Aromatic **2b**....................................**S82**
Figure S26. $^{13}C\{^{1}H\}$ NMR Spectrum (125 MHz, $CDCl_3$) for Aromatic **2b**............................**S83**
Figure S27. ^{1}H NMR Spectrum (500 MHz, $CDCl_3$) for Aromatic **2c**.....................................**S84**
Figure S28. $^{13}C\{^{1}H\}$ NMR Spectrum (125 MHz, $CDCl_3$) for Aromatic **2c**............................**S85**
Figure S29. ^{1}H NMR Spectrum (500 MHz, $CDCl_3$) for Aromatic **2d**....................................**S86**
Figure S30. $^{13}C\{^{1}H\}$ NMR Spectrum (125 MHz, $CDCl_3$) for Aromatic **2d**............................**S87**
Figure S31. ^{1}H NMR Spectrum (500 MHz, $CDCl_3$) for Aromatic **2e**.....................................**S88**
Figure S32. $^{13}C\{^{1}H\}$ NMR Spectrum (125 MHz, $CDCl_3$) for Aromatic **2e**............................**S89**
Figure S33. ^{1}H NMR Spectrum (500 MHz, $CDCl_3$) for Aromatic **2e'**....................................**S90**
Figure S34. $^{13}C\{^{1}H\}$ NMR Spectrum (125 MHz, $CDCl_3$) for Aromatic **2e'**...........................**S91**
Figure S35. ^{1}H NMR Spectrum (500 MHz, $CDCl_3$) for Aromatic **2f**.....................................**S92**
Figure S36. $^{13}C\{^{1}H\}$ NMR Spectrum (125 MHz, $CDCl_3$) for Aromatic **2f**.............................**S93**
Figure S37. ^{1}H NMR Spectrum (500 MHz, $CDCl_3$) for Aromatic **2g**....................................**S94**
Figure S38. $^{13}C\{^{1}H\}$ NMR Spectrum (125 MHz, $CDCl_3$) for Aromatic **2g**............................**S95**
Figure S39. ^{1}H NMR Spectrum (500 MHz, $CDCl_3$) for Aromatic **2h**....................................**S96**
Figure S40. $^{13}C\{^{1}H\}$ NMR Spectrum (125 MHz, $CDCl_3$) for Aromatic **2h**............................**S97**
Figure S41. ^{1}H NMR Spectrum (500 MHz, $CDCl_3$) for Aromatic **2i**......................................**S98**
Figure S42. $^{13}C\{^{1}H\}$ NMR Spectrum (125 MHz, $CDCl_3$) for Aromatic **2i**.............................**S99**
Figure S43. ^{1}H NMR Spectrum (500 MHz, $CDCl_3$) for Aromatic **2j**...................................**S100**
Figure S44. $^{13}C\{^{1}H\}$ NMR Spectrum (125 MHz, $CDCl_3$) for Aromatic **2j**...........................**S101**
Figure S45. ^{1}H NMR Spectrum (500 MHz, $CDCl_3$) for Aromatic **2k**..................................**S102**
Figure S46. $^{13}C\{^{1}H\}$ NMR Spectrum (125 MHz, $CDCl_3$) for Aromatic **2k**..........................**S103**
Figure S47. ^{1}H NMR Spectrum (600 MHz, $CDCl_3$) for Aromatic **2l**...................................**S104**
Figure S48. $^{13}C\{^{1}H\}$ NMR Spectrum (150 MHz, $CDCl_3$) for Aromatic **2l**...........................**S105**
Figure S49. ^{1}H NMR Spectrum (500 MHz, $CDCl_3$) for Aromatic **2m**.................................**S106**
Figure S50. $^{13}C\{^{1}H\}$ NMR Spectrum (125 MHz, $CDCl_3$) for Aromatic **2m**.........................**S107**
Figure S51. ^{1}H NMR Spectrum (600 MHz, $CDCl_3$) for Aromatic **2n**..................................**S108**
Figure S52. $^{13}C\{^{1}H\}$ NMR Spectrum (150 MHz, $CDCl_3$) for Aromatic **2n**..........................**S109**
Figure S53. ^{1}H NMR Spectrum (500 MHz, $CDCl_3$) for Aromatic **2o**..................................**S110**

Figure S54. $^{13}C\{^{1}H\}$ NMR Spectrum (125 MHz, $CDCl_3$) for Aromatic **2o**........................... **S111**
Figure S55. ^{1}H NMR Spectrum (500 MHz, $CDCl_3$) for Aromatic **2p**................................... **S112**
Figure S56. $^{13}C\{^{1}H\}$ NMR Spectrum (125 MHz, $CDCl_3$) for Aromatic **2p**........................... **S113**
Figure S57. ^{1}H NMR Spectrum (600 MHz, $CDCl_3$) for Aromatic **2q**................................... **S114**
Figure S58. $^{13}C\{^{1}H\}$ NMR Spectrum (150 MHz, $CDCl_3$) for Aromatic **2q**........................... **S115**
Figure S59. ^{1}H NMR Spectrum (500 MHz, $CDCl_3$) for Aromatic **2r**.................................... **S116**
Figure S60. $^{13}C\{^{1}H\}$ NMR Spectrum (125 MHz, $CDCl_3$) for Aromatic **2r**............................ **S117**
Figure S61. ^{1}H NMR Spectrum (500 MHz, $CDCl_3$) for Aromatic **2s**................................... **S118**
Figure S62. $^{13}C\{^{1}H\}$ NMR Spectrum (125 MHz, $CDCl_3$) for Aromatic **2s**............................ **S119**
Figure S63. ^{1}H NMR Spectrum (500 MHz, $CDCl_3$) for Aromatic **2t**................................... **S120**
Figure S64. $^{13}C\{^{1}H\}$ NMR Spectrum (125 MHz, $CDCl_3$) for Aromatic **2t**............................ **S121**
Figure S65. ^{1}H NMR Spectrum (500 MHz, $CDCl_3$) for Aromatic **2u**................................... **S122**
Figure S66. $^{13}C\{^{1}H\}$ NMR Spectrum (125 MHz, $CDCl_3$) for Aromatic **2u**........................... **S123**
Figure S67. ^{1}H NMR Spectrum (600 MHz, $CDCl_3$) for Aromatic **2v**................................... **S124**
Figure S68. $^{13}C\{^{1}H\}$ NMR Spectrum (150 MHz, $CDCl_3$) for Aromatic **2v**........................... **S125**
Figure S69. ^{1}H NMR Spectrum (600 MHz, $CDCl_3$) for Aromatic **2w**.................................. **S126**
Figure S70. $^{13}C\{^{1}H\}$ NMR Spectrum (150 MHz, $CDCl_3$) for Aromatic **2w**.......................... **S127**
Figure S71. ^{1}H NMR Spectrum (600 MHz, $CDCl_3$) for Aromatic **2w’**................................. **S128**
Figure S72. $^{13}C\{^{1}H\}$ NMR Spectrum (150 MHz, $CDCl_3$) for Aromatic **2w’**......................... **S129**
Figure S73. ^{1}H NMR Spectrum (600 MHz, $CDCl_3$) for Adduct **3a**....................................... **S130**
Figure S74. $^{13}C\{^{1}H\}$ NMR Spectrum (150 MHz, $CDCl_3$) for Adduct **3a**............................. **S131**
Figure S75. ^{1}H NMR Spectrum (500 MHz, $CDCl_3$) for Compound **4**.................................. **S132**
Figure S76. $^{13}C\{^{1}H\}$ NMR Spectrum (125 MHz, $CDCl_3$) for Compound **4**.......................... **S133**
Figure S77. ^{1}H NMR Spectrum (600 MHz, $CDCl_3$) for Compound **5**.................................. **S134**
Figure S78. $^{13}C\{^{1}H\}$ NMR Spectrum (150 MHz, $CDCl_3$) for Compound **5**.......................... **S135**
Figure S75. ^{1}H NMR Spectrum (600 MHz, DMSO-d_6) for Compound **6**............................. **S136**
Figure S76. $^{13}C\{^{1}H\}$ NMR Spectrum (150 MHz, DMSO-d_6) for Compound **6**..................... **S137**
Figure S77. ^{1}H NMR Spectrum (600 MHz, DMSO-d_6) for Compound **7**............................. **S138**
Figure S78. $^{13}C\{^{1}H\}$ NMR Spectrum (150 MHz, DMSO-d_6) for Compound **7**..................... **S139**

List of Tables

Table S1. Screening of additives for the Diels-Alder and Aromatization reaction.....................**S7**

Table S2. Screening of reaction parameters for the Diels-Alder and Aromatization steps.........**S8**

Table S3. Study of the Diels-Alder and Aromatization reaction starting with pyrone **1e**..**S11**

Table S4. Screening of acids aiming to promote dealkylation of subtract **2e**............................**S12**

Table S5. Boltzmann-weighted HOMO energies (eV) of the dienes and LUMO energies (eV) of the dienophiles...**S27**

Table S6. The HOMO-LUMO energy gap (eV) calculated between the HOMO of each diene and the LUMO of the dienophiles **EVE** and **NEM**...**S38**

Table S7. Electronic energy ($E_{electronic}$), thermostatistical contributions (G_{rrho}), and total Gibbs free energies (G_{total}) for each conformation of each compound. All values are given in Hartree..**S38**

Table S8. Gibbs activation free energies ($\Delta\Delta G^{\ddagger} = \Delta G_{TS} - \Delta G_{react}$) and reaction free energies ($\Delta\Delta G = \Delta G_{prod} - \Delta G_{react}$) for each step of the reaction pathway. All values are given in kcal·mol^{-1}..**S43**

General Information

All commercial chemicals and solvents were purchased and used as received without further purification unless otherwise stated. The synthesis of maleimides were reproduced according to literature procedures.[1] All spectral data obtained for known compounds were in agreement with the ones reported in the literature. Thin layer chromatography (TLC) was carried out in aluminium sheets with silica gel 60 matrix and fluorescent indicator at 254 nm. Visualization of spots were performed with UV irradiation at 254 nm. Purifications of compounds were performed with silica gel at 60 Å pore size and 40−63 μm particle size in Biotage Selekt flash chromatography system. Experiments under microwave irradiation were carried out using a Biotage Initiator Plus microwave reactor. Melting point (mp) was determined on Mettler Toledo MP50 automated melting point system. Melting point values are reported as Celsius temperature values (°C) and are uncorrected. Infrared (FTIR) was recorded on Agilent Cary 630 spectrometer using a diamond ATR sampling accessory. Wavenumbers of infrared bands are reported as $\tilde{\nu}$ values (cm^{-1}). Nuclear magnetic resonance (NMR) was recorded on Bruker Avance 500 MHz or 600 MHz spectrometer operating at 500 or 600 MHz (^{1}H nuclei) and 125 or 150 MHz (^{13}C nuclei) frequencies at 293 K, respectively. Chemical shifts are reported as δ values (ppm) referenced to the signal of chloroform (^{1}H: 7.26 ppm, ^{13}C: 77.2 ppm) or dimethyl sulfoxide (^{1}H: 2.50 ppm, ^{13}C: 39.5 ppm). Multiplicities were given as "s" (singlet), "d" (doublet), "dd" (doublet of doublets), "ddd" (doublet of doublets of doublets), "t" (triplet), "q" (quartet) and "m" (multiplet). Coupling constants (J) are reported in Hertz (Hz). Samples were prepared in deuterated solvent immediately before spectral acquisition. High-resolution mass spectrometry (HRMS) was carried out on Agilent Q-TOF 6545 mass spectrometer equipped with an electrospray source at 298 K for novel compounds. Molecular ions are reported as m/z values. Average mass errors are reported as $|\Delta m/z|$ values (ppm) related to the mass difference between calculated and experimental protonated molecular ion $[M+H]^{+}$ masses. Samples were prepared in HPLC gradient grade acetonitrile or a mixture of acetonitrile and tetrahydrofuran (1:1) immediately before experimental acquisition.

Optimization Study for the Diels-Alder and Aromatization Reaction

Screening of additives

To a 5 mL glass sealed tube, pyrone **1a** (0.1 mmol, 1.0 equiv.), *N*-ethylmaleimide (x mmol, x equiv.), additive (x mmol, x equiv.), toluene (0.5 mL) and a magnetic stir bar were added. The reaction was heated at 200 ºC for 4 or 15h. The starting material conversion and the yield of the following reaction were determined by ^{1}H NMR analysis using 1,3,5-Trimethoxybenzene as an internal standard.

Table S1. Screening of additives for the Diels-Alder and Aromatization reaction.

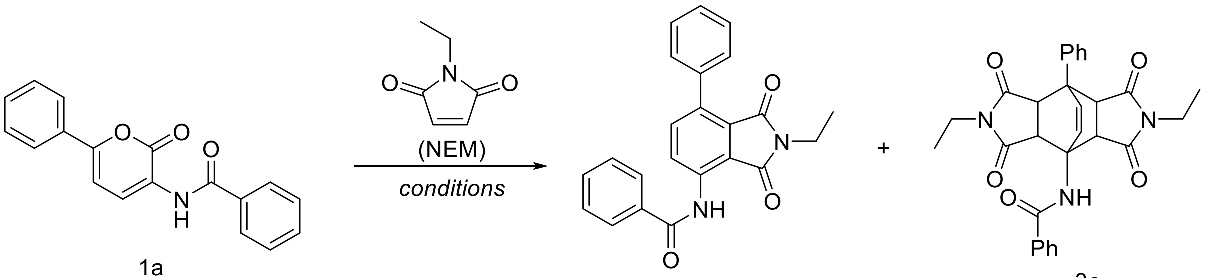

Entry	NEM (equiv.)	Additive (equiv.)	Time (h)	SM Conversion (%)	Yield 2a (%)	Yield 3a (%)
**1**	3.0	None	15	77	32	38
**2**	2.0	None	15	64	23	31
**3**	1.0	None	15	65	14	40
**4**	5.0	None	15	100	-	94[a]
**5**	3.0	Pd/C (0.05 equiv.)	15	92	10	68
**6**	2.0	Pd/C (0.05 equiv.)	15	88	24	56
**7[b]**	3.0	DABCO (1.0 equiv.)	15	47	26	-
**8[b]**	3.0	DABCO (2.0 equiv.)	15	51	30	-
**9[b]**	3.0	H_2SO_4 (0.2 equiv),	15	100	58	37
**10[b]**	2.0	H_2SO_4 (0.2 equiv)	15	80	45	25
**11**	3.0	H_2SO_4 (0.2 equiv)	15	100	47	-
**12**	3.0	DDQ (1.0 equiv.)	15	95	30	56

**13**	3.0	Activated charcoal (50.0 equiv.)	15	100	12	83
**14**	3.0	$ZnCl_2$ (0.2 equiv.)	15	95	6	37
**15**	3.0	I_2 (3.0 equiv.)	4	100	72	-
**16**	3.0	I_2 (2.0 equiv.)	4	100	70	-
**17**	3.0	I_2 (1.0 equiv.)	4	100	83	-
**18**	2.0	I_2 (1.0 equiv.)	4	90	75	-
**19**	1.0	I_2 (1.0 equiv.)	4	58	45	-
**20**	1.0	I_2 (2.0 equiv.), DABCO (1.2 equiv.)	15	62	48	-
**21**	3.0	I_2 (0.5 equiv.)	4	100	84	-
**22**	3.0	I_2 (0.25 equiv.)	4	100	85	-
**23**	**3.0**	**I_2 (0.1 equiv.)**	**4**	**100**	**82**	**-**
**24**	3.0	I_2 (0.05 equiv.)	4	100	53	22
**25**	2.0	HI (0.1 equiv.)	15	100	41	-

a: isolated yield; b: reaction performed in acetonitrile at 150 ºC;

Screening of reaction parameters

To a 5 mL glass sealed tube, pyrone **1a** (0.1 mmol, 1.0 equiv.), *N*-ethylmaleimide (x mmol, x equiv.), iodine (0.01 mmol, 0.1 equiv.), solvent (0.5 mL) and a magnetic stir bar were added. The reaction was heated at the indicated temperature for the indicated time. The starting material conversion and the yield of the following reaction were determined by ^{1}H NMR analysis using 1,3,5-Trimethoxybenzene as an internal standard. Temperature, time, solvent and dienophile's number of equivalents were evaluated.

Table S2. Screening of reaction parameters for the Diels-Alder and Aromatization steps.

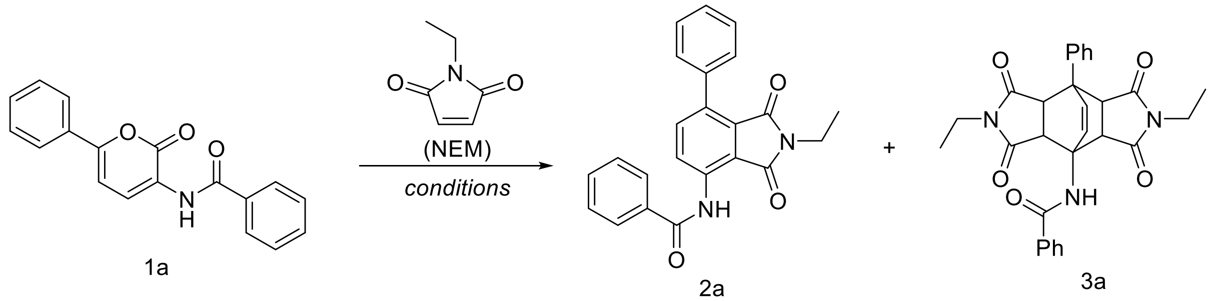

Entry	Solvent	NEM (equiv.)	Temperature (ºC)	Time (h)	SM Conversion (%)	Yield 2a (%)	Yield 3a (%)
**1**	Toluene	3.0	200	4	100	82	-
**2**	Xylenes	3.0	200	4	100	91	-
**3**	Chlorobenzene	3.0	200	4	100	85	-
**4**	Toluene	2.0	200	4	72	60	-
**5**	Toluene	2.0	200	8	94	82	-
6	**Toluene**	**2.0**	**200**	**15**	**100**	**92(84[a])**	**-**
**7**	Toluene	1.5	200	15	81	76	-
**8**	Toluene	1.0	200	15	62	50	-
**9**	Xylenes	2.0	200	15	100	89	-
**10**	Toluene	2.0	175	15	95	77	-
**11**	Toluene	2.0	150	15	83	69	-
**12[b]**	Toluene	2.0	200	1	73	62	-
**13[b]**	Toluene	2.0	200	2	86	76	-
**14[b]**	Toluene	2.0	200	4	95	77	-
**15[b]**	Toluene	3.0	200	1	87	81	-
**16[b]**	Toluene	3.0	200	2	96	83	-

a: isolated yield; b: reaction performed under microwave irradiation.

Study of the reversibility of the second Diels-Alder cycloaddition

This experiment was performed to study the reversibility of the second Diels-Alder reaction at the standard conditions, in order to evaluate if the adduct **3a** could be formed in the reaction and eventually be converted to the desired aromatic compound **2a**.

To a 5 mL glass sealed tube, adduct **3a** (0.1 mmol, 1.0 equiv.), iodine (0.1 mmol, 0.1 equiv.), toluene (0.5 mL) and a magnetic stir bar were added. The reaction was heated at 200 ºC for 15h. The starting material conversion and the yield of the following reaction were determined by ^{1}H NMR analysis using 1,3,5-Trimethoxybenzene as an internal standard. No reaction was observed and the starting material **3a** was entirely recovered.

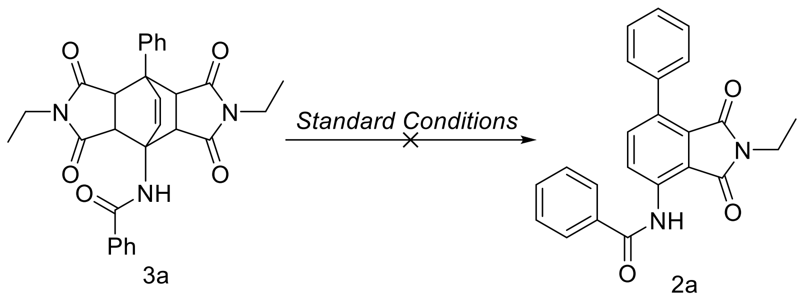

Figure S1. Evaluation of the second Diels-Alder reaction.

Dienophiles tested that did not afford product

Various common dienophiles, more specifically maleimide, acrylonitrile, ethyl vinyl ether, crotonic acid, dimethyl maleate and benzoquinone, were also tested, none of which led to product formation, as either no reaction was observed (NR) or the conversion of the starting material was significantly low (traces).

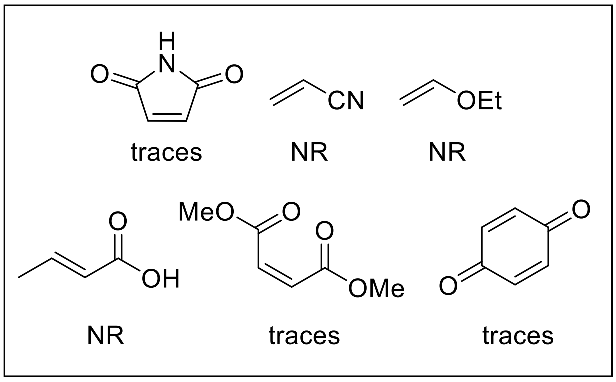

Figure S2. Dienophiles that failed to give the product.

Study of the formation of the dealkylated compound 2e'

We aimed to study the formation of the dealkylated compound **2e'**, obtained along with product **2e** when pyrone **1e** is subject to standard reaction conditions. We thus proceeded to evaluate other conditions for the Diels-Alder, decarboxylation and aromatization of substrate **1e**, in order to understand if the formation of dealkylated product **2e** is somehow related to the presence of iodine. To a 5 mL glass sealed tube, pyrone 1e (0.1 mmol, 1.0 equiv.), N-ethylmaleimide (0.2 mmol, 2.0 equiv.), additive (x mmol, x equiv.), toluene (0.5 mL) and a magnetic stir bar were added. The reaction was heated at 150 or 200 ºC for 15h. The starting material conversion and the yield of the following reaction were determined by ^{1}H NMR analysis using 1,3,5-Trimethoxybenzene as an internal standard. Initially, we evaluated the addition of iodine and an acid (entry 2), to observe whether the formation of compound **2e'** would increase, which did

not occur. We also decided to analyze the addition of iodine and a base – as the base could interact in an acid-base reaction with HI and thus prevent the formation of 2e' – if its formation was associated with the presence of hydriodic acid. When DBU was employed (entries 3 and 4), it inhibited the reaction and none of the products were obtained, as for DABCO (entries 6 and 8), both products were still observed. When other additives were employed without the presence of iodine, such as DABCO and $ZnCl_2$, only the alkylated **2e** was observed. Therefore, the formation of **2e'** is associated with the presence of iodine in the reaction medium.

Table S3. Study of the Diels-Alder and Aromatization reaction starting with pyrone **1e**.

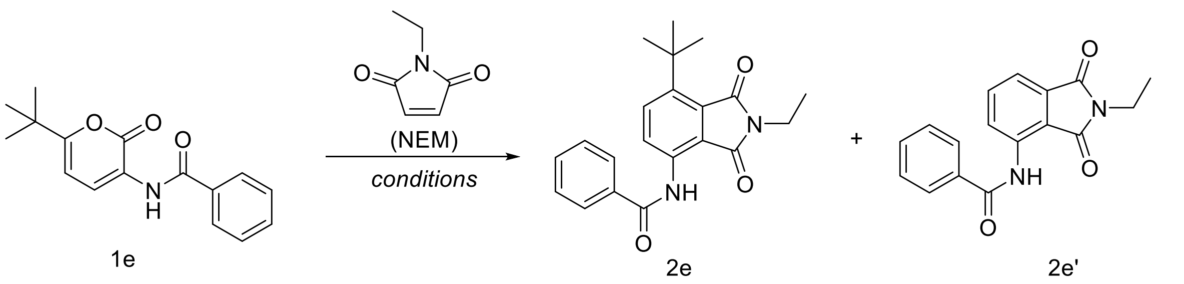

Entry	Additive (equiv.)	Temperature (ºC)	SM Conversion (%)	Yield 2e (%)	Yield 2e' (%)
**1**	I_2 (0.1)	200	92[a]	53[a]	32[a]
**2**	I_2 (0.1) + HCl (0.1)	200	95	44	29
**3**	I_2 (0.1) + DBU (1.0)	200	34	-	-
**4**	I_2 (0.1) + DBU (0.1)	200	30	-	-
**5**	DABCO (1.0)[b]	150	28	10	-
**6**	I_2 (0.1), DABCO (0.1)	200	49	23	15
**7**	I_2 (0.1)	150	60	42	17
**8**	I_2 (0.1) + DABCO (0.5)	200	48	11	traces
**9**[c]	$ZnCl_2$ (0.2)	200	80	7	-
**10**	I_2 (0.1) + HI (0.5)	200	31	-	-

a: isolated yields; b: reaction performed in acetonitrile; c: adduct from double Diels-Alder was also obtained in 10% yield.

Based on previous reports of de-*tert*-butylation of aromatics occurring under acidic conditions,[2–4] we assumed that the dealkylation could take place after the aromatization reaction, due to the presence of hydroiodic acid (HI), generated in the reaction medium. Since there are no reports linking a dealkylation to hydroiodic acid, we decided to test other known conditions for the de-*tert*-butylation of aromatic compounds, subjecting the aromatic **2e** to a series of attempts, although the dealkylated product could not be obtained in any of the tested

conditions. Both Lewis and Brønsted acids were employed and no conversion of starting material was observed (entries 1, 2 and 4), except sulfuric acid (entry 5), which led to the formation of the corresponding deprotected aromatic – with a free NH_2 group – and phosphoric acid, that led to degradation of the starting material. However, when 2 equivalents of hydriodic acid (entry 6) were employed, indeed the dealkylation took place and compound **2e'** (dealkylated) was obtained, along with compound **4** (dealkylated and hydrolyzed).

Table S4. Screening of acids aiming to promote dealkylation of subtract **2e**.

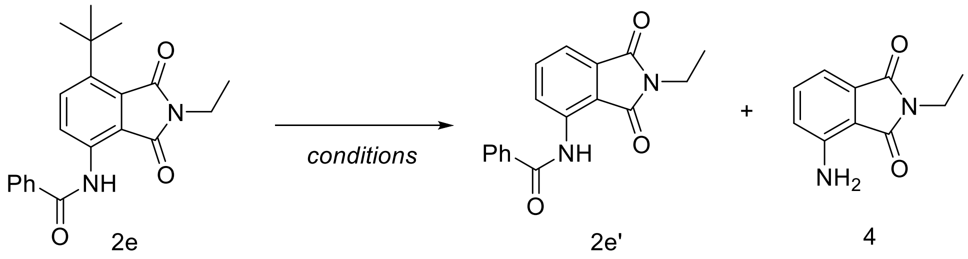

Entry	Solvent	Additive (equiv.)	Temperature (ºC)	Time (h)	Yield 2e'/4 (%)
**1**	Toluene	$AlCl_3$ (5.0)	r.t	6	- / -
**2**	Toluene	$AlCl_3$ (5.0)	Reflux	12	- / -
**3**	-	H_3PO_4[a]	200	6	- / -
**4**	Toluene	HCl (0.3)	200	12	- / -
**5**	-	H_2SO_4[a]	65	6	- / -
**6**	Toluene	HI (2.0)	200	15	23 / 47

a: 0.5 mL of the corresponding acid was added.

Considering the experimental results, we suggested that the dealkylation occurs after the aromatization reaction, as explicit in Scheme 1.

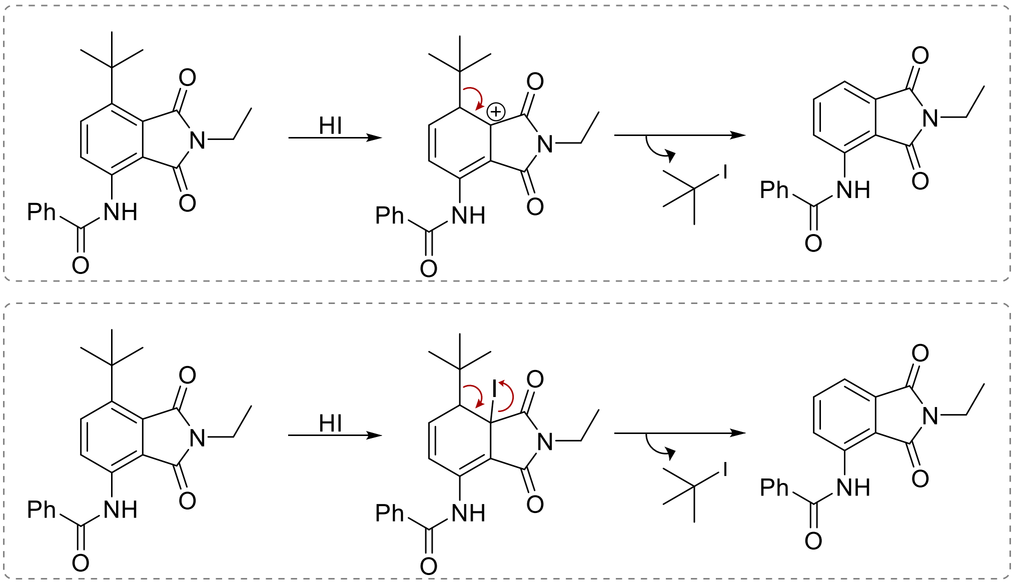

Scheme S1. Proposed mechanisms for the dealkylation step in the presence of hydriodic acid.

Experimental Procedures

Procedure A: Synthesis of Pyrones (1a to 1g)[5]

Pyrones were prepared according to literature procedure.[5] To a 25 mL round-bottom flask were added the corresponding ketone (2 mmol, 1.0 equiv.) and *N,N*-dimethylformamide dimethyl acetal (477.0 mg, 4 mmol, 2.0 equiv.). The reaction mixture was refluxed for 4 h using a heating block. Then, the volatile components were evaporated and the obtained residue was used without any further purification in the next step. The enaminone (1 mmol, 1.0 equiv.), synthesized in the previous step, was diluted in acetic anhydride (1.25 mL) and either *N*-acetylglycine or hippuric acid (1 mmol, 1.0 equiv.) was added. The mixture was heated at 90 ºC for 4 h using a heating block. Acetic anhydride was removed in rotary evaporator under reduced pressure and the reaction mixture was purified by either method A or B.

Method A: Cooled ethanol (0.5 mL) was added to the crude residue and the formation of a precipitate was observed. The solid was filtered, washed with cooled ethanol and dried in a high vacuum system.

Method B: The resulting crude residue was purified by flash column chromatography using hexane/ethyl acetate 15–70% mixture and silica gel (25 g). The fractions were combined, evaporated in rotary evaporator under reduced pressure and dried in a high vacuum system.

Procedure B: Synthesis of Pyrone 1h

To a 25 mL round bottom flask, 3-(dimethylamino)propenal (99.1 mg, 1 mmol, 1.0 equiv.), *N*-acetylglycine (117.0 mg, 1 mmol, 1.0 equiv.), acetic anhydride (1.25 mL) and a magnetic stir bar were added. The reaction mixture was refluxed for four hours using an appropriate heating block. Acetic anhydride was removed in rotary evaporator under reduced pressure and the resulting crude residue was purified by flash column chromatography using hexane/ethyl acetate 0–60% mixture and silica gel (50 g).

Procedure C: Synthesis of Aromatic Compounds (2a to 2v)

To a 5 mL glass sealed tube under air atmosphere, pyrone (0.1 mmol, 1.0 equiv.), dienophile (0.2 mmol, 2.0 equiv.), iodine (0.01 mmol, 0.1 equiv.), toluene (0.5 mL) and a magnetic stir bar were added. The reaction was heated at 200 ºC for 15 h in a heating block. After checking the formation of the product by TLC analysis using hexane/ethyl acetate 20% mixture as mobile phase, the crude residue was purified by either method A or B.

Method A: The reaction mixture was allowed to warm to room temperature and was then diluted in ethyl acetate or dichloromethane and washed with a 0.1 M solution of sodium thiosulfate (10 mL) and a saturated sodium bicarbonate solution (10 mL). The organic layer was dried over magnesium sulfate and the solvent was removed in rotary evaporator under reduced pressure. Unless otherwise stated, the resulting crude residue was purified by flash column chromatography using hexane/ethyl acetate 0–40% mixture and silica gel (10 g). The fractions were combined, evaporated in rotary evaporator under reduced pressure and dried in a high vacuum system.

Method B: The reaction mixture was allowed to warm to room temperature and the formation of a precipitate was observed. The solid was filtered, washed with hexane and dried in a high vacuum system. If the product was still detected in the filtrate by TLC, the solvent was evaporated and recrystallized.

Procedure C': Synthesis of Aromatic 2a – 1 mmol scale

To a 20 mL glass sealed tube under air atmosphere, pyrone (1 mmol, 1.0 equiv.), *N*-ethylmaleimide (2 mmol, 2.0 equiv.), iodine (0.1 mmol, 0.1 equiv.), toluene (5 mL) and a magnetic stir bar were added. The reaction was heated at 200 ºC for 15 h in a heating block. The reaction mixture was allowed to warm to room temperature and was then diluted in dichloromethane (100 mL) and washed with a 0.1 M solution of sodium thiosulfate (100 mL) and a saturated sodium bicarbonate solution (100 mL). The organic layer was dried over

magnesium sulfate and the solvent was removed in rotary evaporator under reduced pressure. The resulting crude residue was purified by flash column chromatography using hexane/ethyl acetate 0–40% mixture and silica gel (25 g). The fractions were combined, evaporated in rotary evaporator under reduced pressure and dried in a high vacuum system. Yield: 75% (279 mg, 0.754 mmol).

Procedure D: Synthesis of Diels-Alder Adduct 3a

To a 5 mL glass sealed tube under air atmosphere, pyrone (0.1 mmol, 1.0 equiv.), *N*-ethylmaleimide (0.5 mmol, 5.0 equiv.), toluene (0.5 mL) and a magnetic stir bar were added. The reaction was heated at 200 ºC for 15 h in a heating block. The reaction mixture was allowed to warm to room temperature and the solvent was removed in rotary evaporator under reduced pressure. The resulting crude residue was purified by flash column chromatography using hexane/ethyl acetate 30–70% mixture and silica gel (10 g). The fractions were combined, evaporated in rotary evaporator under reduced pressure and dried in a high vacuum system.

Procedure E: Synthesis of Compound 4

To a 5 mL glass sealed tube under air atmosphere, compound 2h (0.116 mmol, 1.0 equiv.), concentrated HCl (70 μL), chloroform (0.25 mL) and a magnetic stir bar were added. The reaction was heated up at reflux for 4 h using a heating block. After checking the formation of the product by TLC analysis using hexane/ethyl acetate 20% mixture as mobile phase, the reaction mixture was quenched with $NaHCO_3$ saturated solution (5 mL) and extracted with ethyl acetate (3 x 10 mL). The organic layers were combined, dried over magnesium sulfate and the solvent was removed in rotary evaporator under reduced pressure. The resulting crude residue was purified by flash column chromatography using hexane/ethyl acetate 20–60% mixture and silica gel (10 g). The fractions were combined, evaporated in rotary evaporator under reduced pressure and dried in a high vacuum system. The product (yellow solid) was obtained in 81% yield (18.0 mg, 0.095 mmol).

Procedure E': Synthesis of Compound 4 starting from 1e

To a 5 mL glass sealed tube under air atmosphere, pyrone (0.1 mmol, 1.0 equiv.), dienophile (0.2 mmol, 2.0 equiv.), iodine (0.01 mmol, 0.1 equiv.), toluene (0.5 mL) and a magnetic stir bar were added. The reaction was heated at 200 ºC for 15 h in a heating block. After 15 h, the vial was opened and hydriodic acid was added (0.2 mmol, 2.0 equiv.), the reaction proceeded to be heated at 200 ºC for 6 h. The reaction mixture was allowed to warm to room temperature and

was then diluted in ethyl acetate and washed with a 0.1 M solution of sodium thiosulfate (10 mL) and a saturated sodium bicarbonate solution (10 mL). The organic layer was dried over magnesium sulfate and the solvent was removed in rotary evaporator under reduced pressure. The resulting crude residue was purified by flash column chromatography using hexane/ethyl acetate 0–40% mixture and silica gel (10 g). The fractions were combined, evaporated in rotary evaporator under reduced pressure and dried in a high vacuum system.

Procedure F: Synthesis of Compound 5

To a 5 mL glass sealed tube under air atmosphere, compound 2e (0.045 mmol, 1.0 equiv.), concentrated H_2SO_4 (20 μL), toluene (0.5 mL) and a magnetic stir bar were added. The reaction was heated up at reflux for 12 h in a heating block. After checking the formation of the product by TLC analysis using hexane/ethyl acetate 20% mixture as mobile phase, the reaction mixture was quenched with $NaHCO_3$ saturated solution (5 mL) and extracted with ethyl acetate (3 x 10 mL). The organic layers were combined, dried over magnesium sulfate and the solvent was removed in rotary evaporator under reduced pressure. The resulting crude residue was purified by flash column chromatography using hexane/ethyl acetate 20–60% mixture and silica gel (10 g). The fractions were combined, evaporated in rotary evaporator under reduced pressure and dried in a high vacuum system. The product (yellow solid) was obtained in 77% yield (8.6 mg, 0.035 mmol).

Procedure G: Synthesis of Compound 6

To a 5 mL glass sealed tube under air atmosphere, pyrone 1h (15.3 mg, 0.1 mmol, 1.0 equiv.), maleic anhydride (19.6 mg, 0.2 mmol, 2.0 equiv.), iodine (5.1 mg, 0.02 mmol, 0.2 equiv.), toluene (0.5 mL), and a magnetic stir bar were added. The reaction was heated at 200 ºC for 15 h using a heating block. The reaction mixture was allowed to warm to room temperature and was then diluted in ethyl acetate or dichloromethane and washed with a 0.1 M solution of sodium thiosulfate (10 mL) and a saturated sodium bicarbonate solution (10 mL). The organic layer was dried over magnesium sulfate and the solvent was removed in rotary evaporator under reduced pressure.

The obtained residue was dissolved in 0.5 mL of acetic acid and 3-Aminopiperidine-2,6-dione hydrochloride (12.8 mg, 0.1 mmol, 1.0 equiv.), sodium acetate (9.4 mg, 0.11 mmol, 1.1 equiv.), and a magnetic stir bar were added. The reaction was heated at 120 ºC for 15 h in a heating block. The reaction mixture was allowed to warm to room temperature, a brine solution was added and the aqueous phase was extracted with ethyl acetate (3 x 10 mL). The organic layers

were combined, dried over magnesium sulfate and the solvent was removed in rotary evaporator under reduced pressure. The resulting crude residue was purified by flash column chromatography using hexane/ethyl acetate 30–70% mixture and silica gel (10 g). The fractions were combined, evaporated in rotary evaporator under reduced pressure and dried in a high vacuum system. The product (white solid) was obtained in 32% yield (10.2 mg, 0.032 mmol).

Procedure H: Synthesis of Compound 7 (Pomalyst®)

To a 5 mL glass sealed tube under air atmosphere, compound 6 (13.5 mg, 0.043 mmol, 1.0 equiv.), concentrated HCl (35 µL), methanol (0.25 mL) and a magnetic stir bar were added. The reaction was heated up at reflux for 4 h in a heating block. Next, the reaction mixture was allowed to warm to room temperature and the solid was filtered, washed with methanol and dried. The product (yellow solid) was obtained in 45% yield (5.3 mg, 0.019 mmol).

Structural Characterization

Pyrone 1a

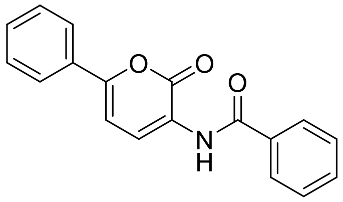

Prepared according to Procedure A and purified by Method A, starting with acetophenone. Yield: 70% (205 mg, 0.701 mmol); Yellow solid; ^{1}H NMR (600 MHz, $CDCl_3$) δ 8.74 (s, 1H), 8.56 (d, J = 7.6 Hz, 1H), 7.93 – 7.91 (m, 2H), 7.82 – 7.80 (m, 2H), 7.61 – 7.58 (m, 1H), 7.54 – 7.50 (m, 2H), 7.48 – 7.42 (m, 3H), 6.81 (d, J = 7.6 Hz, 1H); ^{13}C NMR (150 MHz, $CDCl_3$) δ 166.2, 159.9, 154.0, 133.8, 132.6, 131.3, 130.3, 129.1, 129.1, 127.3, 125.1, 124.5, 124.3, 102.3. The spectral data is consistent with literature data.[5]

Pyrone 1b

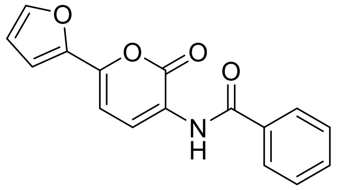

Prepared according to Procedure A and purified by Method A, starting with 2-furyl methyl ketone. Yield: 63% (177.0 mg, 0.630 mmol); Orange solid; ^{1}H NMR (500 MHz, $CDCl_3$) δ 8.70 (s, 1H), 8.53 (d, J = 7.6 Hz, 1H), 7.93 – 7.86 (m, 2H), 7.61 – 7.54 (m, 1H), 7.54 – 7.46 (m, 3H), 6.91 (d, J = 3.5 Hz, 1H), 6.69 (d, J = 7.6 Hz, 1H), 6.53 (dd, J = 3.5, 1.8 Hz, 1H); ^{13}C NMR (125 MHz, $CDCl_3$) δ 166.1, 159.0, 146.5, 146.4, 144.5, 133.8, 132.6, 129.1, 127.3, 124.6, 123.9, 112.5, 110.4, 100.8. The spectral data is consistent with literature data.[5]

Pyrone 1c

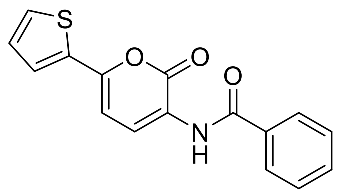

Prepared according to Procedure A and purified by Method A, starting with 2-thienyl methyl ketone. Yield: 81% (241.8 mg, 0.812 mmol); Yellow solid; ^{1}H NMR (500 MHz, $CDCl_3$) δ 8.70 (s, 1H), 8.50 (d, J = 7.7 Hz, 1H), 7.93 – 7.87 (m, 2H), 7.61 – 7.56 (m, 1H), 7.55 – 7.48 (m, 3H), 7.41 (dd, J = 5.0, 1.3 Hz, 1H), 7.10 (dd, J = 5.0, 3.8 Hz, 1H), 6.62 (d, J = 7.7 Hz, 1H); ^{13}C NMR (125 MHz, $CDCl_3$) δ 166.1, 159.2, 150.1, 135.0, 133.8, 132.6, 129.1, 128.5, 128.1, 127.3, 126.3, 124.6, 123.8, 101.6. The spectral data is consistent with literature data.[5]

Pyrone 1d

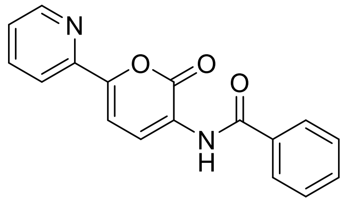

Prepared according to Procedure A and purified by Method B, starting with 2-Acetylpyridine. Yield: 17% (50.5 mg, 0.173 mmol); Light red solid; ^{1}H NMR (500 MHz, $CDCl_3$) δ 8.79 (s, 1H), 8.64 (ddd, J = 4.8, 1.8, 1.0 Hz, 1H), 8.61 (d, J = 7.7 Hz, 1H), 7.95 – 7.90 (m, 3H), 7.81 (td, J = 7.8, 1.8 Hz, 1H), 7.61 – 7.57 (m, 1H), 7.54 – 7.50 (m, 2H), 7.48 (d, J = 7.7 Hz, 1H), 7.31 (ddd, J = 7.6, 4.8, 1.0 Hz, 1H); ^{13}C NMR (125 MHz, $CDCl_3$) δ 166.2, 159.5, 152.4, 150.0, 148.9, 137.3, 133.7, 132.7, 129.1, 127.3, 125.8, 124.4, 124.2, 119.7, 104.6. The spectral data is consistent with literature data.[5]

Pyrone 1e

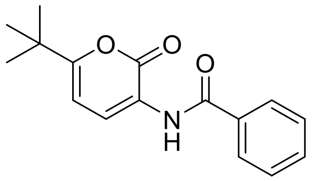

Prepared according to Procedure A and purified by Method A, starting with tert-butyl methyl ketone. Yield: 62% (168.8 mg, 0.622 mmol); Light brown solid; ^{1}H NMR (600 MHz, $CDCl_3$) δ 8.63 (s, 1H), 8.36 (d, J = 7.5 Hz, 1H), 7.89 – 7.87 (m, 2H), 7.58 – 7.55 (m, 1H), 7.51 – 7.48 (m, 2H), 6.14 (d, J = 7.5 Hz, 1H), 1.29 (s, 9H); ^{13}C NMR (150 MHz, $CDCl_3$) δ 166.2, 165.9, 160.5, 133.9, 132.5, 129.0, 127.2, 124.6, 123.2, 100.2, 35.9, 28.1. The spectral data is consistent with literature data.[5]

Pyrone 1f

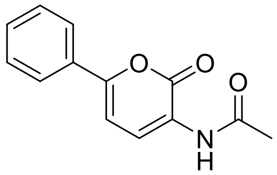

Prepared according to Procedure A and purified by Method B, starting with acetophenone. Yield: 11% (25.8 mg, 0.113 mmol); Light orange solid; ^{1}H NMR (600 MHz, $CDCl_3$) δ 8.37 (d, J = 7.7 Hz, 1H), 7.98 (s, 1H), 7.79 – 7.76 (m, 2H), 7.46 – 7.41 (m, 3H), 6.74 (d, J = 7.7 Hz, 1H), 2.23 (s, 3H); ^{13}C NMR (150 MHz, $CDCl_3$) δ 169.4, 159.6, 153.9, 131.2, 130.3, 129.1, 125.1, 124.4, 124.1, 102.2, 24.8. The spectral data is consistent with literature data.[5]

Pyrone 1g

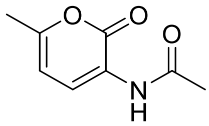

Prepared according to Procedure A and purified by Method B, starting with acetone. Yield: 10% (17.5 mg, 0.104 mmol); Light yellow solid; ^{1}H NMR (500 MHz, $CDCl_3$) δ 8.16 (d, J = 7.4 Hz, 1H), 7.91 (s, 1H), 6.01 (d, J = 7.4, 1H), 2.23 (s, 3H), 2.17 (s, 3H); ^{13}C NMR (125 MHz, $CDCl_3$) δ 169.4, 160.4, 155.1, 124.6, 123.1, 104.1, 24.7, 19.4. The spectral data is consistent with literature data.[5]

Pyrone 1h

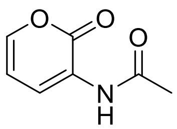

Prepared according to Procedure B. Yield: 10% (16.0 mg, 0.104 mmol); Light orange solid; mp: 166–167 ºC; ^{1}H NMR (500 MHz, $CDCl_3$) δ 8.23 (dd, J = 7.2, 1.9 Hz, 1H), 8.01 (s, 1H), 7.24 (dd, J = 5.2, 1.9 Hz, 1H), 6.29 (dd, J = 7.2, 5.2 Hz, 1H), 2.20 (s, 3H); ^{13}C NMR (125 MHz, $CDCl_3$) δ 169.6, 159.7, 144.4, 126.0, 122.9, 107.3, 24.8; FTIR (ATR) $\tilde{\nu}$ 3332, 1713, 1671, 1529, 1342, 1265, 1129, 1075, 772, 593, 539, 501; HRMS (ESI-TOF) *m/z* $[M+H]^+$ Calculated for $C_7H_8NO_3^+$ 154.0499, found 154.0497 (|Δ*m/z*|= 1.3 ppm).

Aromatic 2a

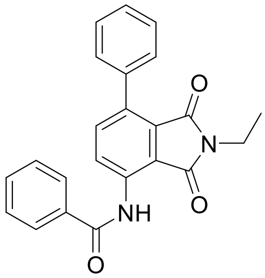

Prepared according to Procedure C and purified by Method A, starting with pyrone 1a (29.1 mg, 0.1 mmol, 1.0 equiv.) and *N*-ethylmaleimide (25 mg, 0.2 mmol, 2.0 equiv.). Yield: 84% (31.1 mg, 0.084 mmol); Light yellow solid; mp: 208–209 ºC; ^{1}H NMR (500 MHz, $CDCl_3$) δ 10.84 (s, 1H), 9.00 (d, J = 8.7 Hz, 1H), 8.09 – 8.06 (m, 2H), 7.68 (d, J = 8.7 Hz, 1H), 7.64 – 7.60 (m, 1H), 7.58 – 7.54 (m, 4H), 7.50 – 7.44 (m, 3H), 3.73 (q, J = 7.2 Hz, 2H), 1.28 (t, J = 7.2 Hz, 3H); ^{13}C{^{1}H} NMR (125 MHz, $CDCl_3$) δ 170.5, 167.3, 165.8, 138.1, 137.1, 136.0, 135.9, 133.7, 132.7, 129.5, 129.2, 128.7, 128.3, 127.5, 126.8, 125.1, 117.0, 33.1, 14.0. FTIR (ATR) $\tilde{\nu}$ 3345, 3058, 1758, 1682, 1623, 1602, 1526, 1446, 1404, 1345, 1254, 1171, 1035, 895, 847, 766, 750, 693, 685, 590, 571; HRMS (ESI-TOF) *m/z* $[M+H]^+$ Calculated for $C_{23}H_{19}N_2O_3^+$ 371.1390, found 371.1386 (|Δ*m/z*|= 1.0 ppm).

Aromatic 2b

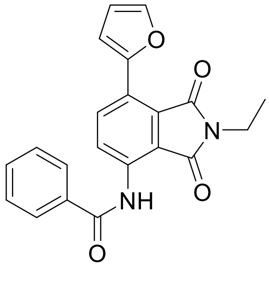

Prepared according to Procedure C and purified by Method A, starting with pyrone 1b (28.1 mg, 0.1 mmol, 1.0 equiv.) and *N*-ethylmaleimide (25 mg, 0.2 mmol, 2.0 equiv.). Yield: 72% (26.0 mg, 0.072 mmol); Yellow solid; mp: 203–204 ºC; ^{1}H NMR (500 MHz, $CDCl_3$) δ 10.92 (s, 1H), 9.00 (d, *J* = 9.0 Hz, 1H), 8.22 (d, *J* = 9.0 Hz, 1H), 8.07 – 8.04 (m, 2H), 7.88 (d, *J* = 3.5 Hz, 1H), 7.62 – 7.59 (m, 1H), 7.57 – 7.53 (m, 3H), 6.58 (dd, *J* = 3.5, 1.8 Hz, 1H), 3.78 (q, *J* = 7.2 Hz, 2H), 1.32 (t, *J* = 7.2 Hz, 3H); $^{13}C\{^{1}H\}$ NMR (125 MHz, $CDCl_3$) δ 170.3, 167.3, 165.8, 148.9, 143.3, 136.7, 133.7, 133.7, 132.7, 129.2, 127.5, 125.3, 124.4, 123.7, 116.9, 113.9, 112.5, 33.2, 14.0. FTIR (ATR) $\tilde{\nu}$ 3343, 3114, 2117, 1743, 1680, 1622, 1528, 1441, 1413, 1346, 1256, 1180, 1014, 885, 843, 762, 696, 567; HRMS (ESI-TOF) *m/z* $[M+H]^+$ Calculated for $C_{21}H_{17}N_2O_4^+$ 361.1183, found 361.1177 (|Δ*m/z*|= 1.6 ppm).

Aromatic 2c

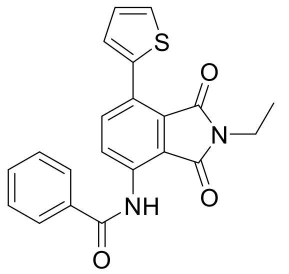

Prepared according to Procedure C and purified by Method A, starting with pyrone **1c** (29.7 mg, 0.1 mmol, 1.0 equiv.) and *N*-ethylmaleimide (25 mg, 0.2 mmol, 2.0 equiv.). Yield: 86% (32.4 mg, 0.086 mmol); Yellow solid; mp: 196–197 ºC; ^{1}H NMR (500 MHz, $CDCl_3$) δ 10.90 (s, 1H), 8.97 (d, *J* = 8.8 Hz, 1H), 8.08 – 8.02 (m, 2H), 7.87 – 7.83 (m, 2H), 7.63 – 7.59 (m, 1H), 7.56 – 7.54 (m, 2H), 7.45 (dd, *J* = 5.1, 1.2 Hz, 1H), 7.16 (dd, *J* = 5.1, 3.6 Hz, 1H), 3.76 (q, *J* = 7.2 Hz, 2H), 1.31 (t, *J* = 7.2 Hz, 3H); $^{13}C\{^{1}H\}$ NMR (125 MHz, $CDCl_3$) δ 170.2, 167.3, 165.8, 137.6, 137.6, 137.0, 133.6, 132.8, 129.6, 129.2, 128.6, 127.9, 127.5, 127.4, 125.7, 125.3, 117.2, 33.2, 14.0. FTIR (ATR) $\tilde{\nu}$ 3332, 3089, 1744, 1677, 1622, 1527, 1491, 1439, 1409, 1327, 1266, 1041, 1180, 1082, 892, 848, 821, 761, 690, 561; HRMS (ESI-TOF) *m/z* $[M+H]^+$ Calculated for $C_{21}H_{17}N_2O_3S^+$ 377.0954, found 377.0950 (|Δ*m/z*|= 1.0 ppm).

Aromatic 2d

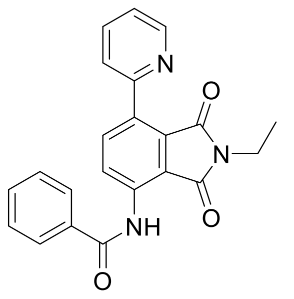

Prepared according to Procedure C and purified by Method A by flash column chromatography using hexane/ethyl acetate 20–60% mixture and silica gel (10 g), starting with pyrone 1d (29.2 mg, 0.1 mmol, 1.0 equiv.) and *N*-ethylmaleimide (25 mg, 0.2 mmol, 2.0 equiv.). Yield: 91% (33.9 mg, 0.091 mmol); Beige solid; mp: 175–176 ºC; ^{1}H NMR (500 MHz, $CDCl_3$) δ 10.86 (s, 1H), 9.04 (d, *J* = 8.8 Hz, 1H), 8.74 – 8.72 (m, 1H), 8.09 – 8.03 (m, 3H), 7.88 (dt, *J* = 7.9, 1.2 Hz, 1H), 7.79 (td, *J* = 7.9, 1.9 Hz, 1H), 7.62 – 7.57 (m, 1H), 7.56 – 7.52 (m, 2H), 7.33 (ddd, *J* = 7.6, 4.9, 1.2 Hz, 1H), 3.73 (q, *J* = 7.2 Hz, 2H), 1.28 (t, *J* = 7.2 Hz, 3H); ^{13}C{^{1}H} NMR (125 MHz, $CDCl_3$) δ 170.3, 167.2, 165.7, 153.4, 149.7, 138.4, 138.0, 136.0, 134.1, 133.5, 132.7, 129.1, 127.5, 127.3, 125.4, 125.0, 123.3, 116.6, 33.1, 13.9; FTIR (ATR) $\tilde{\nu}$ 3322, 3065, 1754, 1684, 1623, 1492, 1409, 1346, 1256, 1168, 1042, 891, 865, 760, 694, 688, 576, 510; HRMS (ESI-TOF) *m/z* $[M+H]^+$ Calculated for $C_{22}H_{18}N_3O_3^+$ 372.1343, found 372.1339 (|Δ*m/z*|= 1.1 ppm).

Aromatic 2e

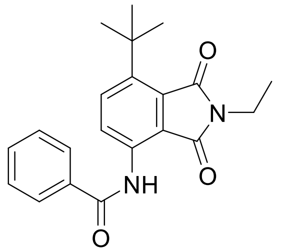

Prepared according to Procedure C and purified by Method A, starting with pyrone 1e (27.1 mg, 0.1 mmol, 1.0 equiv.) and *N*-ethylmaleimide (25 mg, 0.2 mmol, 2.0 equiv.). Obtained as major product. Yield: 53% (18.6 mg, 0.053 mmol); White cream solid; mp: 162–163 ºC; ^{1}H NMR (500 MHz, $CDCl_3$) δ 10.96 (s, 1H), 8.86 (d, *J* = 9.0 Hz, 1H), 8.06 – 8.03 (m, 2H), 7.71 (d, *J* = 9.0 Hz, 1H), 7.61 – 7.57 (m, 1H), 7.56 – 7.52 (m, 2H), 3.75 (q, *J* = 7.2 Hz, 2H), 1.51 (s, 9H), 1.30 (t, *J* = 7.2 Hz, 3H); ^{13}C{^{1}H} NMR (125 MHz, $CDCl_3$) δ 170.8, 167.8, 165.8, 146.7, 136.2, 134.1, 133.9, 132.6, 129.2, 128.1, 127.5, 125.0, 118.1, 35.3, 33.1, 29.9, 14.0; FTIR (ATR) $\tilde{\nu}$ 3343, 2940, 1753, 1681, 1622, 1523, 1494, 1410, 1337, 1322, 1264, 1182, 1045, 896,

853, 768, 701, 600, 574, 511; HRMS (ESI-TOF) *m/z* $[M+H]^+$ Calculated for $C_{21}H_{23}N_2O_3^+$ 351.1703, found 351.1699 (|Δ*m/z*|= 1.1 ppm).

Aromatic 2e’

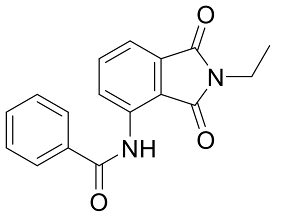

Obtained as minor product alongside with Aromatic 2e. Yield: 32% (9.4 mg, 0.032 mmol); White solid; mp: 126–127 ºC; ^{1}H NMR (500 MHz, $CDCl_3$) δ 10.55 (s, 1H), 8.96 (d, *J* = 8.5 Hz, 1H), 8.06 – 8.03 (m, 2H)-, 7.75 – 7.71 (m, 1H), 7.64 – 7.59 (m, 1H), 7.57 – 7.53 (m, 3H), 3.76 (q, *J* = 7.2 Hz, 2H), 1.31 (t, *J* = 7.2 Hz, 3H); $^{13}C\{^1H\}$ NMR (125 MHz, $CDCl_3$) δ 170.7, 167.9, 165.8, 137.8, 136.1, 133.6, 132.8, 131.9, 129.2, 127.5, 124.9, 118.2, 116.6, 33.1, 14.1; FTIR (ATR) $\tilde{\nu}$ 3342, 2962, 1766, 1691, 1677, 1622, 1541, 1493, 1479, 1443, 1400, 1347, 1331, 1258, 1177, 1034, 819, 743, 704, 691, 649, 641, 542, 509; HRMS (ESI-TOF) *m/z* $[M+H]^+$ Calculated for $C_{17}H_{15}N_2O_3^+$ 295.1077, found 295.1074 (|Δ*m/z*|= 1.0 ppm).

Aromatic 2f

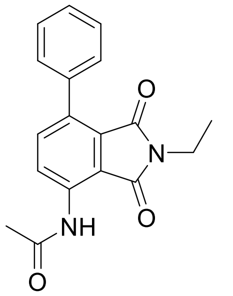

Prepared according to Procedure C and purified by Method A, starting with pyrone 1f (16.7 mg, 0.1 mmol, 1.0 equiv.) and *N*-ethylmaleimide (25 mg, 0.2 mmol, 2.0 equiv.). Yield: 62% (19.2 mg, 0.062 mmol); Yellow solid; mp: 144–145 ºC; ^{1}H NMR (500 MHz, $CDCl_3$) δ 9.83 (s, 1H), 8.80 (d, *J* = 8.7 Hz, 1H), 7.60 (d, *J* = 8.7 Hz, 1H), 7.54 – 7.52 (m, 2H), 7.48 – 7.42 (m, 3H), 3.68 (q, *J* = 7.2 Hz, 2H), 2.29 (s, 3H), 1.26 (t, *J* = 7.2 Hz, 3H); $^{13}C\{^1H\}$ NMR (125 MHz, $CDCl_3$) δ 170.1, 169.5, 167.2, 137.9, 136.8, 136.0, 135.9, 129.5, 128.7, 128.3, 126.8, 125.0, 116.4, 33.0, 25.2, 13.9; FTIR (ATR) $\tilde{\nu}$ 3337, 2983, 1756, 1689, 1624, 1600, 1500, 1477, 1442, 1404, 1338, 1227, 1164, 1038, 986, 891, 854, 760, 701, 669, 579, 500; HRMS (ESI-TOF) *m/z* $[M+H]^+$ Calculated for $C_{18}H_{17}N_2O_3^+$ 309.1234, found 309.1228 (|Δ*m/z*|= 1.9 ppm).

Aromatic 2g

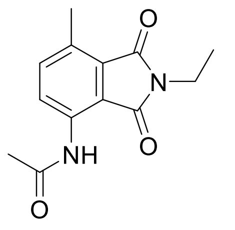

Prepared according to Procedure C and purified by Method A, starting with pyrone 1g (16.7 mg, 0.1 mmol, 1.0 equiv.) and *N*-ethylmaleimide (25 mg, 0.2 mmol, 2.0 equiv.). Yield: 69% (17.0 mg, 0.069 mmol); Yellow solid; mp: 110–111 ºC; ^{1}H NMR (500 MHz, $CDCl_3$) δ 9.56 (s, 1H), 8.61 (d, *J* = 8.6 Hz, 1H), 7.39 (d, *J* = 8.6 Hz, 1H), 3.68 (q, *J* = 7.2 Hz, 2H), 2.60 (s, 3H), 2.24 (s, 3H), 1.26 (t, *J* = 7.2 Hz, 3H); $^{13}C\{^{1}H\}$ NMR (125 MHz, $CDCl_3$) δ 170.2, 169.3, 168.4, 138.3, 135.6, 132.6, 127.9, 124.7, 115.9, 32.8, 25.1, 17.1, 14.1; FTIR (ATR) $\tilde{\nu}$ 3356, 2985, 1758, 1681, 1629, 1609, 1530, 1493, 1443, 1340, 1298, 1213, 1163, 1029, 849, 762, 619, 554, 541, 520; HRMS (ESI-TOF) *m/z* $[M+H]^+$ Calculated for $C_{13}H_{15}N_2O_3^+$ 247.1077, found 247.1074 (|Δ*m/z*|= 1.2 ppm).

Aromatic 2h

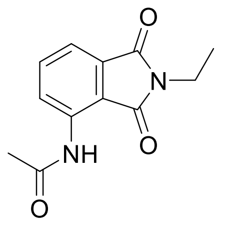

Prepared according to Procedure C and purified by Method A, starting with pyrone 1h (15.3 mg, 0.1 mmol, 1.0 equiv.) and *N*-ethylmaleimide (25 mg, 0.2 mmol, 2.0 equiv.). Yield: 52% (12.1 mg, 0.052 mmol); White solid; mp: 96–97 ºC; ^{1}H NMR (500 MHz, $CDCl_3$) δ 9.54 (s, 1H), 8.76 (d, *J* = 8.5 Hz, 1H), 7.66 (dd, *J* = 8.5, 7.2 Hz, 1H), 7.50 (d, *J* = 7.2 Hz, 1H), 3.72 (q, *J* = 7.2 Hz, 2H), 2.28 (s, 3H), 1.28 (t, *J* = 7.2 Hz, 3H); $^{13}C\{^{1}H\}$ NMR (125 MHz, $CDCl_3$) δ 170.3, 169.4, 167.8, 137.5, 135.9, 131.8, 124.8, 118.1, 115.9, 33.0, 25.1, 14.1; FTIR (ATR) $\tilde{\nu}$ 3348, 2939, 1761, 1688, 1617, 1522, 1475, 1443, 1400, 1343, 1287, 1234, 1161, 1031, 985, 883, 822, 744, 669, 595, 530, 503; HRMS (ESI-TOF) *m/z* $[M+H]^+$ Calculated for $C_{12}H_{13}N_2O_3^+$ 233.0920, found 233.0917 (|Δ*m/z*|= 1.3 ppm).

Aromatic 2i

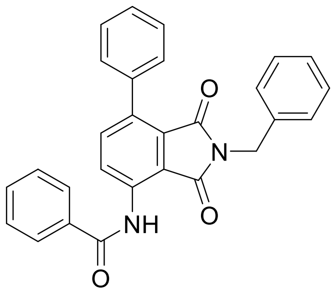

Prepared according to Procedure C and purified by Method A, starting with pyrone 1a (29.1 mg, 0.1 mmol, 1.0 equiv.) and *N*-benzylmaleimide (37.4 mg, 0.2 mmol, 2.0 equiv.). Yield: 49% (21.3 mg, 0.049 mmol); White solid; mp: 222–223 ºC; ^{1}H NMR (500 MHz, $CDCl_3$) δ 10.80 (s, 1H), 9.01 (d, *J* = 8.7 Hz, 1H), 8.08 – 8.04 (m, 2H), 7.68 (d, *J* = 8.7 Hz, 1H), 7.64 – 7.60 (m, 1H), 7.59 – 7.55 (m, 4H), 7.49 – 7.42 (m, 5H), 7.34 – 7.30 (m, 2H), 7.29 – 7.26 (m, 1H), 4.83 (s, 2H); ^{13}C{^{1}H} NMR (125 MHz, $CDCl_3$) δ 170.2, 167.1, 165.9, 138.3, 137.2, 136.2, 136.2, 135.9, 133.7, 132.8, 129.5, 129.2, 128.9, 128.8, 128.7, 128.3, 128.1, 127.6, 126.7, 116.9, 41.7; FTIR (ATR) $\tilde{\nu}$ 3354, 2920, 1758, 1682, 1622, 1605, 1527, 1489, 1431, 1403, 1329, 1292, 1255, 1172, 1066, 938, 848, 751, 687, 625, 571, 540, 507; HRMS (ESI-TOF) *m/z* [M+H]$^+$ Calculated for $C_{28}H_{21}N_2O_3^+$ 433.1547, found 433.1540 (|Δ*m/z*|= 1.6 ppm).

Aromatic 2j

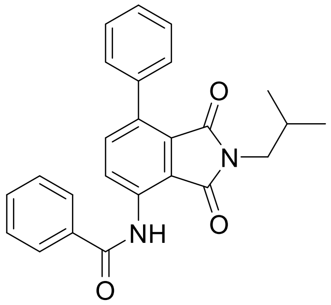

Prepared according to Procedure C and purified by Method A, starting with pyrone 1a (29.1 mg, 0.1 mmol, 1.0 equiv.) and *N*-isobutylmaleimide (30.6 mg, 0.2 mmol, 2.0 equiv.). Yield: 63% (25.3 mg, 0.063 mmol); Light yellow solid; mp: 187–188 ºC; ^{1}H NMR (500 MHz, $CDCl_3$) δ 10.85 (s, 1H), 9.01 (d, *J* = 8.7 Hz, 1H), 8.09 – 8.05 (m, 2H), 7.69 (d, *J* = 8.7 Hz, 1H), 7.64 – 7.59 (m, 1H), 7.59 – 7.54 (m, 4H), 7.50 – 7.41 (m, 3H), 3.48 (d, *J* = 7.4 Hz, 2H), 2.13 (hept, *J* = 6.7 Hz, 1H), 0.95 (d, *J* = 6.7 Hz, 6H); ^{13}C{^{1}H} NMR (125 MHz, $CDCl_3$) δ 170.8, 167.7, 165.8, 138.2, 137.1, 136.0, 135.9, 133.7, 132.7, 129.5, 129.2, 128.7, 128.3, 127.5, 126.6, 125.1, 116.9, 45.5, 27.9, 20.3; FTIR (ATR) $\tilde{\nu}$ 3348, 3061, 1753, 1680, 1606, 1526, 1480, 1435, 1403,

1346, 1297, 1281, 1256, 1173, 1054, 913, 901, 844, 754, 687, 624, 573, 514; HRMS (ESI-TOF) *m/z* $[M+H]^+$ Calculated for $C_{25}H_{23}N_2O_3^+$ 399.1703, found 399.1696 (|Δ*m/z*|= 1.7 ppm).

Aromatic 2k

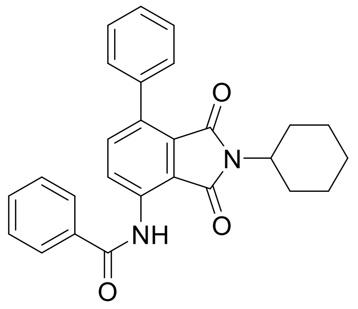

Prepared according to Procedure C and purified by Method A, starting with pyrone 1a (29.1 mg, 0.1 mmol, 1.0 equiv.) and *N*-cyclohexylmaleimide (26.9 mg, 0.15 mmol, 1.5 equiv.). Yield: 61% (25.8 mg, 0.061 mmol); White cream solid; mp: 187–188 ºC; ^{1}H NMR (500 MHz, $CDCl_3$) δ 10.90 (s, 1H), 9.00 (d, *J* = 8.7 Hz, 1H), 8.10 – 8.04 (m, 2H), 7.66 (d, *J* = 8.7 Hz, 1H), 7.64 – 7.60 (m, 1H), 7.58 – 7.54 (m, 4H), 7.50 – 7.42 (m, 3H), 4.09 (tt, *J* = 12.3, 3.9 Hz, 1H), 2.20 (qd, *J* = 12.3, 3.5 Hz, 2H), 1.89 – 1.83 (m, 2H), 1.79 – 1.66 (m, 3H), 1.39 – 1.22 (m, 3H); $^{13}C\{^1H\}$ NMR (125 MHz, $CDCl_3$) δ 170.7, 167.5, 165.8, 138.0, 137.0, 136.1, 135.8, 133.7, 132.7, 129.5, 129.1, 128.6, 128.2, 127.5, 126.6, 124.9, 116.8, 51.2, 30.0, 26.2, 25.2; FTIR (ATR) $\tilde{\nu}$ 3343, 2912, 1752, 1674, 1623, 1600, 1523, 1482, 1447, 1396, 1376, 1326, 1290, 1257, 1164, 1098, 1034, 895, 857, 757, 695, 636, 568, 513; HRMS (ESI-TOF) *m/z* $[M+H]^+$ Calculated for $C_{27}H_{25}N_2O_3^+$ 425.1860, found 425.1852 (|Δ*m/z*|= 1.9 ppm).

Aromatic 2l

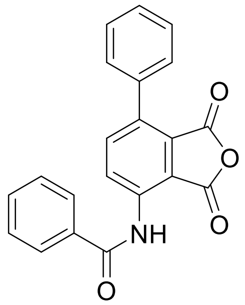

Prepared according to Procedure C and purified by Method A by flash column chromatography using toluene/ethyl acetate 0–5% mixture and silica gel (25 g), starting with pyrone 1a (29.1 mg, 0.1 mmol, 1.0 equiv.), maleic anhydride (19.6 mg, 0.2 mmol, 2.0 equiv.) and iodine (5.2 mg, 0.02 mmol, 0.2 equiv.). Yield: 76% (26.1 mg, 0.076 mmol); Pale yellow solid; mp: 207–

208 ºC; ^{1}H NMR (600 MHz, $CDCl_3$) δ 10.31 (s, 1H), 9.17 (d, *J* = 8.7 Hz, 1H), 8.06 – 8.02 (m, 2H), 7.88 (d, *J* = 8.7 Hz, 1H), 7.67 – 7.63 (m, 1H), 7.60 – 7.56 (m, 4H), 7.53 – 7.48 (m, 3H); $^{13}C\{^1H\}$ NMR (150 MHz, $CDCl_3$) δ 165.8, 165.4, 161.2, 140.2, 138.4, 138.0, 134.7, 133.2, 133.1, 129.4, 129.4, 129.3, 128.7, 127.6, 126.6, 125.9, 116.6; FTIR (ATR) $\tilde{\nu}$ 3354, 3056, 1837, 1763, 1681, 1600, 1524, 1479, 1446, 1364, 1298, 1220, 1207, 1024, 905, 860, 747, 693, 594, 572; HRMS (ESI-TOF) *m/z* $[M+H]^+$ Calculated for $C_{21}H_{14}NO_4^+$ 344.0917, found 344.0910 (|Δ*m/z*|= 2.0 ppm).

Aromatic 2m

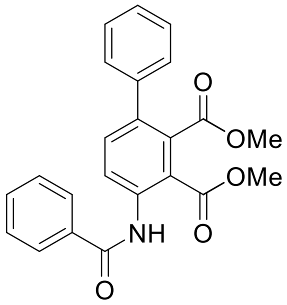

Prepared according to Procedure C and purified by Method A, starting with pyrone 1a (29.1 mg, 0.1 mmol, 1.0 equiv.) and dimethyl acetylenedicarboxylate (57.4 mg, 0.4 mmol, 4.0 equiv.). Yield: 50% (19.5 mg, 0.050 mmol); Light yellow solid; mp: 151–152 ºC; ^{1}H NMR (500 MHz, $CDCl_3$) δ 11.28 (s, 1H), 8.91 (d, *J* = 8.6 Hz, 1H), 8.03 – 8.00 (m, 2H), 7.61 – 7.57 (m, 2H), 7.54 (m, 2H), 7.41 – 7.36 (m, 3H), 7.34 – 7.31 (m, 2H), 3.91 (s, 3H), 3.59 (s, 3H); $^{13}C\{^1H\}$ NMR (125 MHz, $CDCl_3$) δ 169.0, 168.2, 165.7, 139.5, 139.4, 135.6, 135.4, 134.9, 134.6, 132.4, 129.0, 128.6, 128.4, 127.9, 127.5, 122.4, 115.3, 53.2, 52.3; FTIR (ATR) $\tilde{\nu}$ 3330, 2949, 1715, 1696, 1679, 1594, 1527, 1491, 1438, 1319, 1294, 1230, 1158, 1108, 1005, 909, 853, 771, 686, 545; HRMS (ESI-TOF) *m/z* $[M+H]^+$ Calculated for $C_{23}H_{20}NO_5^+$ 390.1336, found 390.1330 (|Δ*m/z*|= 1.5 ppm).

Aromatic 2n

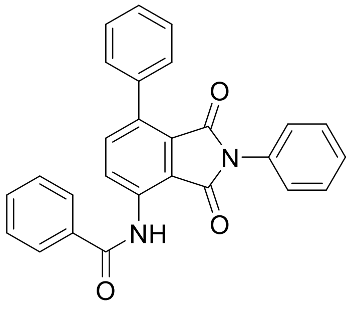

Prepared according to Procedure C and purified by Method B, starting with pyrone 1a (29.1 mg, 0.1 mmol, 1.0 equiv.) and *N*-phenylmaleimide (34.6 mg, 0.2 mmol, 2.0 equiv.). Yield: 72% (30.0 mg, 0.072 mmol); Yellow solid; mp: 273–274 ºC; ^{1}H NMR (600 MHz, $CDCl_3$) δ 10.94 (s, 1H), 9.11 (d, *J* = 8.7 Hz, 1H), 8.09 – 8.06 (m, 2H), 7.77 (d, *J* = 8.7, 1H), 7.62 – 7.59 (m, 3H), 7.55 – 7.49 (m, 4H), 7.49 – 7.40 (m, 6H); ^{13}C{^{1}H} NMR (150 MHz, $CDCl_3$) δ 169.7, 166.4, 165.9, 138.8, 137.7, 136.6, 135.8, 133.5, 132.8, 131.2, 129.6, 129.4, 129.2, 128.8, 128.6, 128.3, 127.6, 126.8, 126.3, 125.5, 116.5; FTIR (ATR) $\tilde{\nu}$ 3343, 3058, 1755, 1699, 1688, 1600, 1525, 1501, 1490, 1482, 1387, 1352, 1284, 1255, 1193, 1115, 888, 847, 752, 685, 625, 505; HRMS (ESI-TOF) *m/z* $[M+H]^+$ Calculated for $C_{27}H_{19}N_2O_3^+$ 419.1390, found 419.1390 (|Δ*m/z*|= 0 ppm).

Aromatic 2o

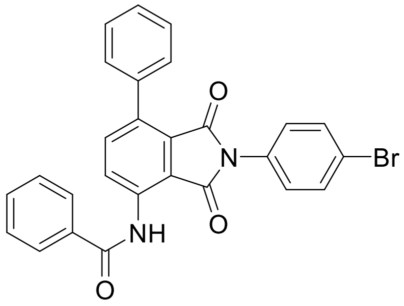

Prepared according to Procedure C and purified by Method B, starting with pyrone 1a (29.1 mg, 0.1 mmol, 1.0 equiv.) and *N*-(4-bromophenyl)maleimide (50.4 mg, 0.2 mmol, 2.0 equiv.). Yield: 95% (47.1 mg, 0.095 mmol); Pale yellow solid; mp: 339–341 ºC; ^{1}H NMR (500 MHz, $CDCl_3$) δ 10.89 (s, 1H), 9.12 (d, *J* = 8.7 Hz, 1H), 8.09 – 8.04 (m, 2H), 7.78 (d, *J* = 8.7 Hz, 1H), 7.64 – 7.62 (m, 2H), 7.61 – 7.52 (m, 5H), 7.50 – 7.44 (m, 3H), 7.36 – 7.32 (m, 2H); ^{13}C{^{1}H} NMR (125 MHz, $CDCl_3$) δ 169.3, 166.0, 165.9, 138.9, 137.8, 136.7, 135.8, 133.5, 132.9, 132.5, 130.3, 129.5, 129.2, 128.9, 128.4, 128.2, 127.6, 126.1, 125.7, 122.3, 116.3; FTIR (ATR) $\tilde{\nu}$ 3347, 3048, 1759, 1688, 1620, 1604, 1525, 1490, 1445, 1387, 1352, 1254, 1195, 1109, 1078, 1018, 832, 810, 693, 688, 650, 631, 568, 501; HRMS (ESI-TOF) *m/z* $[M+H]^+$ Calculated for $C_{27}H_{18}BrN_2O_3^+$ 497.0495, found 497.0496 (|Δ*m/z*|= 0.2 ppm).

Aromatic 2p

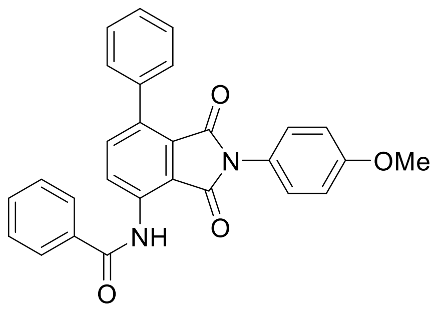

Prepared according to Procedure C and purified by Method B, starting with pyrone 1a (29.1 mg, 0.1 mmol, 1.0 equiv.) and *N*-(4-methoxyphenyl)maleimide (40.6 mg, 0.2 mmol, 2.0 equiv.). Yield: 91% (41.2 mg, 0.091 mmol); Pale yellow solid; mp: 278–279 ºC; ^{1}H NMR (500 MHz, $CDCl_3$) δ 10.94 (s, 1H), 9.10 (d, *J* = 8.6 Hz, 1H), 8.10 – 8.04 (m, 2H), 7.76 (d, *J* = 8.6 Hz, 1H), 7.63 – 7.57 (m, 3H), 7.55 – 7.51 (m, 2H), 7.49 – 7.42 (m, 3H), 7.35 – 7.30 (m, 2H), 7.03 – 6.99 (m, 2H), 3.84 (s, 3H); ^{13}C{^{1}H} NMR (125 MHz, $CDCl_3$) δ 169.9, 166.7, 165.9, 159.6, 138.7, 137.6, 136.5, 135.9, 133.5, 132.8, 129.6, 129.2, 128.8, 128.3, 128.2, 127.6, 126.4, 125.5, 123.8, 116.6, 114.7, 55.7; FTIR (ATR) $\tilde{\nu}$ 3337, 3060, 1752, 1698, 1688, 1624, 1604, 1514, 1491, 1398, 1357, 1289, 1254, 1196, 1172, 1118, 1034, 963, 891, 844, 818, 749, 687, 571, 511; HRMS (ESI-TOF) *m/z* $[M+H]^+$ Calculated for $C_{28}H_{21}N_2O_4^+$ 449.1496, found 449.1494 (|Δ*m/z*|= 0.4 ppm).

Aromatic 2q

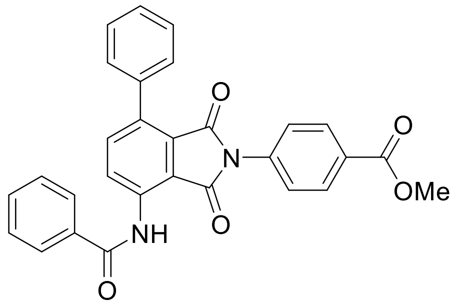

Prepared according to Procedure C and purified by Method B, starting with pyrone 1a (29.1 mg, 0.1 mmol, 1.0 equiv.) and *N*-[4-(methoxycarbonyl)phenyl]maleimide (46.2 mg, 0.2 mmol, 2.0 equiv.). Yield: 92% (44.1 mg, 0.092 mmol); Yellow solid; mp: 340–341 ºC; ^{1}H NMR (600 MHz, $CDCl_3$) δ 10.89 (s, 1H), 9.13 (d, *J* = 8.7 Hz, 1H), 8.19 – 8.15 (m, 2H), 8.09 – 8.06 (m, 2H), 7.79 (d, *J* = 8.7 Hz, 1H), 7.64 – 7.60 (m, 1H), 7.60 – 7.54 (m, 6H), 7.50 – 7.45 (m, 3H), 3.95 (s, 3H); ^{13}C{^{1}H} NMR (150 MHz, $CDCl_3$) δ 169.2, 166.4, 165.9, 165.9, 139.1, 137.8, 136.8, 135.8, 135.4, 133.5, 132.9, 130.6, 129.7, 129.5, 129.2, 128.9, 128.4, 127.6, 126.2, 126.1, 125.8, 116.3, 52.5; FTIR (ATR) $\tilde{\nu}$ 3464, 3347, 1757, 1729, 1689, 1623, 1603, 1525, 1491, 1441, 1388, 1352, 1282, 1190, 1107, 1019, 1001, 846, 766, 756, 749, 687, 633, 568, 505; HRMS

(ESI-TOF) *m/z* $[M+H]^+$ Calculated for $C_{29}H_{21}N_2O_5^+$ 477.1445, found 477.1445 (|Δ*m/z*|= 0 ppm).

Aromatic 2r

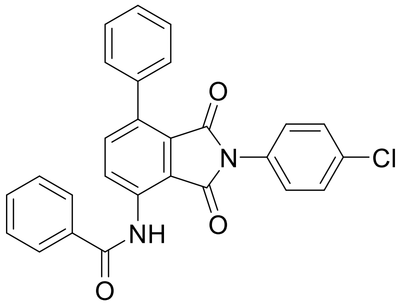

Prepared according to Procedure C and purified by Method B, starting with pyrone 1a (29.1 mg, 0.1 mmol, 1.0 equiv.) and *N*-(4-chlorophenyl)maleimide (41.5 mg, 0.2 mmol, 2.0 equiv.). Yield: 68% (30.6 mg, 0.068 mmol); Pale yellow solid; mp: 282–283 ºC; ^{1}H NMR (500 MHz, $CDCl_3$) δ 10.89 (s, 1H), 9.12 (d, *J* = 8.7 Hz, 1H), 8.09 – 8.04 (m, 2H), 7.78 (d, *J* = 8.8 Hz, 1H), 7.63 – 7.52 (m, 5H), 7.50 – 7.43 (m, 5H), 7.42 – 7.38 (m, 2H); ^{13}C{^{1}H} NMR (125 MHz, $CDCl_3$) δ 169.4, 166.1, 165.9, 138.9, 137.8, 136.7, 135.8, 134.3, 133.5, 132.9, 129.8, 129.5, 129.5, 129.2, 128.9, 128.4, 127.9, 127.6, 126.1, 125.7, 116.3; FTIR (ATR) $\tilde{\nu}$ 3343, 3057, 1757, 1688, 1622, 1596, 1526, 1491, 1392, 1354, 1253, 1195, 1180, 1094, 1019, 962, 848, 815, 752, 698, 686, 568, 509, 453; HRMS (ESI-TOF) *m/z* $[M+H]^+$ Calculated for $C_{27}H_{18}ClN_2O_3^+$ 453.1000, found 453.1000 (|Δ*m/z*|= 0 ppm).

Aromatic 2s

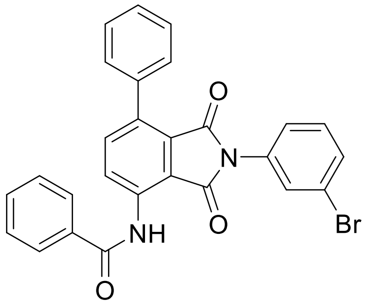

Prepared according to Procedure C and purified by Method B, starting with pyrone 1a (29.1 mg, 0.1 mmol, 1.0 equiv.) and *N*-(3-bromophenyl)maleimide (50.4 mg, 0.2 mmol, 2.0 equiv.). Yield: 58% (29.0 mg, 0.058 mmol); Yellow solid; mp: 278–279 ºC; ^{1}H NMR (500 MHz, $CDCl_3$) δ 10.88 (s, 1H), 9.12 (d, *J* = 8.7 Hz, 1H), 8.09 – 8.06 (m, 2H), 7.78 (d, *J* = 8.7 Hz, 1H), 7.65 (t, *J* = 1.9 Hz, 1H), 7.61 – 7.53 (m, 6H), 7.51 – 7.39 (m, 5H); ^{13}C{^{1}H} NMR (125 MHz, $CDCl_3$)

δ 169.2, 165.9, 165.9, 139.0, 137.8, 136.8, 135.7, 133.5, 132.9, 132.5, 131.5, 130.5, 129.7, 129.5, 129.2, 128.9, 128.4, 127.6, 126.1, 125.7, 125.2, 122.6, 116.2; FTIR (ATR) $\tilde{\nu}$ 3348, 3062, 1757, 1689, 1594, 1525, 1479, 1431, 1387, 1354, 1256, 1191, 1104, 1076, 838, 770, 751, 694, 682, 623, 567, 511, 459; HRMS (ESI-TOF) *m/z* $[M+H]^+$ Calculated for $C_{27}H_{18}BrN_2O_3^+$ 497.0495, found 497.0484 (|Δ*m/z*|= 2.2 ppm).

Aromatic 2t

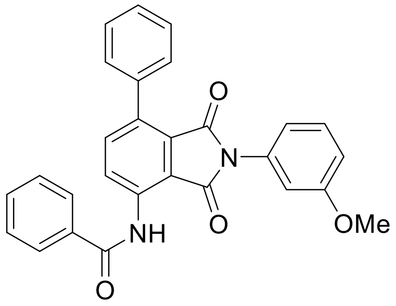

Prepared according to Procedure C and purified by Method B, starting with pyrone 1a (29.1 mg, 0.1 mmol, 1.0 equiv.) and *N*-(3-methoxyphenyl)maleimide (40.6 mg, 0.2 mmol, 2.0 equiv.). Yield: 49% (22.0 mg, 0.049 mmol); Yellow solid; mp: 219–220 ºC; ^{1}H NMR (500 MHz, $CDCl_3$) δ 10.95 (s, 1H), 9.11 (d, *J* = 8.7 Hz, 1H), 8.10 – 8.05 (m, 2H), 7.77 (d, *J* = 8.7 Hz, 1H), 7.62 – 7.58 (m, 3H), 7.56 – 7.52 (m, 2H), 7.49 – 7.38 (m, 4H), 7.02 – 7.00 (m, 1H), 6.97 – 6.94 (m, 2H), 3.82 (s, 3H); ^{13}C{^{1}H} NMR (125 MHz, $CDCl_3$) δ 169.6, 166.3, 165.9, 160.3, 138.8, 137.7, 136.6, 135.8, 133.5, 132.8, 132.2, 130.0, 129.6, 129.2, 128.8, 128.3, 127.6, 126.3, 125.5, 119.1, 116.5, 114.5, 112.6, 55.6; FTIR (ATR) $\tilde{\nu}$ 3341, 3055, 1758, 1700, 1687, 1605, 1595, 1526, 1491, 1389, 1355, 1289, 1250, 1189, 1107, 897, 842, 746, 687, 625, 567, 513; HRMS (ESI-TOF) *m/z* $[M+H]^+$ Calculated for $C_{28}H_{21}N_2O_4^+$ 449.1496, found 449.1497 (|Δ*m/z*|= 0.2 ppm).

Aromatic 2u

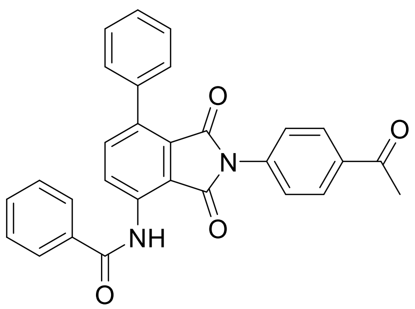

Prepared according to Procedure C and purified by Method B, starting with pyrone 1a (29.1 mg, 0.1 mmol, 1.0 equiv.) and *N*-(4-acetylphenyl)maleimide (43.0 mg, 0.2 mmol, 2.0 equiv.).

Yield: 50% (23.4 mg, 0.050 mmol); Light yellow solid; mp: 310–311 ºC; ^{1}H NMR (500 MHz, $CDCl_3$) δ 10.89 (s, 1H), 9.13 (d, *J* = 8.7 Hz, 1H), 8.10 – 8.06 (m, 4H), 7.79 (d, *J* = 8.7 Hz, 1H), 7.64 – 7.54 (m, 7H), 7.51 – 7.45 (m, 3H), 2.64 (s, 3H); $^{13}C\{^{1}H\}$ NMR (125 MHz, $CDCl_3$) δ 169.2, 165.9, 165.9, 139.1, 137.9, 136.8, 135.7, 133.5, 132.9, 129.5, 129.3, 129.2, 128.9, 128.4, 127.6, 126.4, 125.8, 26.5; FTIR (ATR) $\tilde{\nu}$ 3341, 3053, 1758, 1700, 1683, 1601, 1526, 1491, 1481, 1445, 1384, 1350, 1266, 1196, 1182, 1095, 1078, 963, 894, 820, 751, 687, 634, 589, 567, 492; HRMS (ESI-TOF) *m/z* $[M+H]^+$ Calculated for $C_{29}H_{21}N_2O_4^+$ 461.1496, found 461.1494 (|Δ*m/z*|= 0.4 ppm).

Aromatic 2v

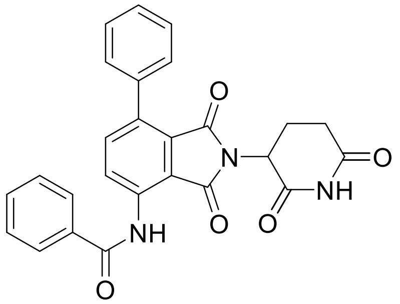

Prepared according to Procedure C and purified by Method A, starting with pyrone 1a (29.1 mg, 0.1 mmol, 1.0 equiv.) and 2,6-Piperidinedione, 3-(2,5-dihydro-2,5-dioxo-1H-pyrrol-1-yl) (41.6 mg, 0.2 mmol, 2.0 equiv.). Yield: 47% (21.4 mg, 0.047 mmol); Light yellow solid; mp: 255–256 ºC; ^{1}H NMR (600 MHz, $CDCl_3$) δ 10.65 (s, 1H), 9.05 (d, *J* = 8.6 Hz, 1H), 8.27 (s, 1H), 8.05 – 8.02 (m, 2H), 7.73 (d, *J* = 8.6 Hz, 1H), 7.63 – 7.59 (m, 1H), 7.57 – 7.53 (m, 4H), 7.49 – 7.44 (m, 3H), 4.96 (dd, *J* = 12.7, 5.4 Hz, 1H), 2.91 – 2.86 (m, 1H), 2.83 – 2.77 (m, 1H), 2.74 – 2.68 (m, 1H), 2.17 – 2.12 (m, 1H); $^{13}C\{^{1}H\}$ NMR (150 MHz, $CDCl_3$) δ 170.9, 169.4, 168.0, 166.3, 165.8, 138.8, 137.6, 136.7, 135.7, 133.5, 132.9, 129.4, 129.2, 128.9, 128.4, 127.5, 126.3, 125.7, 116.4, 49.4, 31.5, 22.7; FTIR (ATR) $\tilde{\nu}$ 3373, 3280, 2921, 1686, 1621, 1600, 1518, 1488, 1400, 1349, 1324, 1289, 1255, 1198, 1185, 1129, 1032, 897, 836, 756, 701, 607, 573, 460, 404; HRMS (ESI-TOF) *m/z* $[M+H]^+$ Calculated for $C_{26}H_{20}N_3O_5^+$ 454.1398, found 454.1396 (|Δ*m/z*|= 0.4 ppm).

Aromatic 2w

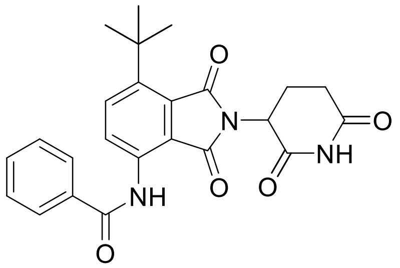

Prepared according to Procedure C and purified by Method A, starting with pyrone 1e (27.1 mg, 0.1 mmol, 1.0 equiv.) and 2,6-Piperidinedione, 3-(2,5-dihydro-2,5-dioxo-1H-pyrrol-1-yl) (41.6 mg, 0.2 mmol, 2.0 equiv.). Yield: 42% (18.3 mg, 0.042 mmol); Light yellow solid; mp: 165–166 ºC; ^{1}H NMR (600 MHz, $CDCl_3$) δ 10.76 (s, 1H), 8.92 (d, *J* = 9.0 Hz, 1H), 8.26 (s, 1H), 8.03 – 8.00 (m, 2H), 7.77 (d, *J* = 9.0 Hz, 1H), 7.61 – 7.57 (m, 1H), 7.55 – 7.51 (m, 2H), 5.01 (dd, *J* = 12.6, 5.5 Hz, 1H), 2.95 – 2.90 (m, 1H), 2.88 – 2.81 (m, 1H), 2.78 – 2.72 (m, 1H), 2.21 – 2.16 (m, 1H), 1.50 (s, 9H); ^{13}C{^{1}H} NMR (150 MHz, $CDCl_3$) δ 170.9, 169.7, 168.0, 166.9, 165.8, 147.4, 136.7, 134.9, 133.7, 132.7, 129.1, 127.5, 125.7, 117.5, 49.4, 35.4, 31.5, 29.9, 22.8; FTIR (ATR) $\tilde{\nu}$ 3337, 3198, 2958, 1696, 1681, 1624, 1522, 1496, 1407, 1324, 1300, 1258, 1188, 1130, 899, 841, 691, 588, 566, 455, 419; HRMS (ESI-TOF) *m/z* $[M+H]^+$ Calculated for $C_{24}H_{24}N_3O_5^+$ 434.1710, found 434.1708 (|Δ*m/z*|= 0.5 ppm).

Aromatic 2w'

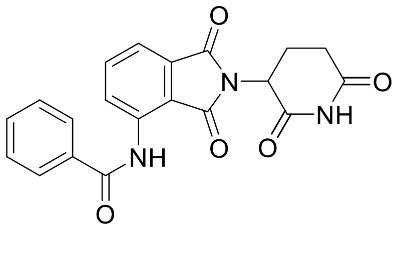

Obtained as minor product alongside with Aromatic 2w. Yield: 19% (7.2 mg, 0.019 mmol); Light yellow solid; mp: 258–259 ºC; ^{1}H NMR (600 MHz, $CDCl_3$) δ 10.39 (s, 1H), 9.02 (d, *J* = 8.5 Hz, 1H), 8.10 (s, 1H), 8.02 – 8.00 (m, 2H), 7.81 – 7.77 (m, 1H), 7.63 – 7.59 (m, 2H), 7.56 – 7.53 (m, 2H), 5.00 (dd, *J* = 12.7, 5.5 Hz, 1H), 2.97 – 2.91 (m, 1H), 2.88 – 2.82 (m, 1H), 2.80 – 2.74 (m, 1H), 2.22 – 2.17 (m, 1H); ^{13}C{^{1}H} NMR (150 MHz, $CDCl_3$) δ 170.7, 169.6, 167.8, 166.8, 165.8, 138.3, 136.8, 133.5, 132.9, 131.3, 129.2, 127.5, 125.5, 118.8, 116.0, 49.5, 31.5, 22.8; FTIR (ATR) $\tilde{\nu}$ 3362, 3198, 3086, 1770, 1690, 1618, 1535, 1477, 1396, 1350, 1292, 1259, 1200, 1180, 1115, 1020, 818, 741, 704, 606, 548, 421, 406; HRMS (ESI-TOF) *m/z* $[M+H]^+$ Calculated for $C_{20}H_{16}N_3O_5^+$ 378.1085, found 378.1082 (|Δ*m/z*|= 0.8 ppm).

Adduct 3a

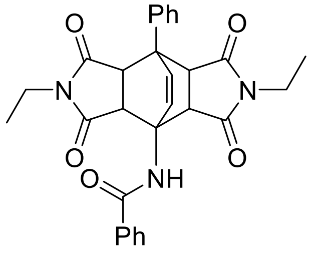

Prepared according to Procedure D, starting with pyrone 1a (29.1 mg, 0.1 mmol, 1.0 equiv.). Yield: 94% (46.6 mg, 0.094 mmol); White solid; mp: 291–292 ºC; ^{1}H NMR (600 MHz, $CDCl_3$) δ 7.96 – 7.92 (m, 2H), 7.65 (dd, *J* = 7.9, 2.5 Hz, 1H), 7.56 – 7.51 (m, 2H), 7.49 – 7.46 (m, 2H), 7.45 (dd, *J* = 7.4, 1.7 Hz, 1H), 7.41 (tt, *J* = 7.4, 1.2 Hz, 1H), 7.34 (d, *J* = 7.9 Hz, 1H), 6.93 (s, 1H), 6.79 (d, *J* = 8.9 Hz, 1H), 6.18 (d, *J* = 8.9 Hz, 1H), 4.53 (d, *J* = 8.4 Hz, 2H), 3.50 (d, *J* = 8.4 Hz, 2H), 3.34 (q, *J* = 7.2 Hz, 4H), 0.99 (t, *J* = 7.2 Hz, 6H); ^{13}C{^{1}H} NMR (150 MHz, $CDCl_3$) δ 174.4, 173.9, 169.6, 136.7, 135.4, 131.9, 130.7, 129.0, 128.9, 128.3, 127.9, 127.4, 127.1, 126.7, 58.4, 49.3, 46.6, 43.7, 33.8, 13.0; FTIR (ATR) $\tilde{\nu}$ 3332, 2978, 1767, 1696, 1540, 1441, 1399, 1345, 1303, 1226, 1128, 1005, 807, 711, 692, 524; HRMS (ESI-TOF) *m/z* $[M+H]^+$ Calculated for $C_{29}H_{28}N_3O_5^+$ 498.2024, found 498.2022 (|Δ*m/z*|= 0.4 ppm).

Compound 4

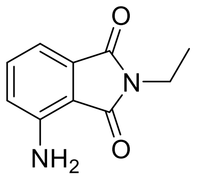

Prepared according to Procedure E, starting with Aromatic 2h (26.9 mg, 0.116 mmol, 1.0 equiv.). Yield: 81% (18.0 mg, 0.095 mmol); or prepared according to Procedure E', starting with Pyrone 1e (27.1 mg, 0.1 mmol, 1.0 equiv.). Yield: 38% (7,2 mg, 0.038 mmol); Yellow solid; mp: 135–136 ºC; ^{1}H NMR (500 MHz, $CDCl_3$) δ 7.39 (dd, *J* = 8.3, 7.2 Hz, 1H), 7.14 (dd, *J* = 7.2, 0.8 Hz, 1H), 6.83 (dd, *J* = 8.3, 0.8 Hz, 1H), 5.19 (s, 2H), 3.68 (q, *J* = 7.2 Hz, 2H), 1.25 (t, *J* = 7.2 Hz, 3H); ^{13}C{^{1}H} NMR (125 MHz, $CDCl_3$) δ 170.3, 168.7, 145.2, 135.2, 133.1, 121.1, 112.8, 111.7, 32.6, 14.2; FTIR (ATR) $\tilde{\nu}$ 3456, 3333, 3195, 1746, 1677, 1633, 1480, 1445, 1409, 1183, 1023, 878, 817, 744, 578, 421; HRMS (ESI-TOF) *m/z* $[M+H]^+$ Calculated for $C_{10}H_{11}N_2O_2^+$ 191.0815, found 191.0814 (|Δ*m/z*|= 0.5 ppm).

Compound 5

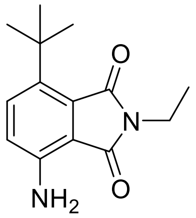

Prepared according to Procedure F, starting with Aromatic 2e (15.9 mg, 0.045 mmol, 1.0 equiv.). Yield: 77% (8.6 mg, 0.035 mmol); Yellow solid; mp: 135–136 ºC; ^{1}H NMR (600 MHz, $CDCl_3$) δ 7.41 (d, *J* = 8.7 Hz, 1H), 6.78 (d, *J* = 8.7 Hz, 1H), 5.24 (s, 2H), 3.68 (q, *J* = 7.2 Hz, 2H), 1.45 (s, 9H), 1.25 (t, *J* = 7.2 Hz, 3H); ^{13}C{^{1}H} NMR (150 MHz, $CDCl_3$) δ 170.4, 168.5, 143.8, 141.1, 133.6, 128.9, 121.2, 113.3, 34.8, 32.6, 30.1; FTIR (ATR) $\tilde{\nu}$ 3449, 3349, 2961, 1740, 1678, 1632, 1494, 1459, 1405, 1344, 1186, 1044, 899, 822, 769, 554; HRMS (ESI-TOF) *m/z* $[M+H]^+$ Calculated for $C_{14}H_{19}N_2O_2^+$ 247.1441, found 247.1439 (|Δ*m/z*|= 0.8 ppm).

Compound 6

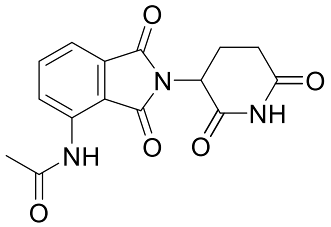

Prepared according to Procedure G, starting with pyrone 1h (15.3 mg, 0.1 mmol, 1.0 equiv.). Yield: 32% (10.2 mg, 0.032 mmol); White solid; mp: 250–252 ºC; ^{1}H NMR (600 MHz, $CDCl_3$) δ 11.15 (s, 1H), 9.73 (s, 1H), 8.44 (d, *J* = 8.4 Hz, 1H), 7.83 (dd, *J* = 8.4, 7.4 Hz, 1H), 7.62 (d, *J* = 7.4 Hz, 1H), 5.14 (dd, *J* = 12.9, 5.4 Hz, 1H), 2.90 (ddd, *J* = 17.2, 13.9, 5.4 Hz, 1H), 2.65 – 2.51 (m, 2H), 2.19 (s, 3H), 2.13 – 2.03 (m, 1H); ^{13}C{^{1}H} NMR (150 MHz, $CDCl_3$) δ 172.8, 169.8, 169.3, 167.5, 166.7, 136.5, 136.1, 131.5, 126.4, 118.4, 117.1, 48.9, 30.9, 24.1, 22.0; FTIR (ATR) $\tilde{\nu}$ 3417, 3284, 3163, 1736, 1688, 1624, 1506, 1442, 1376, 1334, 1274, 1248, 1194, 1184, 816, 742, 600, 571, 446. The spectral data is consistent with literature data.[6]

Compound 7

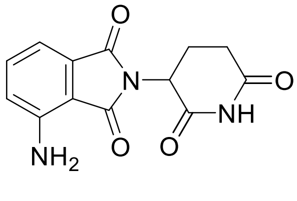

Prepared according to Procedure H, starting with compound 6 (13.5 mg, 0.043 mmol, 1.0 equiv.). Yield: 45% (5.3 mg, 0.019 mmol); Yellow solid; mp: 314–316 ºC (decomposition); ^{1}H NMR (500 MHz, DMSO) δ 11.08 (s, 1H), 7.47 (dd, J = 8.4, 7.0 Hz, 1H), 7.02–6.98 (m, 2H), 6.52 (s, 2H), 5.04 (dd, J = 12.8, 5.4 Hz, 1H), 2.88 (ddd, J = 16.8, 13.8, 5.4 Hz, 1H), 2.62 – 2.51 (m, 2H), 2.05 – 1.97 (m, 1H); ^{13}C{^{1}H} NMR (125 MHz, DMSO) δ 172.8, 170.1, 168.6, 167.4, 146.7, 135.5, 132.0, 121.7, 111.0, 108.5, 48.5, 31.0, 22.1; FTIR (ATR) $\tilde{\nu}$ 3473, 3370, 3241, 3099, 1703, 1687, 1631, 1477, 1407, 1357, 1319, 1257, 1189, 1015, 815, 742, 594, 472. The spectral data is consistent with literature data.[7]

Computational Studies

All molecular structures were initially generated and pre-optimized using the Rowan Scientific web server.[8] The pre-optimizations employed the Organic-focused Neural Network Potential (AIMNet2), a machine learning-based interatomic potential capable of rapidly optimizing geometries. AIMNet2 was trained to reproduce data at the ωB97M-D3/def2-TZVPP level of theory and was used here as a reliable and efficient starting point for subsequent calculations.[8–11]

For the evaluation of HOMO–LUMO gaps, the pre-optimized geometries were submitted to a conformational sampling with the CREST package[12] at the semi-empirical extended tight-binding GFN2-xTB level.[13,14] The resulting ensembles were further refined with Density Functional Theory (DFT) methods implemented in the ORCA 6.0.0 program.[15] Multiple theoretical levels with different energy cutoffs were applied to filter conformers, following previously validated protocols.[16,17] Thermostatistical contributions to free energies were computed from single-point Hessians (SPH) using the modified rigid-rotor harmonic oscillator (mRRHO) approximation,[18] in order to speed up the calculations while retaining reliable accuracy, as demonstrated in prior studies.[19–22] The SPH calculations were performed at the GFN2-xTB level with the ALPB solvation model[23] for toluene at 473.15 K at the xTB 6.7.1 software. In addition, the SMD implicit solvation model[24] for toluene was consistently applied across all optimization stages.

The refinement DFT protocol consisted of sequential optimization steps. In the first stage, electronic energies were calculated at the B3LYP-D4/6-31G level,[25–27] and only structures within 6.5 kcal·mol^{-1} of the lowest-energy conformer were retained. These geometries were then re-optimized at the B3LYP-D4/6-311++G(d,p) level,[25–27] with conformers higher than 4

kcal·mol^{-1} being discarded. Finally, the remaining ensemble was refined with a final optimization at the PBE0-D4/6-311++G(d,p) level,[26–28] and structures lying more than 3 kcal·mol^{-1} above the global minimum were removed. For the final conformer ensemble, the HOMO–LUMO gap values (in eV) were evaluated and reported as Boltzmann-weighted averages over the remaining structures.

For the investigation of reaction pathways and mechanisms, all structures - including reactants, products, intermediates, and transition states (TS) - were fully optimized, and their thermodynamic contributions were computed at the high-accuracy ωB97X-D4/def2-TZVP level[29,30] with the CPCM implicit solvation model [31] for toluene. The initial transition-state guess geometries were generated and pre-optimized as TS structures using the AIMNet2 machine learning potential implemented in Rowan. These structures were subsequently refined at the ωB97X-D4/def2-TZVP level with CPCM solvation for toluene as implemented in the ORCA 6.0.0 program, where transition-state optimizations were performed and vibrational frequency analyses were carried out to confirm their nature as a TS structure and provide their thermodynamic corrections. In specific cases, such as the decarboxylation step, transition states were identified through multidimensional scans using the ωB97X-D4/def2-TZVP/CPCM method, followed by optimization at the same level of theory. Frequency calculations were systematically performed to verify the presence of a single imaginary mode corresponding to the transition vibration. To further validate the connectivity of each transition state, intrinsic reaction coordinate (IRC) calculations[32] were conducted in both forward and backward directions at the same level of theory as the TS optimization, using a maximum of 30 iterations per direction.

To refine the energies along the reaction pathway, single-point electronic energies were calculated at the DLPNO-CCSD(T) level of theory with the def2-TZVPP basis set. The RIJCOSX approximation was employed in combination with the def2-TZVPP/C and def2/J auxiliary basis sets. Solvent effects of toluene were modeled using the CPCM approach, and Tight SCF convergence criteria were applied in all calculations. The final Gibbs free energies were obtained by combining the electronic energies from the DLPNO-CCSD(T)/def2-TZVPP/CPCM single-point calculations with the thermochemical corrections (at 473.15 K) derived from frequency calculations at the ωB97X-D4/def2-TZVP/CPCM level of theory.

HOMO-LUMO Energy gap

The energy gap was calculated as the difference between the Highest Occupied Molecular Orbital (HOMO) of the diene (**3A2P**, **3A6M2P**, and **3B6P2P**) and the Lowest Unoccupied Molecular Orbital (LUMO) of the dienophile (**EVE** and **NEM**).

Table S5. Boltzmann-weighted HOMO energies (eV) of the dienes and LUMO energies (eV) of the dienophiles.

HOMO [eV]			LUMO [eV]	
**3A2P**	**3A6M2P**	**3B6P2P**	**EVE**	**NEM**
-6.506	-6.283	-6.134	0.170	-2.595

Table S6. The HOMO-LUMO energy gap (eV) calculated between the HOMO of each diene and the LUMO of the dienophiles **EVE** and **NEM** (Normal Demand).

GAP [eV]	3A2P	3A6M2P	3B6P2P
**EVE**	-6.677	-6.453	-6.304
**NEM**	-3.912	-3.688	-3.539

The HOMO–LUMO energy gaps, in decreasing order, are as follows:

When **EVE** is the dienophile:

3A2P > 3A6M2P > 3B6P2P

When **NEM** is the dienophile:

3A2P > 3A6M2P > 3B6P2P

Table S7. Electronic energy ($E_{electronic}$), thermostatistical contributions (G_{rrho}), and total Gibbs free energies (G_{total}) for each conformation of each compound. All values are given in Hartree.

Compound	Conformation	$E_{electronic}$ [Eh]	G_{rrho} [Eh]	G_{total} [Eh]
**3A2P**	1	-550.9607603	0.0637313	-550.8970290
	2	-550.9610308	0.0637986	-550.8972321
**3A6M2P**	1	-590.2496797	0.0855238	-590.1641559
**3B6P2P**	1	-973.3926155	0.1705615	-973.2220539
**EVE**	1	-232.2317307	0.0579860	-232.1737446
	2	-232.2295021	0.0587356	-232.1707665
	3	-232.2282875	0.0571656	-232.1711219
	4	-232.2284699	0.0559961	-232.1724738

	5	-232.2284630	0.0559922	-232.1724708
**NEM**	1	-437.7141277	0.0601987	-437.6539290

Optimized coordinates of conformation 1 for compound **3A2P**:

O	2.46240649405735	0.57827845324745	-0.00019059043335
C	1.16978125722201	0.99914559312111	0.00071017541619
C	0.14363568757981	-0.03557338354822	0.00049673798987
C	0.49484845835428	-1.35214831299328	0.00031652890038
C	1.87570954188541	-1.70057057317617	-0.00008328009757
C	2.80172774803812	-0.72782195315749	-0.00042714969186
O	0.93973121041908	2.18775239293439	-0.00041315159752
N	-1.13325113544324	0.49827805159101	0.00059640073658
C	-2.32553484785118	-0.17809262037270	0.00046377045773
C	-3.53127645185976	0.72080920055465	-0.00029550166983
O	-2.39217346020235	-1.39355681914664	-0.00022689594739
H	-0.27059475909608	-2.11550654986806	0.00040155819791
H	2.18787815138814	-2.73663444813372	-0.00026828383213
H	3.87473782846827	-0.86601954834714	-0.00089569542443
H	-1.15233997765006	1.51132868568414	0.00074591619362
H	-4.43195362358530	0.10910379077072	0.00039338303613
H	-3.53129219087674	1.36669819548828	0.88279465464381
H	-3.53145554464775	1.36477387225166	-0.88480853007813

Optimized coordinates of conformation 1 for compound **3A2P**:

O	2.46466632267375	0.57652068079378	-0.00025664887524
C	1.17274964212376	1.00072183436878	-0.00054656728824
C	0.14492808821837	-0.03182917377945	-0.00031962894482
C	0.49253097121008	-1.34925912304197	0.00016636732192
C	1.87242876144587	-1.70106991887533	0.00043793075610
C	2.80042417561622	-0.73009042276012	0.00021474098858
O	0.94580461668871	2.18973419159496	-0.00102250099629
N	-1.13166311312001	0.50517458431950	-0.00070198603702
C	-2.32184874580626	-0.17094392240717	-0.00048093549578
C	-3.54335824832388	0.70497108444078	0.00097804564708
O	-2.38900338404281	-1.38725982145054	-0.00039522421506
H	-0.27537717321065	-2.11016229668491	0.00028275813596
H	2.18236712897137	-2.73777599607364	0.00080906454934
H	3.87311869388027	-0.87114092243021	0.00036542225401
H	-1.14850672364436	1.51760338620721	-0.00090764077906
H	-4.15028823601292	0.45934706510404	-0.87402253493998
H	-4.13811941095029	0.47291714548769	0.88811452058705
H	-3.31682312581724	1.77244738858660	-0.00841339526855

Optimized coordinates for compound **3A6M2P**:

C	0.71170624318641	1.13242487515378	-0.00117480882174
O	2.03291441202004	0.80685344468001	-0.00119255629515
C	-0.23182603345745	0.02771406718835	0.00005975741183
C	0.22479861467902	-1.25553067305457	0.00127359497837
C	1.62579657432072	-1.50101636629133	0.00121263809047
C	2.49459154355021	-0.46891200519710	-0.00004032913608
O	0.39902259916588	2.30297971097803	-0.00244745057068

N	-1.54937595865394	0.45945343505781	-0.00020403443742
C	-2.68290468762405	-0.30743692018562	0.00036774777999
C	-3.95688658457010	0.49314636496086	0.00006357275757
O	-2.65533400852831	-1.52528396540446	0.00066988842215
C	3.97372395280261	-0.53397094459697	-0.00042395462684
H	-0.47816017526360	-2.07709084019460	0.00222410905718
H	2.00451601674791	-2.51464309405504	0.00213606563960
H	-1.64792951718517	1.46749117313200	-0.00099364608815
H	-4.80558358524611	-0.18892503215924	-0.00484924283347
H	-4.01161444872066	1.13176710703300	0.88676410788780
H	-4.00716946955508	1.13950756076779	-0.88123837952972
H	4.30609572992390	-1.57246893650076	0.00061868187794
H	4.38200061525391	-0.03245494070559	-0.88385773519673
H	4.38256312225391	-0.03049132740635	0.88162909743314

Optimized coordinates for compound **3B6P2P**:

O	1.87449085016030	-0.93239572374387	0.12027780141707
C	0.52355379428163	-1.07361907502351	0.14532631887151
C	-0.25894685853977	0.14331658810673	0.02665645852565
C	0.37357648271813	1.34608031661181	-0.09280620340475
C	1.78907160218602	1.39616705147803	-0.10610432033450
C	2.51625361190805	0.25655230205320	-0.00201413277064
O	0.05426565558028	-2.18402095114651	0.26179009083764
N	-1.61930791557969	-0.10338551099000	0.05030548205966
C	-2.63043509869534	0.81338615334807	-0.01987110579248
C	-4.00855237627813	0.24107379556648	-0.02216598247783
O	-2.42775341788908	2.01588808045625	-0.06749629065533
C	3.97226405224115	0.12118054071303	-0.00257116404860
C	4.56578491330050	-1.13484669407674	0.17044448169539
C	5.94764798782010	-1.26505802156030	0.17611738772499
C	6.75961457926128	-0.14971681468763	0.00894821749408
C	6.17745525209644	1.10289230959961	-0.16619548626511
C	4.79881534569021	1.23951890012796	-0.17310699655648
C	-5.04934902479650	1.08115034970024	0.37568055566079
C	-6.35652414950511	0.61750342434525	0.39007279288406
C	-6.63768854070309	-0.68591088774754	-0.00818690350484
C	-5.60702101749438	-1.52267830327564	-0.42183644555646
C	-4.29596287862535	-1.06393910562593	-0.42779670884228
H	-0.20757181150578	2.25398855167877	-0.17380569182789
H	2.28623631320989	2.35123984048747	-0.19839670402398
H	-1.84853530025629	-1.08124544375805	0.17754854242031
H	3.94065222534602	-2.00944592347263	0.30235547075502
H	6.39069824822580	-2.24606001297451	0.31276917830629
H	7.83962156726697	-0.25346755831231	0.01367755785135
H	6.80233981660858	1.97974943698832	-0.30001420024399
H	4.36827302381522	2.22423879618798	-0.31438331749455
H	-4.81459905351227	2.09709271167484	0.67320957258309
H	-7.15883667384232	1.27403725407408	0.71021079512201
H	-7.66071472144875	-1.04791401790203	-0.00154806475178
H	-5.82364580066487	-2.53454858835926	-0.74772435304752
H	-3.51138724287985	-1.72651883844176	-0.78032222390992

Optimized coordinates of conformation 1 for compound **EVE**:

C	-2.00468525584875	-0.61829935780164	0.00004365697148
C	-1.31487134171685	0.52426950254465	-0.00007429624825
O	0.01856612997591	0.69059233393894	-0.00010954723413
C	0.81571707834485	-0.48777315634938	-0.00000457242323
C	2.26468577173418	-0.07645156643219	-0.00009147857354
H	-3.08670042384269	-0.56548004655932	0.00005086646412
H	-1.54708625769497	-1.60001984350012	0.00013415296503
H	-1.81400143402372	1.48942600991047	-0.00016302559441
H	0.57345844729078	-1.08648782639219	0.88764902651926
H	0.57340741646141	-1.08668191946521	-0.88751380501194
H	2.50376872290325	0.51525562656061	-0.88778515910447
H	2.50381547094898	0.51546221416026	0.88745186973131
H	2.90134666196759	-0.96578618441488	-0.00000576046122

Optimized coordinates of conformation 2 for compound **EVE**:

C	-1.62659300579558	-0.80344741186790	-0.24983787759304
C	-1.21804319113905	0.46198767123704	-0.13849363684521
O	-0.03004625177536	0.93551221795862	0.27743837565589
C	0.97763882254299	-0.00504728309170	0.64442641900546
C	1.70717154195023	-0.56826637297557	-0.55504695781799
H	-2.63983834250453	-0.98540928058839	-0.58704498445265
H	-1.00919634787866	-1.66467055622320	-0.02519932867698
H	-1.87759750251983	1.28874099591527	-0.38868511854139
H	1.66474783458742	0.55928760565803	1.27868795533730
H	0.53154950924925	-0.79683839841500	1.25754541265440
H	2.18084512230855	0.23435711019599	-1.12701801956132
H	2.49070365028851	-1.25431527464926	-0.21865941466003
H	1.03769585808604	-1.11782890995395	-1.22061448770444

Optimized coordinates of conformation 3 for compound **EVE**:

C	-2.21621901199236	-0.39575189754833	0.25118379588994
C	-1.03526789719760	-0.15676855067781	-0.31330058143551
O	-0.11096585597131	0.65661644312124	0.23603115521567
C	1.19656250906532	0.56508943411464	-0.32575232080557
C	1.94304212332512	-0.65593578151332	0.15992001457337
H	-2.93553111727304	-1.01961447970076	-0.26439738314547
H	-2.48583412332909	0.03018350218354	1.21171064297268
H	-0.76353189302081	-0.58794445951579	-1.27794272204162
H	1.12107451329096	0.57358869606131	-1.42116034887542
H	1.70497027885495	1.48169647829395	-0.01930976264223
H	2.02483764726700	-0.65204477501017	1.25040647059565
H	1.44010834493457	-1.57878928854594	-0.14474260903590
H	2.95342506404627	-0.66710643926254	-0.26054024546561

Optimized coordinates of conformation 4 for compound **EVE**:

C	-2.39754599422944	0.32117739224558	-0.00118204385995
C	-1.23718466787712	-0.33039898225344	0.00150422208112
O	-0.04552048444373	0.29939900453936	-0.00116283862758
C	1.09551442582317	-0.55633543276076	0.00180374081667
C	2.33230279151768	0.30203010810902	-0.00087041713983
H	-3.32280483337399	-0.24106208244882	0.00130346672924
H	-2.44357259637447	1.40502196516250	-0.00573705273194

H	-1.19358832203689	-1.41975005063652	0.00612042811033
H	1.06652940831458	-1.19701383675116	0.89270424410520
H	1.06673791337302	-1.20289731296101	-0.88484116344076
H	3.22335510844636	-0.33223992073032	0.00140441905218
H	2.36550428710790	0.93658866478269	-0.89055219651331
H	2.36517196175292	0.94254862160286	0.88454012121865

Optimized coordinates of conformation 5 for compound **EVE**:

C	-2.39743626788128	0.13706485452632	-0.29046440391118
C	-1.23326008126278	-0.27320305176300	0.20673186320461
O	-0.04650233413621	0.07167322891655	-0.33121594635002
C	1.10013121993655	-0.32556299845069	0.41796275734692
C	2.33115457972066	0.05452330752726	-0.36098551832693
H	-3.31896395742640	-0.19170110995651	0.17321405180554
H	-2.45031009425634	0.78879746496913	-1.15610163379967
H	-1.18194056977490	-0.92662249204949	1.07805344895801
H	1.08602306415448	0.17430916769475	1.39513985646931
H	1.06450461313586	-1.40951310036805	0.58838860003396
H	2.36882809395068	1.13406165739853	-0.52950572765963
H	3.22651849318623	-0.23681444327465	0.19549647821684
H	2.35039563155343	-0.44967445187016	-1.33089287558779

Optimized coordinates for compound **NEM**:

C	0.50997932021818	-1.13893229152571	-0.06009196861567
C	1.89335225644539	-0.67044985529745	0.25784130080094
C	1.89728224642331	0.65970446137245	0.25800050782655
C	0.51664574182700	1.13644086759912	-0.05952116561831
N	-0.25682623528000	0.00105248575414	-0.24391059647531
C	-1.67716313908324	0.00526210871676	-0.53594072092410
C	-2.52971793833505	0.00530035813023	0.72028281401110
O	0.11421131152332	-2.27536229847524	-0.15312037252495
O	0.12763782627319	2.27522420167527	-0.15225416996911
H	2.70540634830646	-1.36042486228182	0.44284046791496
H	2.71339732948320	1.34482666362050	0.44317294340562
H	-1.87595664337491	0.89160540030357	-1.14241665110409
H	-1.88075920578081	-0.87775655436546	-1.14567475107057
H	-3.58976404293130	0.00862136399777	0.45114121454837
H	-2.33600966347549	-0.88429825703476	1.32548488363309
H	-2.33122622763924	0.89168639311063	1.32864829476149

Reaction Pathways and Mechanisms

The first reaction steps to be computationally investigated were the first Diels-Alder (DA) reaction, followed by decarboxylation, and the second Diels-Alder (DA) reaction, as shown below.

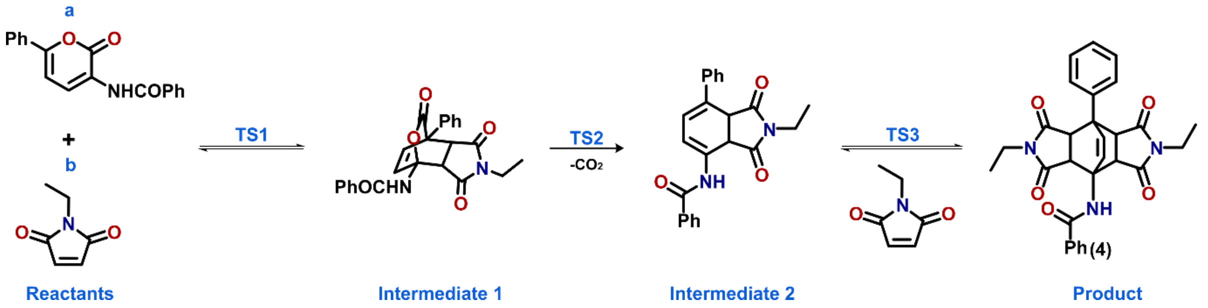

Figure S3. Reaction steps (the first Diels-Alder reaction, decarboxylation, and the second Diels-Alder reaction) that were computationally investigated.

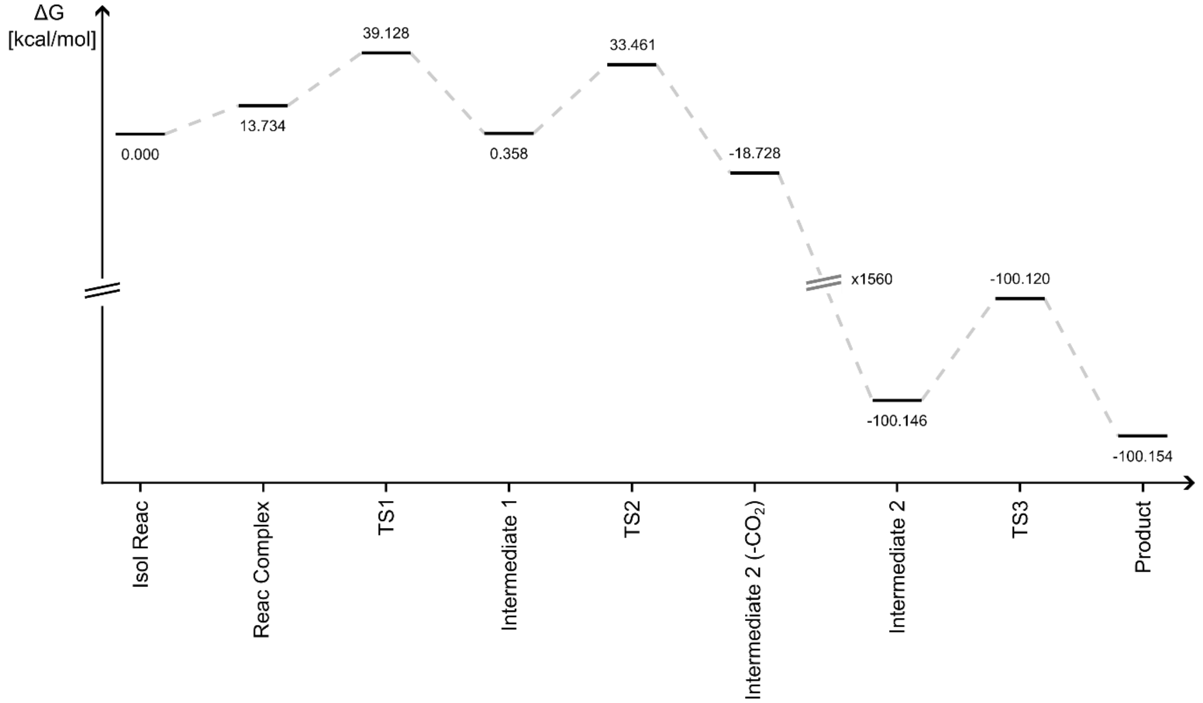

Figure S4. Reaction pathway involving the first Diels-Alder reaction, decarboxylation, and the second Diels-Alder reaction. The pathway includes the isolated reactants (Isol React), the reactant complex (Reac Complex), three transition states (TS1, TS2, TS3), two intermediates (Intermediate 1 and Intermediate 2), and the final product (Product). Gibbs free energies (ΔG, kcal mol^{-1}) were obtained by combining electronic energies from DLPNO-CCSD(T)/def2-TZVPP/CPCM single-point calculations with thermochemical corrections from ωB97X-D4/def2-TZVP/CPCM frequency calculations at 473.15 K. The energies of Intermediate 2, TS3, and the Product are presented as their values divided by a factor of 1560.

The Gibbs activation free energies ($\Delta\Delta G^{\ddagger}$) and reaction free energies (ΔΔG) for each step are summarized in the table below.

Table S8. Gibbs activation free energies ($\Delta\Delta G^{\ddagger} = \Delta G_{TS} - \Delta G_{react}$) and reaction free energies ($\Delta\Delta G = \Delta G_{prod} - \Delta G_{react}$) for each step of the reaction pathway. All values are given in kcal·mol^{-1}.

Reaction Step	ΔG‡ [kcal/mol]	ΔΔG [kcal/mol]
**1st DA**	39.128	0.358
**Decarboxylation**	33.103	-19.086
**2nd DA**	39.245	-13.730

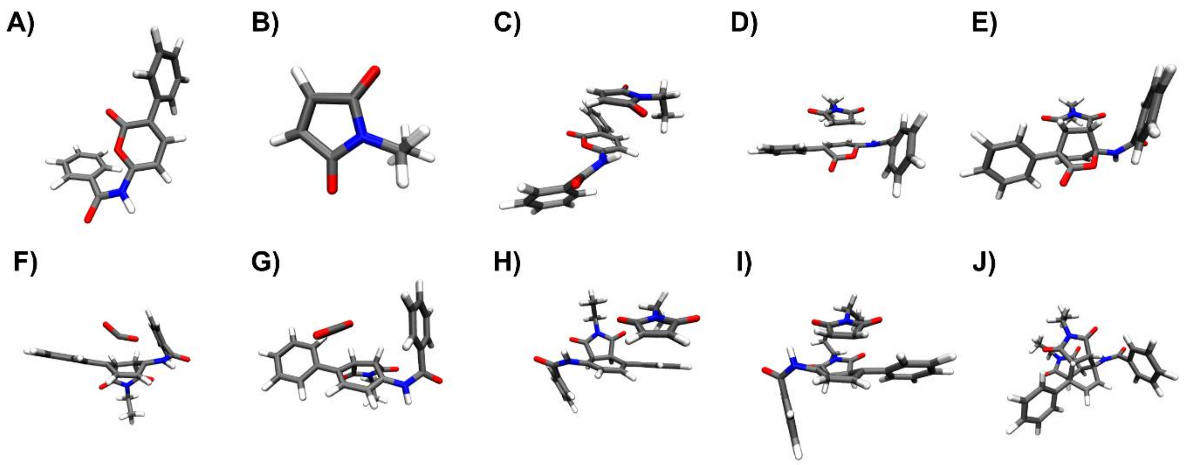

Figure S5. Optimized structures of: **A)** and **B)** the isolated reactants, **C)** the reactant complex, **D)** the transition state of the first Diels–Alder reaction, **E)** the first intermediate, **F)** the transition state of the decarboxylation, **G)** the decarboxylation intermediate, **H)** the second intermediate (reactant complex for the second Diels–Alder), **I)** the transition state of the second Diels–Alder reaction, and **J)** the final product.

The intrinsic reaction coordinate (IRC) for each reaction step is shown in the figure below.

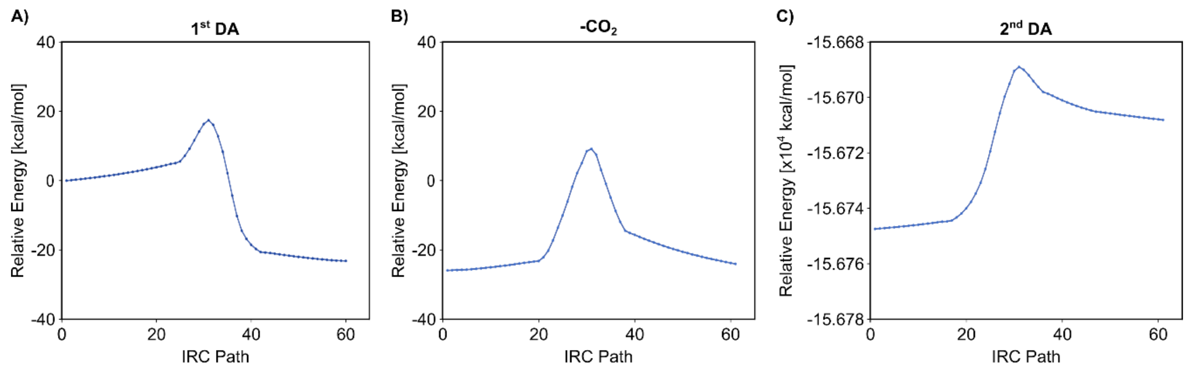

Figure S6. Calculated Intrinsic Reaction Coordinate (IRC) profiles for: **A)** the first Diels–Alder reaction, **B)** decarboxylation, and **C)** the second Diels–Alder reaction. All energy values are reported relative to the first point of IRC Path in the first Diels–Alder reaction.

The IRC path summary for the first Diels–Alder reaction is provided, with all gradients reported in Eh/Bohr.

Step	E(Eh)	dE(kcal/mol)	max(|G|)	RMS(G)	
1	-1413.332086	-17.418484	0.002079	0.000477	
2	-1413.331868	-17.281805	0.002103	0.000491	
3	-1413.331640	-17.139153	0.002191	0.000508	
4	-1413.331404	-16.990888	0.002235	0.000529	
5	-1413.331158	-16.836263	0.002322	0.000549	
6	-1413.330902	-16.675773	0.002366	0.000571	
7	-1413.330636	-16.508617	0.002456	0.000593	
8	-1413.330359	-16.334966	0.002495	0.000618	
9	-1413.330071	-16.154355	0.002579	0.000643	
10	-1413.329772	-15.966539	0.002584	0.000669	
11	-1413.329460	-15.770588	0.002686	0.000696	
12	-1413.329135	-15.566756	0.002690	0.000726	
13	-1413.328796	-15.354086	0.002752	0.000755	
14	-1413.328443	-15.132544	0.003046	0.000789	
15	-1413.328074	-14.901184	0.003179	0.000823	
16	-1413.327689	-14.659840	0.003517	0.000862	
17	-1413.327286	-14.406943	0.003708	0.000901	
18	-1413.326865	-14.142649	0.004098	0.000947	
19	-1413.326423	-13.864879	0.004350	0.000993	
20	-1413.325959	-13.573955	0.004820	0.001047	
21	-1413.325470	-13.267172	0.005113	0.001100	
22	-1413.324950	-12.940649	0.005646	0.001157	
23	-1413.324430	-12.614286	0.005875	0.001295	
24	-1413.324075	-12.391454	0.006622	0.001636	
25	-1413.323258	-11.878828	0.008603	0.001573	
26	-1413.320645	-10.239540	0.008800	0.001725	
27	-1413.317384	-8.193005	0.011625	0.002022	
28	-1413.313579	-5.805626	0.012163	0.002238	
29	-1413.309557	-3.281656	0.012112	0.002179	
30	-1413.306049	-1.080495	0.009566	0.001625	
31	-1413.304327	0.000000	0.000358	0.000062	<= TS
32	-1413.306440	-1.325373	0.011863	0.001988	
33	-1413.311675	-4.610365	0.020597	0.003699	
34	-1413.318786	-9.072672	0.029555	0.004972	
35	-1413.328597	-15.229075	0.032641	0.005788	
36	-1413.339000	-21.757388	0.030245	0.005547	
37	-1413.348360	-27.630872	0.025270	0.004507	
38	-1413.355086	-31.851660	0.013296	0.003425	
39	-1413.358878	-34.230873	0.006508	0.001983	
40	-1413.361582	-35.927701	0.005215	0.001283	
41	-1413.363514	-37.140178	0.004023	0.001002	
42	-1413.364963	-38.049620	0.004625	0.001087	
43	-1413.365160	-38.173190	0.004192	0.000899	
44	-1413.365375	-38.308052	0.003989	0.000839	
45	-1413.365679	-38.498649	0.003528	0.000713	
46	-1413.365999	-38.699717	0.003414	0.000683	
47	-1413.366305	-38.891586	0.003284	0.000651	
48	-1413.366598	-39.075183	0.003170	0.000623	
49	-1413.366877	-39.250354	0.003043	0.000595	

50	-1413.367144	-39.417949	0.002928	0.000569
51	-1413.367399	-39.577843	0.002808	0.000544
52	-1413.367643	-39.731097	0.002692	0.000520
53	-1413.367876	-39.877336	0.002580	0.000497
54	-1413.368099	-40.017060	0.002466	0.000475
55	-1413.368311	-40.150361	0.002359	0.000456
56	-1413.368513	-40.277168	0.002250	0.000443
57	-1413.368702	-40.395488	0.002150	0.000448
58	-1413.368869	-40.500431	0.002281	0.000495
59	-1413.368933	-40.540496	0.002556	0.000466
60	-1413.369010	-40.588985	0.001981	0.000382

The IRC path summary for the decarboxylation reaction is provided, with all gradients reported in Eh/Bohr.

Step	E(Eh)	dE(kcal/mol)	max(|G|)	RMS(G)
1	-1413.375320	-35.078526	0.002789	0.000401
2	-1413.375226	-35.019805	0.002869	0.000411
3	-1413.375130	-34.959302	0.002955	0.000419
4	-1413.375041	-34.903604	0.003029	0.000485
5	-1413.374969	-34.858293	0.003113	0.000524
6	-1413.374780	-34.739913	0.003235	0.000487
7	-1413.374575	-34.611363	0.003449	0.000492
8	-1413.374355	-34.473394	0.003613	0.000502
9	-1413.374124	-34.328104	0.003835	0.000520
10	-1413.373881	-34.176103	0.004022	0.000542
11	-1413.373628	-34.017100	0.004255	0.000565
12	-1413.373364	-33.851448	0.004458	0.000591
13	-1413.373088	-33.678262	0.004700	0.000617
14	-1413.372799	-33.496957	0.004921	0.000646
15	-1413.372496	-33.306852	0.005162	0.000676
16	-1413.372183	-33.110477	0.005386	0.000727
17	-1413.371844	-32.897789	0.005618	0.000757
18	-1413.371519	-32.693785	0.005796	0.000911
19	-1413.371237	-32.516830	0.005984	0.000978
20	-1413.371039	-32.392249	0.006289	0.001295
21	-1413.369290	-31.295057	0.006341	0.001516
22	-1413.366237	-29.379084	0.010000	0.002356
23	-1413.361492	-26.401303	0.015360	0.003036
24	-1413.355717	-22.777591	0.016372	0.003194
25	-1413.350014	-19.199253	0.015640	0.003322
26	-1413.343695	-15.233845	0.021675	0.003682
27	-1413.336868	-10.949815	0.022415	0.003784
28	-1413.330593	-7.012337	0.020490	0.003313
29	-1413.325962	-4.106366	0.015962	0.002701
30	-1413.320509	-0.684516	0.022335	0.004211
31	-1413.319418	0.000000	0.024190	0.004208 <= TS
32	-1413.322125	-1.698667	0.030108	0.004777
33	-1413.329126	-6.091877	0.020524	0.003706
34	-1413.335455	-10.062991	0.021464	0.003567
35	-1413.341814	-14.053229	0.023934	0.003445
36	-1413.347877	-17.858088	0.022620	0.003225
37	-1413.353006	-21.076544	0.018735	0.002950
38	-1413.357015	-23.591962	0.015517	0.003039
39	-1413.358130	-24.291619	0.019525	0.002649
40	-1413.358961	-24.813506	0.013312	0.002206
41	-1413.359879	-25.389216	0.011551	0.002019
42	-1413.360790	-25.961140	0.011176	0.001941

43	-1413.361652	-26.501914	0.010647	0.001847
44	-1413.362479	-27.021020	0.010156	0.001772
45	-1413.363268	-27.515806	0.009968	0.001689
46	-1413.364025	-27.990790	0.009472	0.001620
47	-1413.364747	-28.444371	0.009241	0.001547
48	-1413.365442	-28.879943	0.008756	0.001486
49	-1413.366106	-29.296634	0.008506	0.001420
50	-1413.366744	-29.697299	0.008051	0.001366
51	-1413.367356	-30.081127	0.007802	0.001308
52	-1413.367944	-30.450566	0.007382	0.001260
53	-1413.368509	-30.804693	0.007141	0.001207
54	-1413.369053	-31.146062	0.006759	0.001164
55	-1413.369574	-31.473278	0.006531	0.001117
56	-1413.370077	-31.788651	0.006201	0.001078
57	-1413.370559	-32.091393	0.005952	0.001038
58	-1413.371023	-32.382526	0.005681	0.000998
59	-1413.371471	-32.663521	0.005450	0.000964
60	-1413.371901	-32.933561	0.005201	0.000929
61	-1413.372316	-33.193880	0.004999	0.000899

The IRC path summary for the second Diels–Alder reaction is provided, with all gradients reported in Eh/Bohr.

Step	E(Eh)	dE(kcal/mol)	max(|G|)	RMS(G)	
1	-1663.125190	-58.405896	0.002167	0.000429	
2	-1663.124968	-58.266480	0.002228	0.000445	
3	-1663.124735	-58.120404	0.002373	0.000464	
4	-1663.124491	-57.967312	0.002451	0.000484	
5	-1663.124237	-57.808044	0.002591	0.000506	
6	-1663.123971	-57.641178	0.002674	0.000527	
7	-1663.123695	-57.467536	0.002806	0.000551	
8	-1663.123406	-57.286294	0.002889	0.000574	
9	-1663.123105	-57.097357	0.003014	0.000599	
10	-1663.122791	-56.900361	0.003099	0.000624	
11	-1663.122464	-56.695180	0.003211	0.000651	
12	-1663.122122	-56.480926	0.003298	0.000678	
13	-1663.121767	-56.257971	0.003395	0.000708	
14	-1663.121395	-56.024738	0.003481	0.000737	
15	-1663.121043	-55.803871	0.003554	0.000862	
16	-1663.120890	-55.707383	0.007111	0.001239	
17	-1663.120374	-55.384091	0.003704	0.000930	
18	-1663.118582	-54.259212	0.003910	0.001013	
19	-1663.116313	-52.835211	0.004490	0.001272	
20	-1663.113407	-51.012098	0.006562	0.001671	
21	-1663.109637	-48.646291	0.008753	0.002174	
22	-1663.104776	-45.595772	0.010639	0.002793	
23	-1663.098718	-41.794475	0.018499	0.003678	
24	-1663.090719	-36.775277	0.028054	0.004548	
25	-1663.080557	-30.398334	0.034407	0.005286	
26	-1663.069435	-23.419364	0.035279	0.005492	
27	-1663.058673	-16.666039	0.032903	0.005014	
28	-1663.049214	-10.730551	0.028538	0.004215	
29	-1663.041755	-6.049452	0.032575	0.005071	
30	-1663.034346	-1.400416	0.013136	0.002013	
31	-1663.032114	0.000000	0.000144	0.000026	<= TS
32	-1663.033747	-1.024418	0.009382	0.001371	
33	-1663.036793	-2.936029	0.011916	0.001744	
34	-1663.040249	-5.104543	0.012209	0.001848	

35	-1663.043558	-7.181348	0.011747	0.001812
36	-1663.046442	-8.991201	0.010195	0.001856
37	-1663.047410	-9.598291	0.009495	0.001666
38	-1663.048694	-10.403752	0.008900	0.001360
39	-1663.050043	-11.250711	0.008276	0.001296
40	-1663.051265	-12.017648	0.007258	0.001190
41	-1663.052416	-12.739861	0.006651	0.001104
42	-1663.053491	-13.414254	0.005790	0.001031
43	-1663.054495	-14.043967	0.005324	0.000970
44	-1663.055432	-14.632113	0.004710	0.000925
45	-1663.056304	-15.179244	0.004341	0.000904
46	-1663.057101	-15.679743	0.004175	0.000919
47	-1663.057806	-16.121614	0.004047	0.000985
48	-1663.058079	-16.293399	0.003502	0.000872
49	-1663.058431	-16.514292	0.003219	0.000750
50	-1663.058814	-16.754333	0.003216	0.000737
51	-1663.059186	-16.987864	0.002925	0.000719
52	-1663.059551	-17.216840	0.002950	0.000703
53	-1663.059908	-17.441195	0.002706	0.000688
54	-1663.060259	-17.660864	0.002747	0.000674
55	-1663.060601	-17.876039	0.002520	0.000660
56	-1663.060937	-18.086728	0.002564	0.000646
57	-1663.061267	-18.293544	0.002427	0.000634
58	-1663.061589	-18.495832	0.002403	0.000621
59	-1663.061905	-18.694331	0.002324	0.000609
60	-1663.062215	-18.888772	0.002257	0.000596
61	-1663.062519	-19.079608	0.002221	0.000585

Optimized coordinates for isolated reactant **a**:

H	-13.04277562364929	-0.82265242624791	-4.74276616828291
H	-14.25387160952776	-1.62233795526194	-2.73618160802762
H	-13.01069305444086	-2.77327550272010	-0.92122397148463
H	-8.63823105277396	-3.56640155123984	0.13370976996373
H	-5.64113142436933	-0.01563345944901	-0.99966581634699
C	-7.54261102674684	0.89548576517401	-0.97045666105737
C	-8.97655414542563	0.67088604262659	-0.87090738829002
H	-9.36142830115227	-2.27796342885668	-3.11285593389966
C	-8.51923549004569	-1.68486394727401	-0.66386342127293
H	-5.59788269430324	1.73211136983507	-2.63099744103574
H	-4.67102141889477	4.00388432298797	-2.85896165427998
C	-13.18252309820577	-1.76799263907353	-2.81850540823812
C	-12.49133740917352	-2.40692025854423	-1.79917678862873
H	-10.59831098625961	-1.16805521182956	-4.93370670637029
H	-6.52448923976223	-2.36361130840471	-0.82200815232062
H	-8.37768117900022	3.15394414626277	0.30303923058985
O	-9.36950974663701	-0.65065514101891	-0.65569482643510
O	-9.86375080666671	1.47997684943688	-0.94035952542097
H	-7.45837751501351	5.42641611554080	0.07149551770779
H	-5.59980263191494	5.86649900341822	-1.50927141517781
N	-9.13457636961069	-2.90786800855413	-0.44890905019049
C	-7.03782628090828	2.27991466479954	-1.13235190981226
C	-6.71167731561383	-0.17702950925156	-0.92975275204754
C	-7.18974772873555	-1.51210438168922	-0.80592936691267
C	-10.41560307409240	-3.30784908020371	-0.79875146188963
O	-10.90811749385042	-4.27036371893388	-0.24487019746680
C	-7.56228533984771	3.33622711350174	-0.38579016450923
C	-7.04383163793289	4.61653439297596	-0.51932280910003
C	-5.99983956732360	4.86374567620156	-1.40391297373730

C	-5.47779968727500	3.82000340054630	-2.15739302926749
C	-5.99585673068679	2.53850364874482	-2.02372442430503
C	-11.11388755102849	-2.57478812022422	-1.89510804511998
C	-10.43239465892284	-2.12952591901879	-3.02443683342363
C	-11.12813662171595	-1.50742622733533	-4.05059922892033
C	-12.50157847559226	-1.31875952262098	-3.94430318578944

The electronic energy, computed at the DLPNO-CCSD(T)/def2-TZVPP/CPCM level of theory, the thermochemical corrections obtained at the ωB97X-D4/def2-TZVP/CPCM level, the resulting total Gibbs free energy, and the first 15 vibrational frequencies of the isolated reactant **a** are:

Electronic energy: -972.520176613911 Eh
Thermochemical correction energy: 0.18696821 Eh
Total Gibbs free energy: -972.333208403910930 Eh

Vibrational Frequencies:

1: 0.00 cm**-1
2: 0.00 cm**-1
3: 0.00 cm**-1
4: 0.00 cm**-1
5: 0.00 cm**-1
6: 18.41 cm**-1
7: 27.89 cm**-1
8: 33.83 cm**-1
9: 60.05 cm**-1
10: 67.42 cm**-1
11: 70.39 cm**-1
12: 112.29 cm**-1
13: 136.04 cm**-1
14: 163.44 cm**-1
15: 200.08 cm**-1

Optimized coordinates for isolated reactant **b**:

C	-4.61654375658955	-1.53787688185575	2.37055182573376
C	-3.99520871961829	-1.60667935909477	0.98161309494688
H	-8.36315751243605	1.30614152485800	2.29382791054831
H	-9.29718070306939	-1.21417710243784	2.02178584495601
H	-4.04421274073445	-0.87642790779299	3.02272925733177
H	-4.65479038740958	-2.52589803885307	2.83167548948722
C	-7.82159566621505	0.37103906751581	2.26921984215229
C	-8.28014584030143	-0.86635743245905	2.13545322213866
H	-2.97457624910689	-1.99090176524323	1.04812333743442
C	-7.11126861766887	-1.81185687859151	2.16377253120932
N	-5.97961847944842	-1.02998860704100	2.33718908173033
C	-6.32425070516335	0.31190863640366	2.39342992306701
O	-5.55220595473102	1.23090896728052	2.51885268175633
O	-7.12606064423864	-3.01414269136563	2.06024957414017
H	-4.57143482137739	-2.27146108459685	0.33390047285011
H	-3.96028064299158	-0.61464285692628	0.52549209401737

The electronic energy, computed at the DLPNO-CCSD(T)/def2-TZVPP/CPCM level of theory, the thermochemical corrections obtained at the ωB97X-D4/def2-TZVP/CPCM level, the resulting total Gibbs free energy, and the first 15 vibrational frequencies of the isolated reactant **b** are:

Electronic energy: -437.363912887841 Eh
Thermochemical correction energy: 0.06647359 Eh
Total Gibbs free energy: -437.297439297841038 Eh

Vibrational Frequencies:

1: 0.00 cm**-1
2: 0.00 cm**-1
3: 0.00 cm**-1
4: 0.00 cm**-1
5: 0.00 cm**-1
6: 44.76 cm**-1
7: 102.90 cm**-1
8: 140.24 cm**-1
9: 207.73 cm**-1
10: 297.56 cm**-1
11: 299.87 cm**-1
12: 354.80 cm**-1
13: 439.09 cm**-1
14: 576.32 cm**-1
15: 614.95 cm**-1

Optimized coordinates for reactant complex **ab**:

H	-13.07141654470263	-1.04189842630182	-4.84261987041283
H	-14.28132602938701	-1.68817454619639	-2.78086374826243
H	-13.03845720461582	-2.70918147517802	-0.88917665631297
H	-8.65929533356680	-3.43528673518052	0.21996008870795
H	-5.65680846429816	-0.00043191853333	-1.15374049216975
C	-7.54057909943492	0.93612896991889	-1.01564754889109
C	-7.82837452867848	0.37412007381023	2.26753000841993
C	-8.30442733269877	-0.86263726239708	2.21100515774974
C	-8.54399461099066	-1.63629344417548	-0.73738712101700
C	-7.14618114055681	-1.81766517131280	2.18542571923253
N	-6.00014847840410	-1.04836151205783	2.27258158318029
C	-6.32868256237779	0.30173252944118	2.31113126013355
O	-5.53830154916279	1.20998364792155	2.37278136131543
O	-7.18262318542117	-3.02379829051086	2.09276773701149
H	-6.57049995404722	-2.33861164438578	-0.96952455650982
H	-8.30283812837506	3.14889331258895	0.38090826546201
C	-4.64200288518360	-1.56707958667326	2.34509791750278
C	-3.93766410045526	-1.57132054146018	0.99526216019543
H	-7.33823948229299	5.40802035704856	0.23998584739680
H	-5.50845571388401	5.88673103107538	-1.36244625822139
N	-9.14742952712996	-2.84929781993757	-0.44710703444771
C	-7.01528626457019	2.31737552283673	-1.12620957943125
C	-6.72599268575755	-0.14881823913281	-1.05174248611699
C	-7.22062519588015	-1.47676926329161	-0.93677840972911
C	-10.44644153672984	-3.24271192777304	-0.73015908852983
O	-10.95675130385666	-4.13376529001926	-0.08180017633264

C	-7.49940694070690	3.34619036801864	-0.31740698328092
C	-6.95631130098885	4.62019889814603	-0.40040378785995
C	-5.92820785208098	4.88872631316322	-1.29687598047161
C	-5.44662693609682	3.87275430710549	-2.11278925981107
C	-5.98832495825113	2.59717626129622	-2.02840952425717
C	-11.14308477856736	-2.59085266881755	-1.87826414536390
C	-10.46259234839142	-2.23154584806034	-3.03839997157826
C	-11.15803286041598	-1.68201340750405	-4.10574430453406
C	-12.53027882328139	-1.48013730177968	-4.01093373071293
C	-13.21063213935047	-1.84341711977174	-2.85382809716452
C	-12.51972110872829	-2.40916626327992	-1.79228170959646
H	-4.65240552924404	4.07417047506284	-2.82370087267013
H	-5.62128602530406	1.81328853360164	-2.68275233137328
H	-8.36104070376271	1.31424985947065	2.28924425382859
H	-9.33016507822619	-1.20090686187120	2.17041787180297
H	-4.09862175155795	-0.94685735857597	3.06020327015788
H	-4.71252656721916	-2.57665535228901	2.75213483695408
H	-2.91926033200850	-1.94912032810537	1.11204448211100
H	-4.46050744918409	-2.21537204509656	0.28511172336331
H	-3.88200379598814	-0.56072421030502	0.58512684055381
H	-9.39319477737339	-2.39365182907029	-3.11935138182871
H	-10.62899039803396	-1.41111107211091	-5.01269523534141
O	-9.38663744478726	-0.59269724457554	-0.71674215557907
C	-8.97578168309663	0.72812594837086	-0.88233458364921
O	-9.84842400270713	1.55610838130423	-0.88625092097825

The electronic energy, computed at the DLPNO-CCSD(T)/def2-TZVPP/CPCM level of theory, the thermochemical corrections obtained at the ωB97X-D4/def2-TZVP/CPCM level, the resulting total Gibbs free energy, and the first 15 vibrational frequencies of the reactant complex **ab** are:

Electronic energy: -1409.899662896732 Eh
Thermochemical correction energy: 0.29090153 Eh
Total Gibbs free energy: -1409.608761366732097 Eh

Vibrational Frequencies:

1: 0.00 cm**-1
2: 0.00 cm**-1
3: 0.00 cm**-1
4: 0.00 cm**-1
5: 0.00 cm**-1
6: 13.61 cm**-1
7: 20.42 cm**-1
8: 22.34 cm**-1
9: 28.56 cm**-1
10: 32.12 cm**-1
11: 38.37 cm**-1
12: 50.36 cm**-1
13: 50.92 cm**-1
14: 63.23 cm**-1
15: 66.07 cm**-1

Optimized coordinates for transition state 1 (**TS1**):

H	-14.11930834587738	-0.59313915090893	-2.22196655602750
H	-13.77895027729005	-1.79311586495005	-0.08061529037684
H	-11.73054527943351	-3.14921153300915	0.25991104278573
H	-7.75425664681511	-3.76894515635383	-1.34128963196026
H	-5.08531213251634	-0.07611565072308	-0.92995963920091
C	-7.03872438198015	0.77544220897476	-1.30742846010626
C	-8.18000887584509	0.32430881097138	0.51632551811663
C	-8.56196029744694	-1.01168624935991	0.46888779627732
C	-7.92770686550807	-1.76217391982684	-1.45913746505940
C	-7.64954982243192	-1.78147009791902	1.34865230498743
N	-6.70990083012784	-0.87644148543855	1.83203582030541
C	-7.01650632447012	0.42307338539846	1.45592323990714
O	-6.43367071116891	1.40940847740790	1.83980324830288
O	-7.66536170052473	-2.96573266862219	1.60343908180899
H	-5.91814695959492	-2.41444400276755	-1.01304609241117
H	-8.57576797567060	2.98404574308071	-0.85223747950937
C	-5.59720016173328	-1.22857585609815	2.69888013749413
C	-4.29886444432433	-1.40553259743084	1.92229499843638
H	-7.83765958526909	5.30266503289993	-0.61267747255059
H	-5.43699557997449	5.88547741875321	-0.84854627838255
N	-8.42958204910494	-3.05175757180600	-1.57180606513120
C	-6.60259867770648	2.19466261114306	-1.20343689560230
C	-6.10901988213439	-0.28215982631347	-1.21084666849691
C	-6.54830360798977	-1.57171036678564	-1.26452064995140
C	-9.72654458313357	-3.54050074809070	-1.42196968714395
O	-9.86505429365154	-4.70596504099737	-1.11561057988145
C	-7.52303900325750	3.21326877621376	-0.94822020165135
C	-7.10637065469996	4.52840042802436	-0.81773643994916
C	-5.76184130233127	4.85560579188988	-0.94947480942263
C	-4.84001814997380	3.85387196467969	-1.21866917528521
C	-5.25766940593125	2.53562326548369	-1.34413432889375
C	-10.90475138595410	-2.65318670949823	-1.65747419349959
C	-11.09686907933472	-1.98561425435400	-2.86252081822802
C	-12.25828116047185	-1.25426737696730	-3.06597300890954
C	-13.21795827326675	-1.17542541551079	-2.06336762117299
C	-13.02804614233227	-1.84740896798551	-0.86102244162065
C	-11.87964824176000	-2.60084290030603	-0.66396679806256
H	-3.78928936541435	4.09564133719062	-1.33654313906686
H	-4.51886486549365	1.77761121709626	-1.57399012157888
H	-8.83944701458535	1.16928714382554	0.38868433143540
H	-9.56689087168667	-1.35553997250446	0.27472654691190
H	-5.49946521766079	-0.43451381402211	3.44105930494954
H	-5.87534575581187	-2.15002254032953	3.21179345484890
H	-3.48858551179320	-1.67123060727876	2.60516964482898
H	-4.39697922003993	-2.20216130201385	1.18071384685136
H	-4.02588205229769	-0.47924472354302	1.41128833297737
H	-10.34688946851985	-2.04089115905204	-3.64145470528795
H	-12.41090873317470	-0.73971514573678	-4.00801029817374
O	-8.61750014783524	-0.82676718391740	-2.15667913255212
C	-8.25337740094188	0.49790432073660	-2.09165997795721
O	-8.95185367370603	1.28563636665134	-2.67040314812108

The electronic energy, computed at the DLPNO-CCSD(T)/def2-TZVPP/CPCM level of theory, the thermochemical corrections obtained at the ωB97X-D4/def2-TZVP/CPCM level, the resulting total Gibbs free energy, and the first 15 vibrational frequencies of the transition state (**TS1**) are:

Electronic energy: -1409.863931641858 Eh
Thermochemical correction energy: 0.29563848 Eh
Total Gibbs free energy: -1409.568293161858037 Eh

Vibrational Frequencies:

1: 0.00 cm**-1
2: 0.00 cm**-1
3: 0.00 cm**-1
4: 0.00 cm**-1
5: 0.00 cm**-1
6: -569.60 cm**-1 ***imaginary mode***
7: 3.25 cm**-1
8: 27.32 cm**-1
9: 32.26 cm**-1
10: 34.91 cm**-1
11: 50.21 cm**-1
12: 54.94 cm**-1
13: 66.97 cm**-1
14: 77.83 cm**-1
15: 79.59 cm**-1

Optimized coordinates for **intermediate 1**:

H	-14.05239566516842	-1.20939781834864	-0.75114944371722
H	-13.20974556058369	-3.00251955291354	0.73638354539519
H	-11.01884840554940	-4.07051790964570	0.26490269285819
H	-7.40353284566450	-3.37967973287764	-2.15979964272032
H	-4.95465065628325	0.23075533545467	-0.96432102605936
C	-7.10392413961548	0.94634852749010	-1.05197101010694
C	-8.03067702606462	0.45459851353682	0.10068016425225
C	-8.53545353075951	-0.96581163539529	-0.18761209184629
C	-7.86499348446504	-1.48195107615148	-1.48217814194311
C	-8.12776049448138	-1.78863699955919	1.01852621462786
N	-7.44255325081984	-0.96953577629460	1.88983513950909
C	-7.28647723426952	0.32398726341411	1.42609660702512
O	-6.66366478580640	1.17849818040817	2.00551822007790
O	-8.34824887457412	-2.96172715512902	1.20059689549263
H	-5.71377889911697	-2.18128215712771	-1.35962114605763
H	-8.07181934774998	3.06309042107420	0.48227290955935
C	-6.84142826013251	-1.44838001620420	3.12919679402598
C	-5.42874891136386	-1.96849638110758	2.89839917787514
H	-7.30870020928826	5.38191485681940	0.45926804608887
H	-5.42216026423506	6.07062685853602	-0.99456974251417
N	-8.18752523751820	-2.80428262028928	-1.88980609563170
C	-6.63605875892710	2.38353419365384	-0.97396594167928
C	-5.98147418651918	-0.07301499121763	-1.11988248569475
C	-6.37064837115488	-1.32086344890898	-1.32639955754942
C	-9.33573925918780	-3.54804282668992	-1.72608270931452
O	-9.28377662252065	-4.75754485943203	-1.84486634318450
C	-7.25414180774601	3.33495448505327	-0.17198032447943
C	-6.81866183482576	4.65454543557498	-0.17891783347424
C	-5.76187515445466	5.04057502521205	-0.99174598453426
C	-5.14415390713100	4.09696690043530	-1.80360647956389
C	-5.58172983876310	2.78068487942903	-1.79365347122882
C	-10.63145098756963	-2.85298445766769	-1.45552682609011
C	-11.10469218404825	-1.85189330766183	-2.29820562049039
C	-12.33994962588419	-1.26751587278989	-2.04646091326483

C	-13.09223715762074	-1.67314822244186	-0.95077981503379
C	-12.61994785018433	-2.68061548086697	-0.11492585230822
C	-11.39639171966428	-3.28068738759773	-0.37385193424008
H	-4.32277487545309	4.38617644986737	-2.45024209055252
H	-5.10295572978985	2.05889065924587	-2.44763336747452
H	-8.86548606618047	1.14285948751568	0.23358843790786
H	-9.61645197503369	-1.00008837821072	-0.29574619929853
H	-6.84406162349154	-0.61201018374436	3.82863482713449
H	-7.49240393264087	-2.23315589801031	3.51562921641123
H	-5.00272793864530	-2.31791816254333	3.84137758355387
H	-5.43625164501007	-2.80341525866162	2.19400684838202
H	-4.78756349880464	-1.17775478761446	2.50259935656697
H	-10.51442896556072	-1.53145283997679	-3.14733792066645
H	-12.71379160887071	-0.49422436912852	-2.70856976385756
O	-8.30775312535254	-0.54802604505796	-2.51727166534322
C	-7.94488622197445	0.73336986448683	-2.32486236882667
O	-8.28218486008113	1.58731609376494	-3.09487214520228

The electronic energy, computed at the DLPNO-CCSD(T)/def2-TZVPP/CPCM level of theory, the thermochemical corrections obtained at the ωB97X-D4/def2-TZVP/CPCM level, the resulting total Gibbs free energy, and the first 15 vibrational frequencies of the **intermediate 1** are:

Electronic energy: -1409.932750798310 Eh
Thermochemical correction energy: 0.30267406 Eh
Total Gibbs free energy: -1409.630076738309981 Eh

Vibrational Frequencies:

1: 0.00 cm**-1
2: 0.00 cm**-1
3: 0.00 cm**-1
4: 0.00 cm**-1
5: 0.00 cm**-1
6: 22.77 cm**-1
7: 31.11 cm**-1
8: 36.31 cm**-1
9: 40.76 cm**-1
10: 48.96 cm**-1
11: 63.36 cm**-1
12: 67.17 cm**-1
13: 69.27 cm**-1
14: 77.67 cm**-1
15: 82.52 cm**-1

Optimized coordinates for transition state 2 (**TS2**):

H	-14.03285990678578	-1.31418433767311	-0.67928992034698
H	-13.14526854338559	-3.05553068434693	0.84260118568786
H	-10.92614133156003	-4.07237811337718	0.39596749933298
H	-7.39224402662467	-3.37717095568822	-2.28692919364273
H	-4.93643677796433	0.29121440711610	-1.67978694363150
C	-6.82812513929003	0.97189156224689	-0.90337034997091
C	-7.86802521458017	0.46432353969347	0.07951229330098

C	-8.42956892606969	-0.93075757903585	-0.25463448254406
C	-7.68515047378357	-1.59966817479415	-1.38267311840883
C	-8.19167587341403	-1.76849533984248	1.00109951715638
N	-7.54085556034711	-0.99339080719049	1.92564541069386
C	-7.25926623000353	0.28443334530799	1.47538202887001
O	-6.63854158114596	1.09574338272237	2.11531827762811
O	-8.51017164269171	-2.92286384218078	1.15900174841645
H	-5.66499839835574	-2.03091079817591	-2.00688566720918
H	-7.67396371396223	2.99233284366204	0.77532460583437
C	-7.09972502763407	-1.49693166049318	3.22185845449581
C	-5.70786162519238	-2.10864456016567	3.13730133640689
H	-7.11011192035197	5.36057010199903	0.64234983623506
H	-5.53909233598685	6.18703259854527	-1.09182322999197
N	-8.11517440499306	-2.80910131257726	-1.86490899078306
C	-6.46607494356575	2.40643344379262	-0.91235086880769
C	-5.92450315959792	-0.00545741072310	-1.34333239932267
C	-6.31049878095334	-1.32295424294873	-1.50120713783927
C	-9.27225413103391	-3.55995229791619	-1.63429252279947
O	-9.20449217216876	-4.76296321206856	-1.75946462398375
C	-7.00905400806503	3.32347576804970	-0.01069590921245
C	-6.67909892635128	4.67131526870956	-0.07619900580529
C	-5.79813965899753	5.13487595820642	-1.04356884932638
C	-5.25985340792644	4.23526671826115	-1.95680437159545
C	-5.60018782451241	2.89308524816141	-1.89761867472051
C	-10.56826029210578	-2.87797358316504	-1.34660755508090
C	-11.06806498574397	-1.90869738757547	-2.21106386572516
C	-12.31844578114308	-1.35214981298090	-1.97237601686664
C	-13.06052380391909	-1.75599315243502	-0.86935787044383
C	-12.56275855969175	-2.73438337292066	-0.01390472434104
C	-11.32352508419730	-3.30674737578107	-0.25882391418241
H	-4.58462477003451	4.58326118988365	-2.73145167498016
H	-5.20439040139168	2.21967262650834	-2.64969941673890
H	-8.68690368292078	1.17924685923009	0.17885575051945
H	-9.49867418624879	-0.90695808179042	-0.44689152938709
H	-7.11824277943393	-0.65392421134370	3.91297578610257
H	-7.83593738081415	-2.23390907169167	3.54424502846362
H	-5.40389904099700	-2.47557326830903	4.12013003433068
H	-5.69751059160552	-2.94760180760328	2.43781593396759
H	-4.98019871756849	-1.36429885874782	2.80673621210266
H	-10.48666917518529	-1.59386325980796	-3.06795467809116
H	-12.71241478378833	-0.60342342543430	-2.65078059462408
O	-8.39224808022098	-0.41248498612037	-2.68674714072699
C	-8.08577246394032	0.75727006062593	-2.69564844951451
O	-8.19946149175272	1.81921162218249	-3.19891216890027

The electronic energy, computed at the DLPNO-CCSD(T)/def2-TZVPP/CPCM level of theory, the thermochemical corrections obtained at the ωB97X-D4/def2-TZVP/CPCM level, the resulting total Gibbs free energy, and the first 15 vibrational frequencies of the transition state 2 (**TS2**) are:

Electronic energy: -1409.875709437853 Eh
Thermochemical correction energy: 0.29838519 Eh
Total Gibbs free energy: -1409.577324247852857 Eh

Vibrational Frequencies:

1: 0.00 cm**-1
2: 0.00 cm**-1
3: 0.00 cm**-1
4: 0.00 cm**-1
5: 0.00 cm**-1
6: -641.74 cm**-1 ***imaginary mode***
7: 22.03 cm**-1
8: 32.17 cm**-1
9: 40.64 cm**-1
10: 48.79 cm**-1
11: 52.44 cm**-1
12: 63.85 cm**-1
13: 65.43 cm**-1
14: 72.43 cm**-1
15: 73.91 cm**-1

Optimized coordinates for **intermediate 2** with CO2:

H	-13.80914279779699	-1.14818908969921	-1.44557880806146
H	-13.46242411064171	-2.78042623663633	0.38510369130610
H	-11.31167384013825	-4.01296409577573	0.55058161002392
H	-7.21363037012193	-3.80155333438907	-1.32534610410461
H	-5.86990698396636	0.55326687713727	-2.74855974001271
C	-6.85763223686665	0.95351230330386	-0.93990319479209
C	-7.67953133984955	0.37556491325028	0.18136492757709
C	-8.35581825251562	-0.98658343622433	-0.05203176692361
C	-7.72601880972359	-1.80121852447775	-1.16653291456693
C	-8.16364336044249	-1.71246141806393	1.27916417954989
N	-7.24317784176223	-1.01226114670749	2.03119157677728
C	-6.83668300829563	0.16786666426370	1.43894517106804
O	-5.98837982336013	0.90116003872100	1.88364028488591
O	-8.69797586181787	-2.73581273211306	1.63121336240262
H	-6.42039015002514	-1.86308693197937	-2.79567115649829
H	-8.41410562466151	3.05399013864637	-0.13906056536312
C	-6.73178013994063	-1.48699208268650	3.31145415265269
C	-5.52464047052448	-2.39735464487744	3.12990447877698
H	-7.83644719476171	5.43306491884023	-0.16651921359353
H	-5.59916096793162	6.17172662409348	-0.94051394618113
N	-8.00756619993872	-3.18257602696699	-1.24663300381860
C	-6.50796608571972	2.39188774331092	-0.90217009028953
C	-6.45971892543015	0.15208900644152	-1.93200170605299
C	-6.84198055782383	-1.25284728434331	-2.00386006477232
C	-9.19481577408392	-3.84024995139057	-1.05801159911859
O	-9.21230924993172	-5.04630347793897	-0.88099100860272
C	-7.42728173039113	3.35188139513683	-0.47510955199433
C	-7.10480385747331	4.70247288537337	-0.49485619791712
C	-5.85315854370215	5.11734937804175	-0.93131538287524
C	-4.92510018677151	4.16907083986286	-1.34668229011659
C	-5.24931865433869	2.82065949766335	-1.33007805478279
C	-10.45896306105135	-3.04739920752047	-1.15863904573989
C	-10.65269024389450	-2.13257777625659	-2.18908424717849
C	-11.86078326442330	-1.45499906348573	-2.29548759058183
C	-12.86859972365132	-1.68305922337948	-1.36684558189924
C	-12.67477209644155	-2.60101896708103	-0.33858040302360
C	-11.47582165322417	-3.29111177391468	-0.24061712092707
H	-3.93911872738384	4.48030831070491	-1.67509401579052
H	-4.51053636098745	2.08585326753592	-1.62989729525758
H	-8.45079289003445	1.08976472653197	0.47860860760040

H	-9.42740113657311	-0.88591689189039	-0.22409038765642
H	-6.47395779343293	-0.60458589090152	3.89784533048571
H	-7.54916615649169	-2.01265052971757	3.80648153170604
H	-5.16590727484642	-2.73773523080603	4.10386447147496
H	-5.78986442081422	-3.27484442209392	2.53563769401732
H	-4.71267287086960	-1.86421344181059	2.63028664532572
H	-9.86377959259941	-1.95255886733934	-2.91107354037086
H	-12.01350516390605	-0.74687139260752	-3.10235888265447
O	-9.75147133253459	1.33982033374907	-2.18487155028774
C	-9.06126906916076	1.66903964371956	-3.05716976917732
O	-8.38488924964208	1.99959488514012	-3.93701848814664

The electronic energy, computed at the DLPNO-CCSD(T)/def2-TZVPP/CPCM level of theory, the thermochemical corrections obtained at the ωB97X-D4/def2-TZVP/CPCM level, the resulting total Gibbs free energy, and the first 15 vibrational frequencies of the **intermediate 2 with CO2** are:

Electronic energy: -1409.952360949358 Eh
Thermochemical correction energy: 0.29186899 Eh
Total Gibbs free energy: -1409.660491959358069 Eh

Vibrational Frequencies:

1: 0.00 cm**-1
2: 0.00 cm**-1
3: 0.00 cm**-1
4: 0.00 cm**-1
5: 0.00 cm**-1
6: 9.62 cm**-1
7: 18.57 cm**-1
8: 25.46 cm**-1
9: 29.79 cm**-1
10: 37.06 cm**-1
11: 40.69 cm**-1
12: 44.01 cm**-1
13: 52.09 cm**-1
14: 60.19 cm**-1
15: 62.32 cm**-1

Optimized coordinates for **intermediate 2 with b**:

C	-3.39314335637289	-0.52380401807210	-1.81273572210538
C	-2.56922233950651	0.42261480450954	-2.23959429167329
C	-2.25149834364210	1.32691606983556	-1.08201642413682
N	-2.97423282926703	0.85341480201404	0.00267778822674
C	-3.66474226211625	-0.29597750468319	-0.35178671439445
C	-6.84228637310066	0.95468968167857	-0.96745318977282
C	-7.62862118906667	0.35182242176543	0.17254362078778
C	-8.23088706335581	-1.05263861243186	-0.03331640609643
C	-7.65593880055523	-1.82820020690880	-1.19922711624960
C	-7.96312681937783	-1.76563617097788	1.29016276499262
N	-7.15571692820171	-0.96745309889800	2.06796757956570
C	-6.84845073177241	0.24562217643233	1.48392470325429
O	-6.14259948891117	1.08371910112933	1.98727737594378

O	-8.38882519388893	-2.84398907065271	1.62945386513783
O	-4.32809570722687	-0.98654658586475	0.38339219257465
O	-1.50083822655296	2.27203440472969	-1.07195350665060
C	-6.70112337442276	-1.35976441311359	3.39740871606320
C	-5.45079699446485	-2.22684623444017	3.34686252302355
C	-2.87766046225221	1.39778645537943	1.35454190733318
C	-3.42886920694280	2.81251956578546	1.46655045047305
N	-7.87085523847372	-3.22264081927227	-1.27169391820029
C	-6.44345156289061	2.38155725512747	-0.89372718914825
C	-6.54897237083345	0.19127226453921	-2.02582827672294
C	-6.89559745964713	-1.22022566333920	-2.10987747712088
C	-9.01549906333314	-3.92824176813443	-1.01330818144947
O	-8.97889015523636	-5.13340921094452	-0.83473195017483
C	-7.12144518476243	3.29748233426097	-0.08699807317442
C	-6.73789345368691	4.63117113166218	-0.04030879168637
C	-5.66184425968955	5.08046099027840	-0.79427045159925
C	-4.97173121267482	4.17950911567812	-1.59637546808623
C	-5.35768096005762	2.84963423553369	-1.64155473259154
C	-10.31058227645772	-3.17946966017933	-1.04146108222652
C	-10.60028832874509	-2.29117371795123	-2.07302024876968
C	-11.83052776206610	-1.64658950211553	-2.10676735591010
C	-12.76452585874324	-1.88093735935703	-1.10465123105776
C	-12.47511155177525	-2.77342128932959	-0.07676893631347
C	-11.25383514690252	-3.43109243399103	-0.05059430727492
H	-3.82431440254234	-1.35536628279722	-2.35117554508475
H	-2.14694827250412	0.57754695703238	-3.22247964659311
H	-8.45534966414120	1.02704886562854	0.41019466416292
H	-9.31570115071326	-0.99999324120112	-0.14226536691102
H	-6.51748264182950	-0.43751126673447	3.94933905137543
H	-7.52693597281042	-1.89345695501257	3.87049394024394
H	-5.15479517827569	-2.50130923191096	4.36217322530468
H	-5.64011174063913	-3.14335379101585	2.78393437236264
H	-4.62929813096416	-1.69063902119866	2.87047589943720
H	-3.43763603290666	0.71501760500742	1.99324590325475
H	-1.82632791272853	1.36714071597310	1.65361367166124
H	-3.33964555917295	3.14664475505773	2.50341470442348
H	-2.87022132768567	3.50239780665959	0.83317797348882
H	-4.48101588071011	2.83939188522732	1.18686261699391
H	-7.05017561741770	-3.80320021330676	-1.36718784085379
H	-6.03847722792934	0.62129306634751	-2.87898146492226
H	-6.52767522638880	-1.79368061991681	-2.95437490365175
H	-7.95811643780327	2.98015230847894	0.52259354748789
H	-7.28288455616418	5.32124312513740	0.59501882235209
H	-5.35740123271031	6.12043272236380	-0.74992454710000
H	-4.11634714327915	4.50873061203229	-2.17661979983894
H	-4.79003240834077	2.16181554859909	-2.25580039917032
H	-9.86891707295951	-2.10724909245780	-2.85218216221864
H	-12.06006982299195	-0.96255814663119	-2.91660655535462
H	-13.72243729796512	-1.37217038404212	-1.12684240427922
H	-13.20544023334587	-2.95891340719330	0.70333056643171
H	-11.01537860198711	-4.13219219756133	0.74038437704447

The electronic energy, computed at the DLPNO-CCSD(T)/def2-TZVPP/CPCM level of theory, the thermochemical corrections obtained at the ωB97X-D4/def2-TZVP/CPCM level, the resulting total Gibbs free energy, and the first 15 vibrational frequencies of the **intermediate 2 with b** are:

Electronic energy: -1658.989600781479 Eh
Thermochemical correction energy: 0.39520143 Eh
Total Gibbs free energy: -1658.594399351479069 Eh

Vibrational Frequencies:

1: 0.00 cm**-1
2: 0.00 cm**-1
3: 0.00 cm**-1
4: 0.00 cm**-1
5: 0.00 cm**-1
6: 12.61 cm**-1
7: 20.29 cm**-1
8: 26.52 cm**-1
9: 35.35 cm**-1
10: 37.07 cm**-1
11: 41.20 cm**-1
12: 48.18 cm**-1
13: 52.71 cm**-1
14: 61.41 cm**-1
15: 66.32 cm**-1

Optimized coordinates for transition state 3 (**TS3**):

C	-4.92985191727197	-0.87441508451649	-1.27037220304864
C	-4.78832169231851	0.48550554864974	-1.48459565731200
C	-3.94093491784445	1.02881651077703	-0.37589151631048
N	-3.74315740373387	-0.00184356396702	0.53898924901934
C	-4.26681321949606	-1.19128344176229	0.03536135543657
C	-6.84852329712616	1.37371429785703	-1.33086779606176
C	-7.37219681600673	0.74498186792205	-0.05152100704547
C	-7.74593404363416	-0.73765338823542	-0.16384177695385
C	-7.17481703351692	-1.39896408223627	-1.39617602237754
C	-7.36676115603270	-1.33943375913967	1.17226754441047
N	-6.72093664672846	-0.37760058500968	1.91999417128564
C	-6.58741860619930	0.83701533122998	1.25857299319119
O	-6.03067070744223	1.80157361260708	1.71683871883222
O	-7.63601248485246	-2.44899664894582	1.56354977144015
O	-4.14089659337328	-2.28184400548555	0.54232544996719
O	-3.42423986489575	2.11725449039099	-0.31406630001612
C	-6.42593189692184	-0.56679026541390	3.34150435119594
C	-5.39259806225983	-1.64734977607773	3.62422451393094
C	-2.72449578542793	0.06681216834631	1.58506137621744
C	-3.02456030367669	1.06263090926784	2.69543080506072
N	-7.08453332665000	-2.82153324708891	-1.43514533845518
C	-6.60661864148621	2.84185209675943	-1.37357044578947
C	-7.20230674587943	0.72014889411091	-2.51153665154273
C	-7.30460032124989	-0.66701331079260	-2.55883665664767
C	-8.06392064095295	-3.71632408338768	-1.07418193651545
O	-7.78386823211251	-4.87128590139442	-0.81201538239712
C	-7.08606447799732	3.70094443837855	-0.38498589091040
C	-6.89294558283163	5.07268483796612	-0.48033154995805
C	-6.20957760307605	5.61272793682051	-1.56079830374745
C	-5.72307681984970	4.76755284244624	-2.55135435420672
C	-5.92115774336234	3.39961959349510	-2.45681594512836
C	-9.48267961478490	-3.23671052679171	-1.07842154141931
C	-10.01882310507806	-2.56153665489623	-2.17005595971731
C	-11.35217553543442	-2.17004134417219	-2.15717640429114

C	-12.14601423350548	-2.44444793366413	-1.05044387800334
C	-11.61131531660283	-3.12877349169888	0.03704461483017
C	-10.28487476994444	-3.53396308822292	0.01971432865479
H	-4.86741365221082	-1.63030440104071	-2.03698416986026
H	-4.66762330449181	0.95324862128248	-2.44634608525066
H	-8.29251186976658	1.29364379348259	0.17891520551840
H	-8.83146642841799	-0.85314214668575	-0.23278262528774
H	-6.10007187694319	0.40490291934278	3.71166369170787
H	-7.37112039145500	-0.81703877070417	3.83092553308969
H	-5.21994976358048	-1.69241640825547	4.70229273263239
H	-5.74182768276286	-2.62223051673146	3.28666330286774
H	-4.44524029088561	-1.43561426030459	3.13294035172726
H	-2.62290936511275	-0.94642638255353	1.97467434092577
H	-1.77954504433577	0.33049962984971	1.10020178559532
H	-2.19308870864926	1.05584352104880	3.40490964155328
H	-3.13351543697375	2.07073227077793	2.29701437528602
H	-3.93658550399400	0.80733151789453	3.23180641875628
H	-6.15814119693958	-3.22023984031394	-1.36196988410791
H	-7.18146061994694	1.27594562679871	-3.44159369956204
H	-7.29161710473274	-1.18064334761009	-3.51396192583558
H	-7.61549793712862	3.31265701970853	0.47376691184274
H	-7.27766281148903	5.72045876012299	0.29992319853409
H	-6.05230481535931	6.68362206382843	-1.62957318583693
H	-5.17980041463946	5.17393495123041	-3.39753269806627
H	-5.52139680842990	2.76066552250814	-3.23562449022705
H	-9.40434970831528	-2.35797495867851	-3.03840313508899
H	-11.77138355308158	-1.65456773118904	-3.01450452103570
H	-13.18518133321119	-2.13321327425267	-1.03788117848252
H	-12.23208658306232	-3.35040545329919	0.89838143037277
H	-9.85565050452536	-4.06914639038234	0.85788374261486

The electronic energy, computed at the DLPNO-CCSD(T)/def2-TZVPP/CPCM level of theory, the thermochemical corrections obtained at the ωB97X-D4/def2-TZVP/CPCM level, the resulting total Gibbs free energy, and the first 15 vibrational frequencies of the transition state 3 (**TS3**) are:

Electronic energy: -1658.932655827717 Eh
Thermochemical correction energy: 0.40079695 Eh
Total Gibbs free energy: -1658.531858877717013 Eh

Vibrational Frequencies:

1: 0.00 cm**-1
2: 0.00 cm**-1
3: 0.00 cm**-1
4: 0.00 cm**-1
5: 0.00 cm**-1
6: -574.08 cm**-1 ***imaginary mode***
7: 20.36 cm**-1
8: 32.39 cm**-1
9: 43.52 cm**-1
10: 53.47 cm**-1
11: 58.74 cm**-1
12: 65.38 cm**-1
13: 74.65 cm**-1
14: 80.01 cm**-1

15: 83.12 cm**-1

Optimized coordinates for **product**:

C	-5.43683605005849	-0.89664425699301	-1.11034972514667
C	-5.29059853790362	0.61540387761936	-1.28646999431064
C	-4.14191454269436	0.99793826750202	-0.37152613461430
N	-3.79702981551512	-0.11265966235516	0.39033067617416
C	-4.53388486589403	-1.23623751763634	0.06605786686774
C	-6.66795997206575	1.29823505437015	-1.05868172503754
C	-7.28779982066076	0.78924455245998	0.27125243836386
C	-7.63450369922446	-0.70188711635411	0.19192830946486
C	-6.92149121311666	-1.33829596562664	-1.03132350647236
C	-7.28640745835412	-1.26503058095480	1.54840354087961
N	-6.59112322636891	-0.30286129721027	2.25448945281573
C	-6.48088676746254	0.90102314049390	1.56718932529809
O	-5.89152906375715	1.86568632852533	1.98765555307832
O	-7.60282360838764	-2.34688499255214	1.98241958452825
O	-4.42426310390336	-2.32041879265649	0.59206096927638
O	-3.55483200971832	2.04927922686953	-0.34363705138074
C	-6.19042084919852	-0.48268859747353	3.64995575213013
C	-5.19208370279865	-1.61366107332507	3.85792561258817
C	-2.62779962410771	-0.12985227647949	1.27115428302028
C	-2.72079118789316	0.84253530179444	2.43884182173053
N	-6.94164082623353	-2.79031201419796	-1.04646484738493
C	-6.59361319688927	2.80922326113712	-1.21250562588906
C	-7.51105723744184	0.68063691733814	-2.17575218191793
C	-7.58939528723857	-0.64372853000042	-2.20278592741875
C	-7.99691097805673	-3.65350165068287	-1.07160161054729
O	-7.82396586284625	-4.84620832548691	-0.88022953152390
C	-7.01509685753397	3.69976334028330	-0.23286654755766
C	-6.95585886273143	5.07376350649117	-0.44711202352628
C	-6.47326864875645	5.57878641684044	-1.64436501435950
C	-6.05247136825552	4.69650995919228	-2.63387052202924
C	-6.11616124222697	3.32942400067388	-2.41754723879720
C	-9.37647978673637	-3.13640813282027	-1.37167813096022
C	-9.83479694384501	-3.09921560718871	-2.68428769553934
C	-11.13914839827902	-2.70665608807015	-2.95341701768617
C	-11.99086494135791	-2.35962675724595	-1.91073931496384
C	-11.53840442287080	-2.41801044102563	-0.59797615342573
C	-10.23396910903193	-2.81188013283870	-0.32575765497940
H	-5.00147243114536	-1.42552863596482	-1.96262018935352
H	-4.96118227290944	0.86601625770497	-2.29356725417588
H	-8.20079011320842	1.36010972896939	0.45793864454006
H	-8.70180507718386	-0.85805410088915	0.05442129527699
H	-5.78049259553140	0.47508707908278	3.97008603060205
H	-7.09952699079483	-0.67289785700181	4.22676140770859
H	-4.91053155734016	-1.64384685670547	4.91315763362501
H	-5.62503297085690	-2.57542478051320	3.58600237642649
H	-4.28990378924477	-1.46590148583898	3.26579879744988
H	-2.52772258761379	-1.15879333659355	1.61709509992870
H	-1.75706392438055	0.10717702937199	0.65399784157624
H	-1.82120729689966	0.73980229031522	3.05036779525634
H	-2.78858469707224	1.87166584045838	2.08907818491625
H	-3.58722811899959	0.63629374086575	3.06527617435560
H	-6.10299672022626	-3.22793729239128	-0.68504717427814
H	-7.95860926235322	1.32085486417585	-2.92596883238908
H	-8.10423699458090	-1.20497100150958	-2.96868349289632
H	-7.37623582853088	3.34240073696887	0.72096657840294
H	-7.28633882742200	5.74867306275602	0.33524889294145

H	-6.42366242229253	6.64980067587891	-1.80822328901189
H	-5.67314067009962	5.07453322978182	-3.57725202692241
H	-5.78842261196526	2.66023050721823	-3.20642577038318
H	-9.17163817753573	-3.38203721402126	-3.49566482621735
H	-11.49287323483309	-2.67745400677013	-3.97833614377894
H	-13.01023360511639	-2.05436096361131	-2.12109860538102
H	-12.20500290920947	-2.16416651334928	0.21922954234742
H	-9.87949269506986	-2.87517887242577	0.69754973061122

The electronic energy, computed at the DLPNO-CCSD(T)/def2-TZVPP/CPCM level of theory, the thermochemical corrections obtained at the ωB97X-D4/def2-TZVP/CPCM level, the resulting total Gibbs free energy, and the first 15 vibrational frequencies of the **product** are:

Electronic energy: -1659.023505458945 Eh
Thermochemical correction energy: 0.40722557 Eh
Total Gibbs free energy: -1658.616279888944973 Eh

Vibrational Frequencies:

1: 0.00 cm**-1
2: 0.00 cm**-1
3: 0.00 cm**-1
4: 0.00 cm**-1
5: 0.00 cm**-1
6: 16.66 cm**-1
7: 27.70 cm**-1
8: 41.19 cm**-1
9: 53.78 cm**-1
10: 59.71 cm**-1
11: 67.59 cm**-1
12: 74.04 cm**-1
13: 77.38 cm**-1
14: 84.32 cm**-1
15: 88.53 cm**-1

NMR Spectra

Figure S7. ^{1}H NMR Spectrum (600 MHz, $CDCl_3$) for Pyrone **1a**

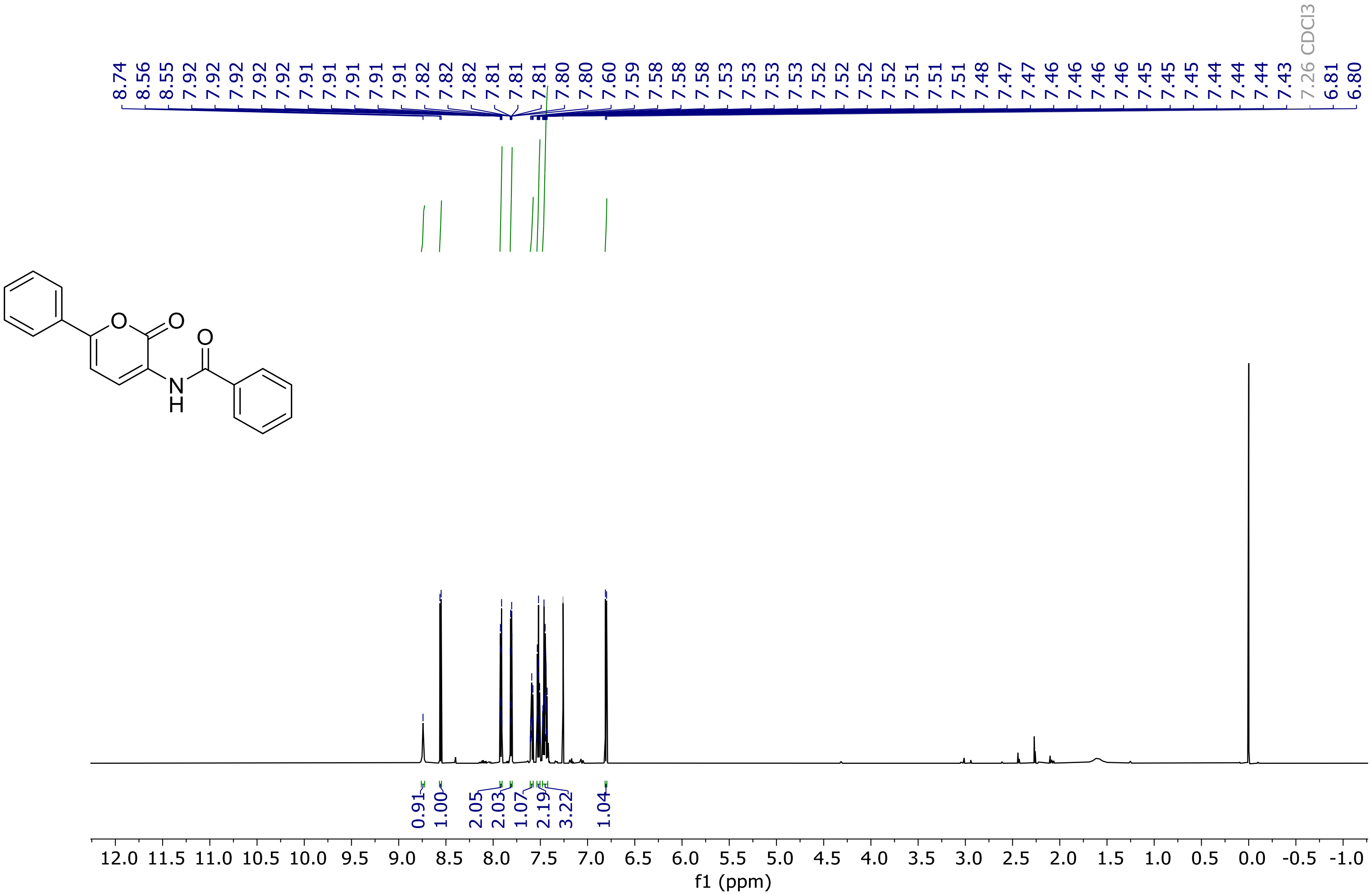

Figure S8. $^{13}C\{^{1}H\}$ NMR Spectrum (150 MHz, $CDCl_3$) for Pyrone **1a**

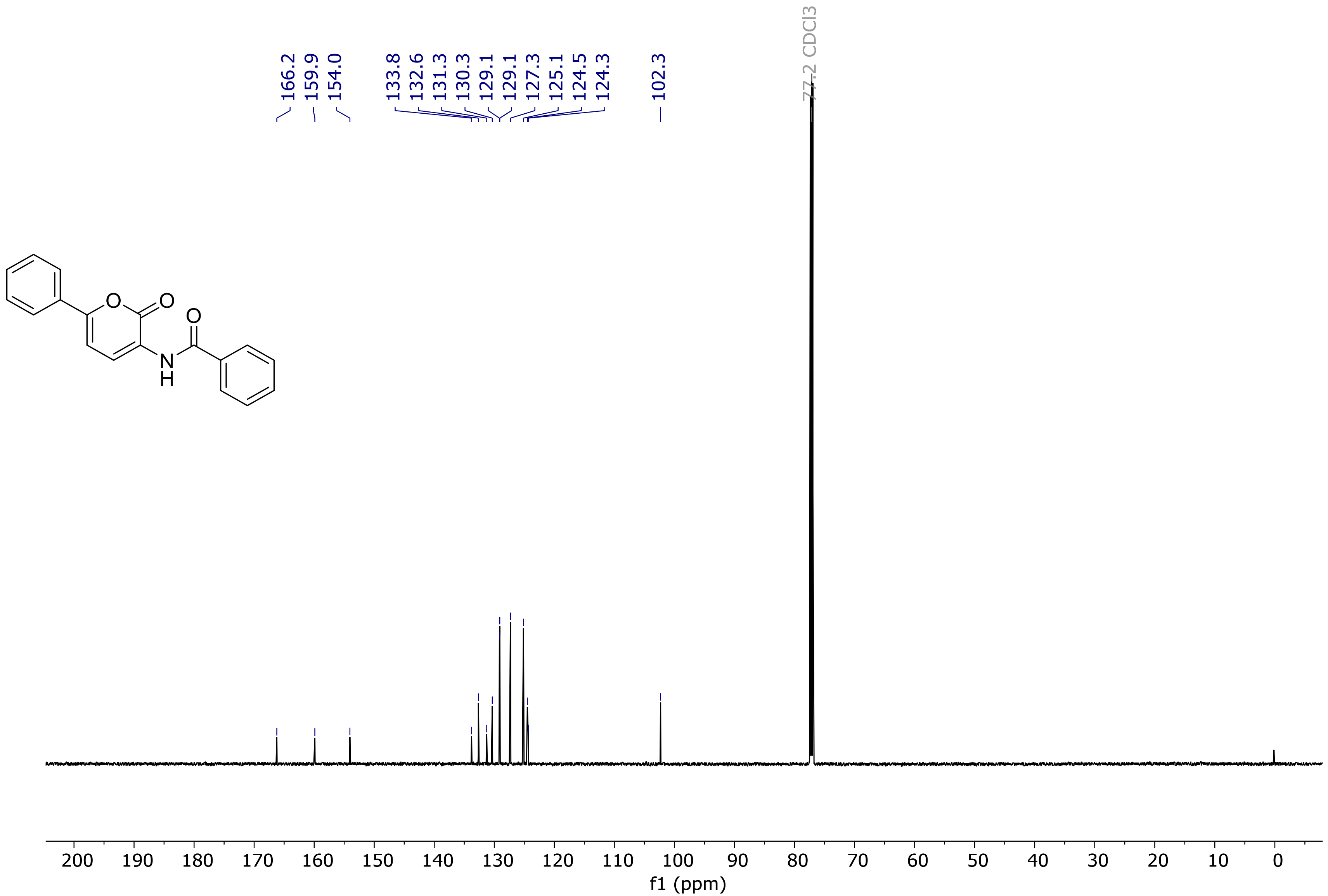

Figure S9. ^{1}H NMR Spectrum (500 MHz, $CDCl_3$) for Pyrone **1b**

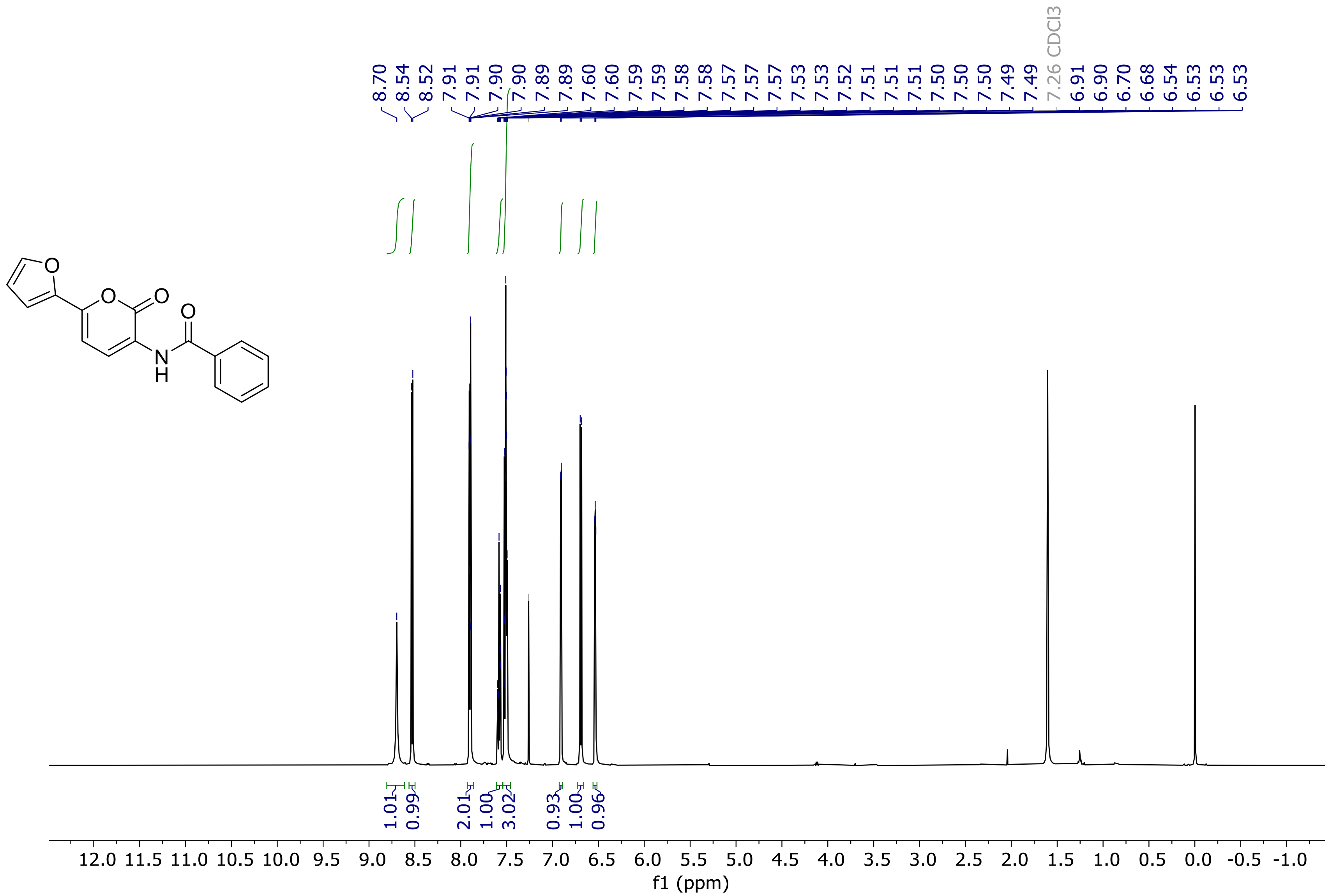

Figure S10. $^{13}C\{^{1}H\}$ NMR Spectrum (125 MHz, $CDCl_3$) for Pyrone **1b**

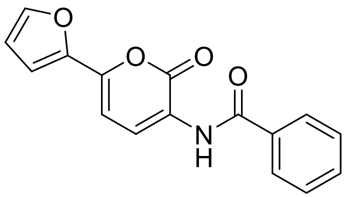

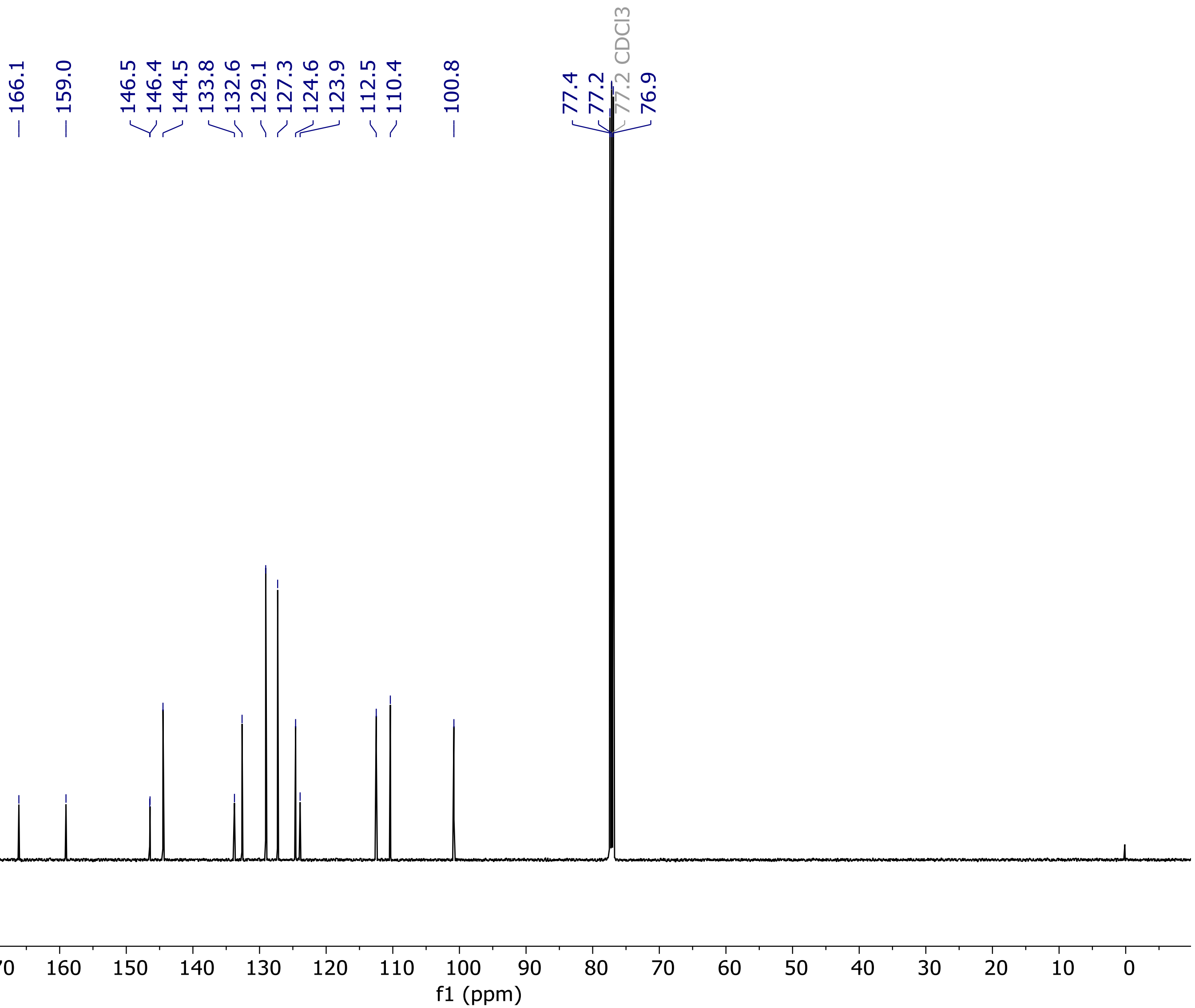

Figure S11. ^{1}H NMR Spectrum (500 MHz, $CDCl_3$) for Pyrone **1c**

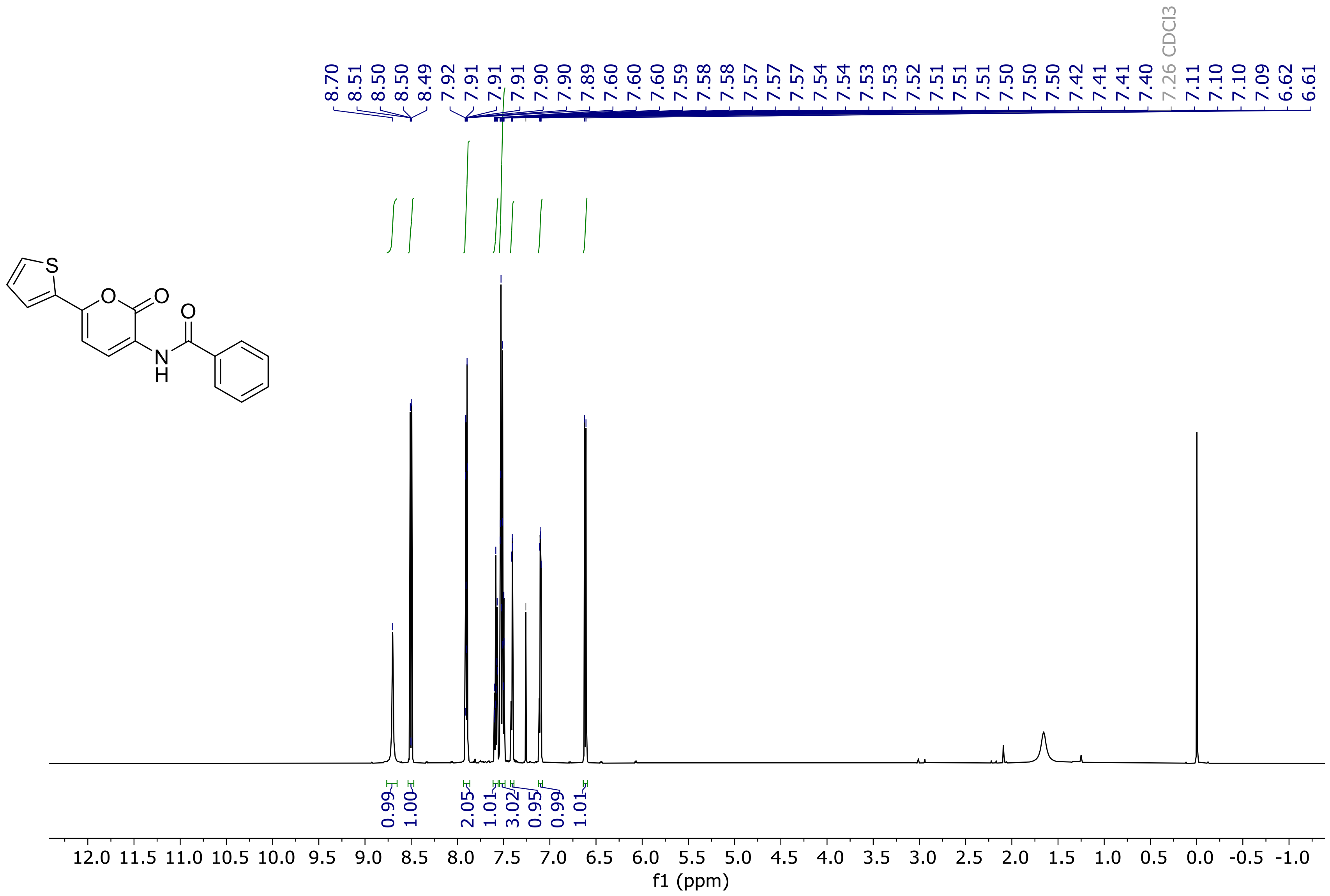

Figure S12. $^{13}C\{^{1}H\}$ NMR Spectrum (125 MHz, $CDCl_3$) for Pyrone **1c**

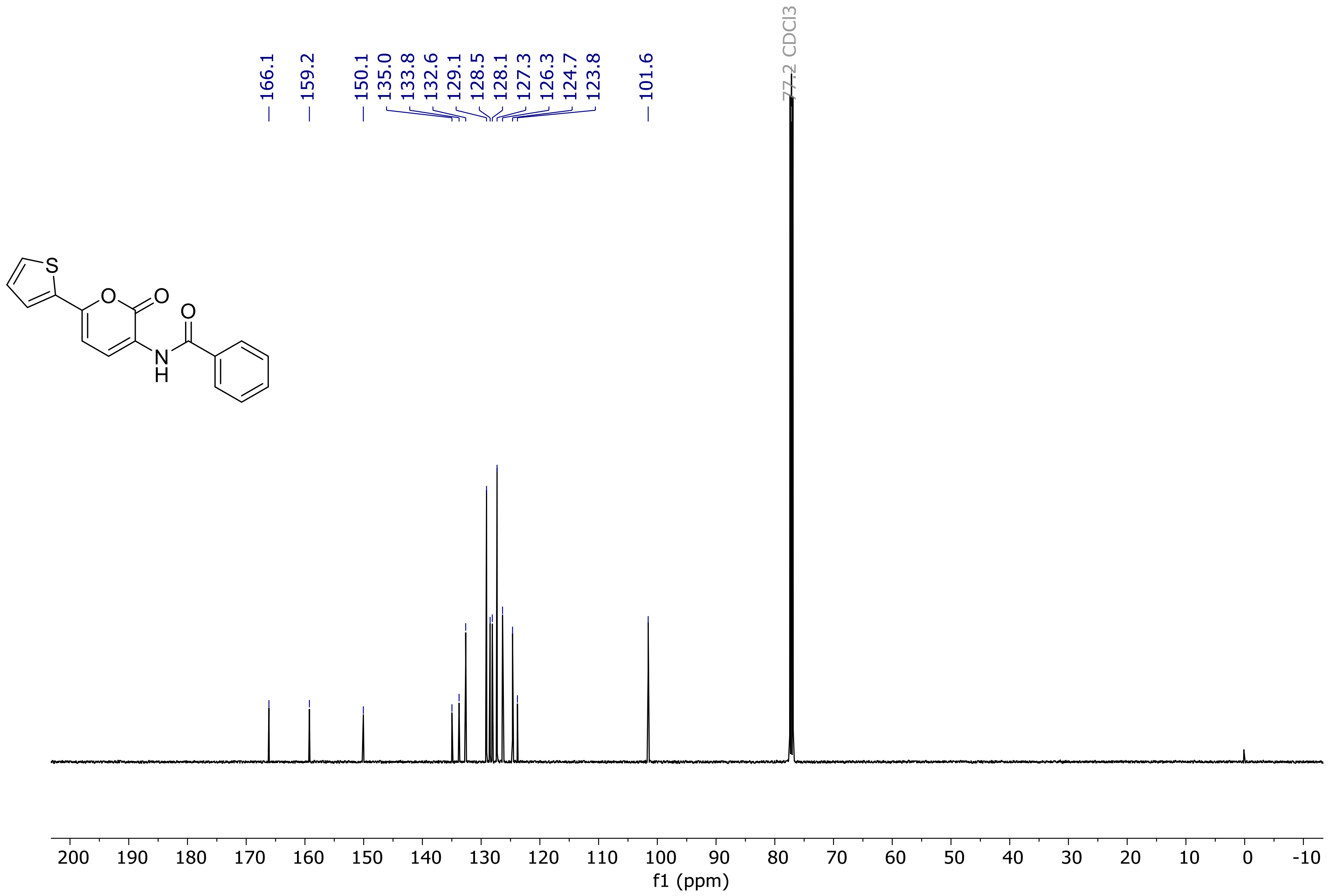

Figure S13. ^{1}H NMR Spectrum (500 MHz, $CDCl_3$) for Pyrone **1d**

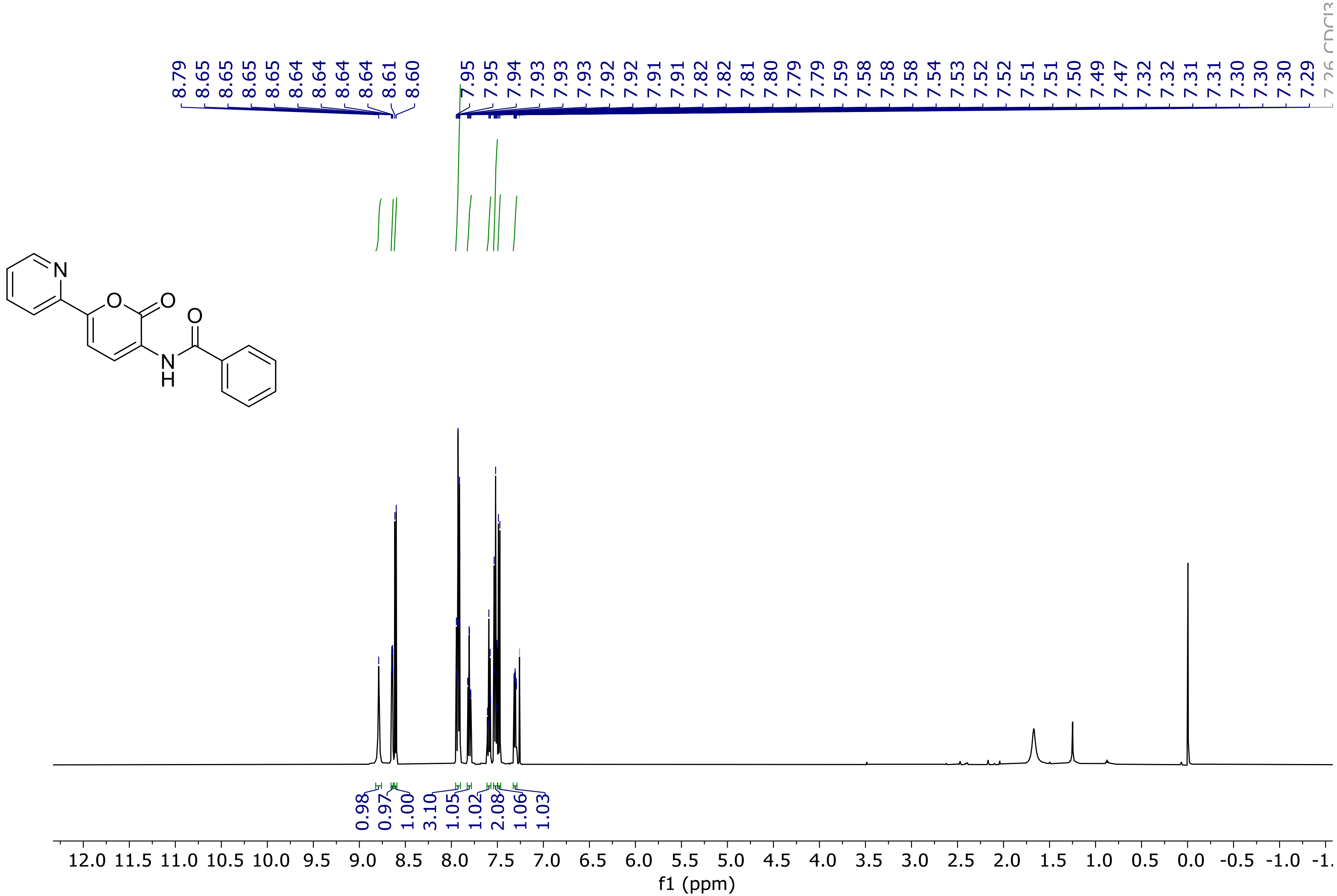

Figure S14. $^{13}C\{^{1}H\}$ NMR Spectrum (125 MHz, $CDCl_3$) for Pyrone **1d**

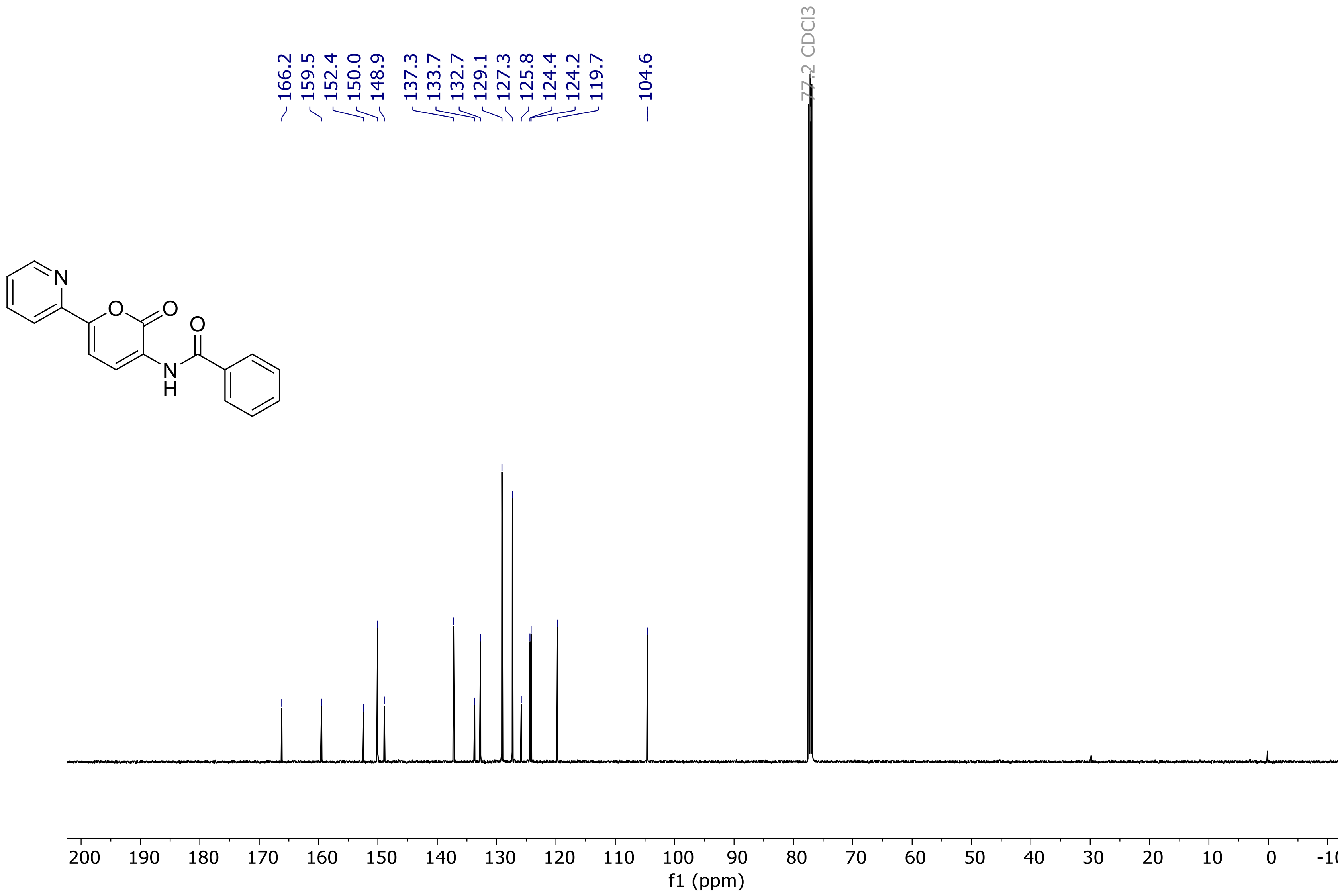

Figure S15. ^{1}H NMR Spectrum (600 MHz, $CDCl_3$) for Pyrone **1e**

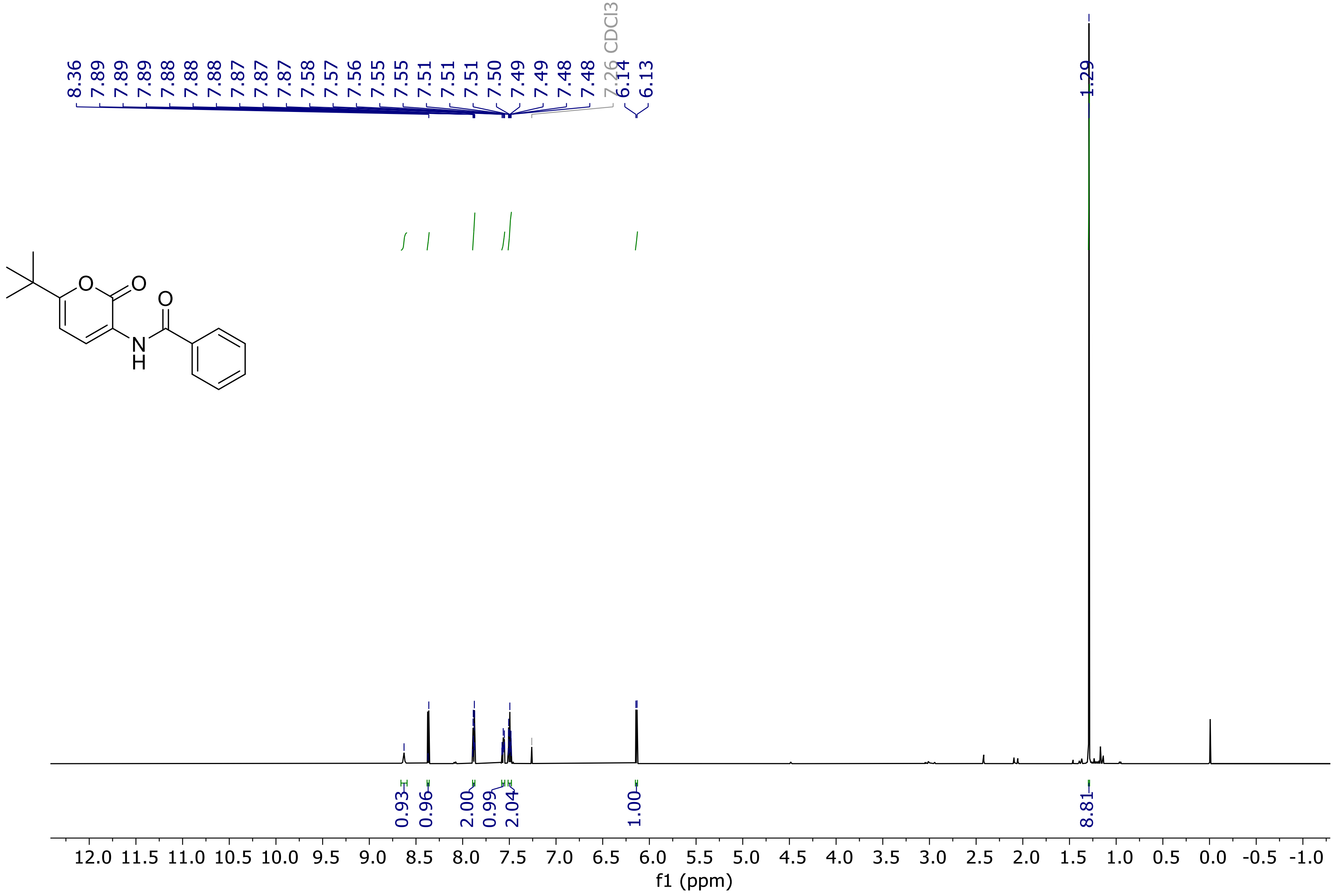

Figure S16. $^{13}C\{^{1}H\}$ NMR Spectrum (150 MHz, $CDCl_3$) for Pyrone **1e**

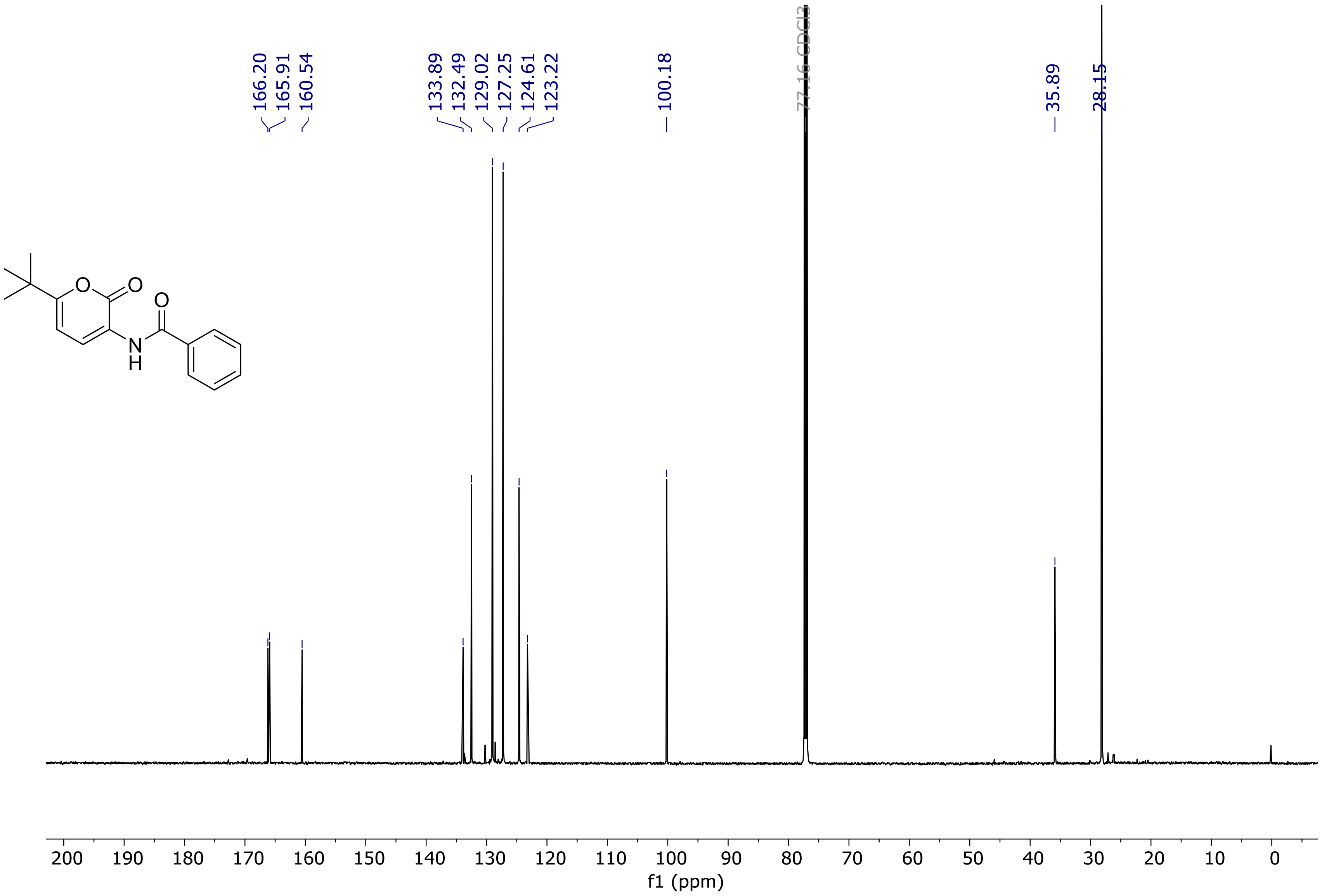

Figure S17. ^{1}H NMR Spectrum (600 MHz, $CDCl_3$) for Pyrone **1f**

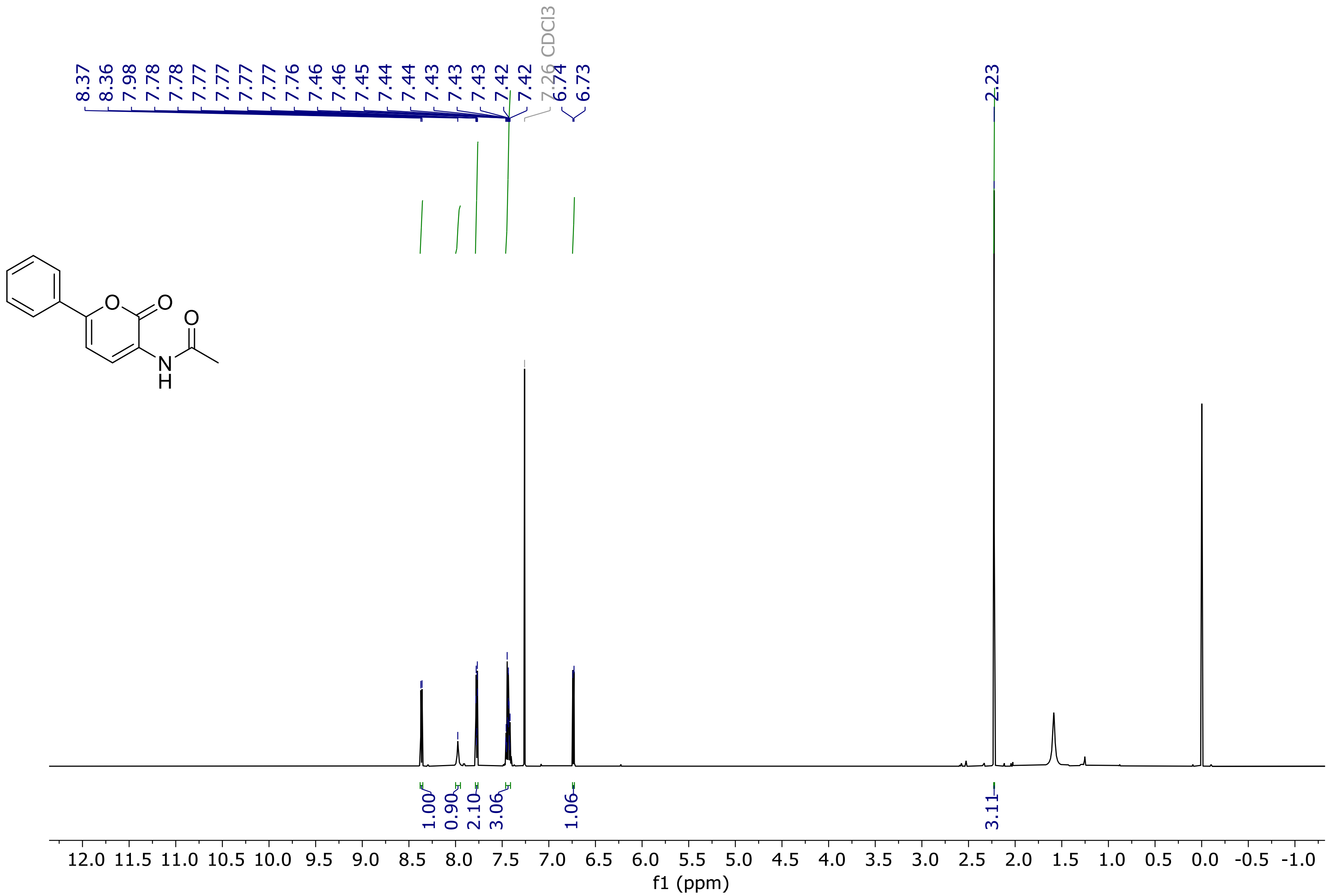

Figure S18. $^{13}C\{^{1}H\}$ NMR Spectrum (150 MHz, $CDCl_3$) for Pyrone **1f**

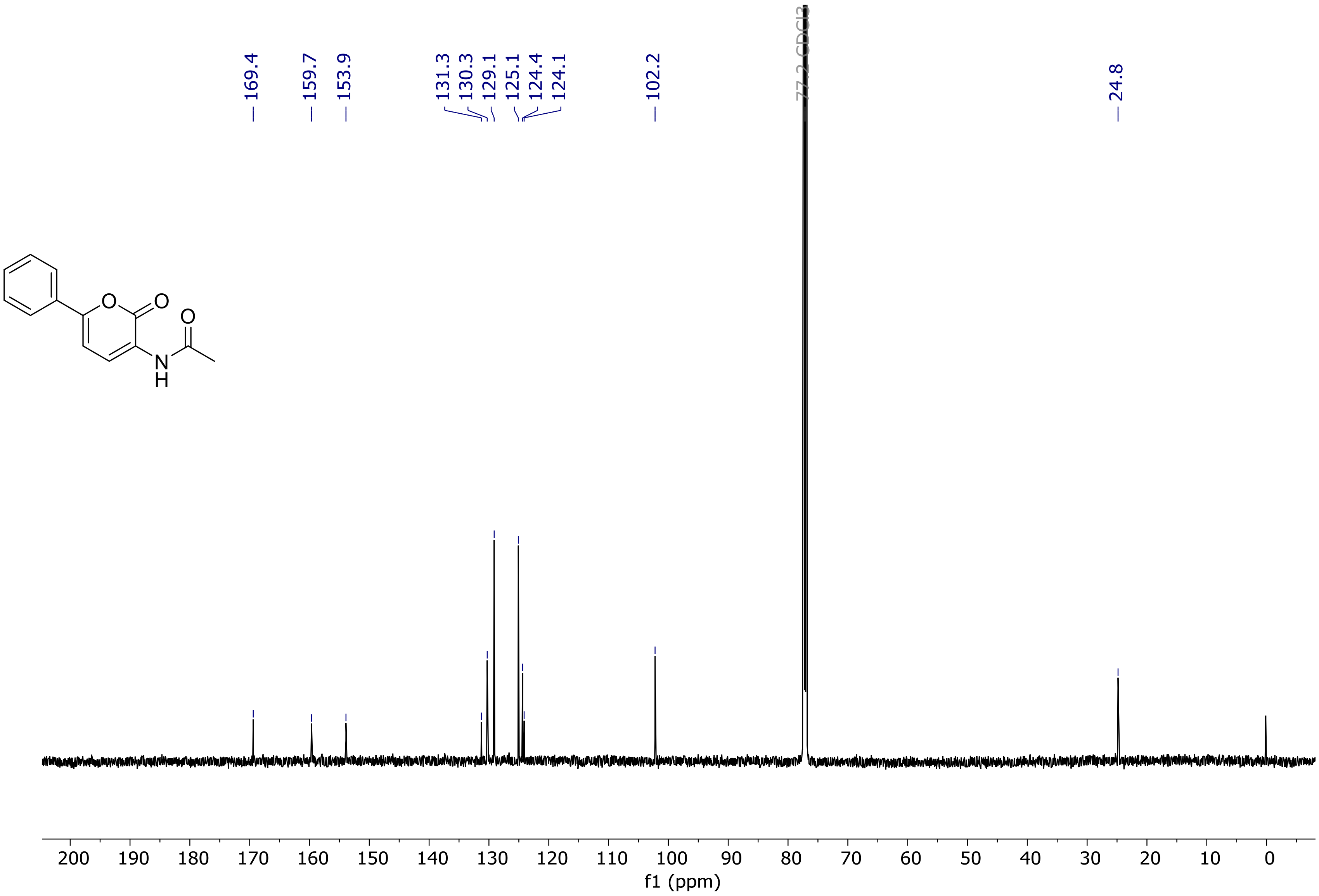

Figure S19. ^{1}H NMR Spectrum (500 MHz, $CDCl_3$) for Pyrone **1g**

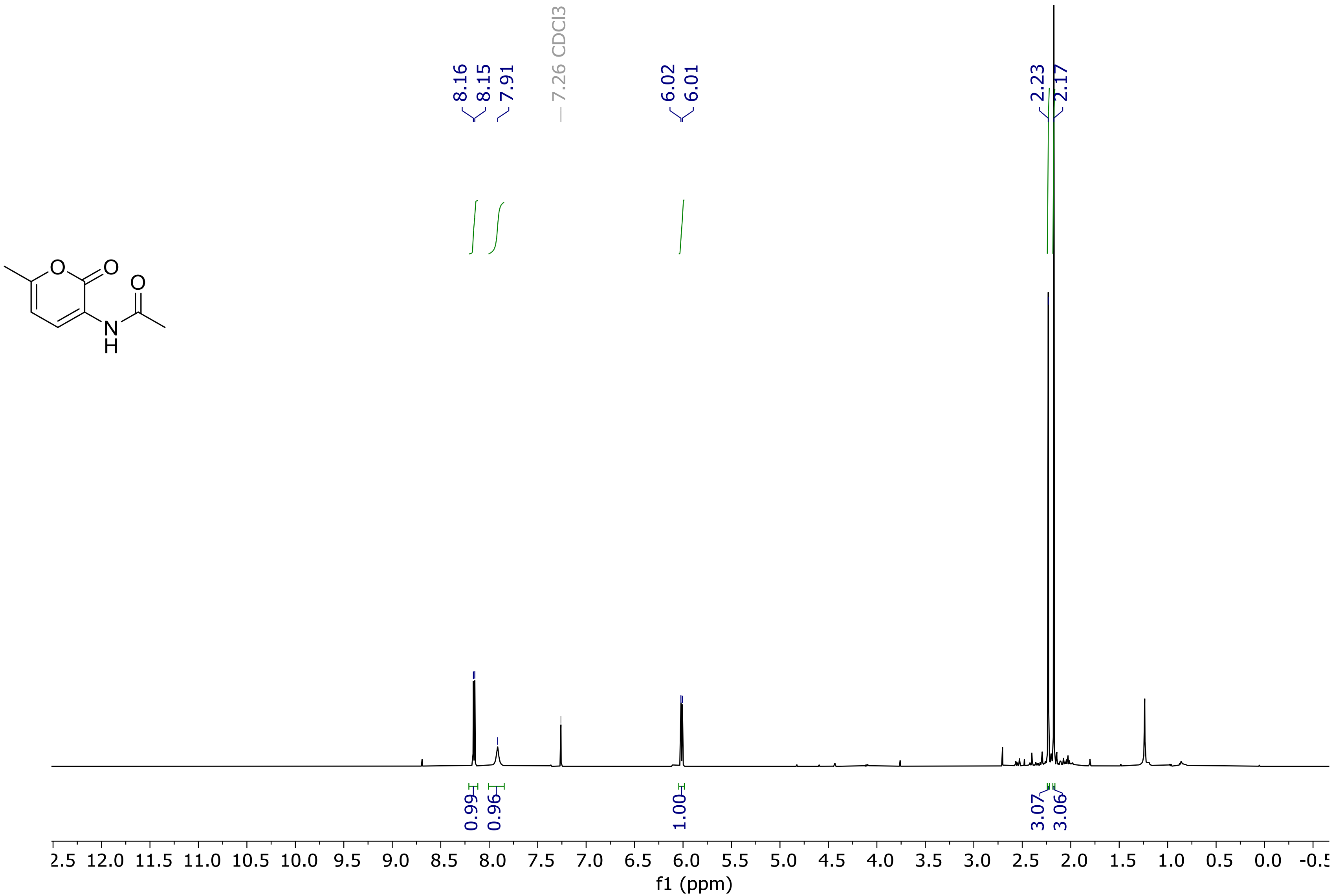

Figure S20. $^{13}C\{^1H\}$ NMR Spectrum (125 MHz, $CDCl_3$) for Pyrone **1g**

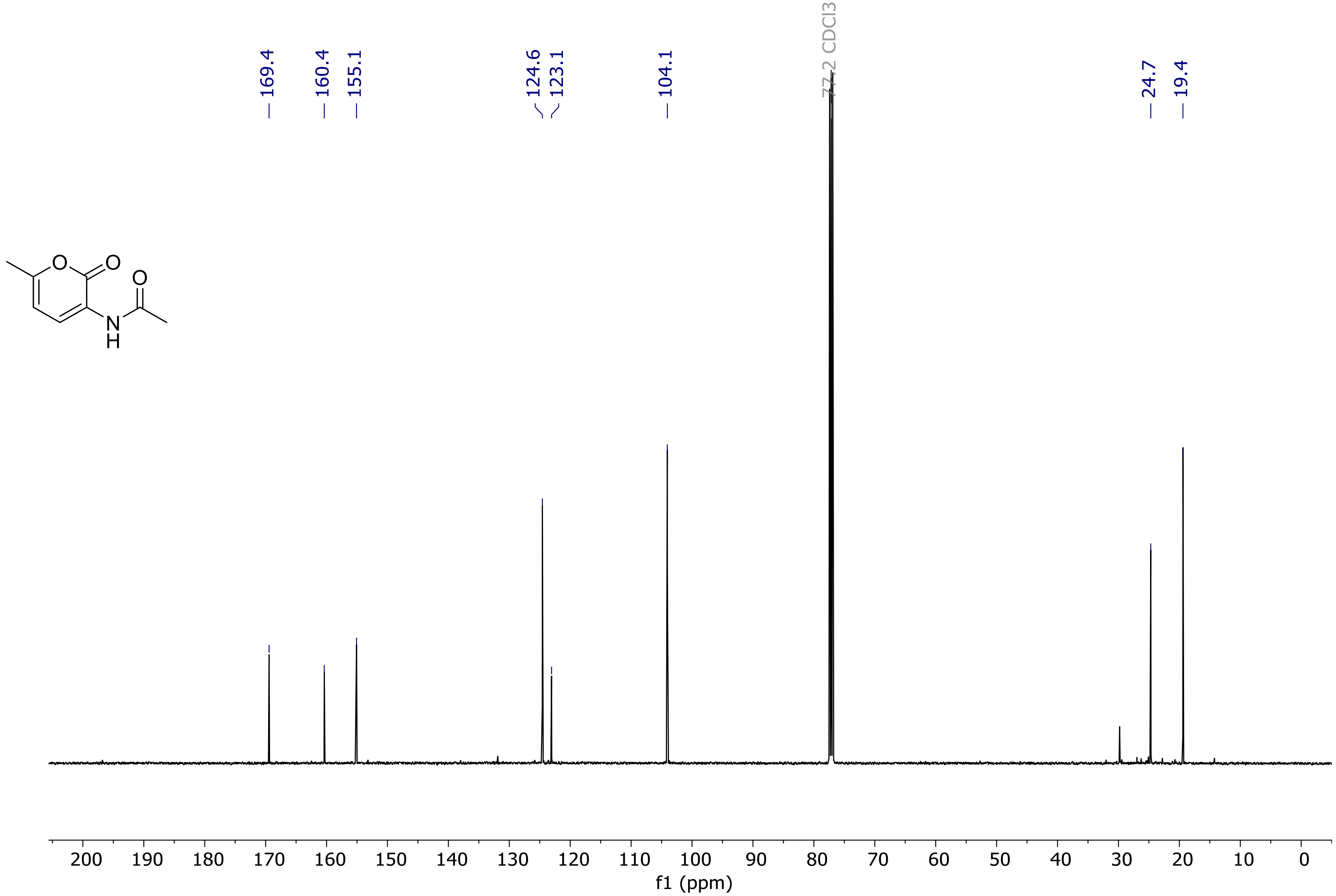

Figure S21. ^{1}H NMR Spectrum (500 MHz, $CDCl_3$) for Pyrone **1h**

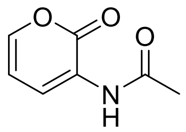

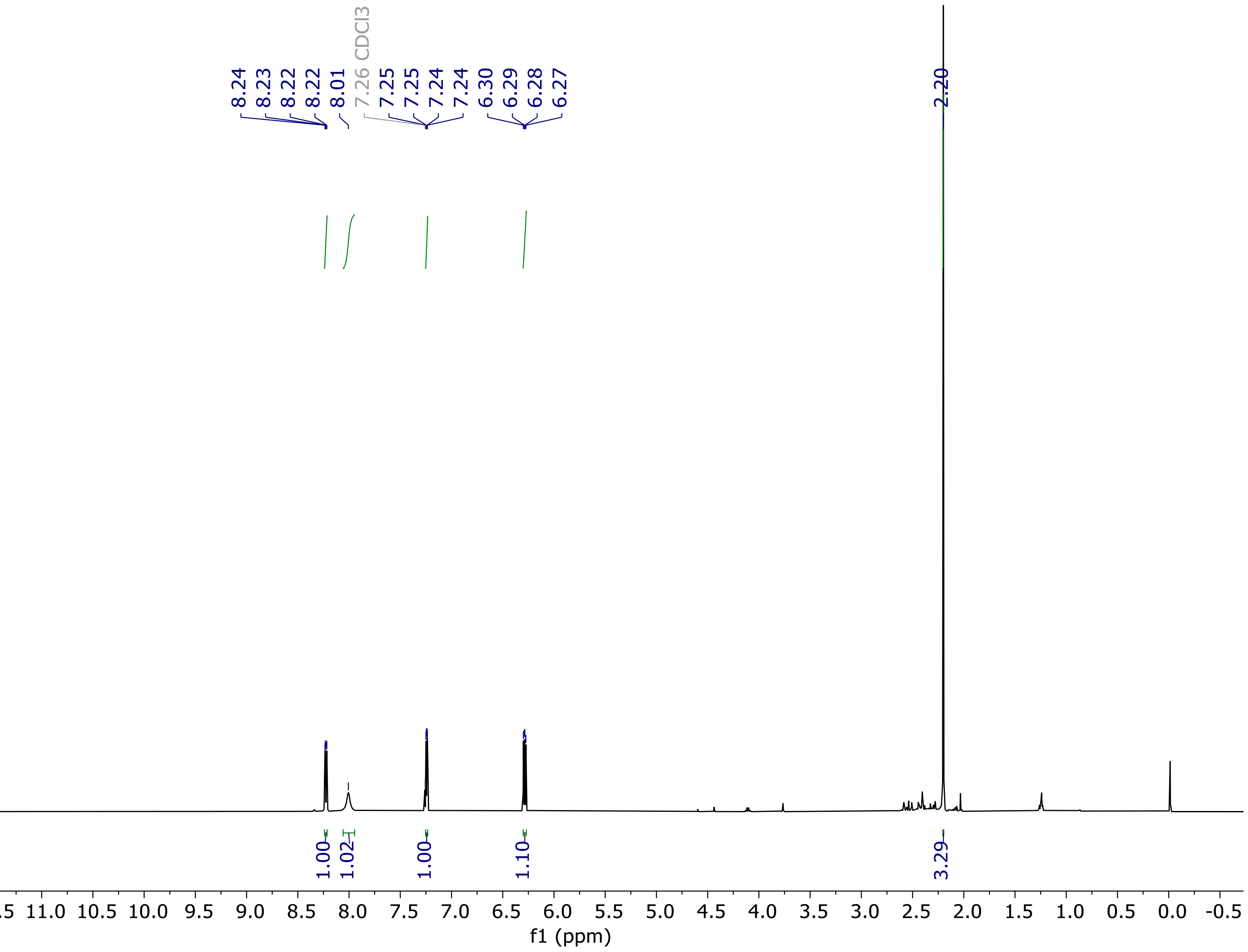

Figure S22. $^{13}C\{^{1}H\}$ NMR Spectrum (125 MHz, $CDCl_3$) for Pyrone **1h**

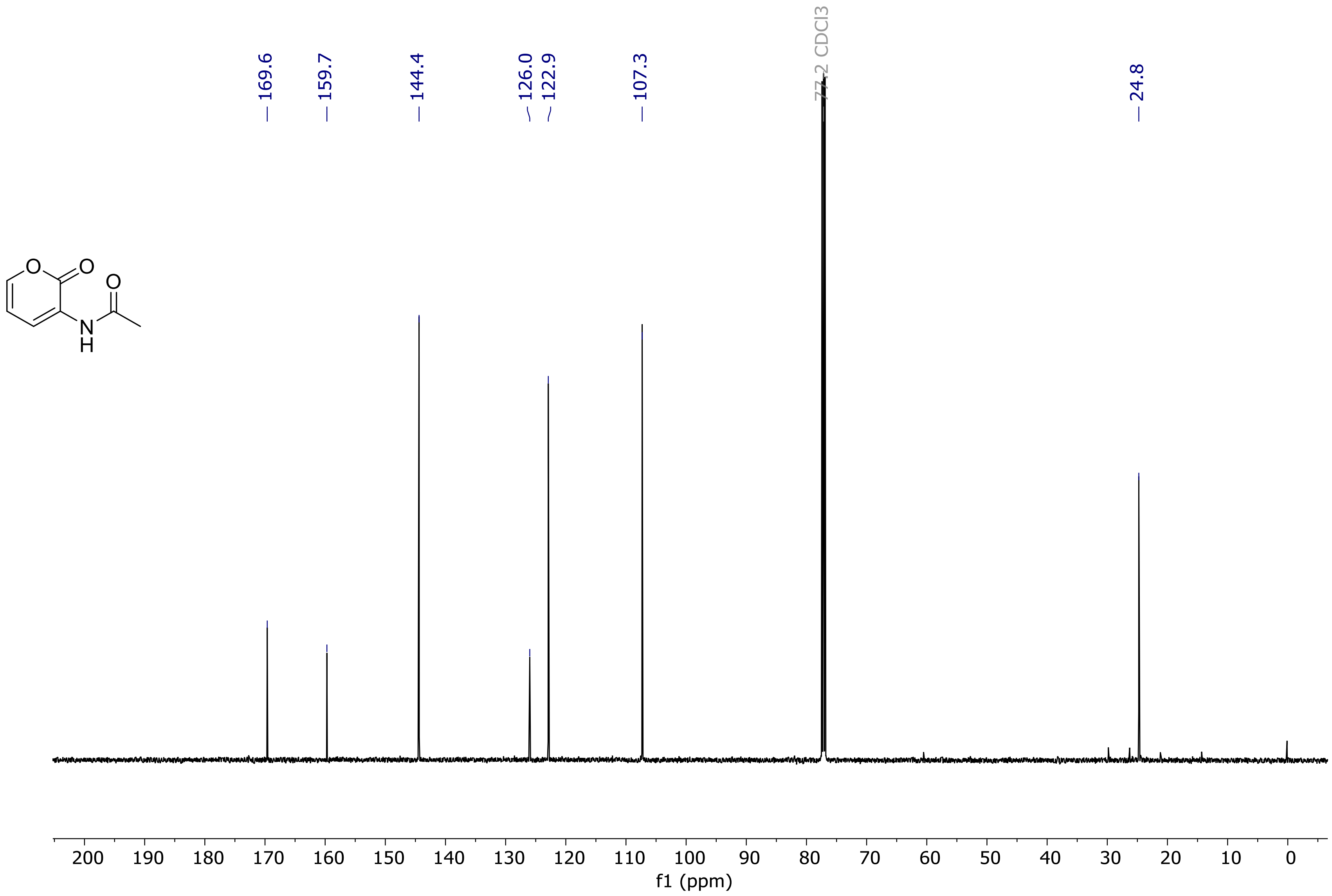

Figure S23. ^{1}H NMR Spectrum (500 MHz, $CDCl_3$) for Aromatic **2a**

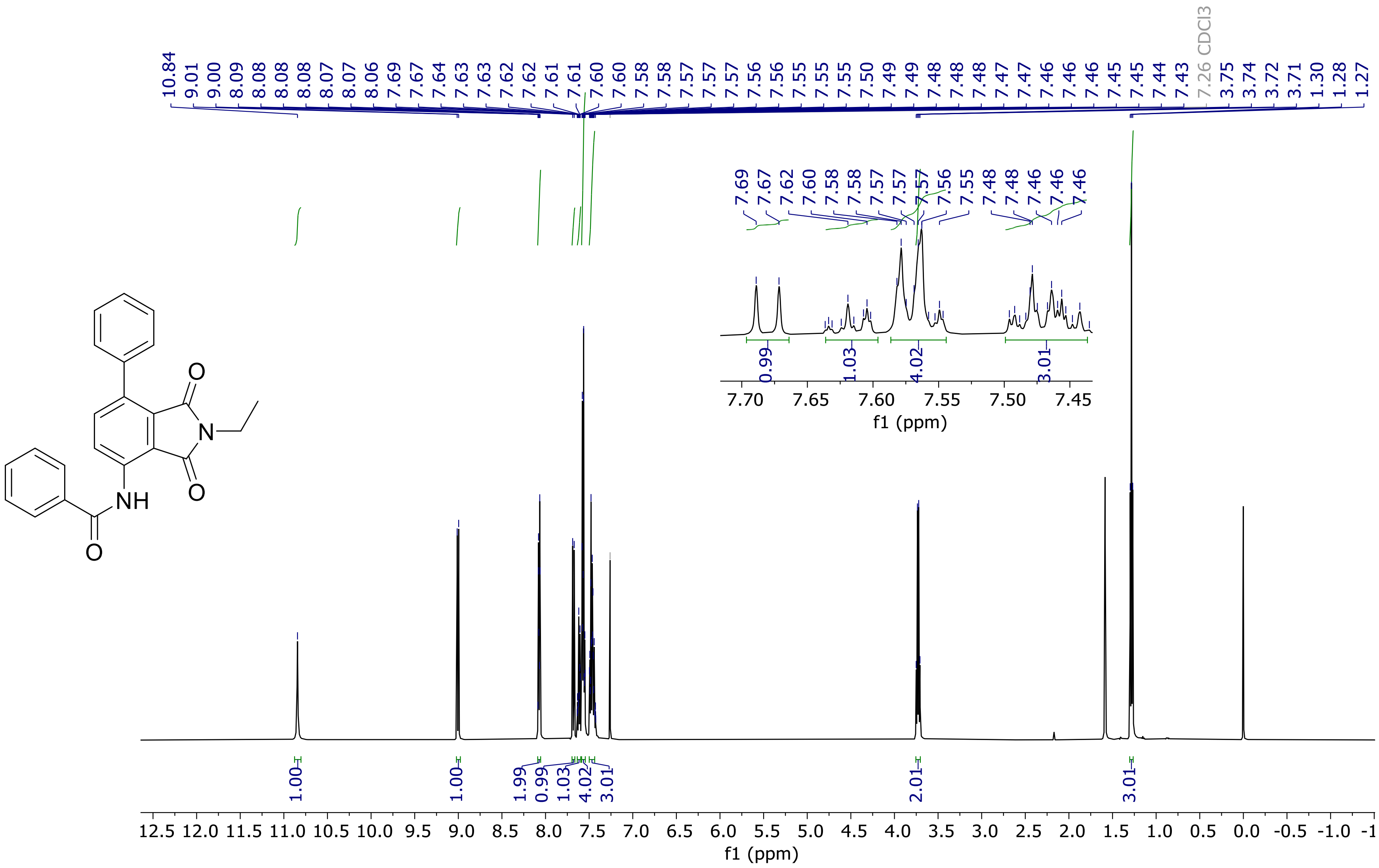

Figure S24. $^{13}C\{^{1}H\}$ NMR Spectrum (125 MHz, $CDCl_3$) for Aromatic **2a**

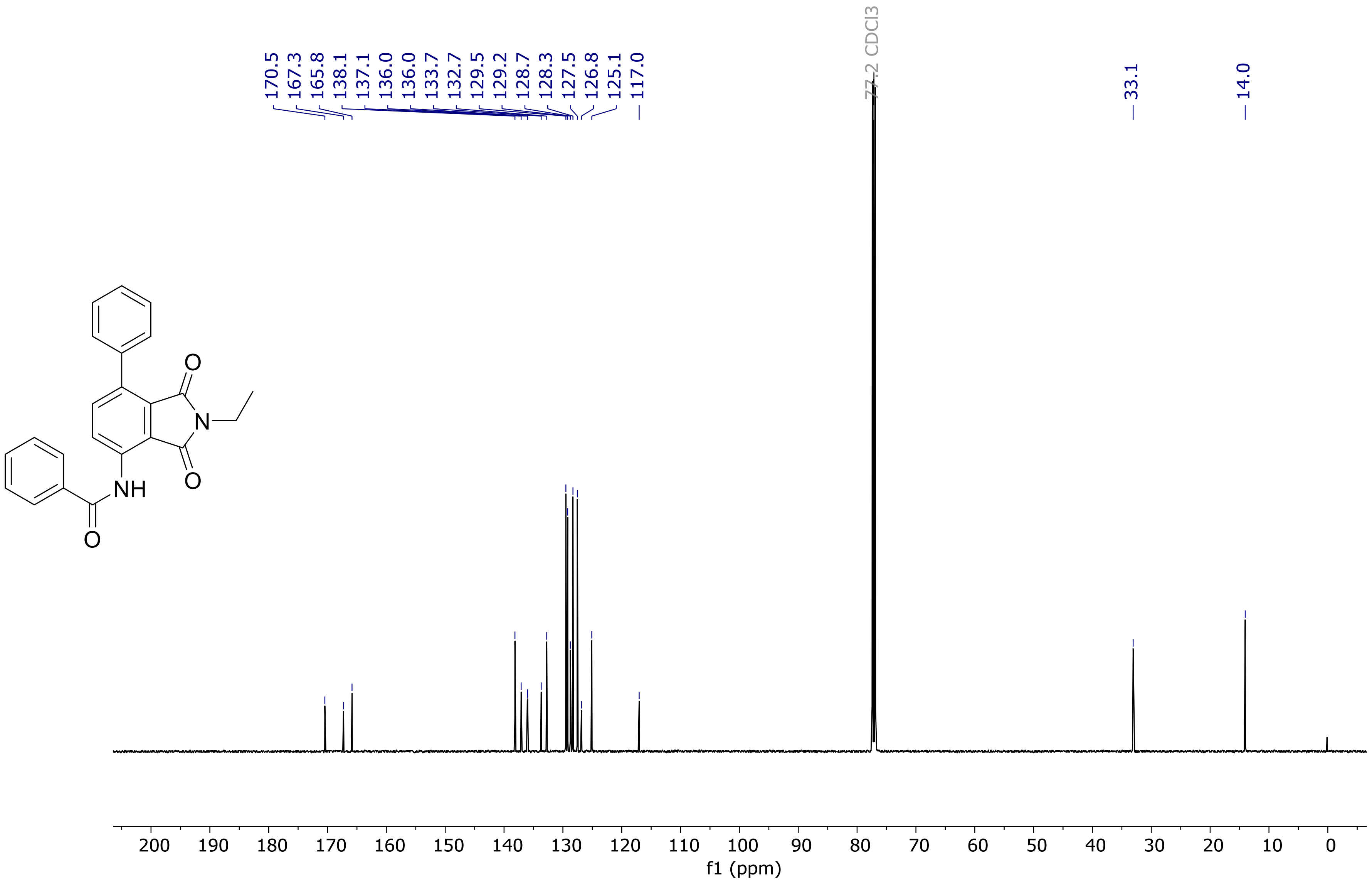

Figure S25. ^{1}H NMR Spectrum (500 MHz, $CDCl_3$) for Aromatic **2b**

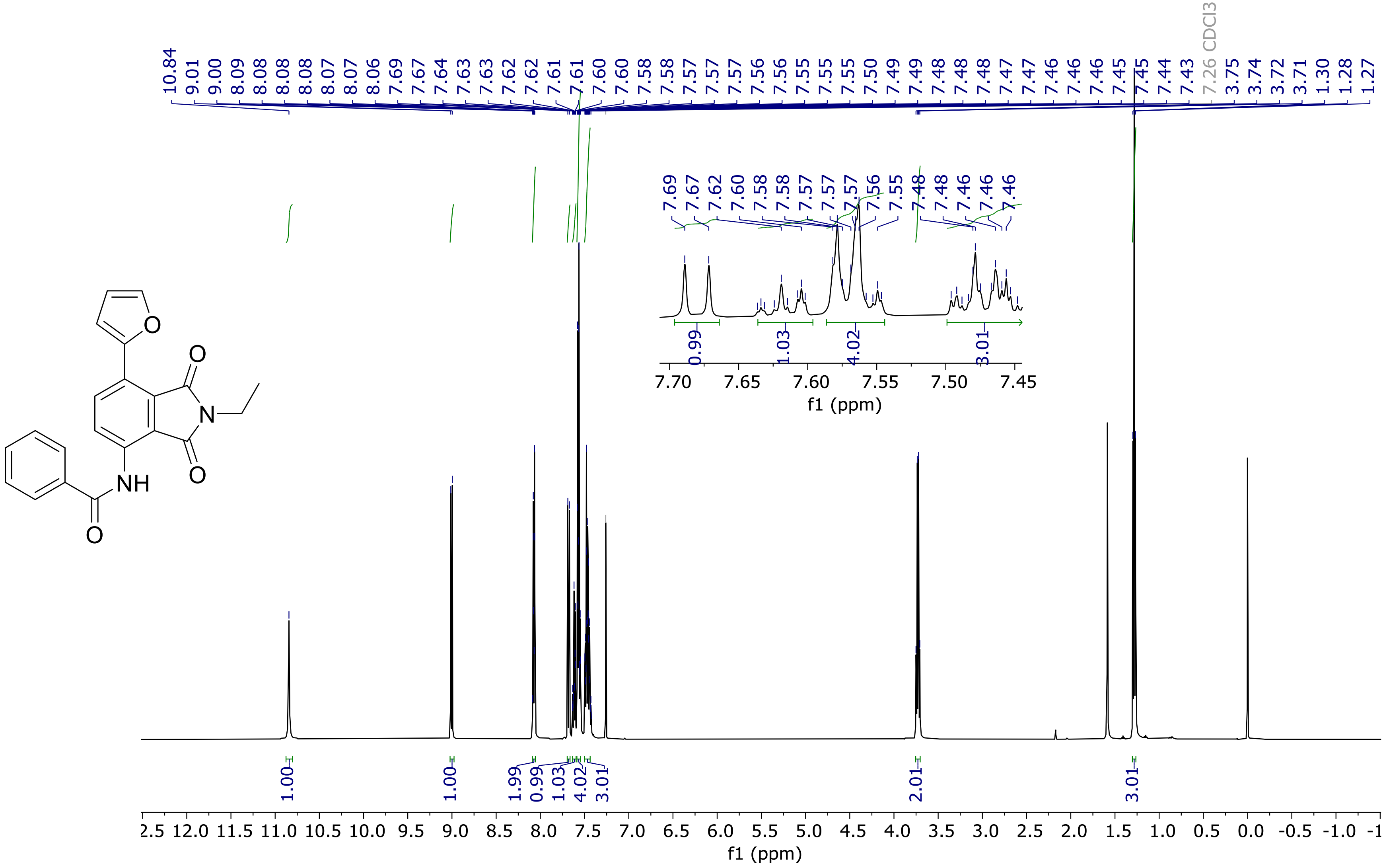

Figure S26. $^{13}C\{^{1}H\}$ NMR Spectrum (125 MHz, $CDCl_3$) for Aromatic **2b**

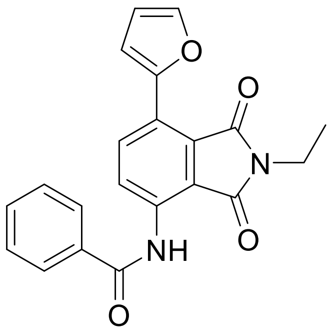

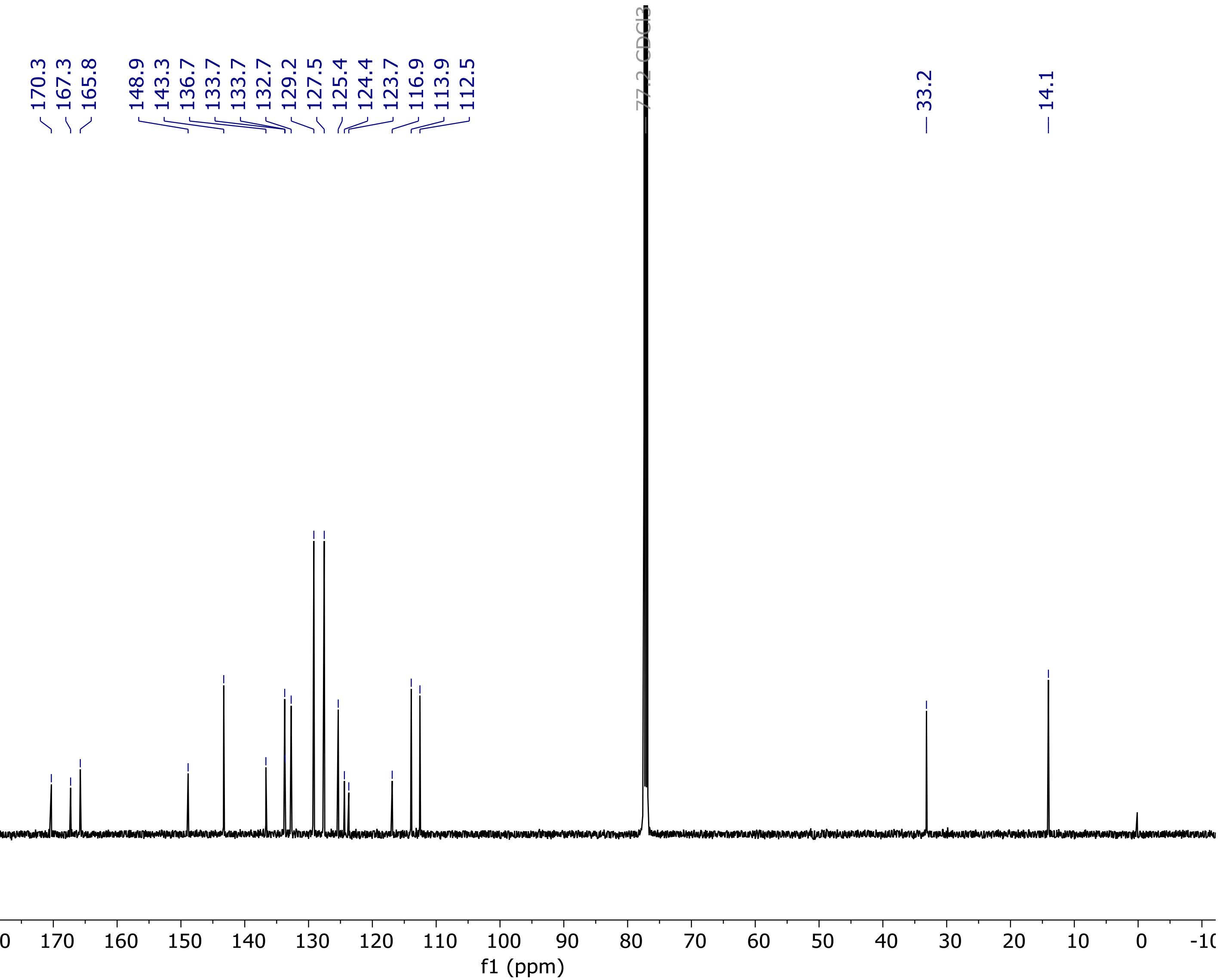

Figure S27. ^{1}H NMR Spectrum (500 MHz, $CDCl_3$) for Aromatic **2c**

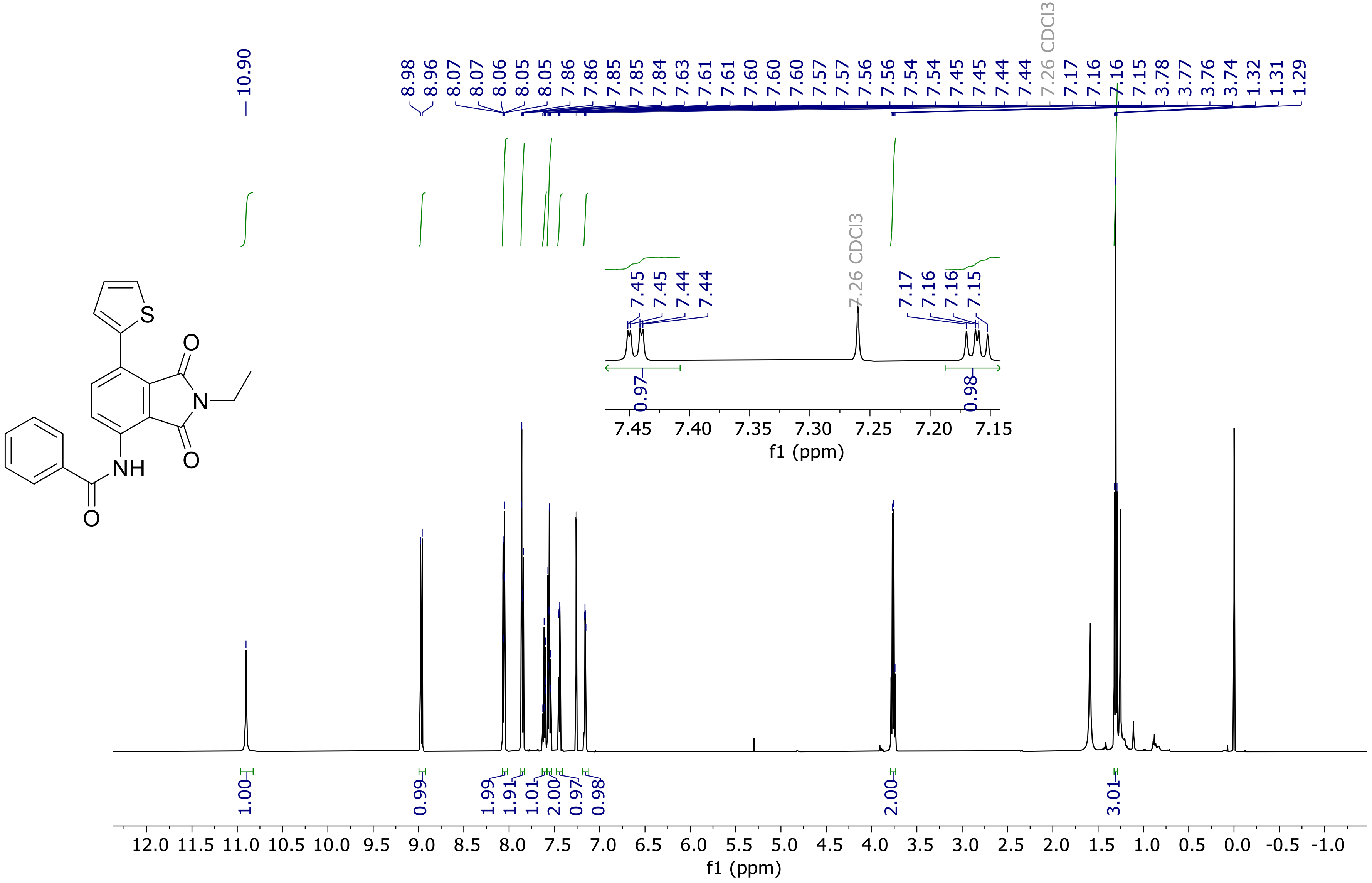

Figure S28. $^{13}C\{^1H\}$ NMR Spectrum (125 MHz, $CDCl_3$) for Aromatic **2c**

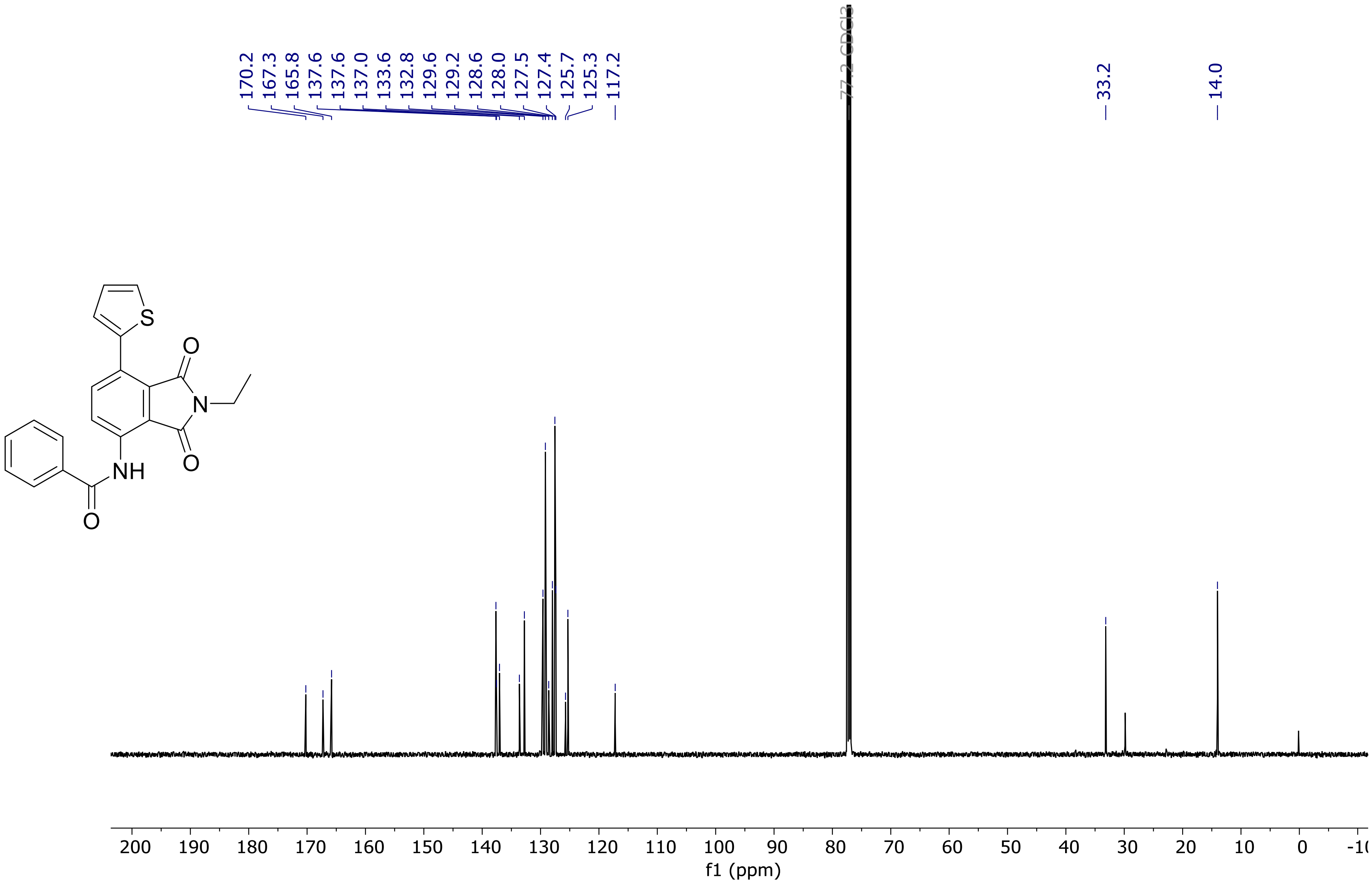

Figure S29. ¹H NMR Spectrum (500 MHz, $CDCl_3$) for Aromatic **2d**

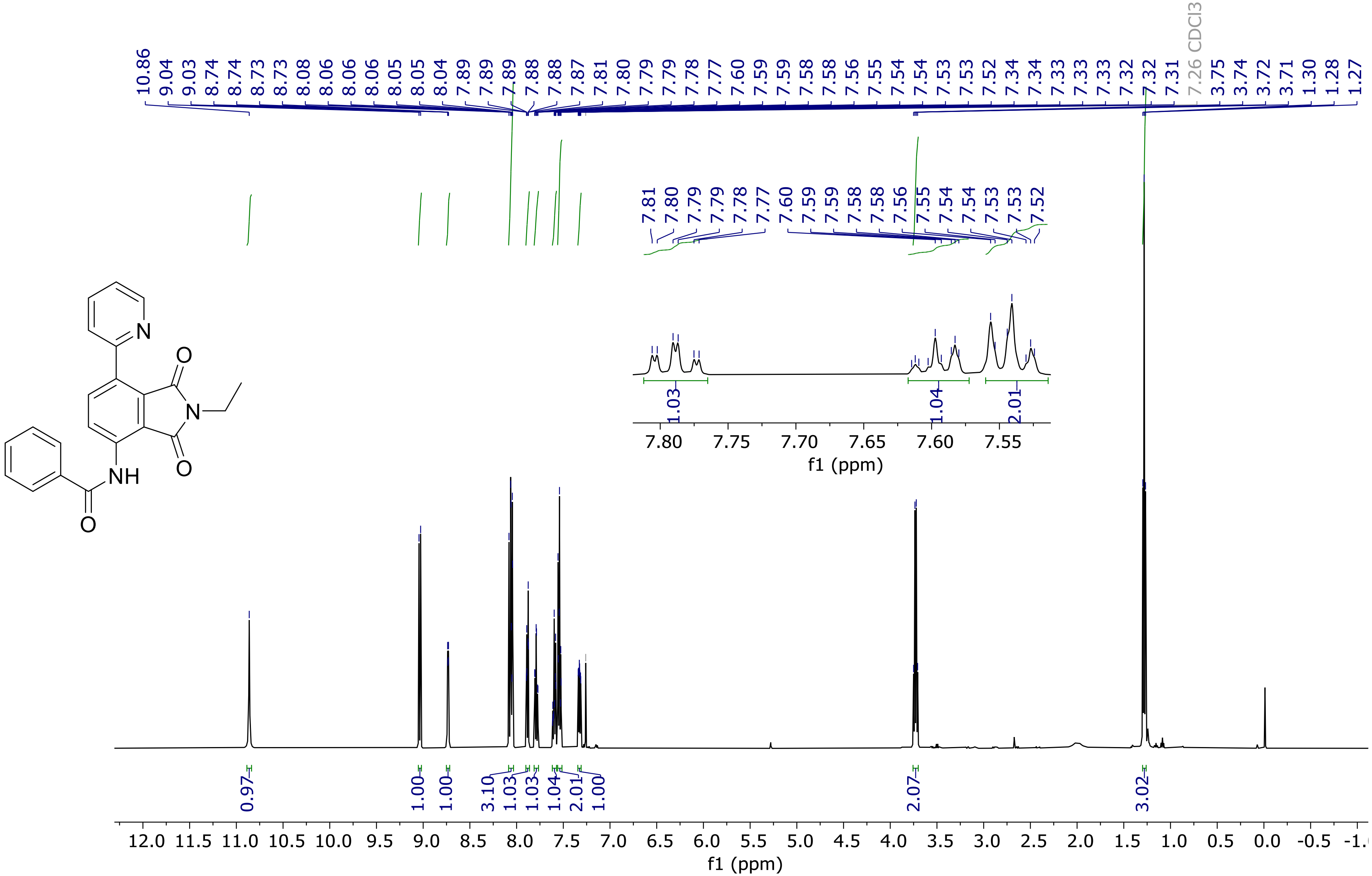

Figure S30. $^{13}C\{^{1}H\}$ NMR Spectrum (125 MHz, $CDCl_3$) for Aromatic **2d**

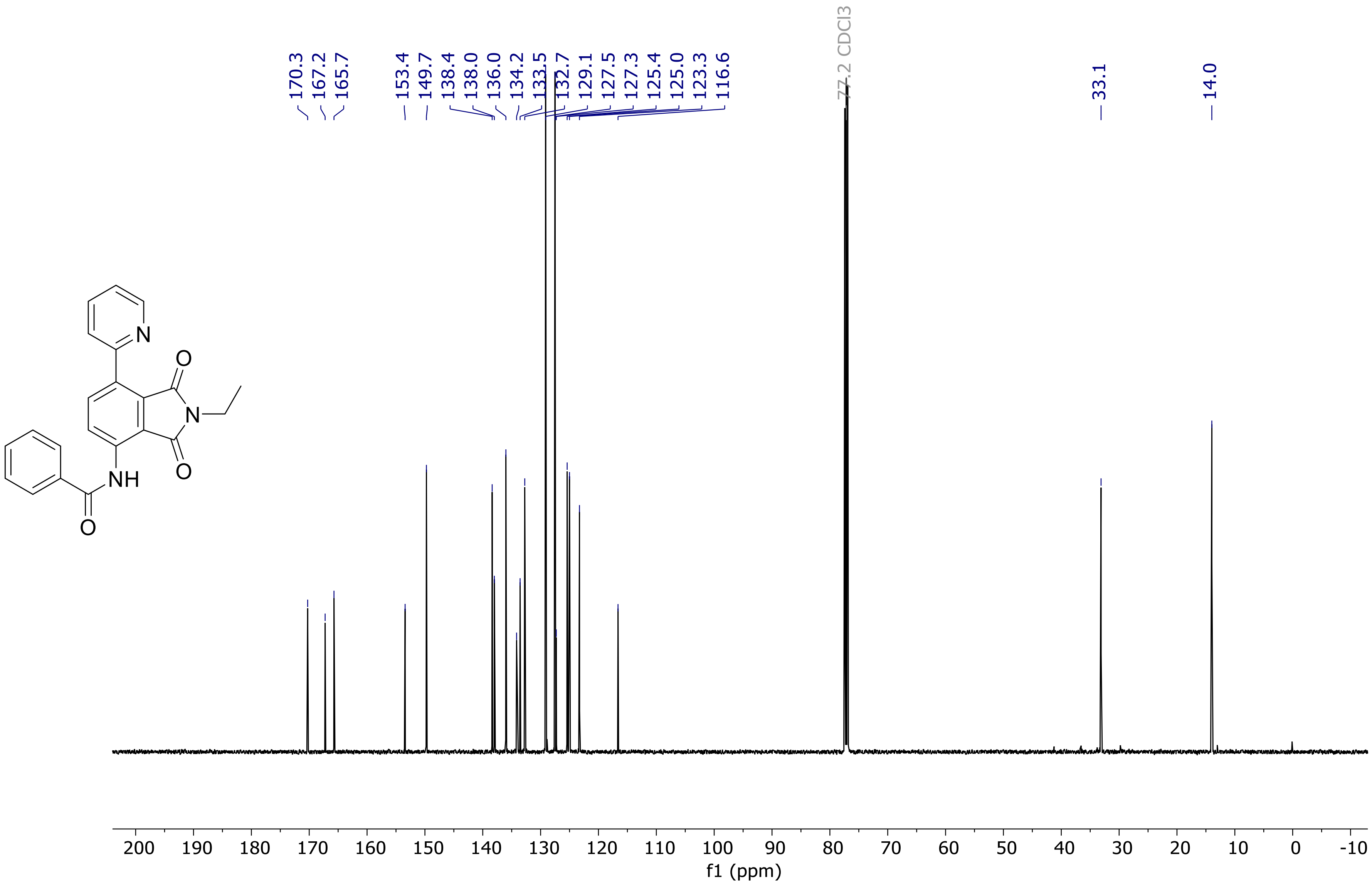

Figure S31. ^{1}H NMR Spectrum (500 MHz, $CDCl_3$) for Aromatic **2e**

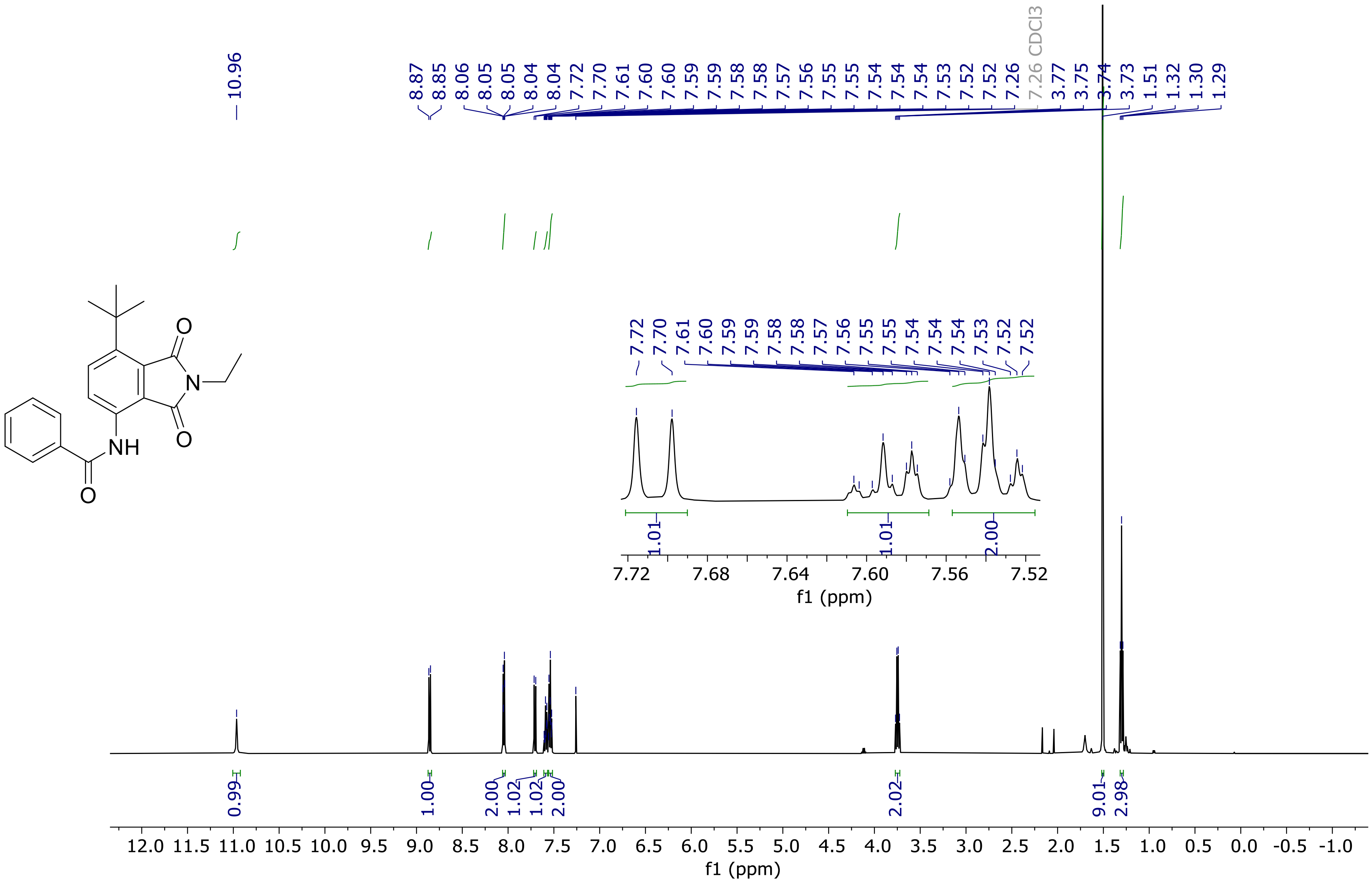

Figure S32. $^{13}C\{^{1}H\}$ NMR Spectrum (125 MHz, $CDCl_3$) for Aromatic **2e**

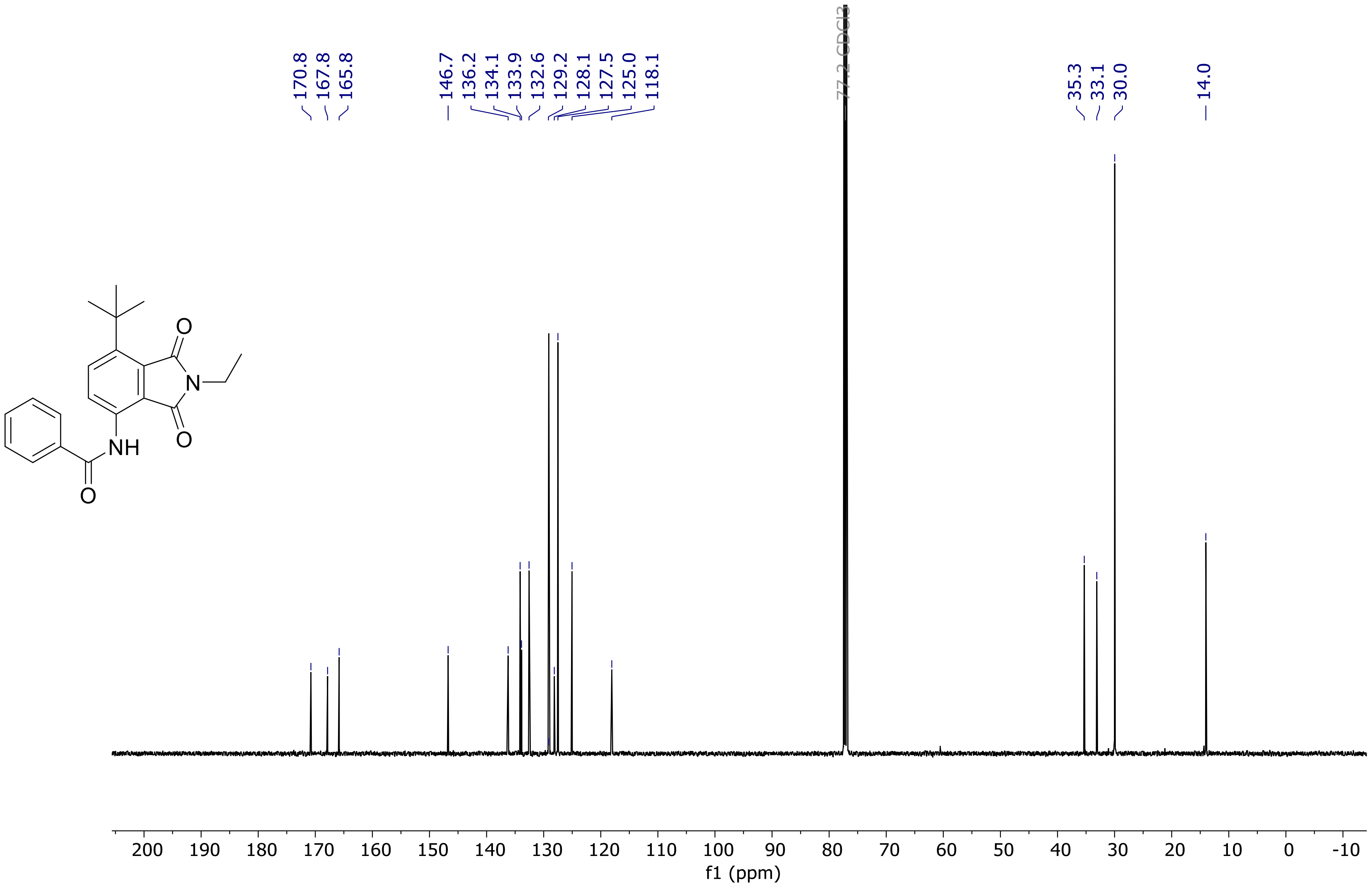

Figure S33. ^{1}H NMR Spectrum (500 MHz, $CDCl_3$) for Aromatic **2e'**

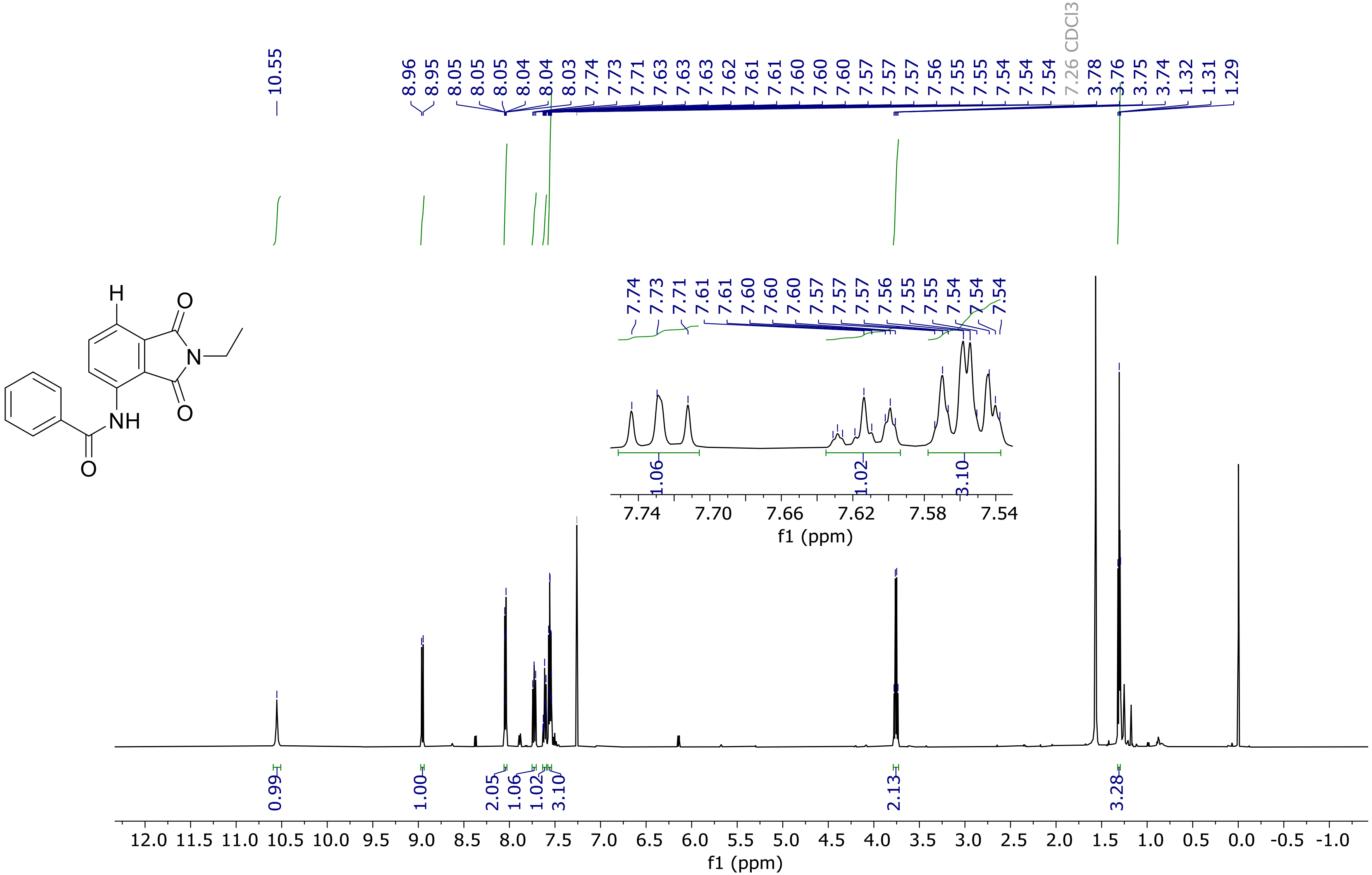

Figure S34. $^{13}C\{^1H\}$ NMR Spectrum (125 MHz, $CDCl_3$) for Aromatic **2e'**

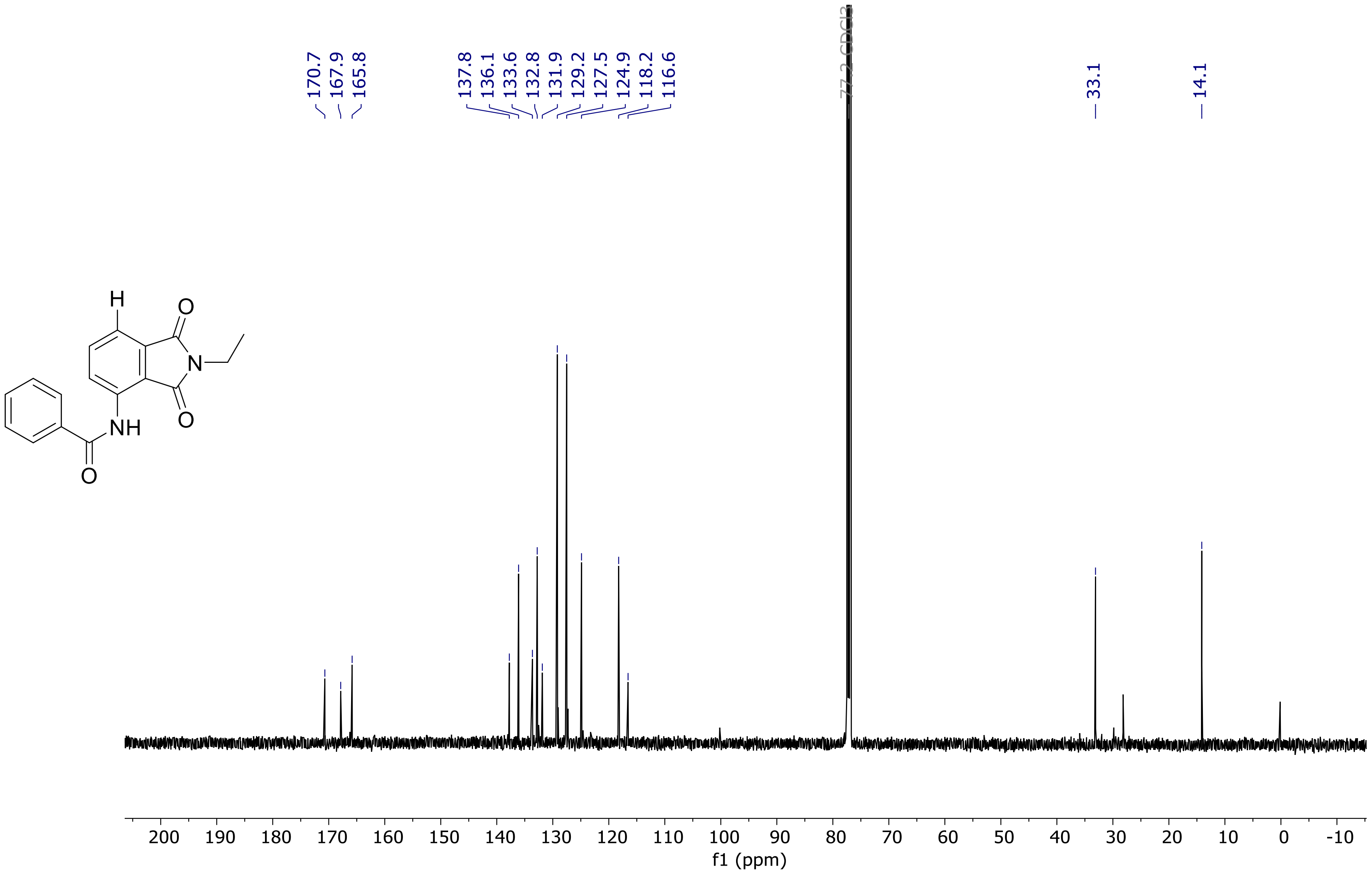

Figure S35. ^{1}H NMR Spectrum (500 MHz, $CDCl_3$) for Aromatic **2f**

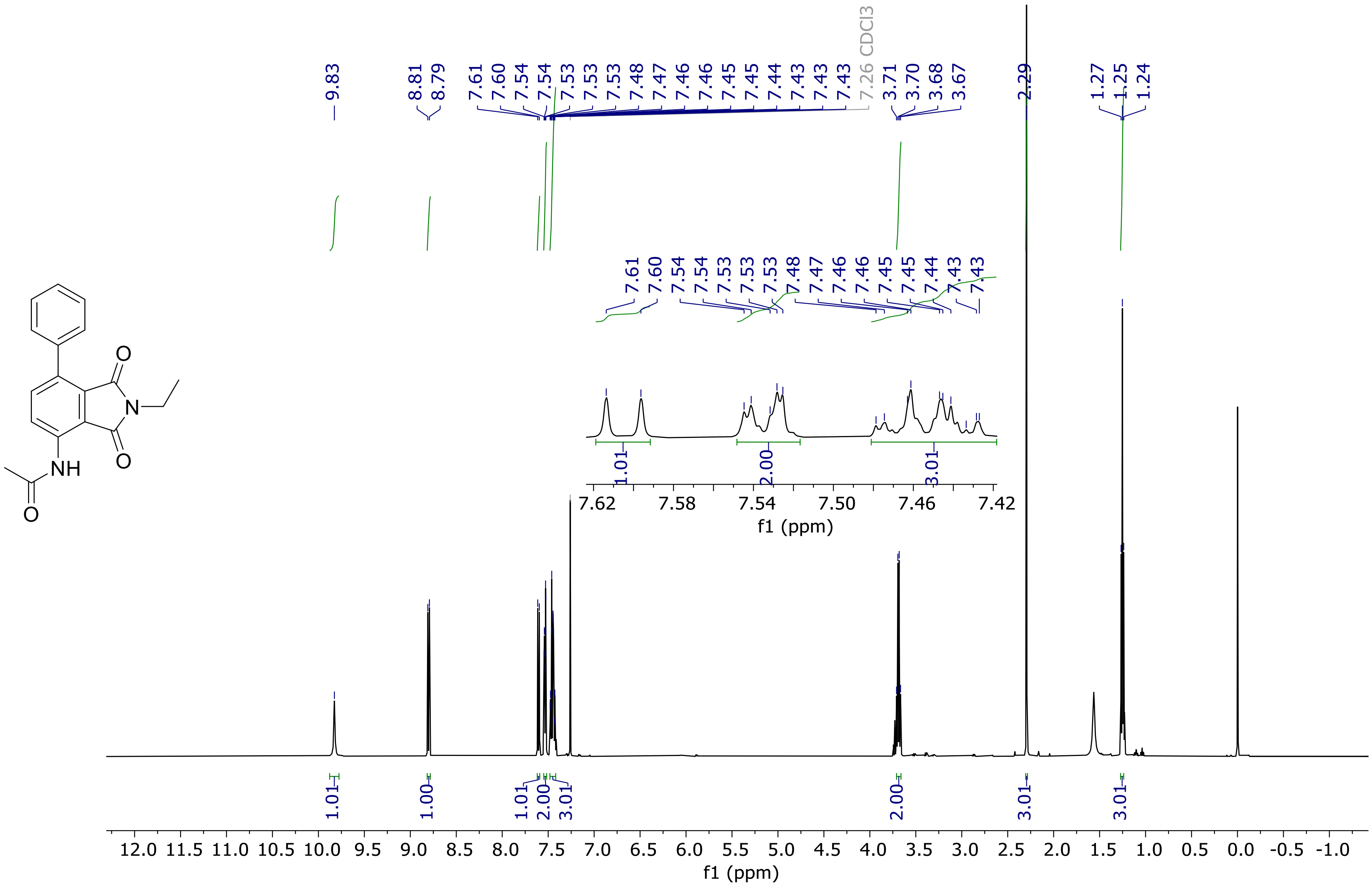

Figure S36. $^{13}C\{^{1}H\}$ NMR Spectrum (125 MHz, $CDCl_3$) for Aromatic **2f**

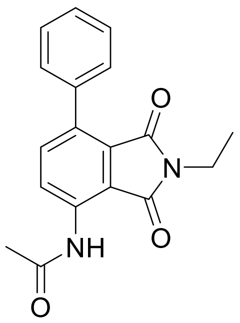

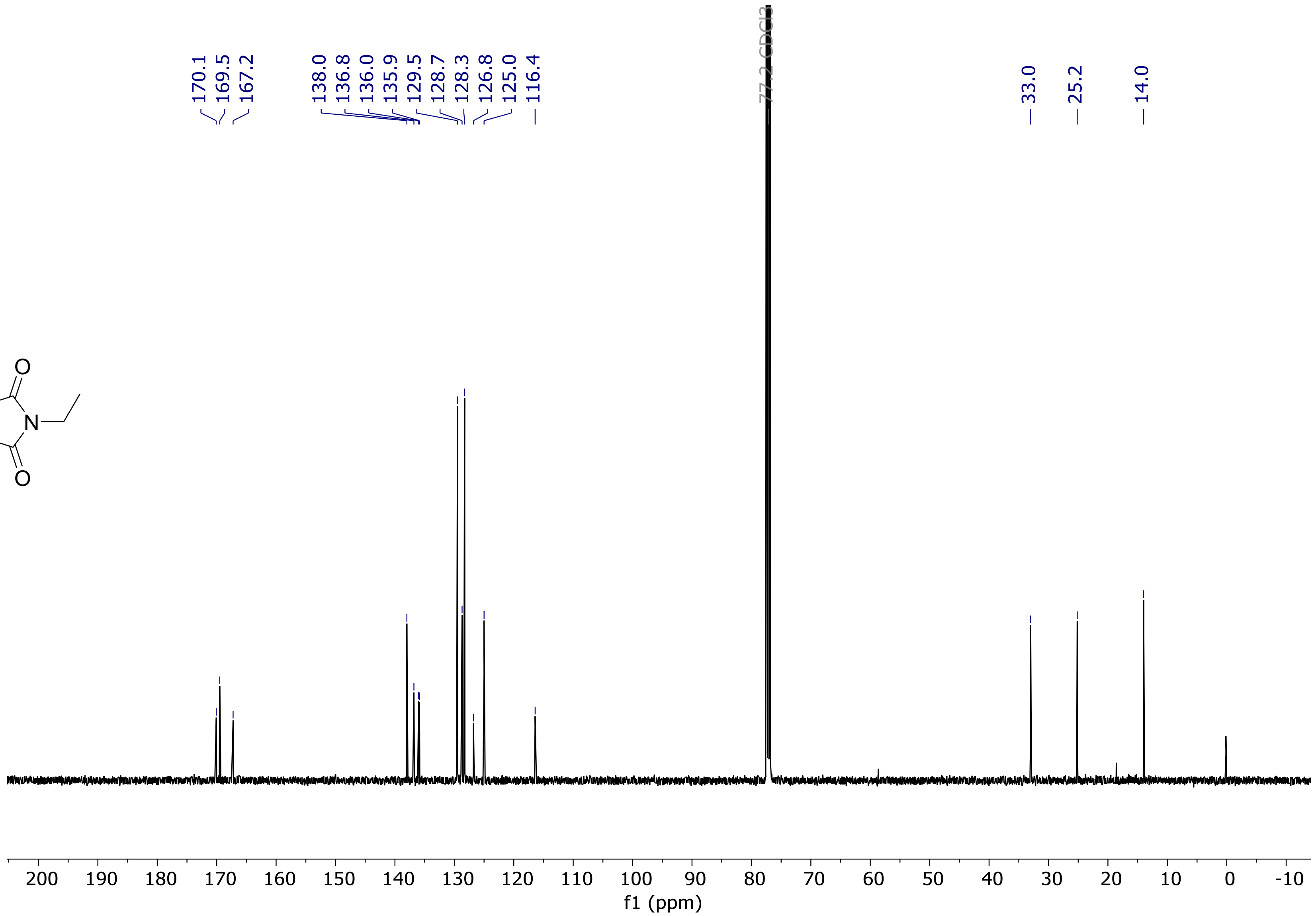

Figure S37. ^{1}H NMR Spectrum (500 MHz, $CDCl_3$) for Aromatic **2g**

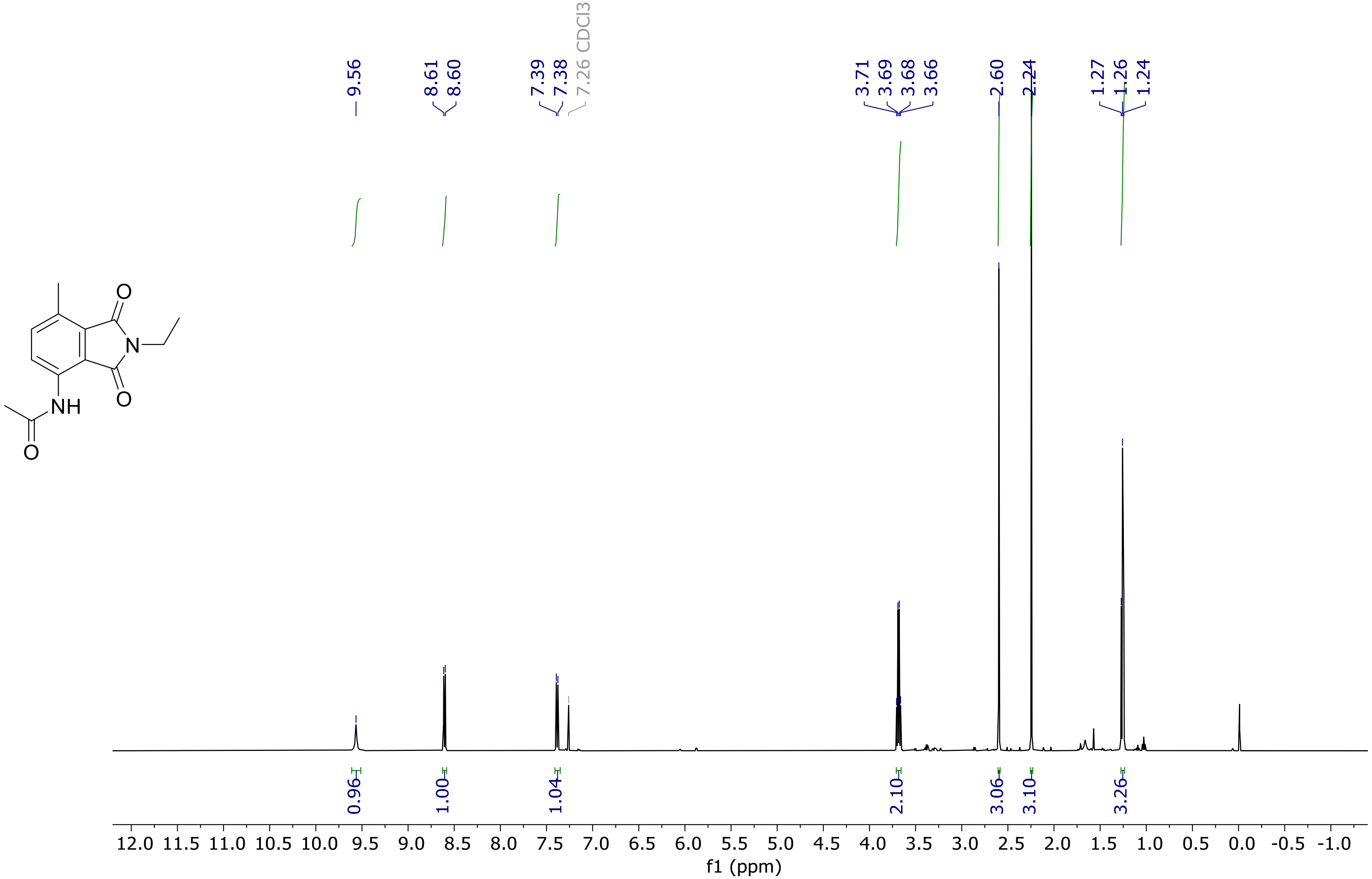

Figure S38. $^{13}C\{^1H\}$ NMR Spectrum (125 MHz, $CDCl_3$) for Aromatic **2g**

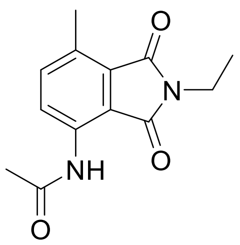

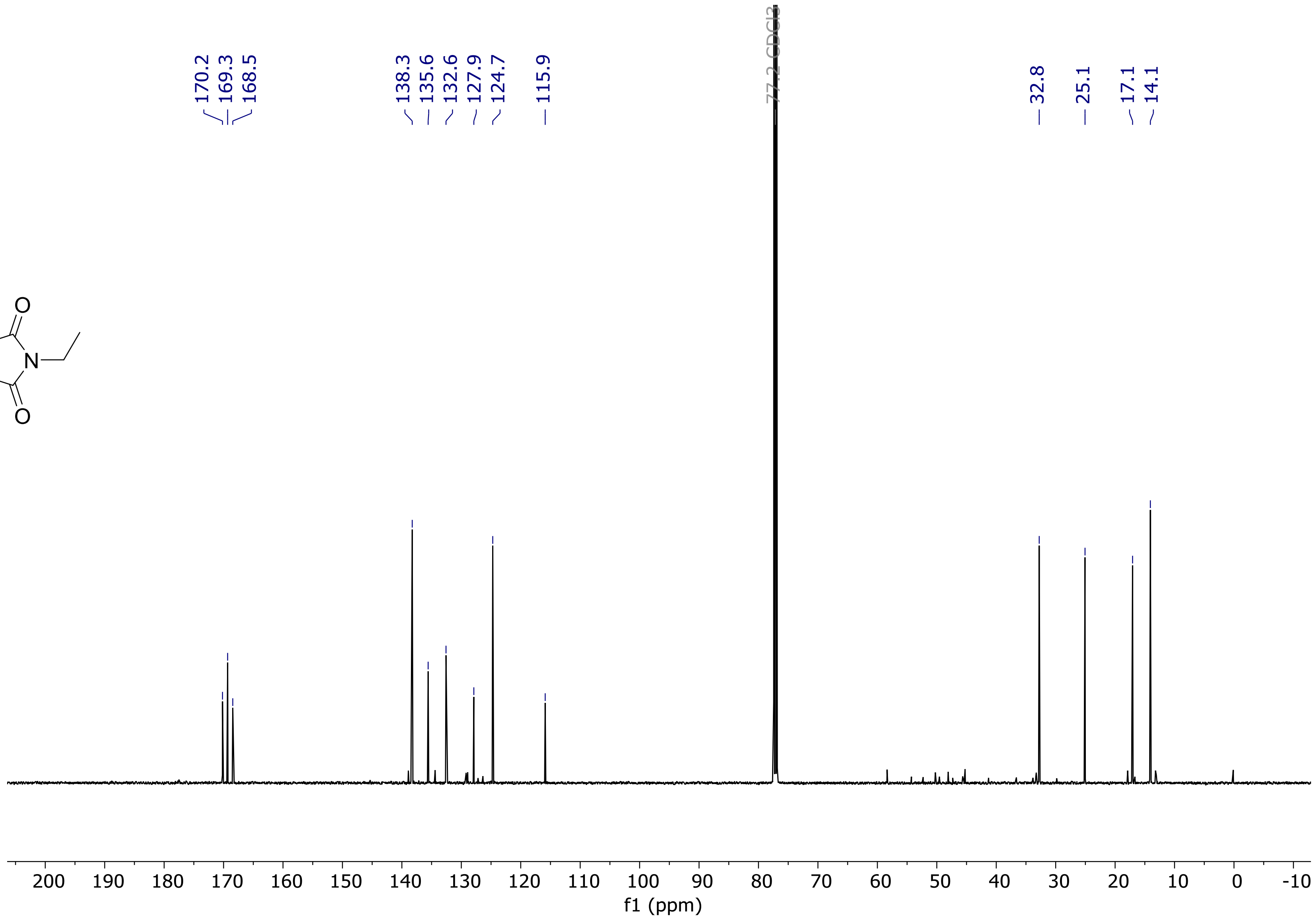

Figure S39. ^{1}H NMR Spectrum (500 MHz, $CDCl_3$) for Aromatic **2h**

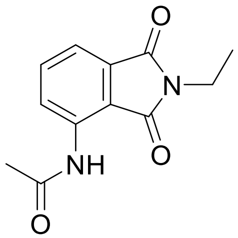

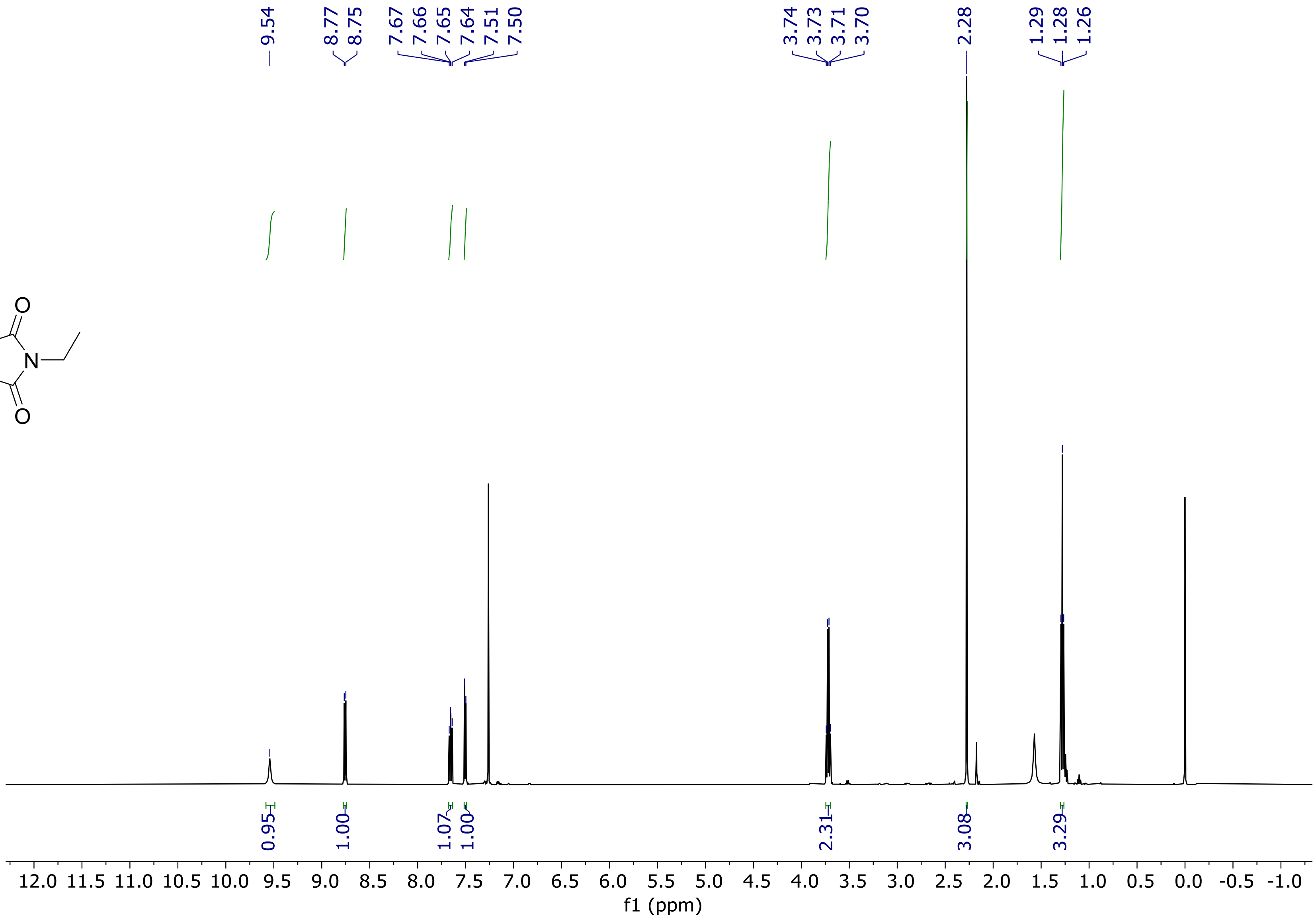

Figure S40. $^{13}C\{^{1}H\}$ NMR Spectrum (125 MHz, $CDCl_3$) for Aromatic **2h**

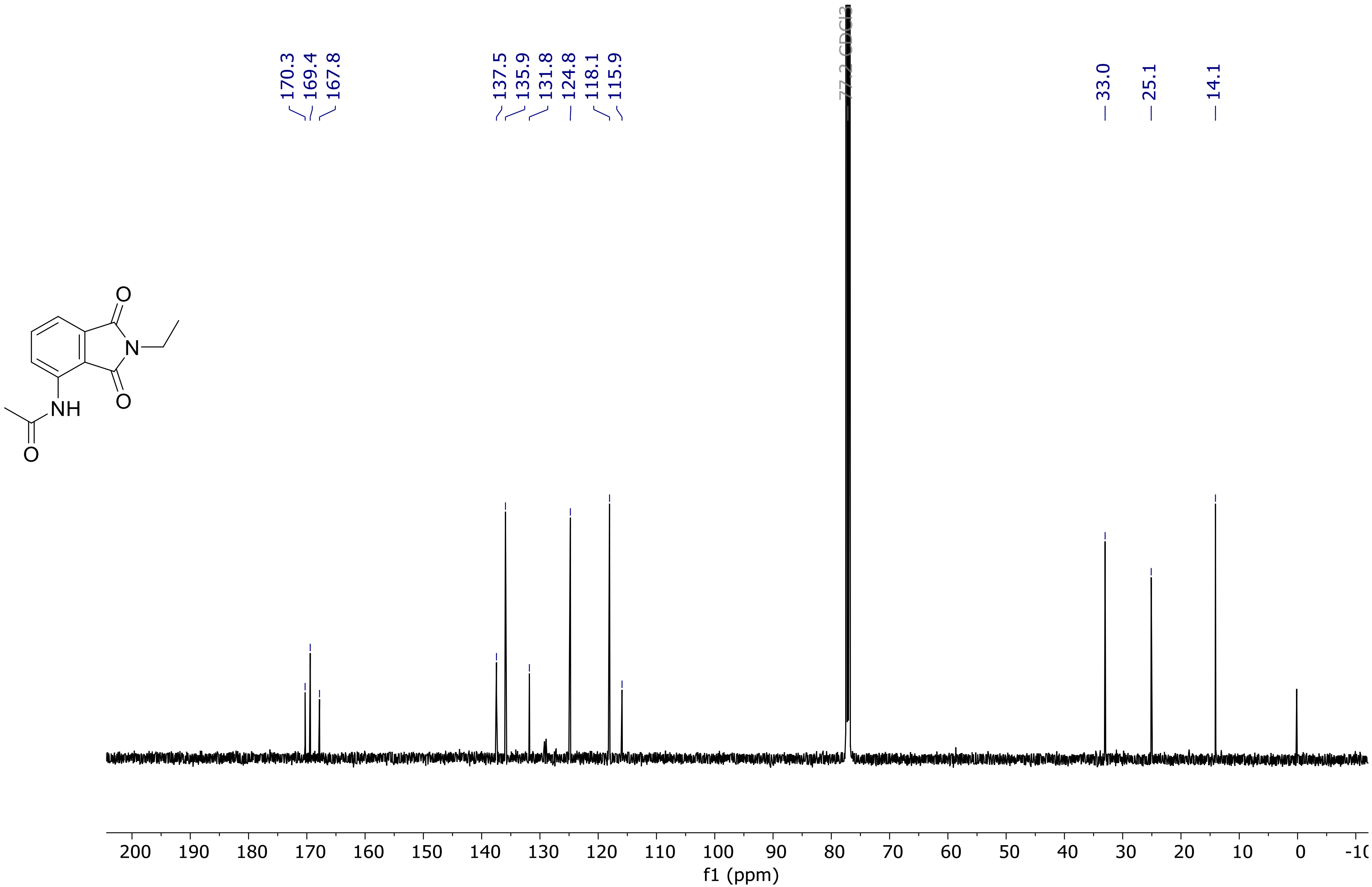

Figure S41. ^{1}H NMR Spectrum (500 MHz, $CDCl_3$) for Aromatic **2i**

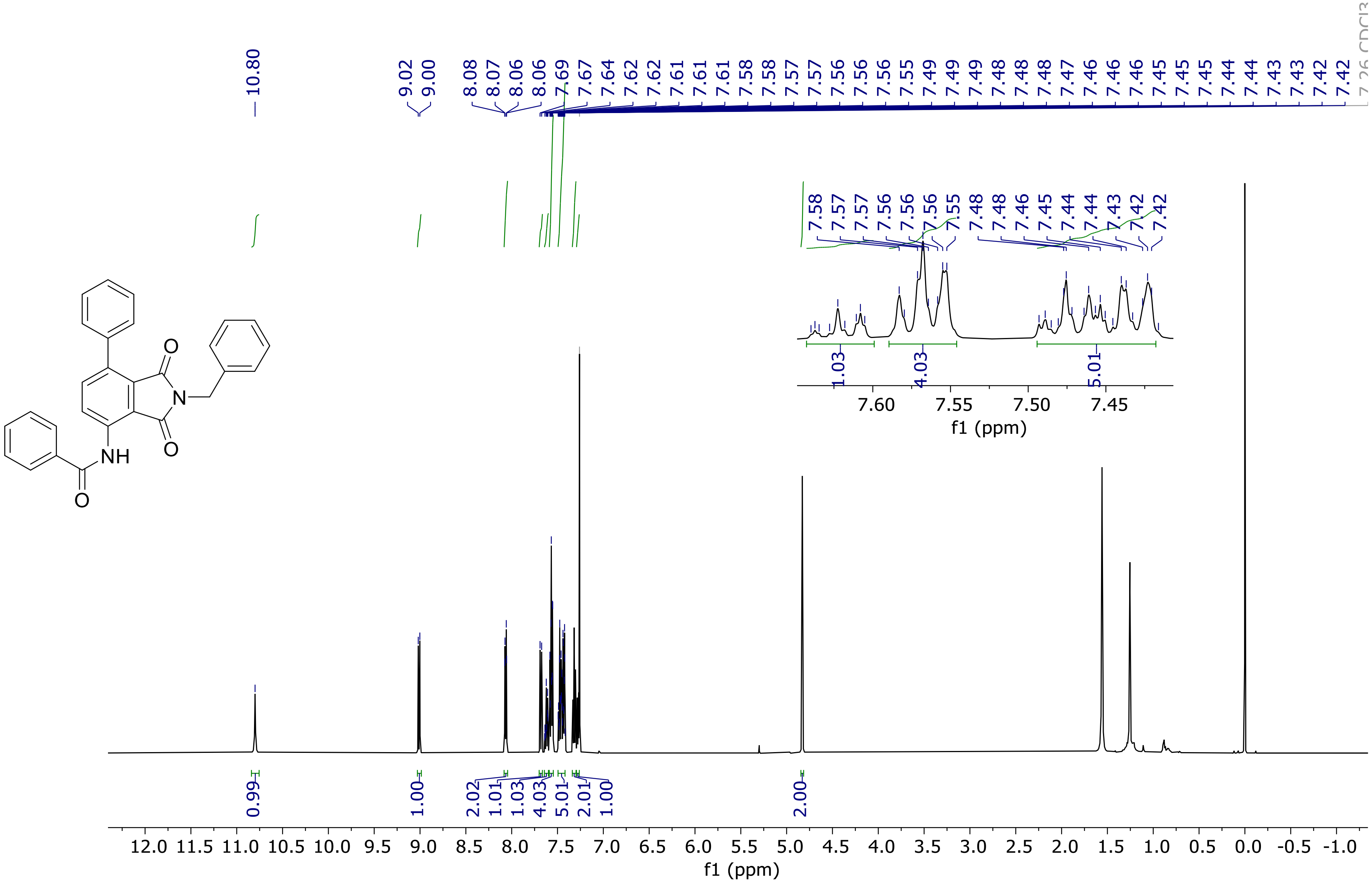

Figure S42. $^{13}C\{^{1}H\}$ NMR Spectrum (125 MHz, $CDCl_3$) for Aromatic **2i**

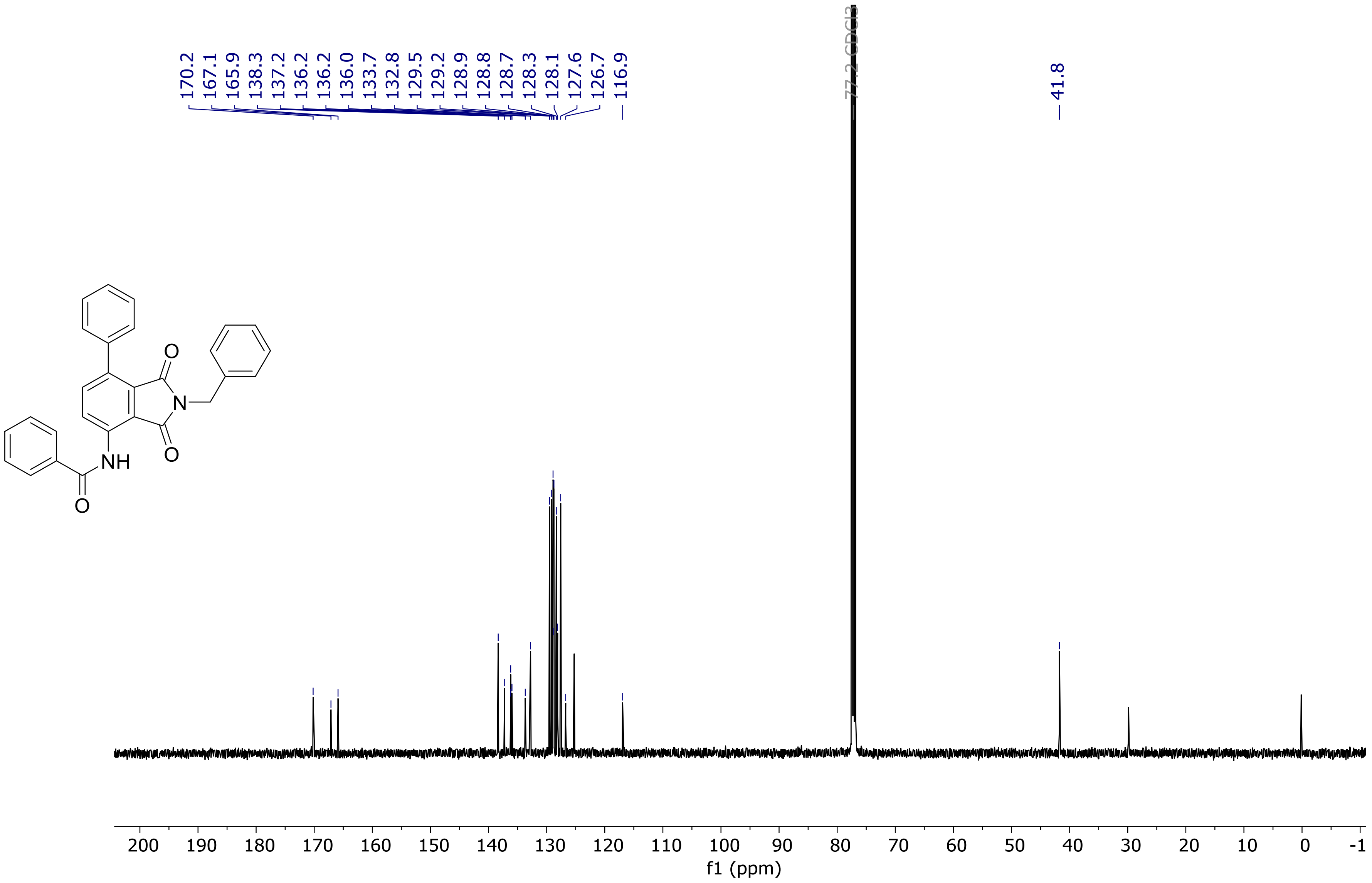

Figure S43. ^{1}H NMR Spectrum (500 MHz, $CDCl_3$) for Aromatic **2j**

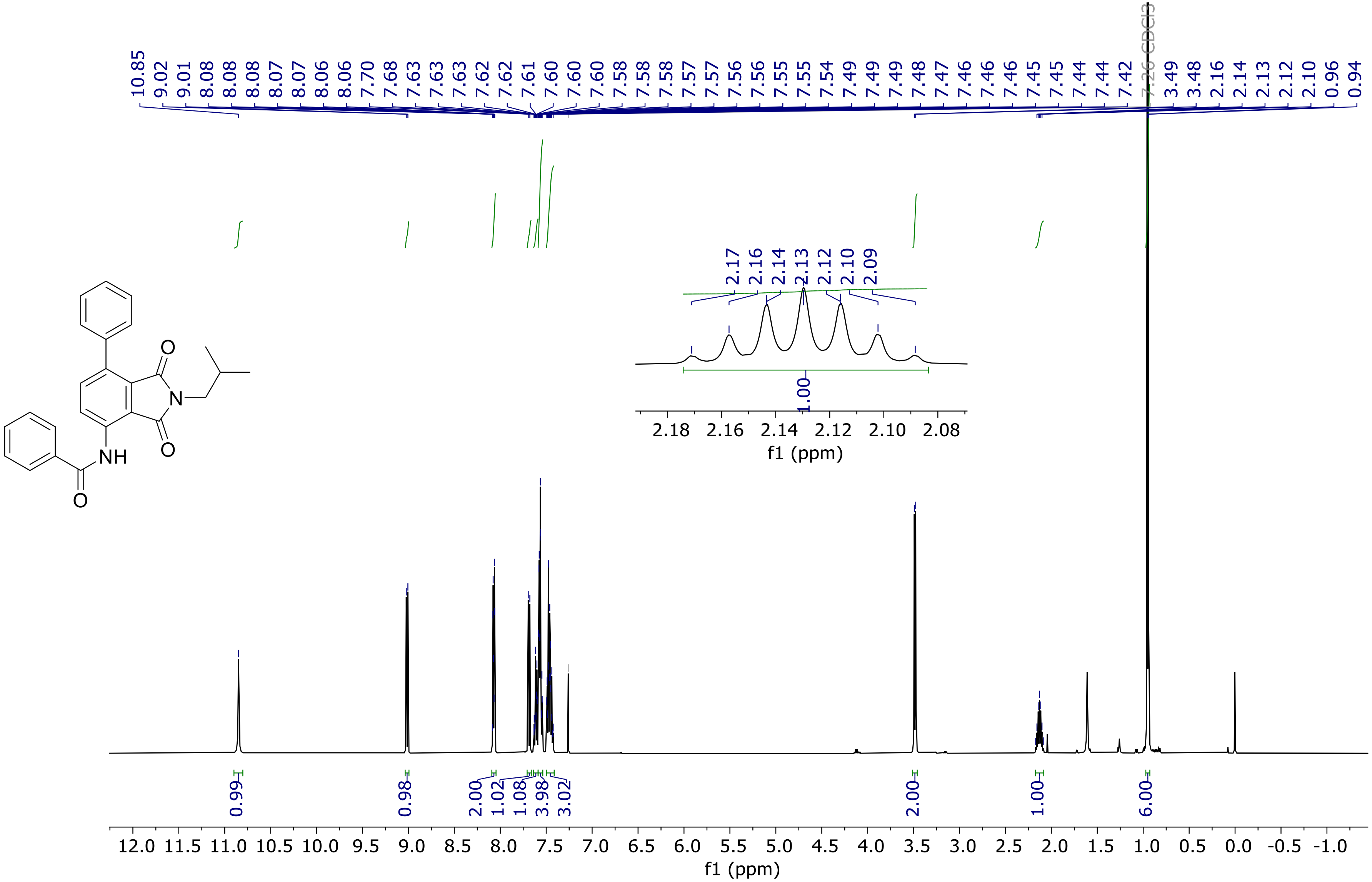

Figure S44. $^{13}C\{^1H\}$ NMR Spectrum (125 MHz, $CDCl_3$) for Aromatic **2j**

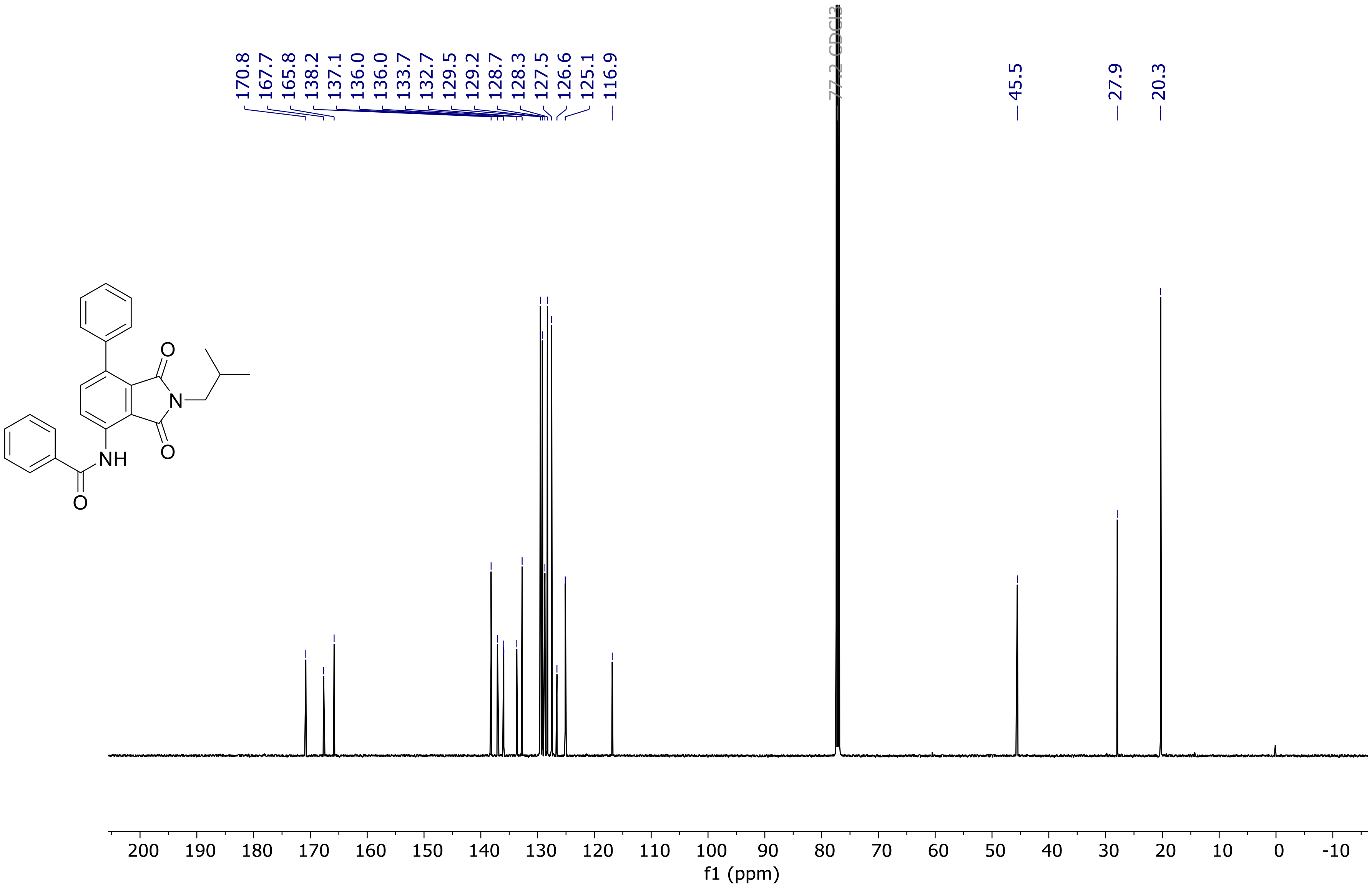

Figure S45. ^{1}H NMR Spectrum (500 MHz, $CDCl_3$) for Aromatic **2k**

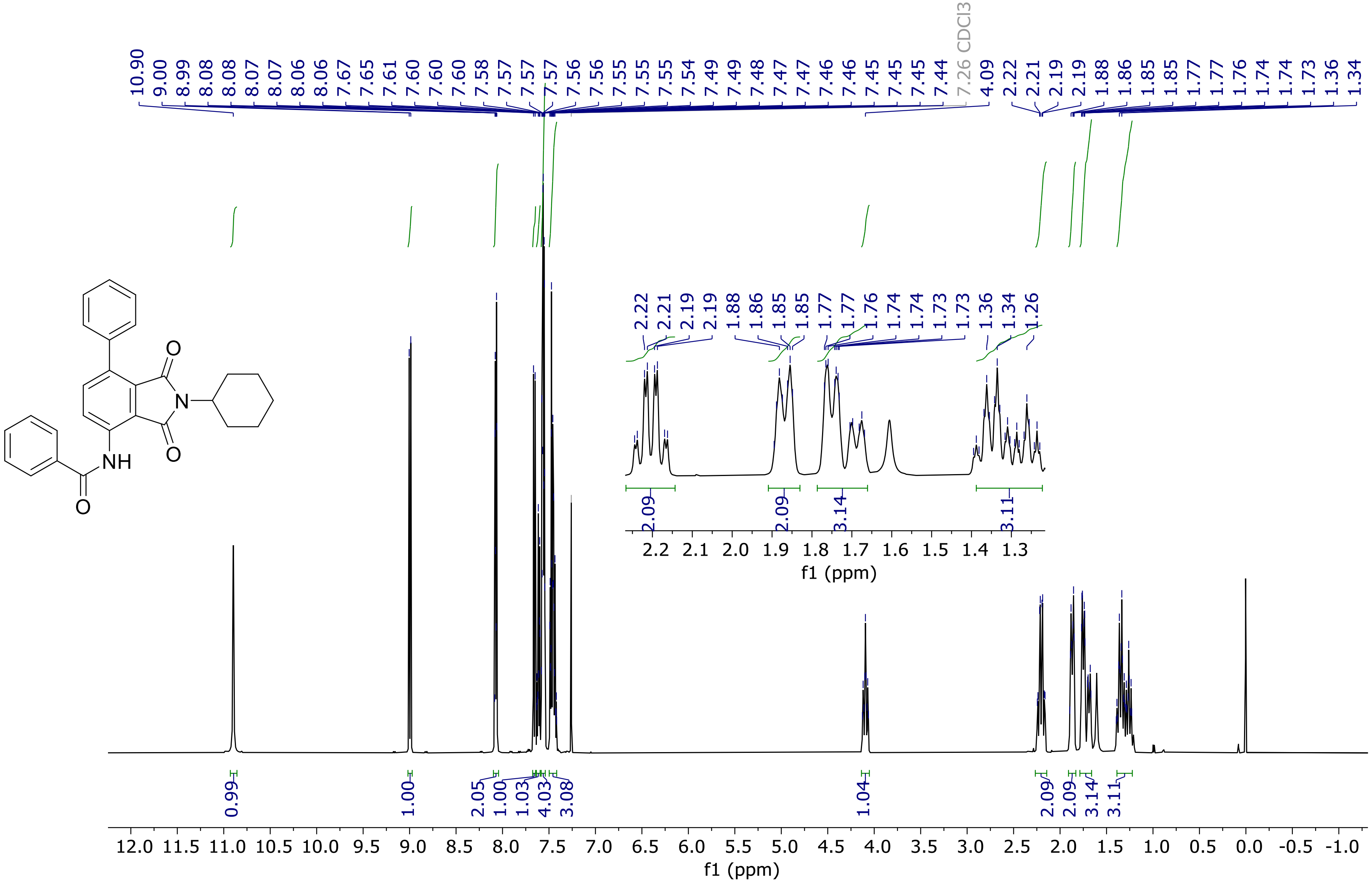

Figure S46. $^{13}C\{^{1}H\}$ NMR Spectrum (125 MHz, $CDCl_3$) for Aromatic **2k**

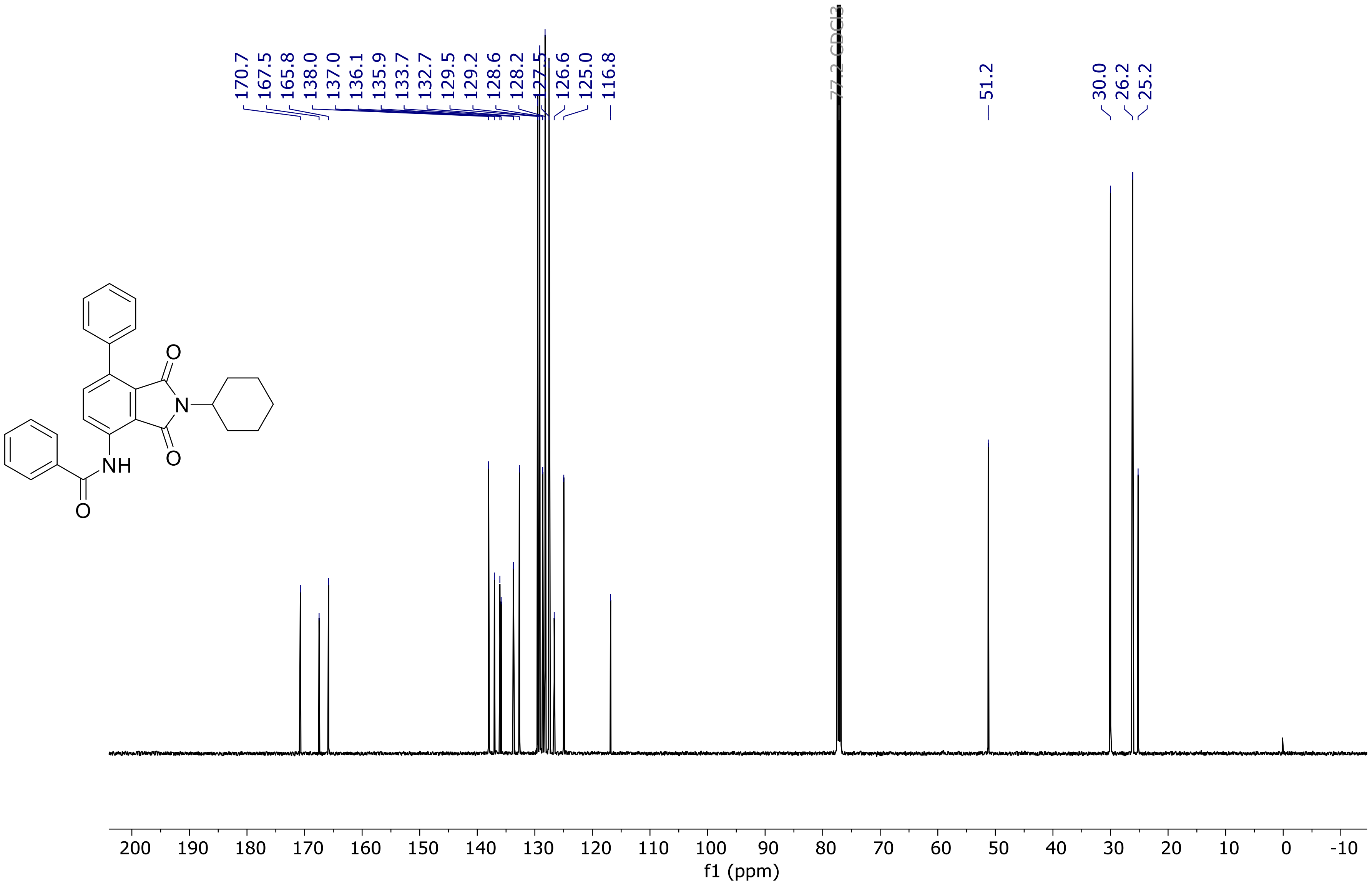

Figure S47. ^{1}H NMR Spectrum (600 MHz, $CDCl_3$) for Aromatic **2l**

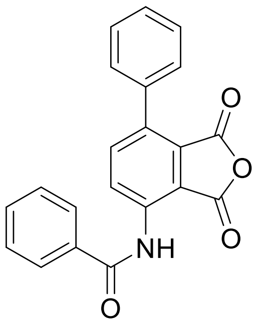

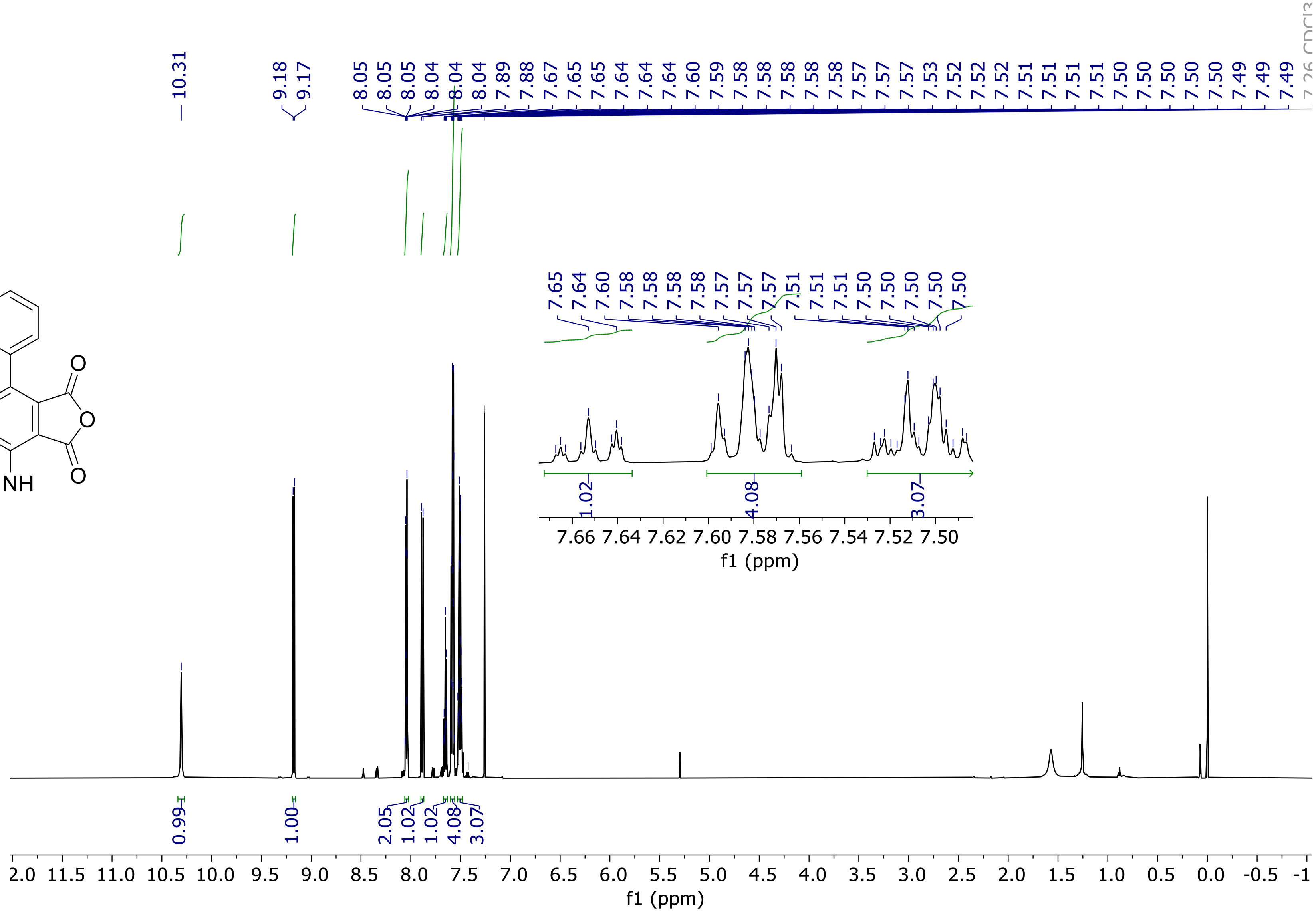

Figure S48. $^{13}C\{^{1}H\}$ NMR Spectrum (150 MHz, $CDCl_3$) for Aromatic **2l**

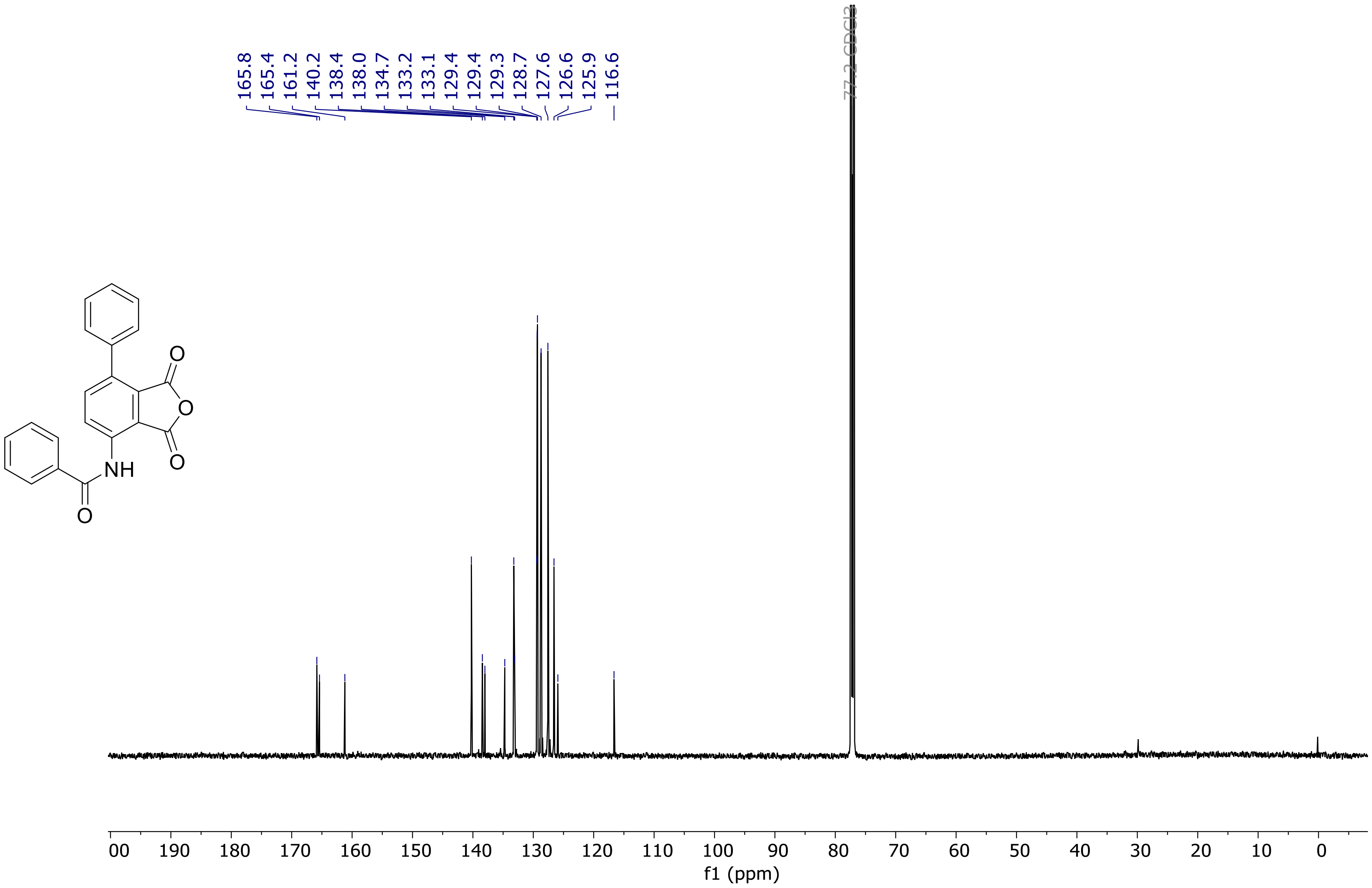

Figure S49. ^{1}H NMR Spectrum (500 MHz, $CDCl_3$) for Aromatic **2m**

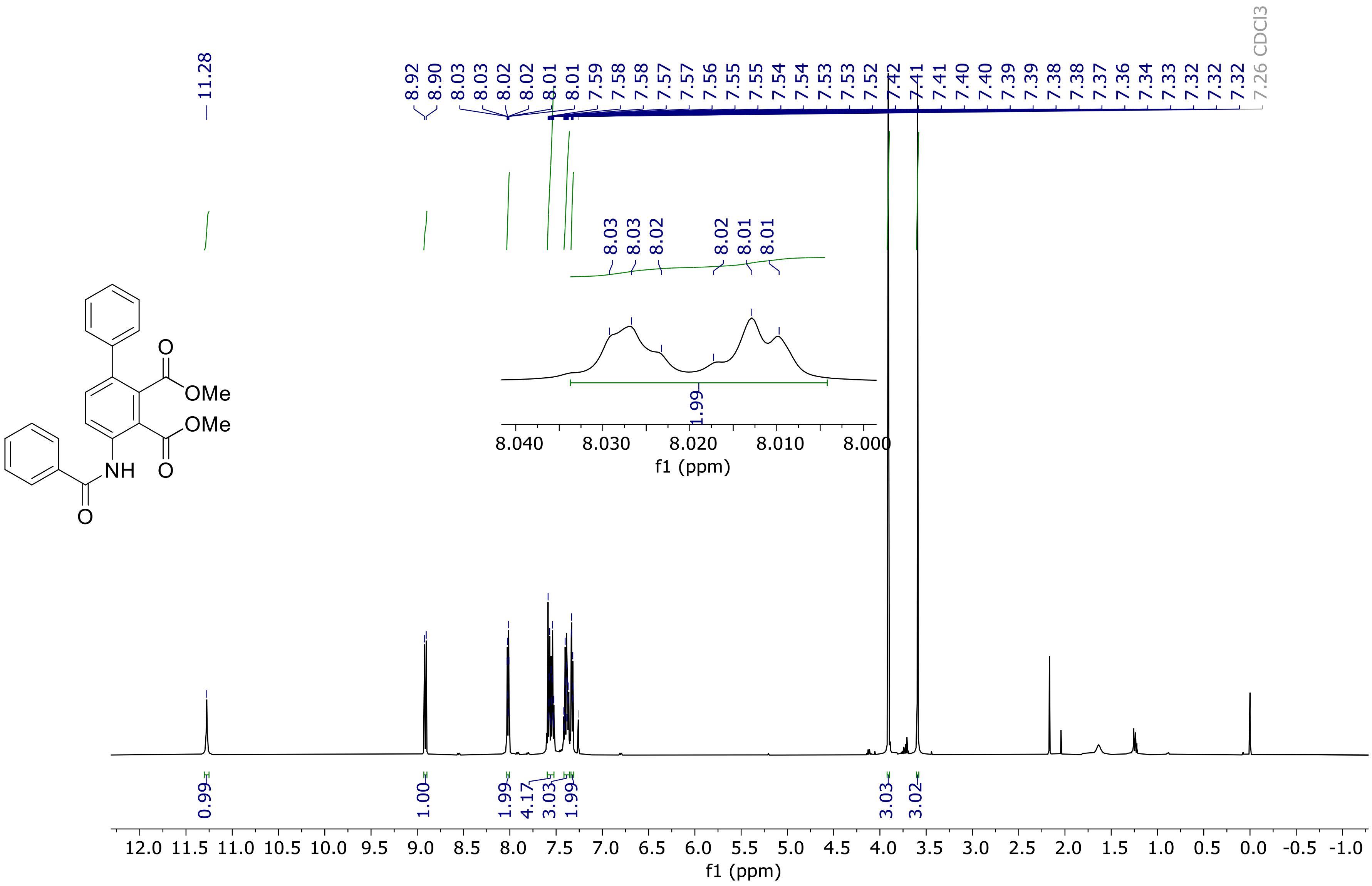

Figure S50. $^{13}C\{^1H\}$ NMR Spectrum (125 MHz, $CDCl_3$) for Aromatic **2m**

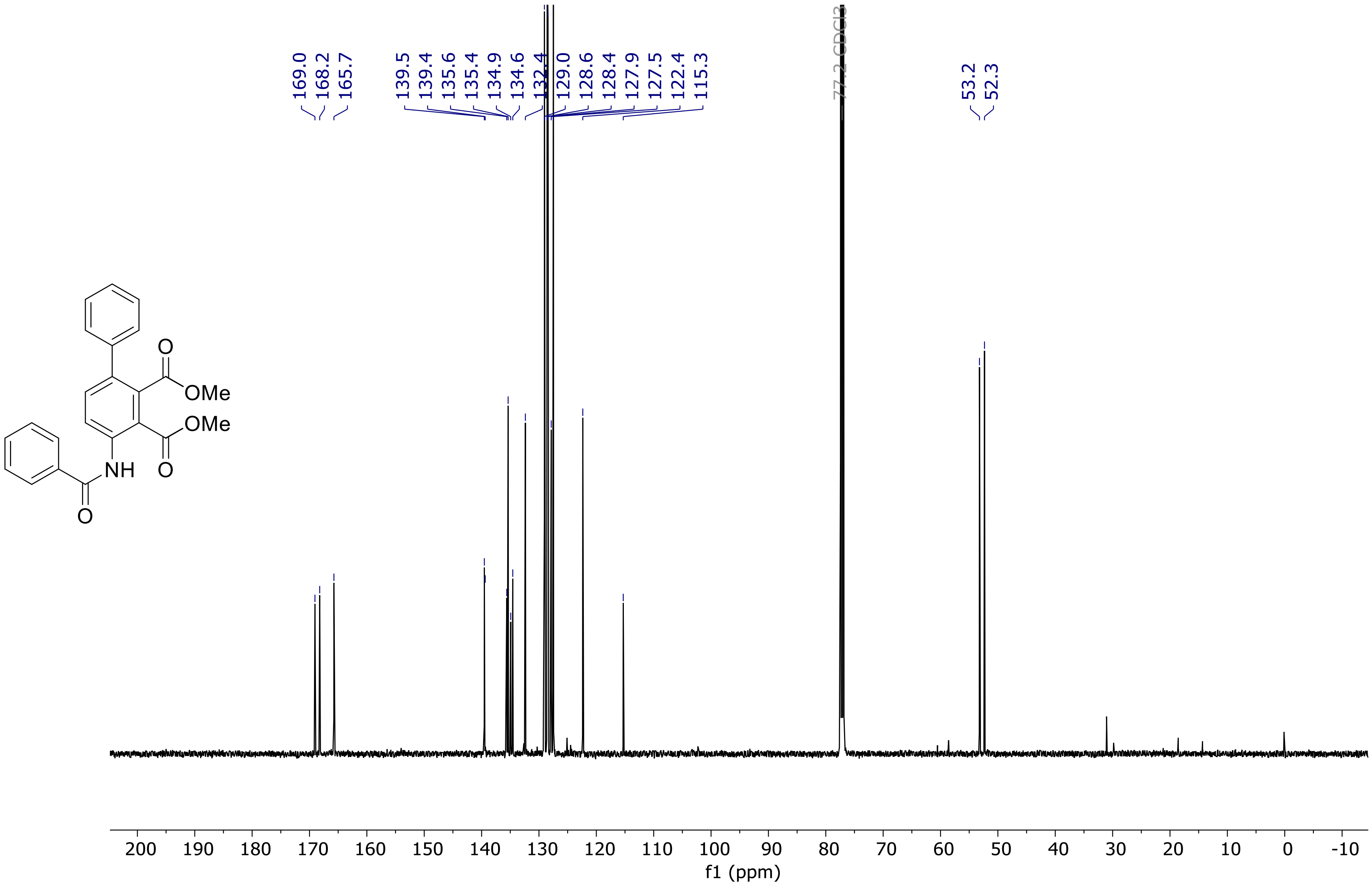

Figure S51. ^{1}H NMR Spectrum (600 MHz, $CDCl_3$) for Aromatic **2n**

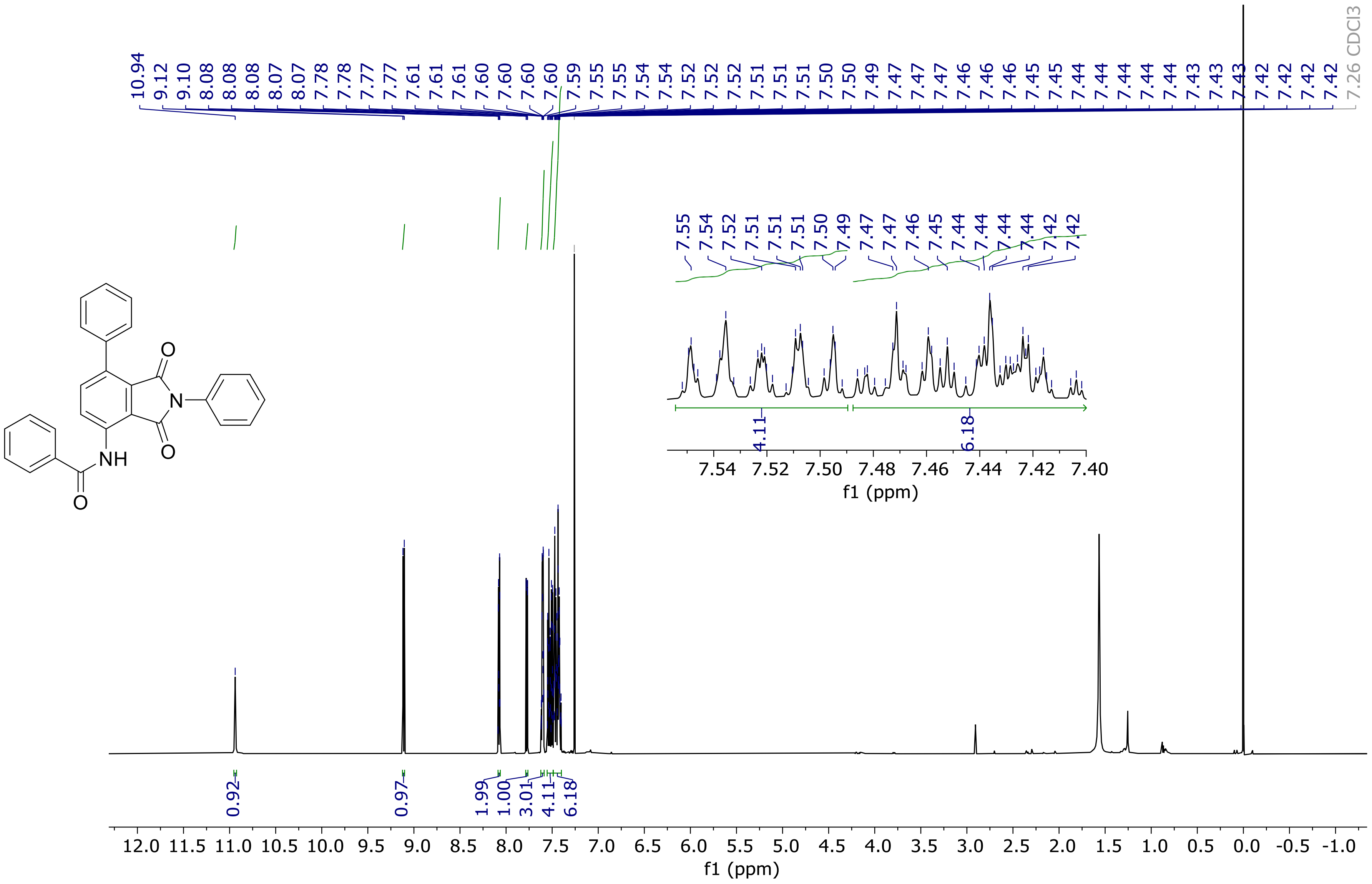

Figure S52. $^{13}C\{^1H\}$ NMR Spectrum (150 MHz, $CDCl_3$) for Aromatic **2n**

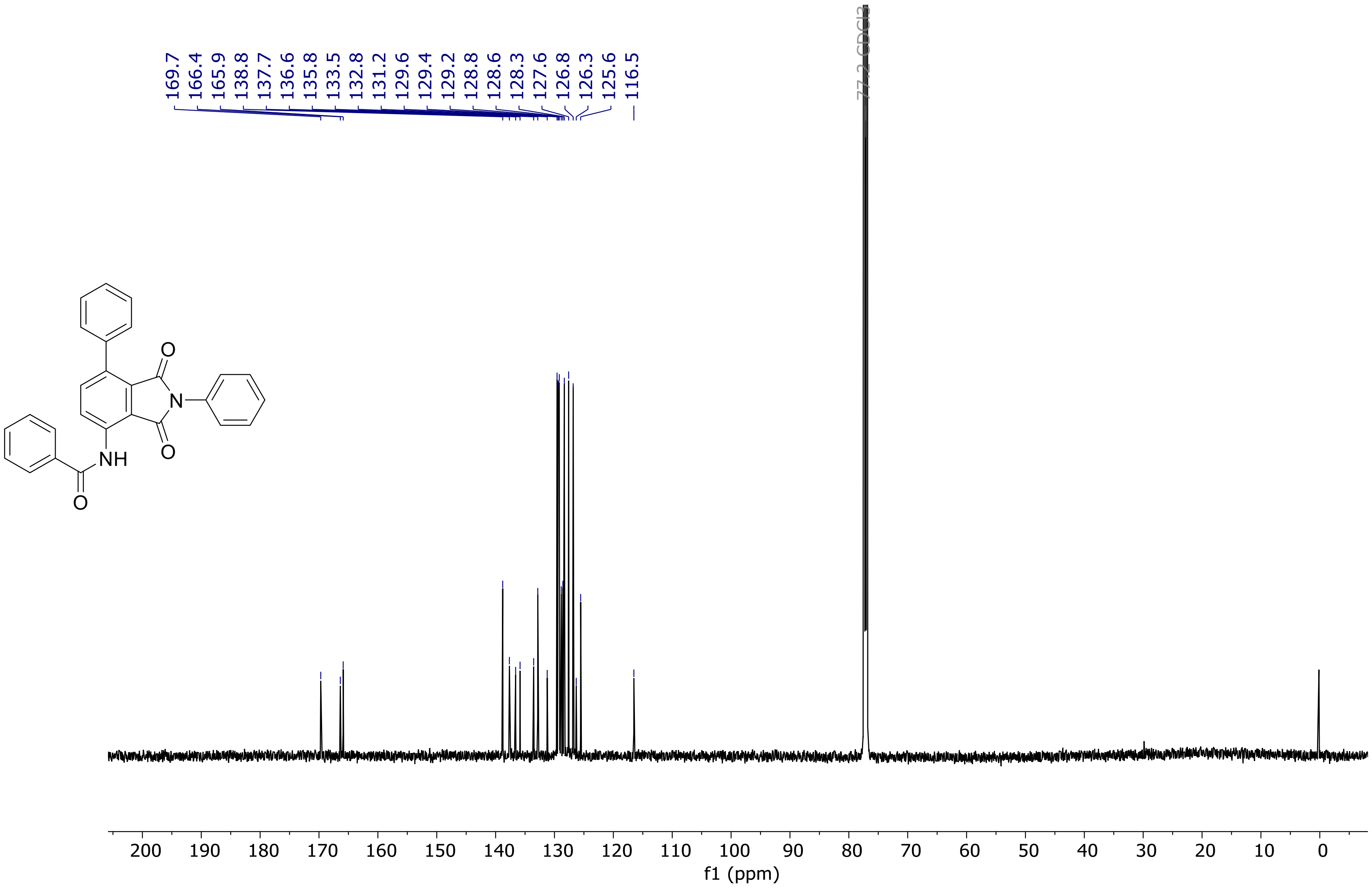

Figure S53. ^{1}H NMR Spectrum (500 MHz, $CDCl_3$) for Aromatic **2o**

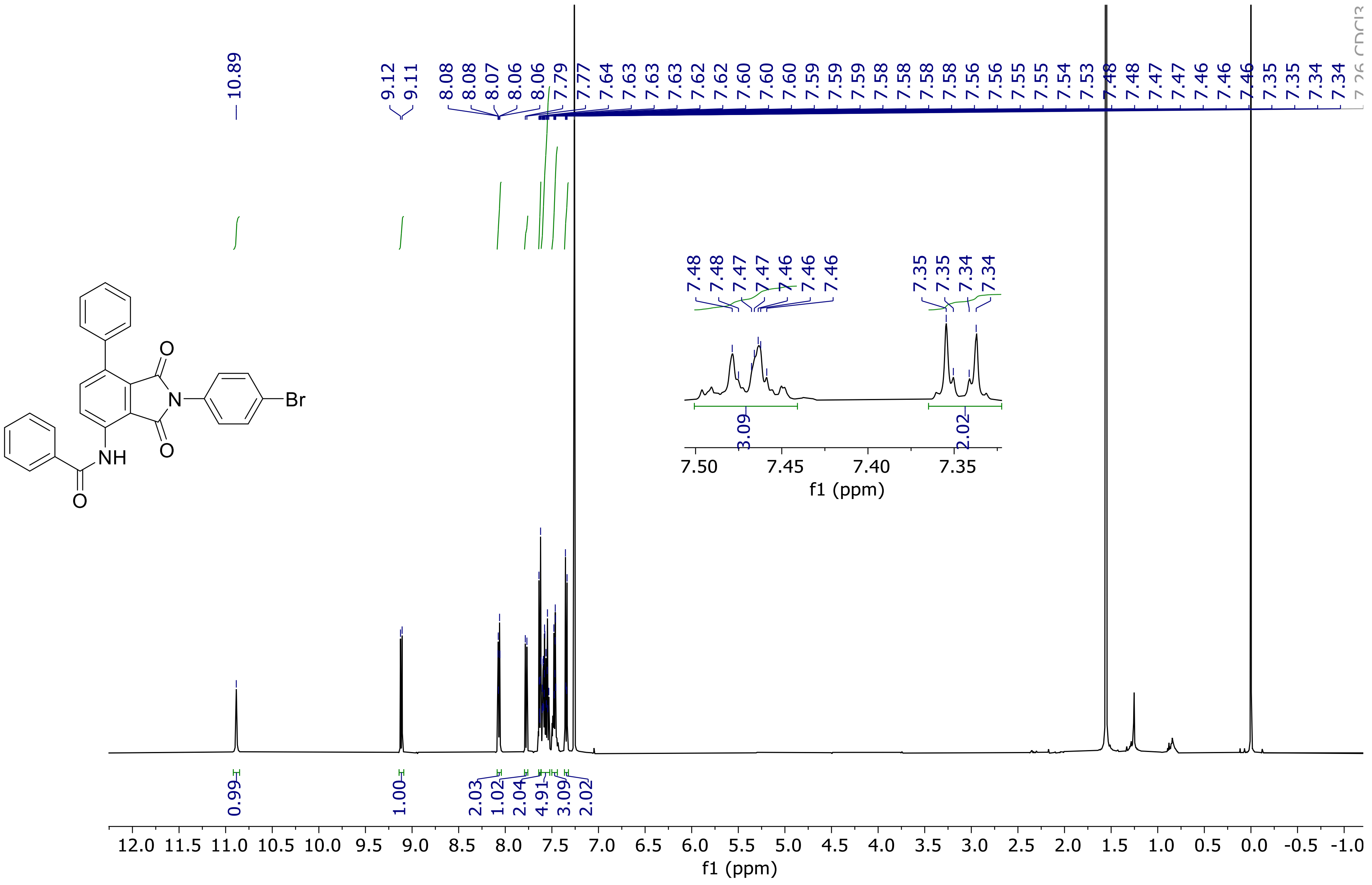

Figure S54. $^{13}C\{^1H\}$ NMR Spectrum (125 MHz, $CDCl_3$) for Aromatic **2o**

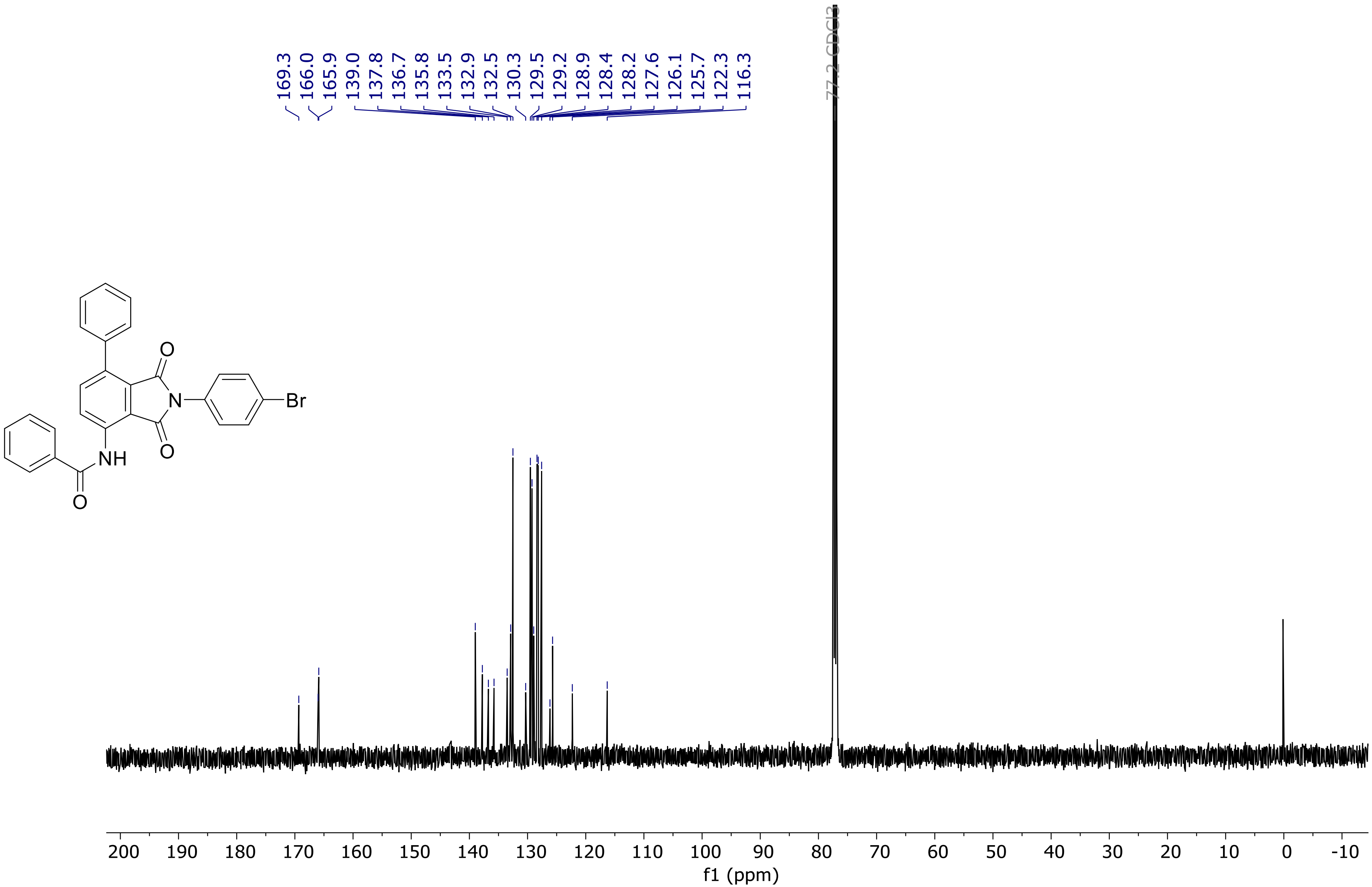

Figure S55. ^{1}H NMR Spectrum (500 MHz, $CDCl_3$) for Aromatic **2p**

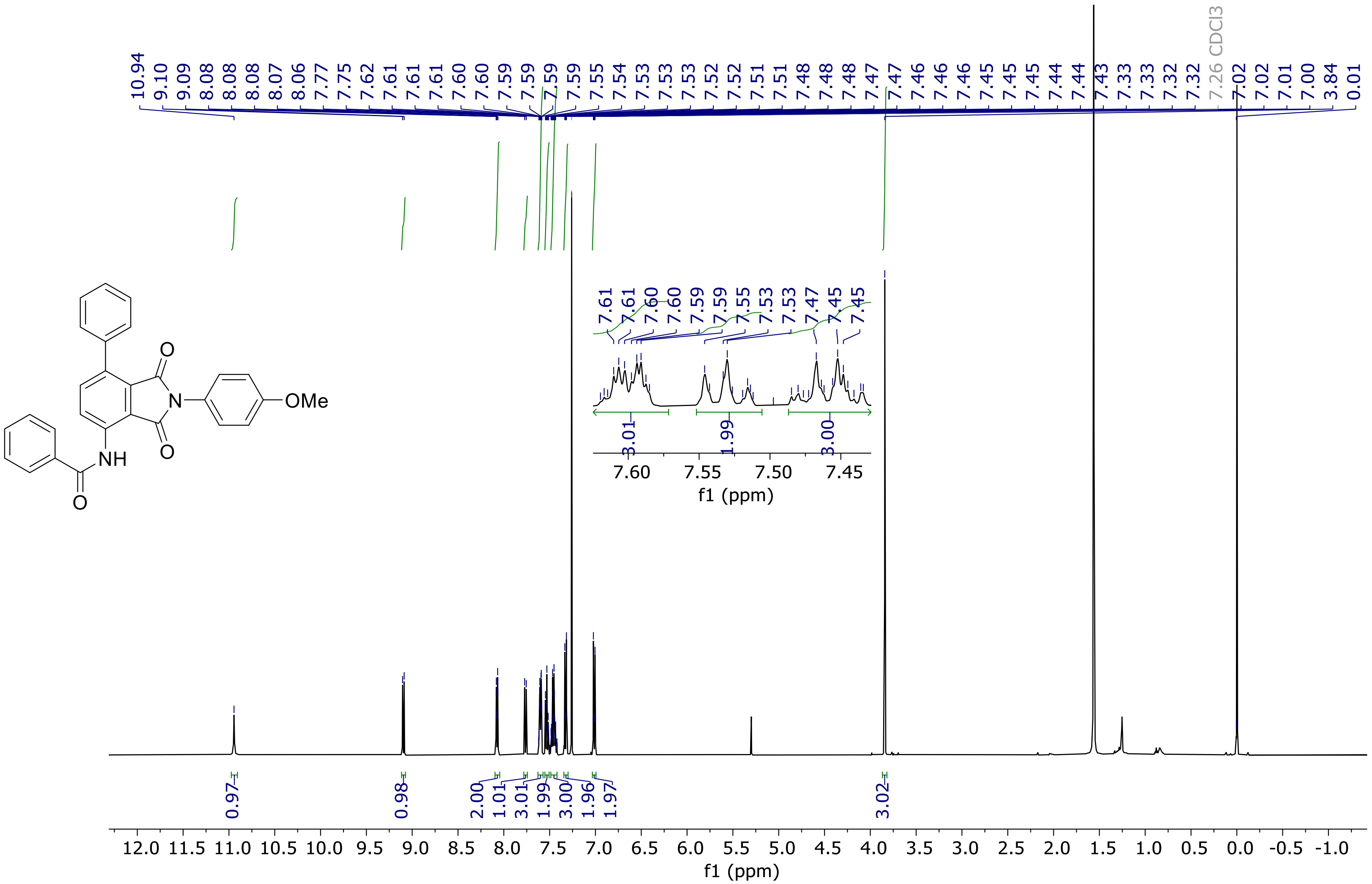

Figure S56. $^{13}C\{^1H\}$ NMR Spectrum (125 MHz, $CDCl_3$) for Aromatic **2p**

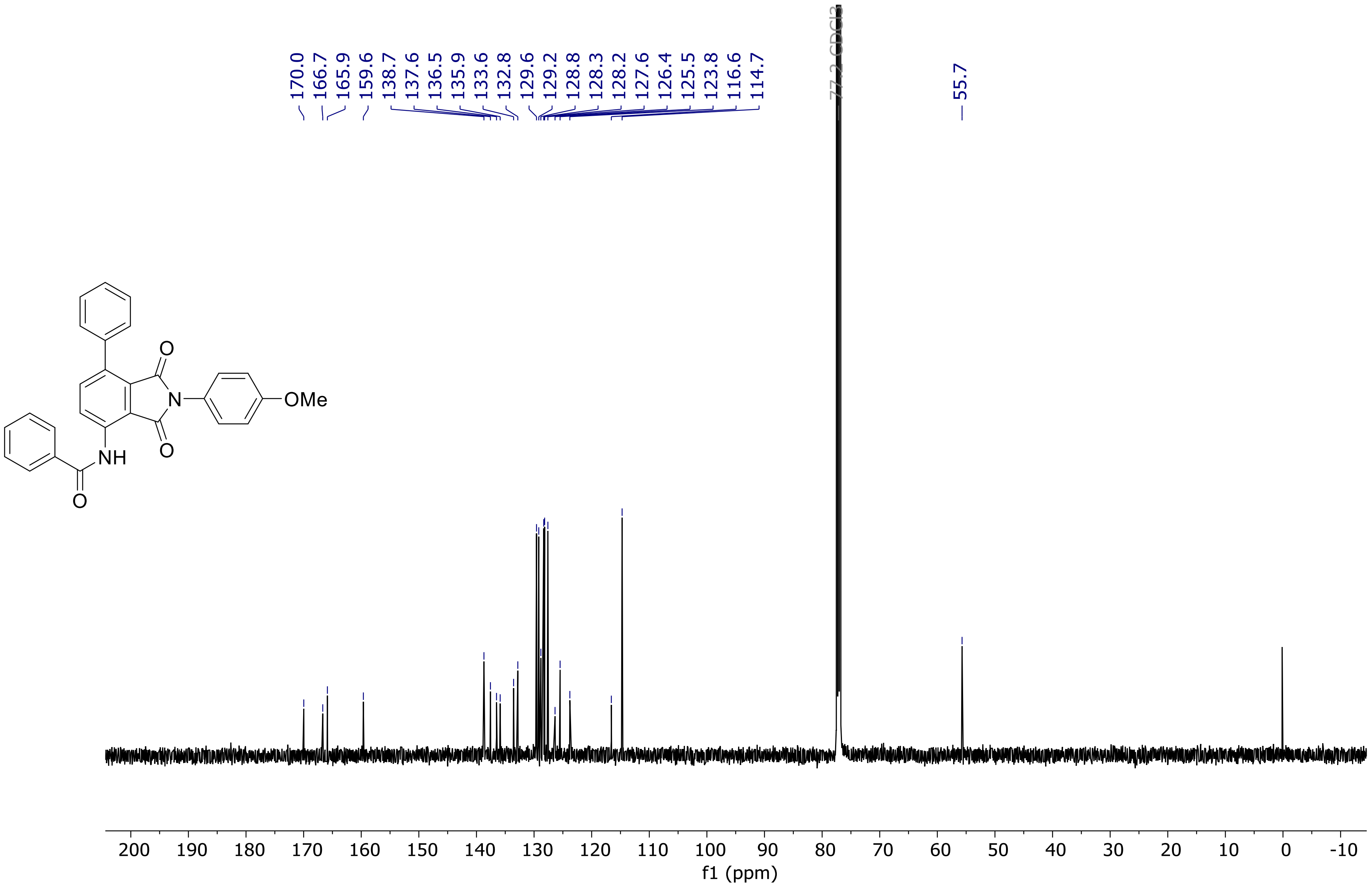

Figure S57. ^{1}H NMR Spectrum (600 MHz, $CDCl_3$) for Aromatic **2q**

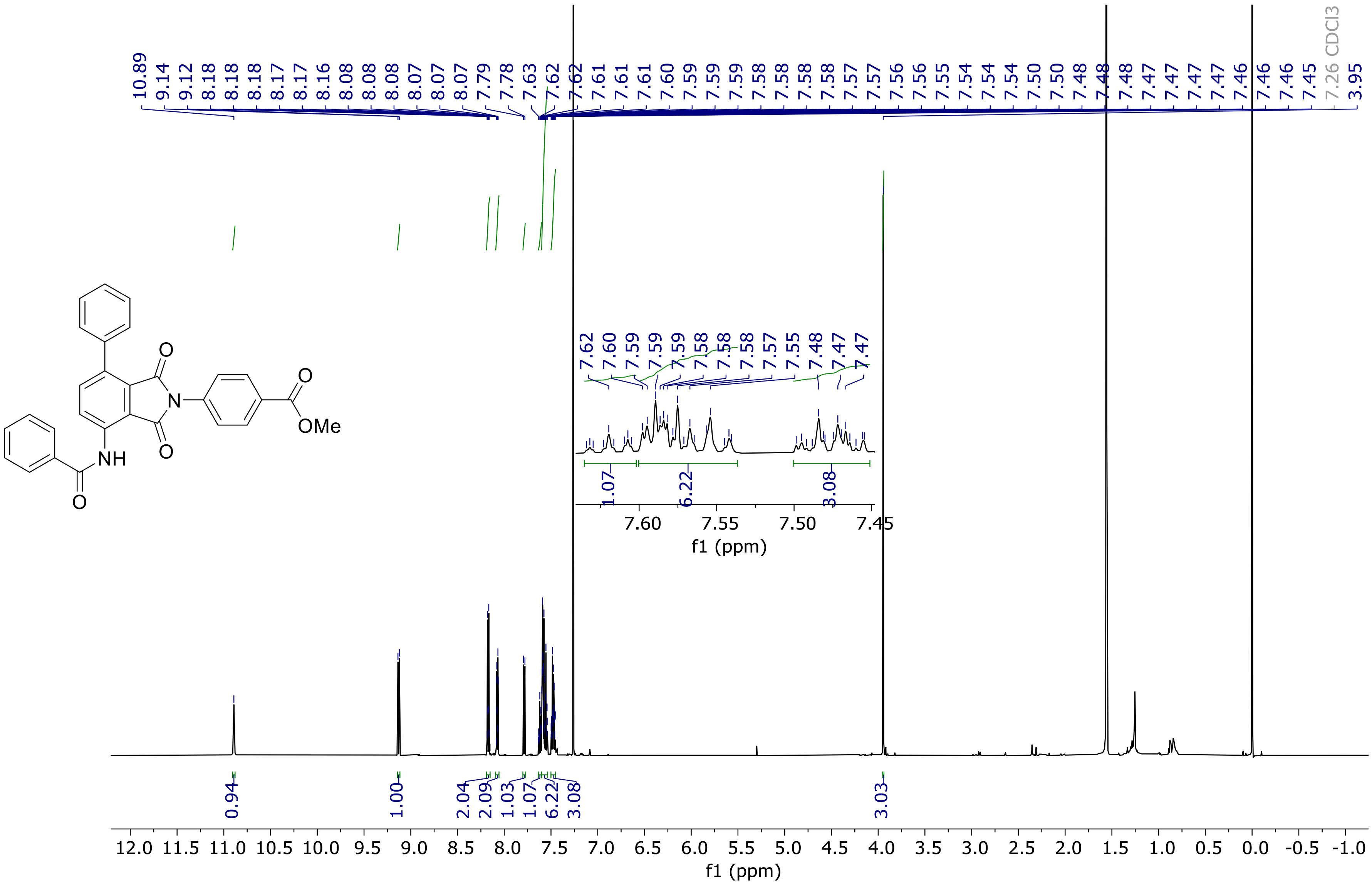

Figure S58. $^{13}C\{^{1}H\}$ NMR Spectrum (150 MHz, $CDCl_3$) for Aromatic **2q**

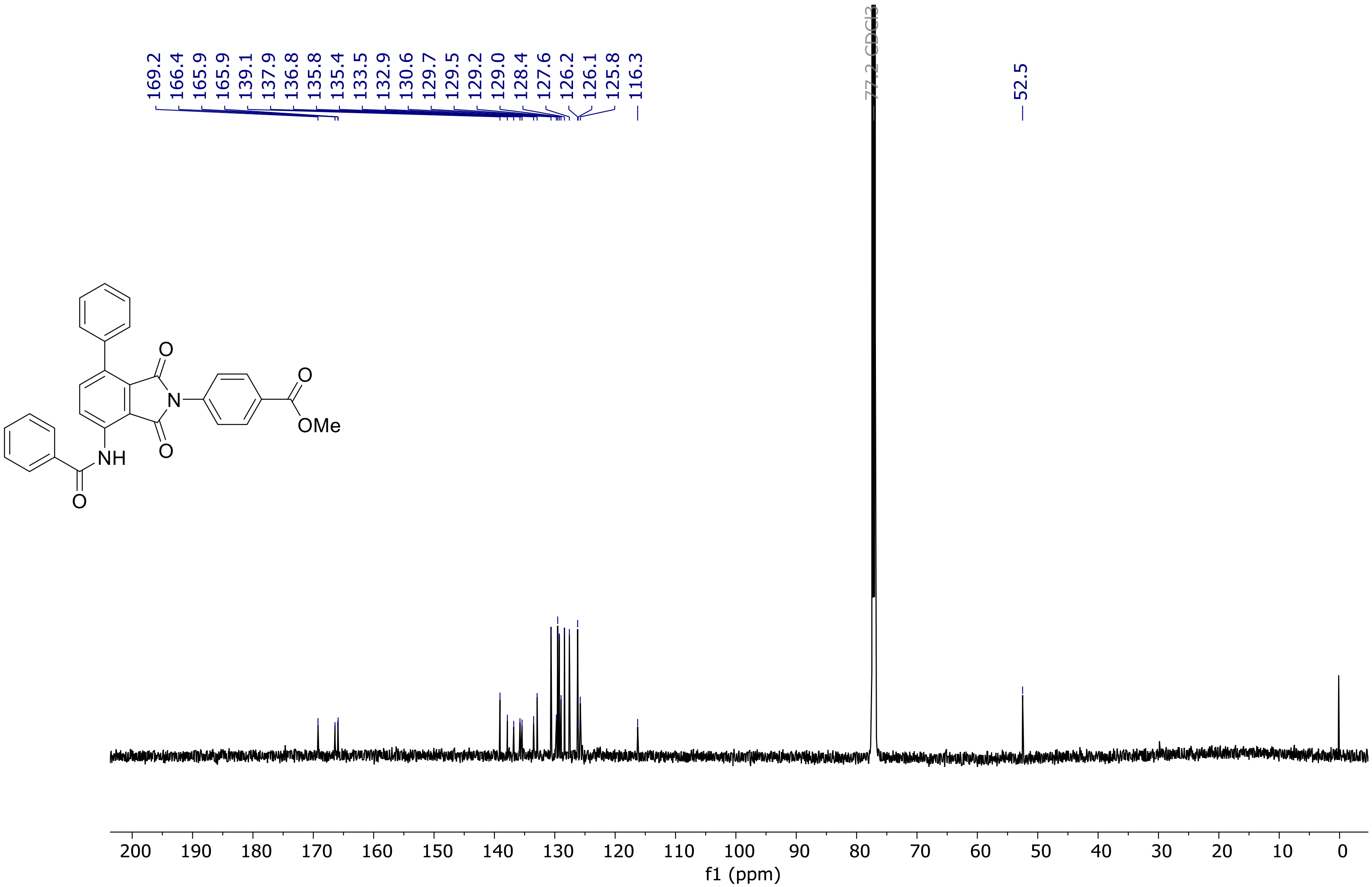

Figure S59. ^{1}H NMR Spectrum (500 MHz, $CDCl_3$) for Aromatic **2r**

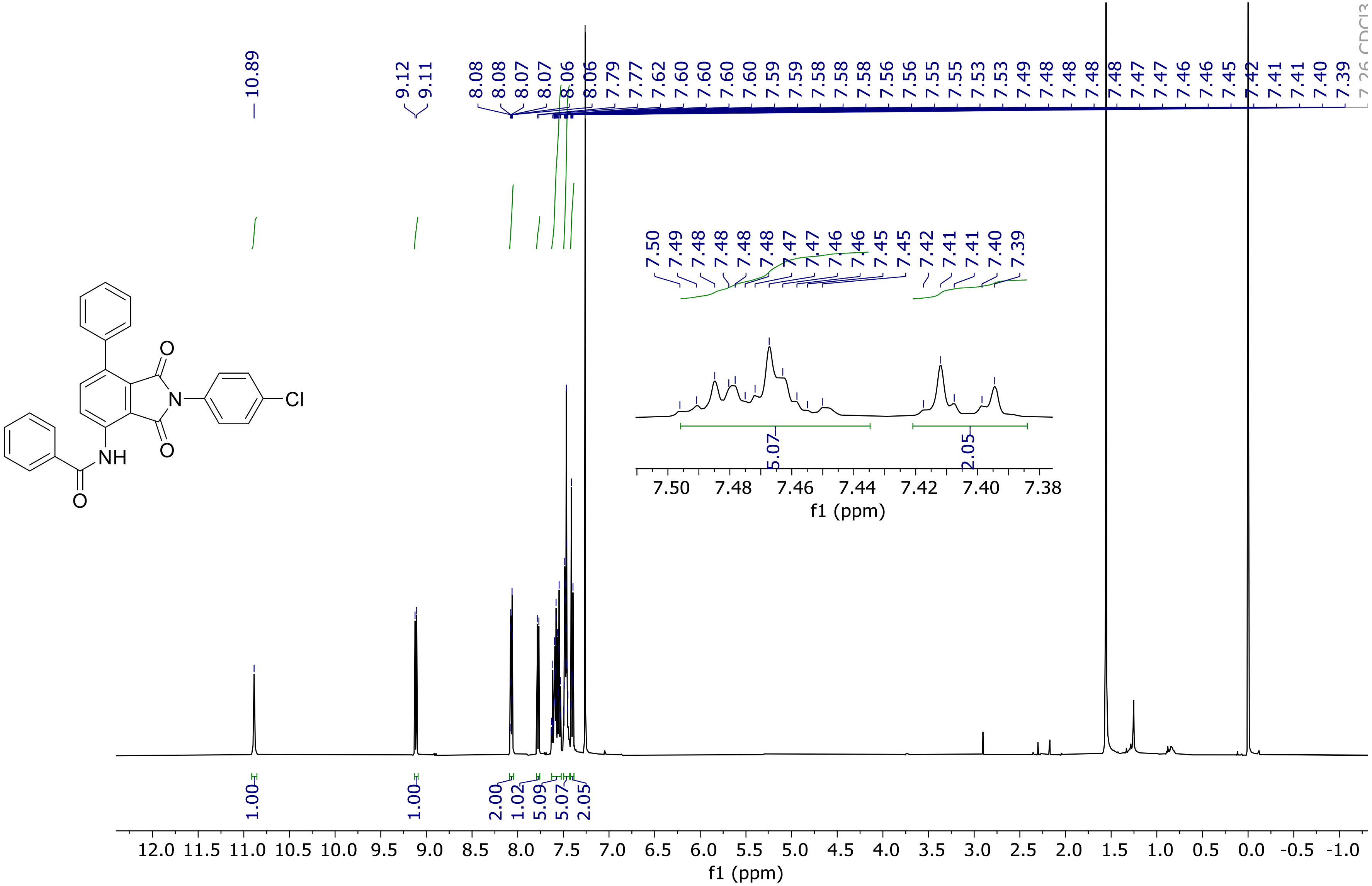

Figure S60. $^{13}C\{^1H\}$ NMR Spectrum (125 MHz, $CDCl_3$) for Aromatic **2r**

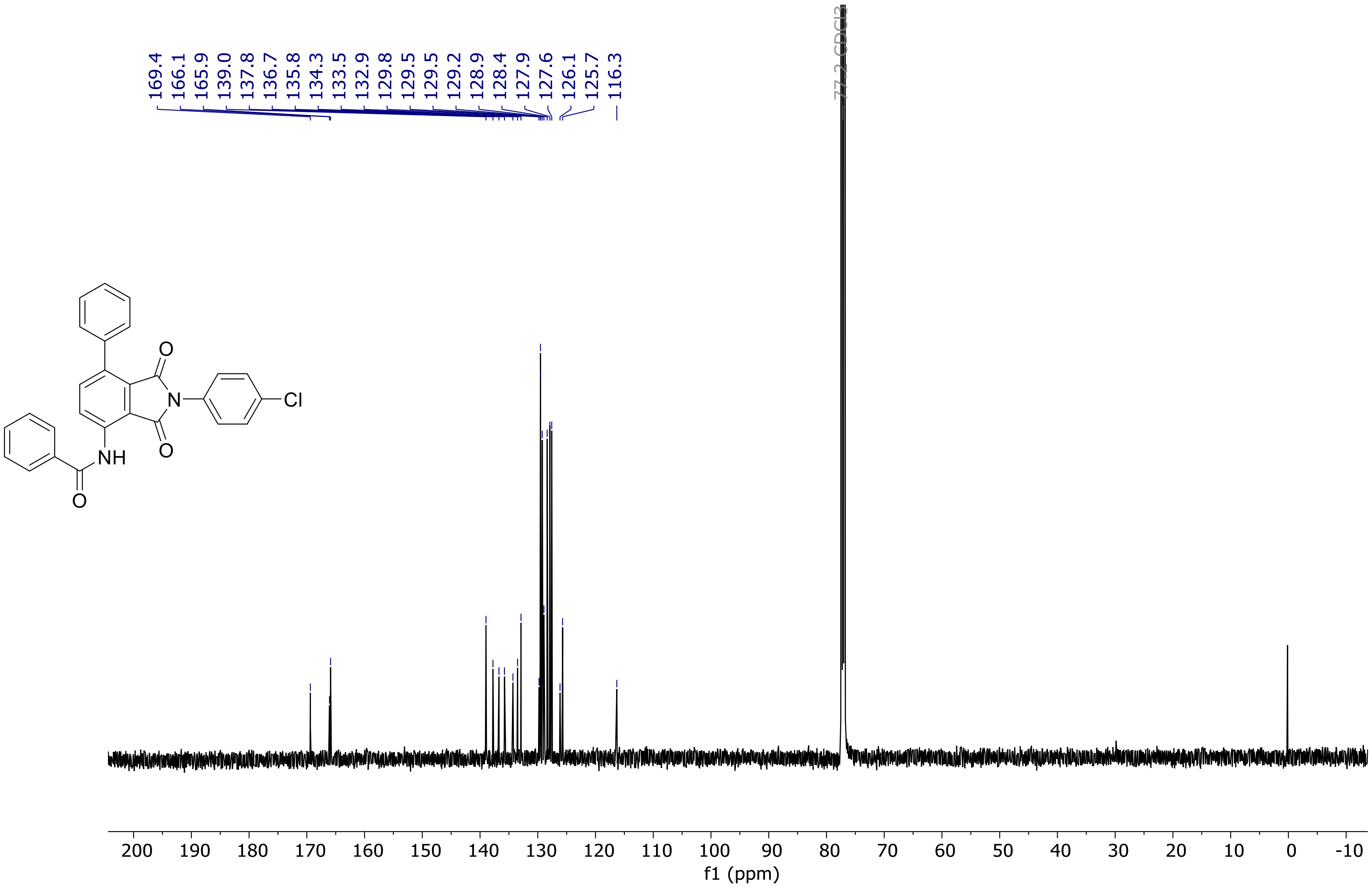

Figure S61. ^{1}H NMR Spectrum (500 MHz, $CDCl_3$) for Aromatic **2s**

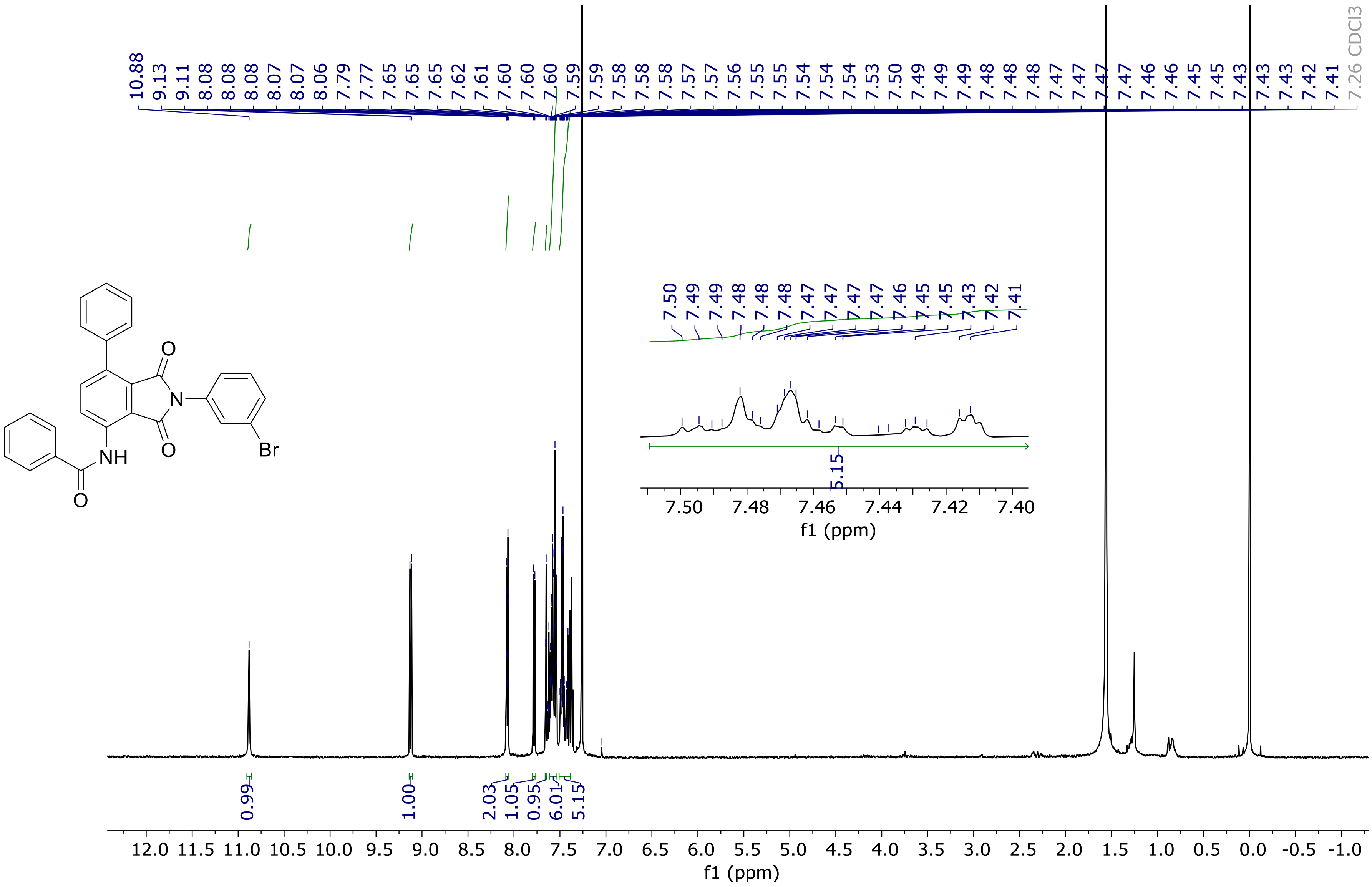

Figure S62. $^{13}C\{^1H\}$ NMR Spectrum (125 MHz, $CDCl_3$) for Aromatic **2s**

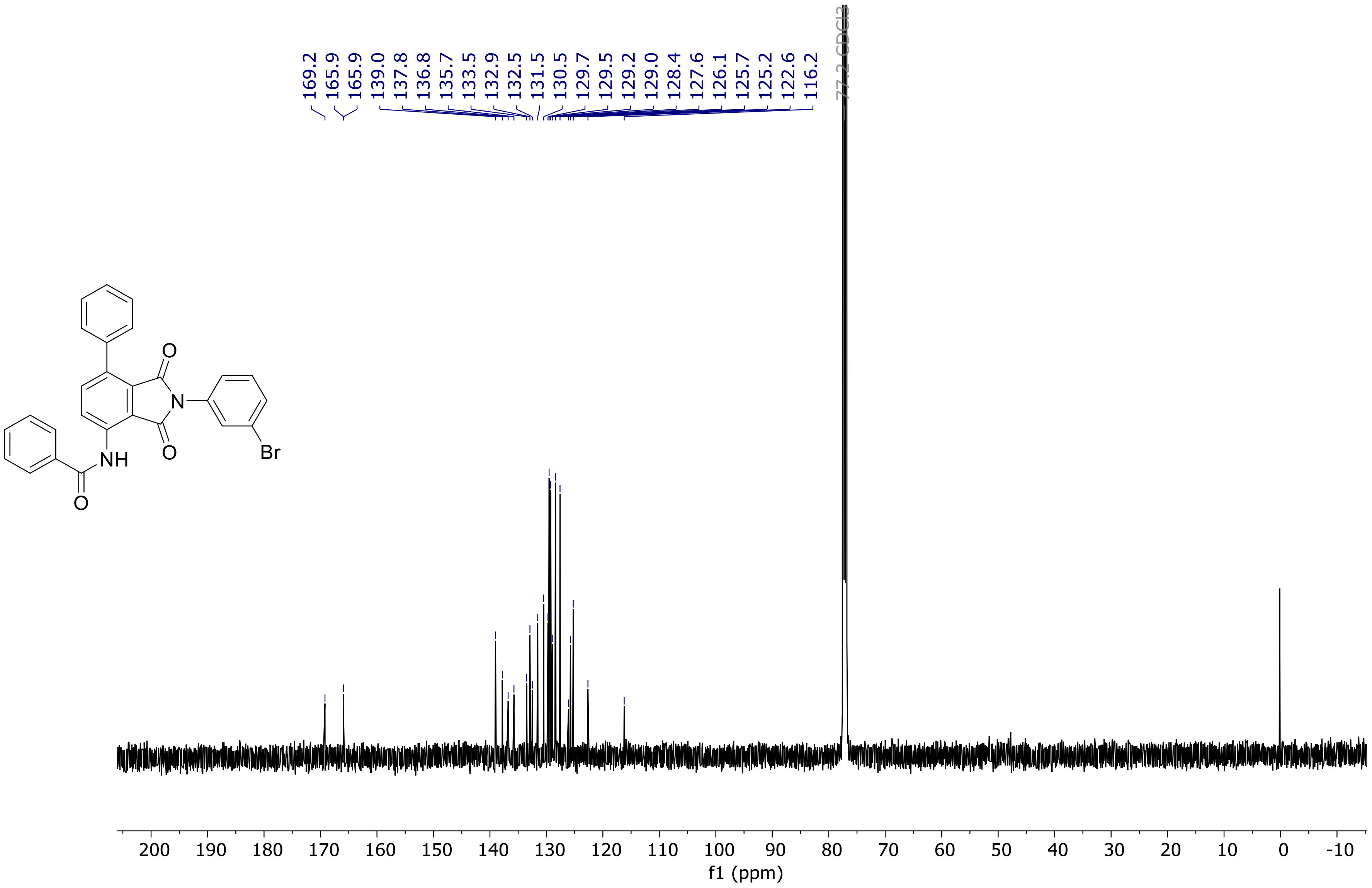

Figure S63. ^{1}H NMR Spectrum (500 MHz, $CDCl_3$) for Aromatic **2t**

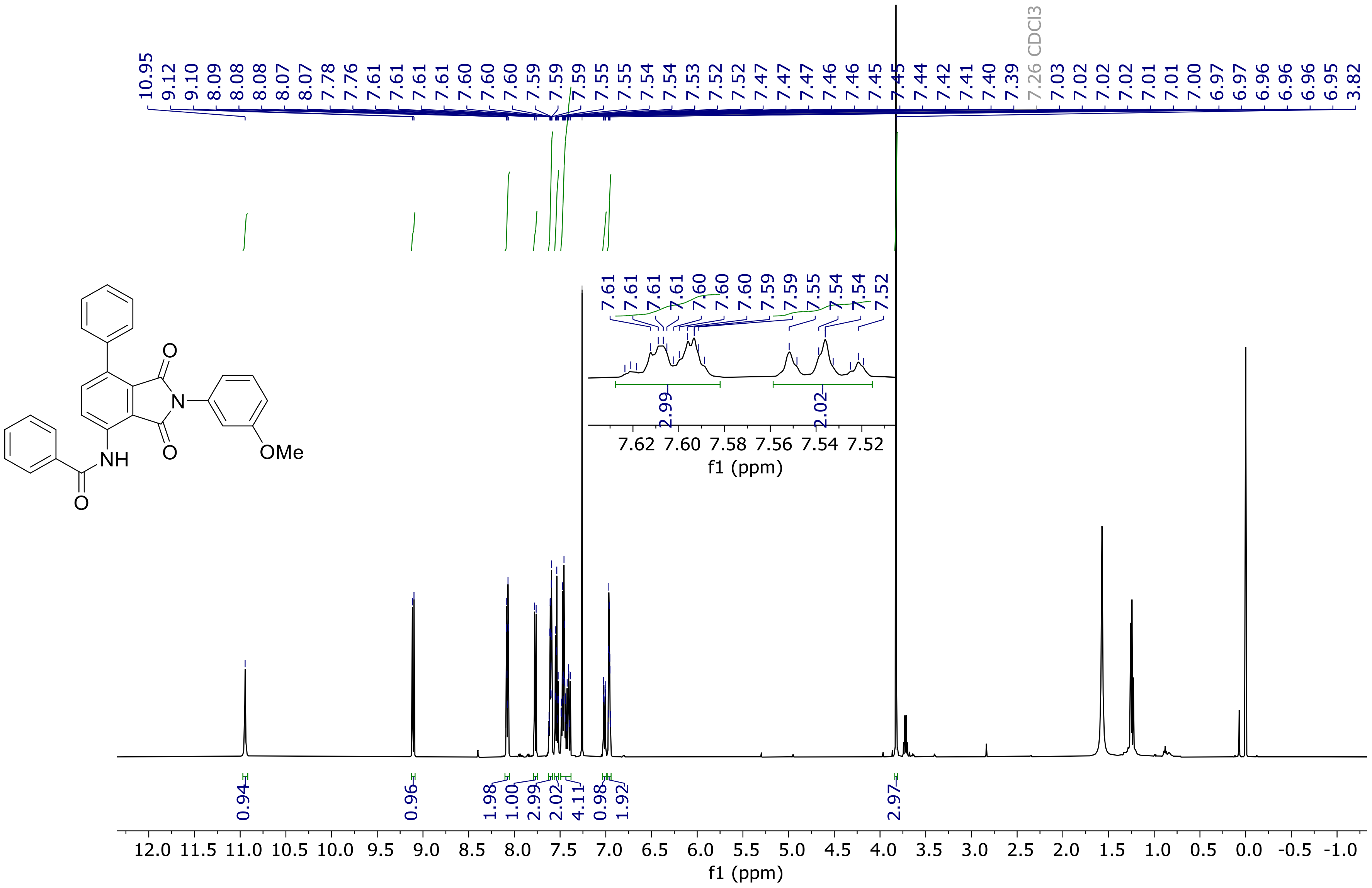

Figure S64. $^{13}C\{^{1}H\}$ NMR Spectrum (125 MHz, $CDCl_3$) for Aromatic **2t**

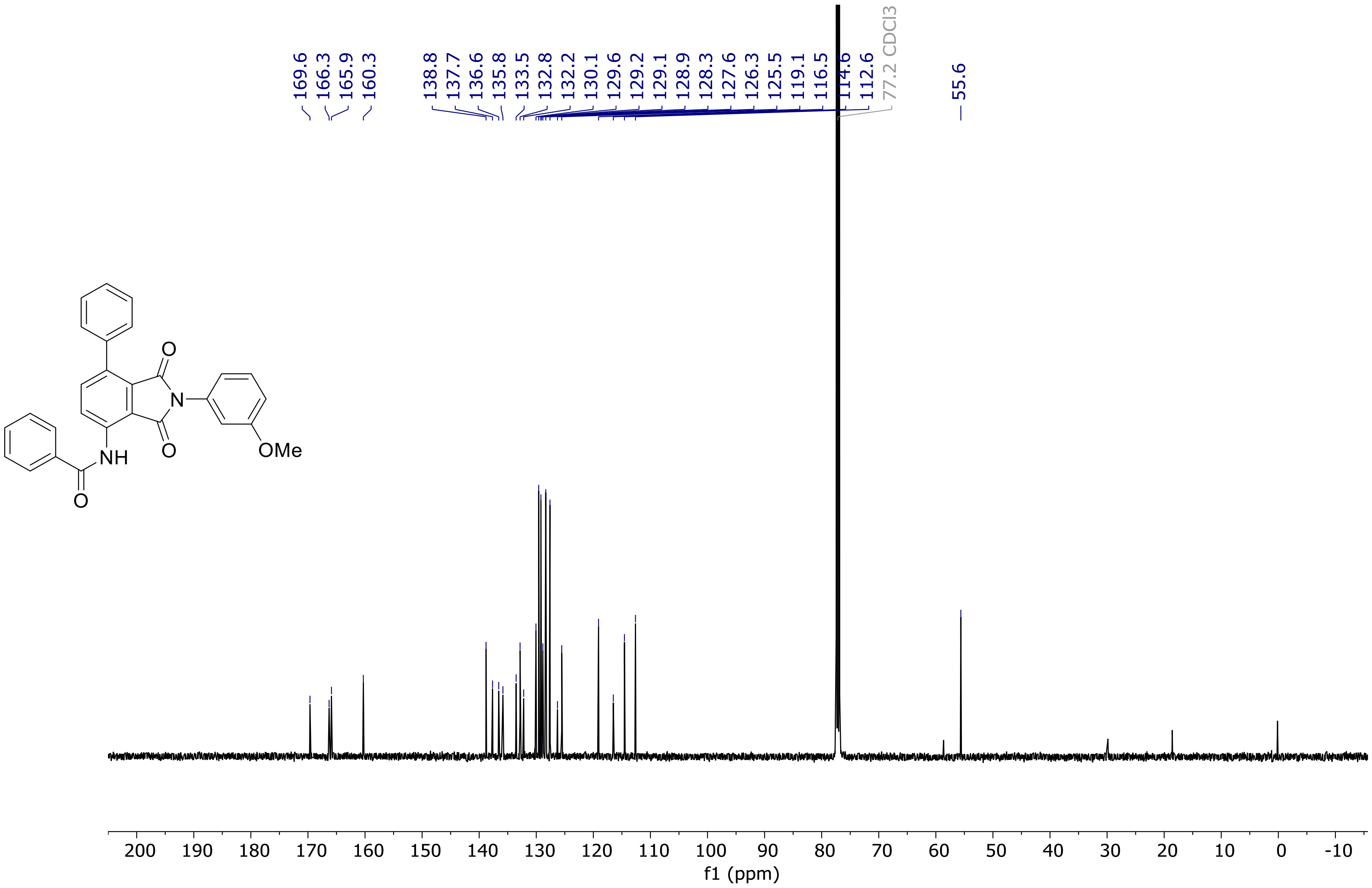

Figure S65. ^{1}H NMR Spectrum (500 MHz, $CDCl_3$) for Aromatic **2u**

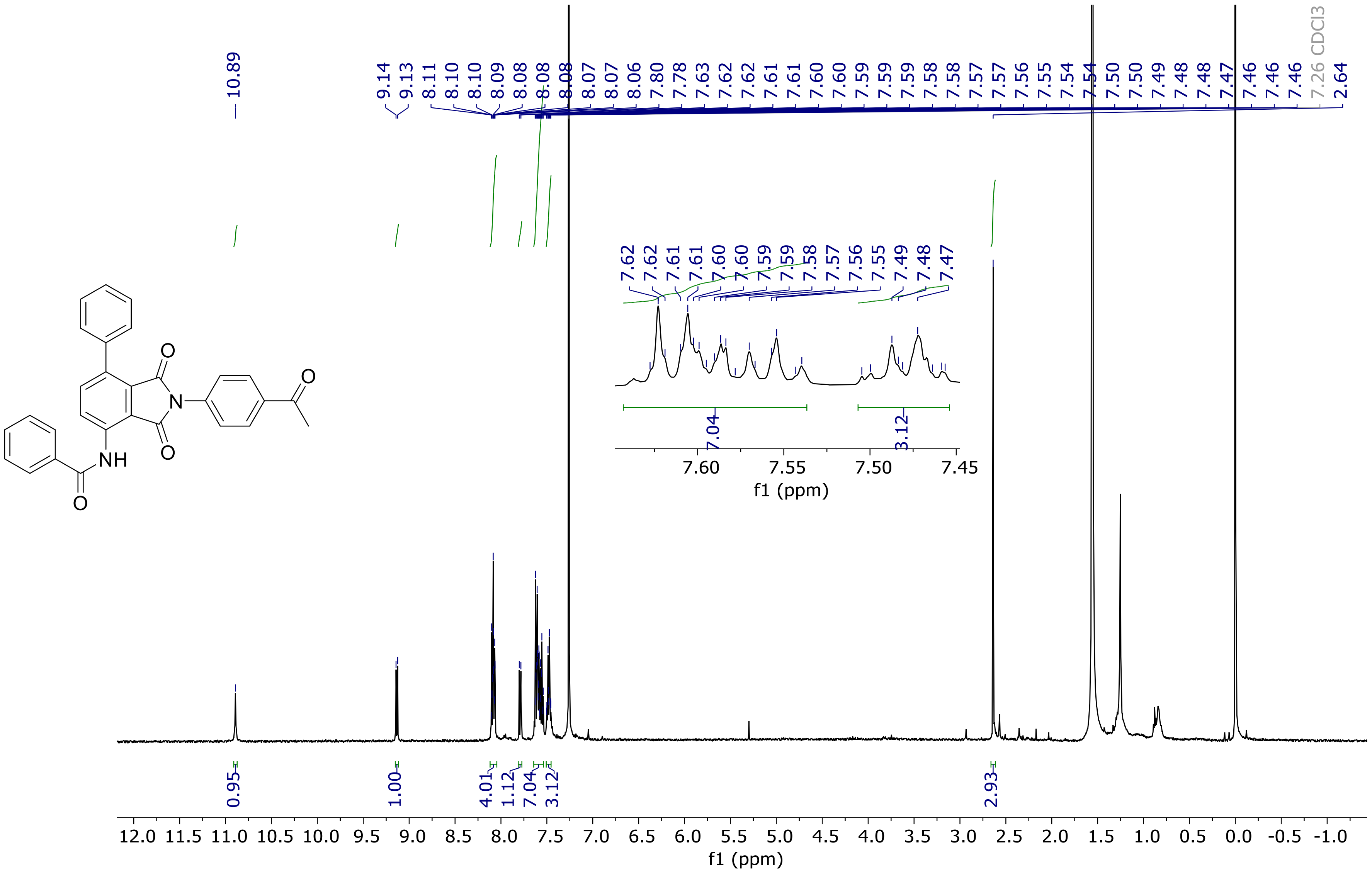

Figure S66. $^{13}C\{^{1}H\}$ NMR Spectrum (125 MHz, $CDCl_3$) for Aromatic **2u**

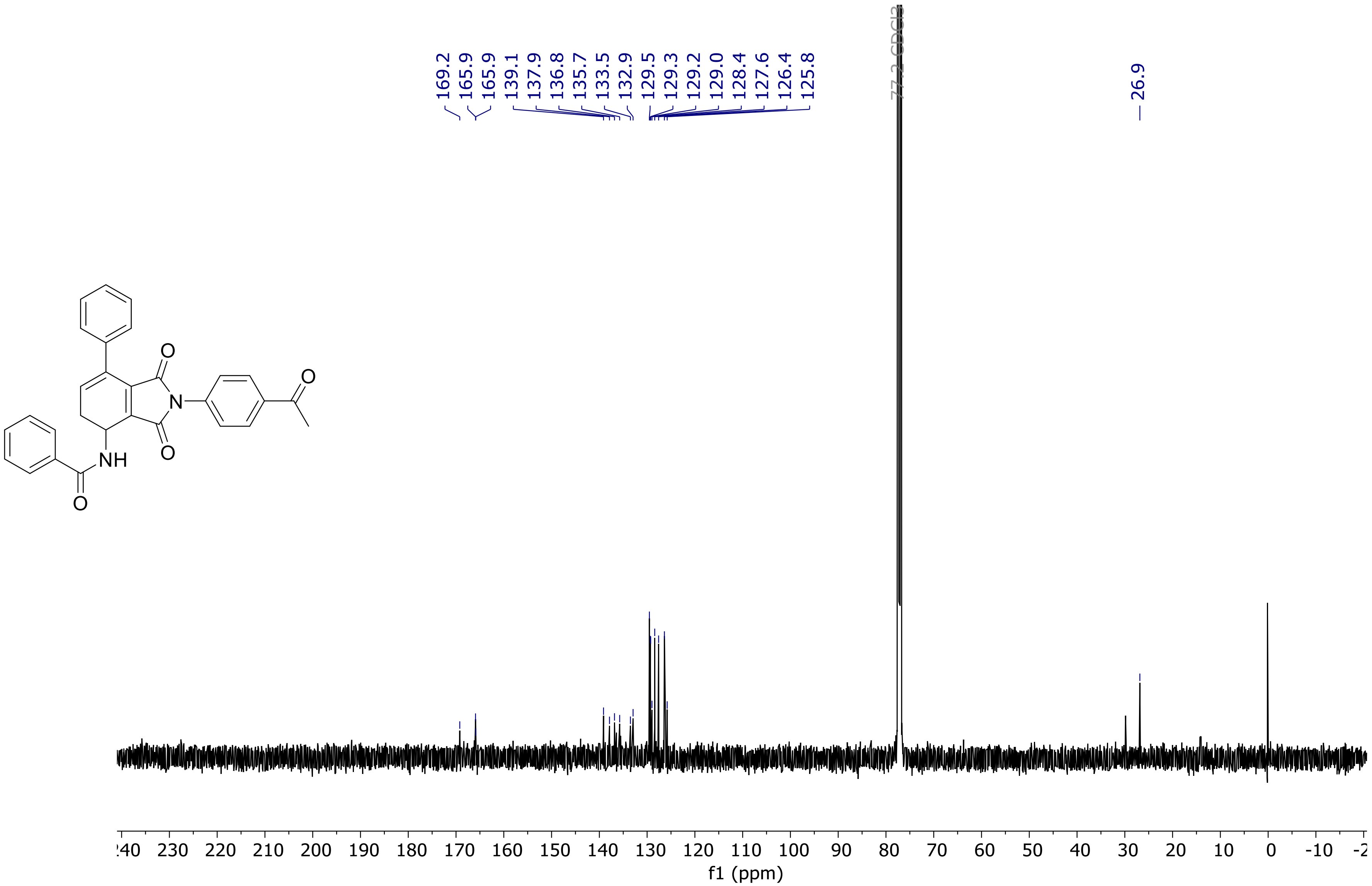

Figure S67. ^{1}H NMR Spectrum (600 MHz, $CDCl_3$) for Aromatic **2v**

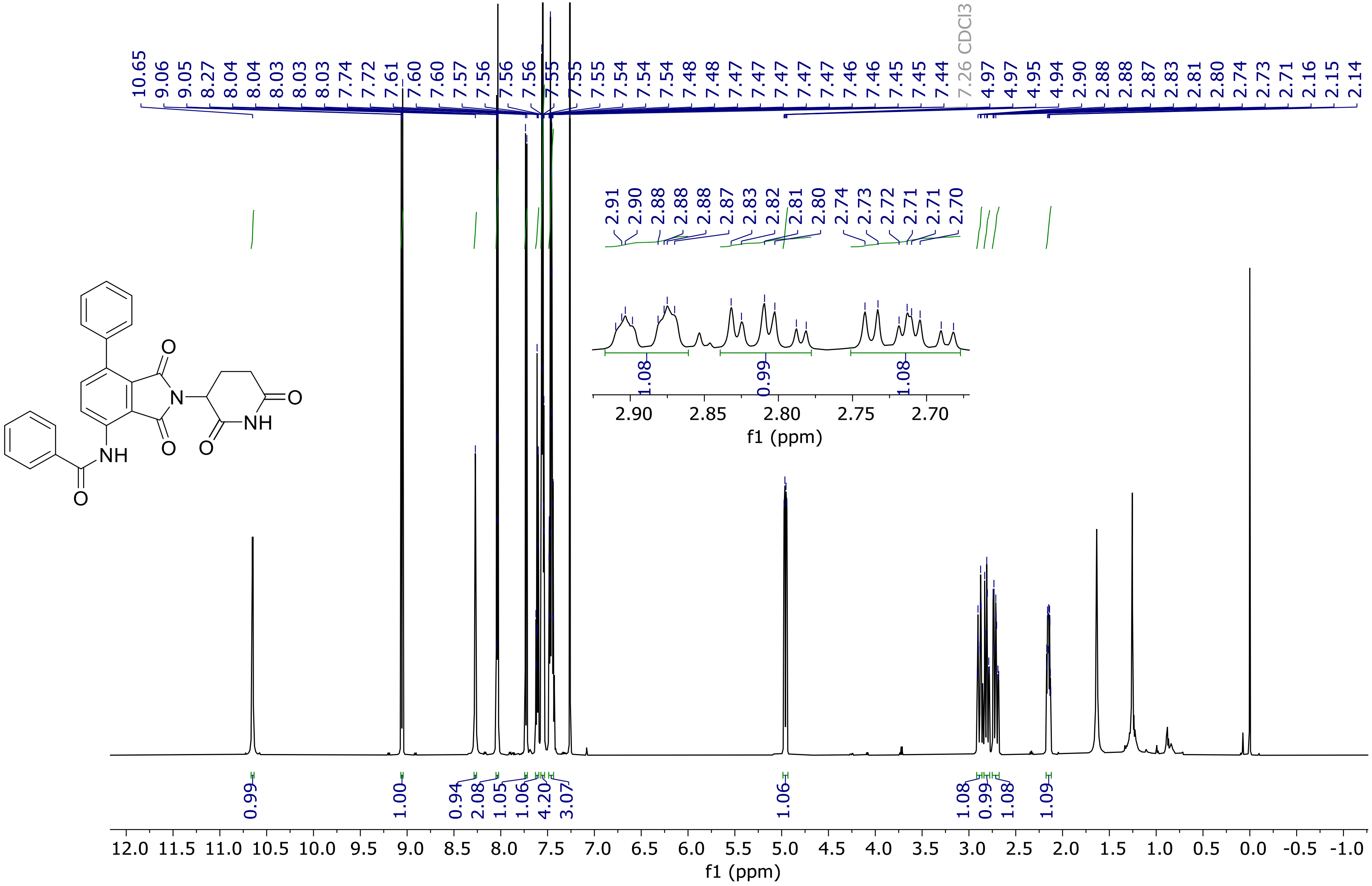

Figure S68. $^{13}C\{^{1}H\}$ NMR Spectrum (150 MHz, CDCl$_3$) for Aromatic **2v**

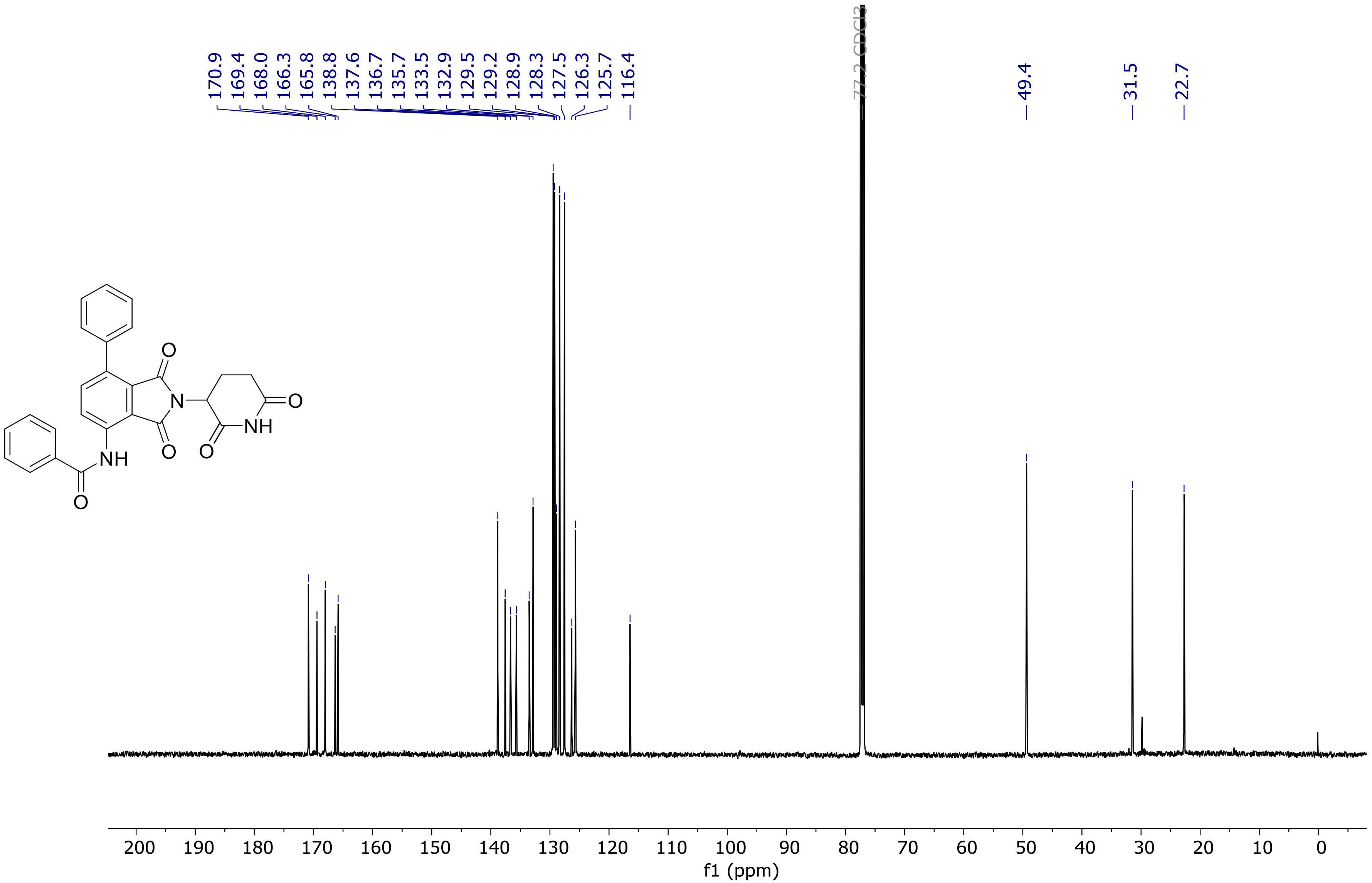

Figure S69. ^{1}H NMR Spectrum (600 MHz, $CDCl_3$) for Aromatic **2w**

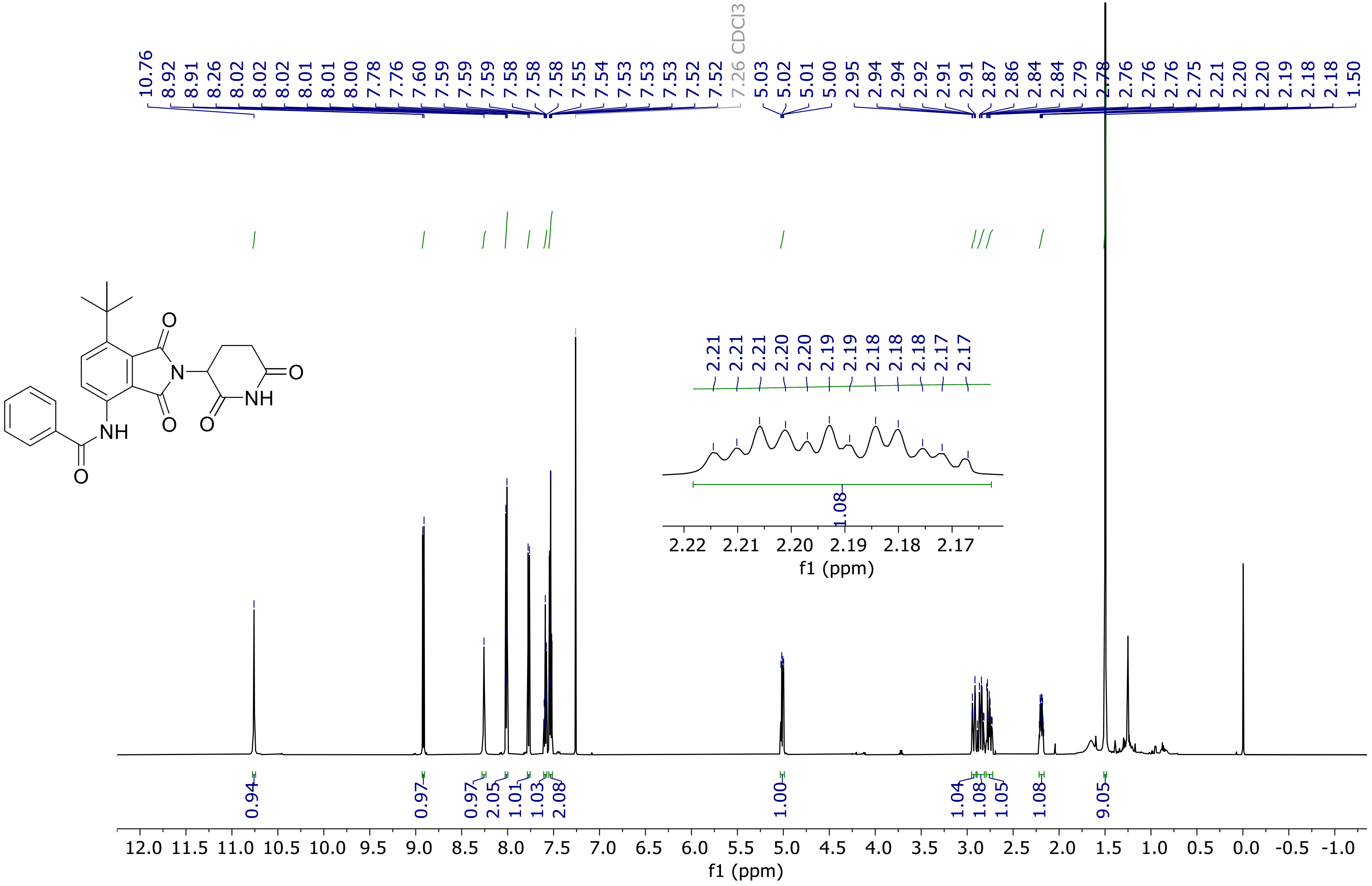

Figure S70. $^{13}C\{^{1}H\}$ NMR Spectrum (150 MHz, $CDCl_3$) for Aromatic **2w**

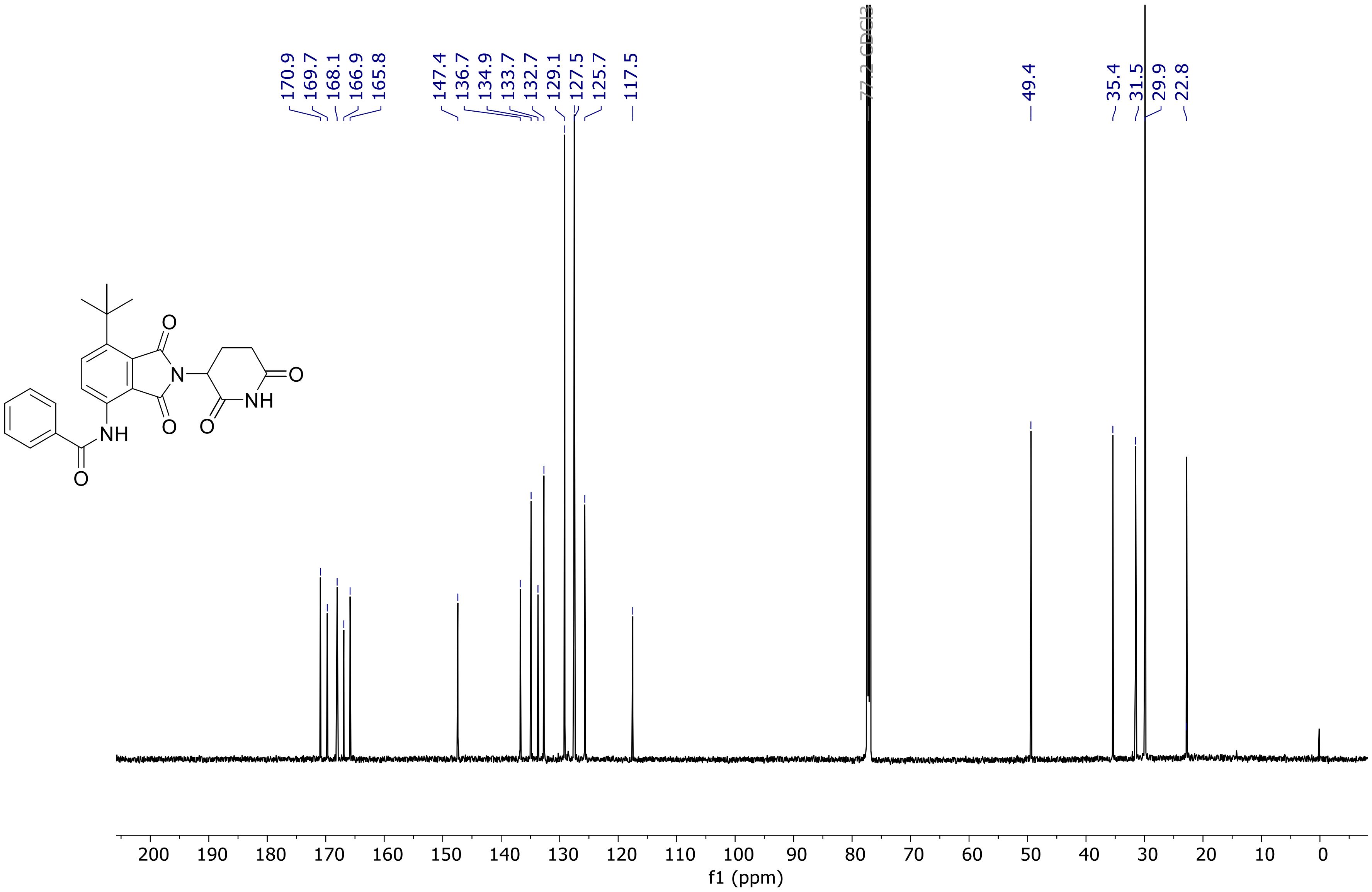

Figure S71. ^{1}H NMR Spectrum (600 MHz, $CDCl_3$) for Aromatic **2w'**

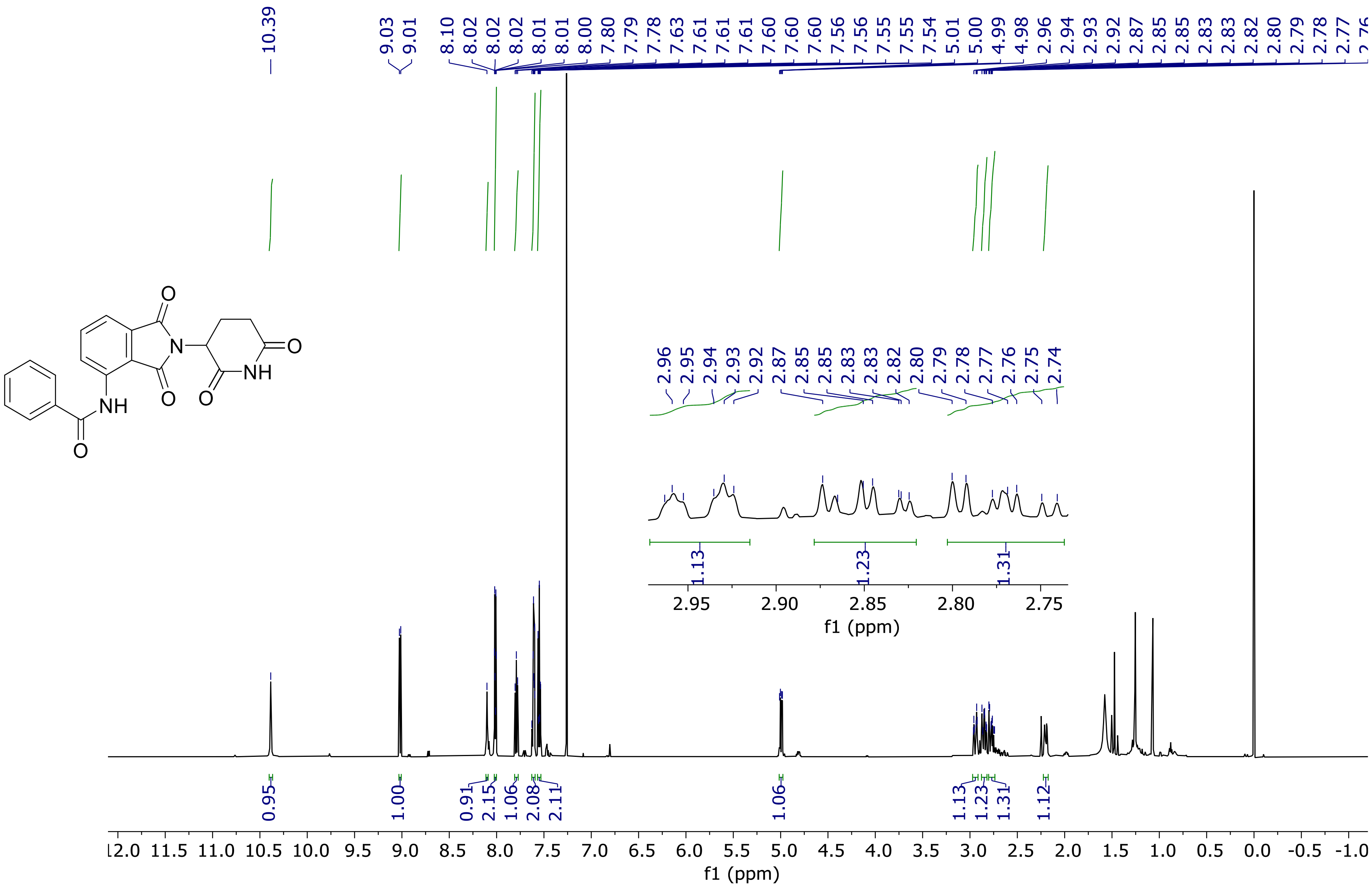

Figure S72. $^{13}C\{^1H\}$ NMR Spectrum (150 MHz, $CDCl_3$) for Aromatic **2w'**

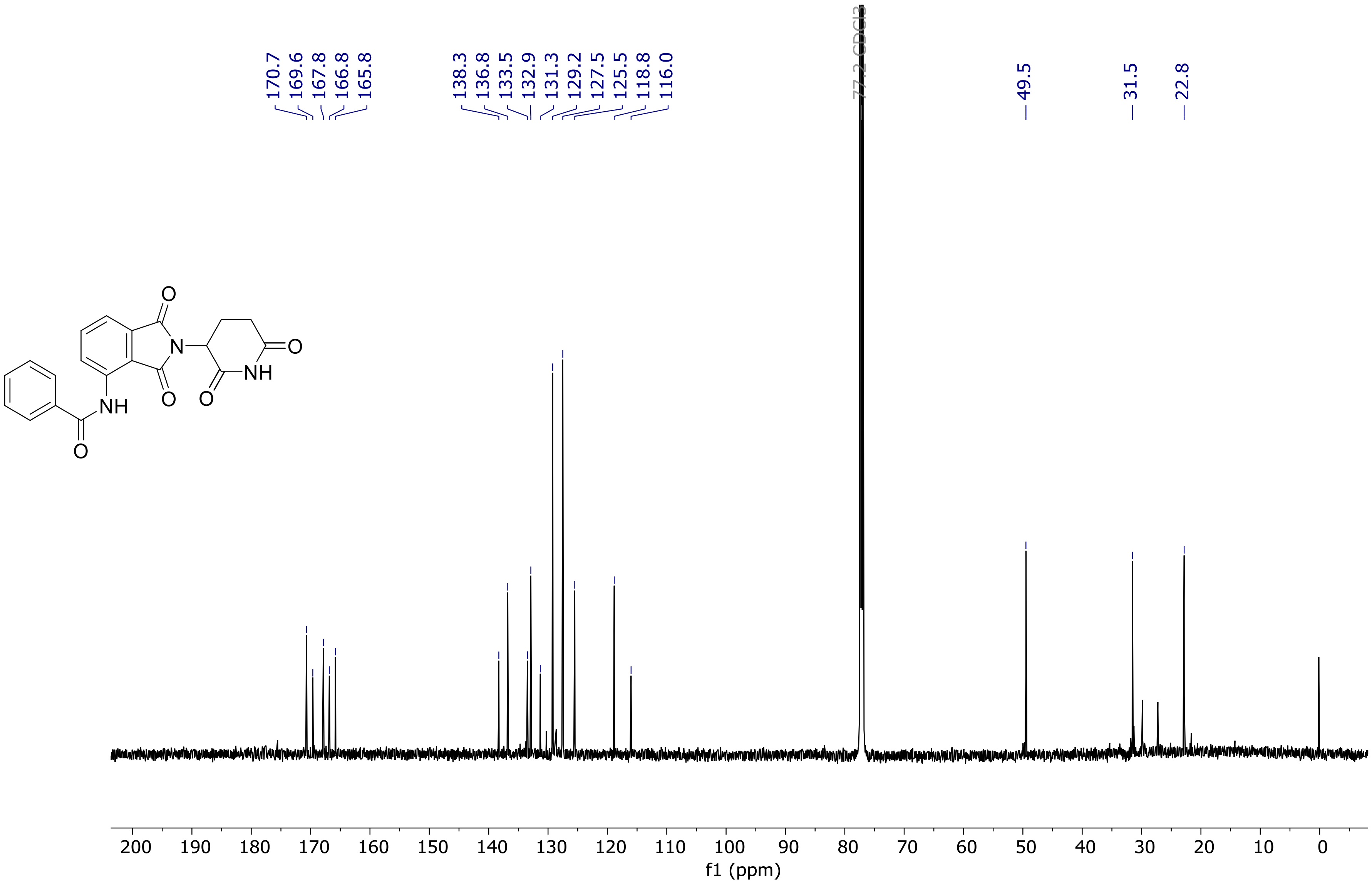

Figure S73. ^{1}H NMR Spectrum (600 MHz, $CDCl_3$) for Adduct **3a**

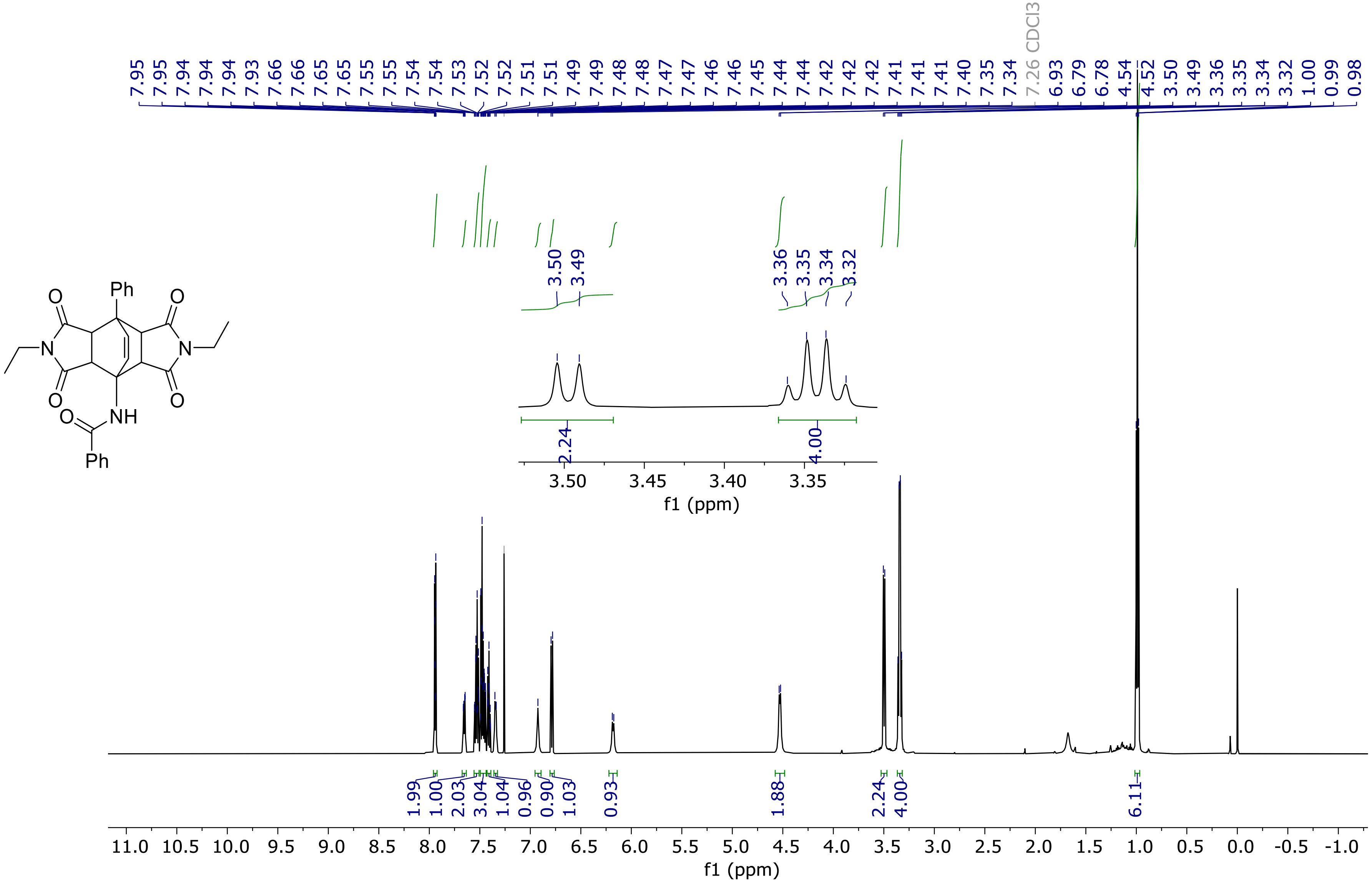

Figure S74. $^{13}C\{^{1}H\}$ NMR Spectrum (150 MHz, $CDCl_3$) for Adduct **3a**

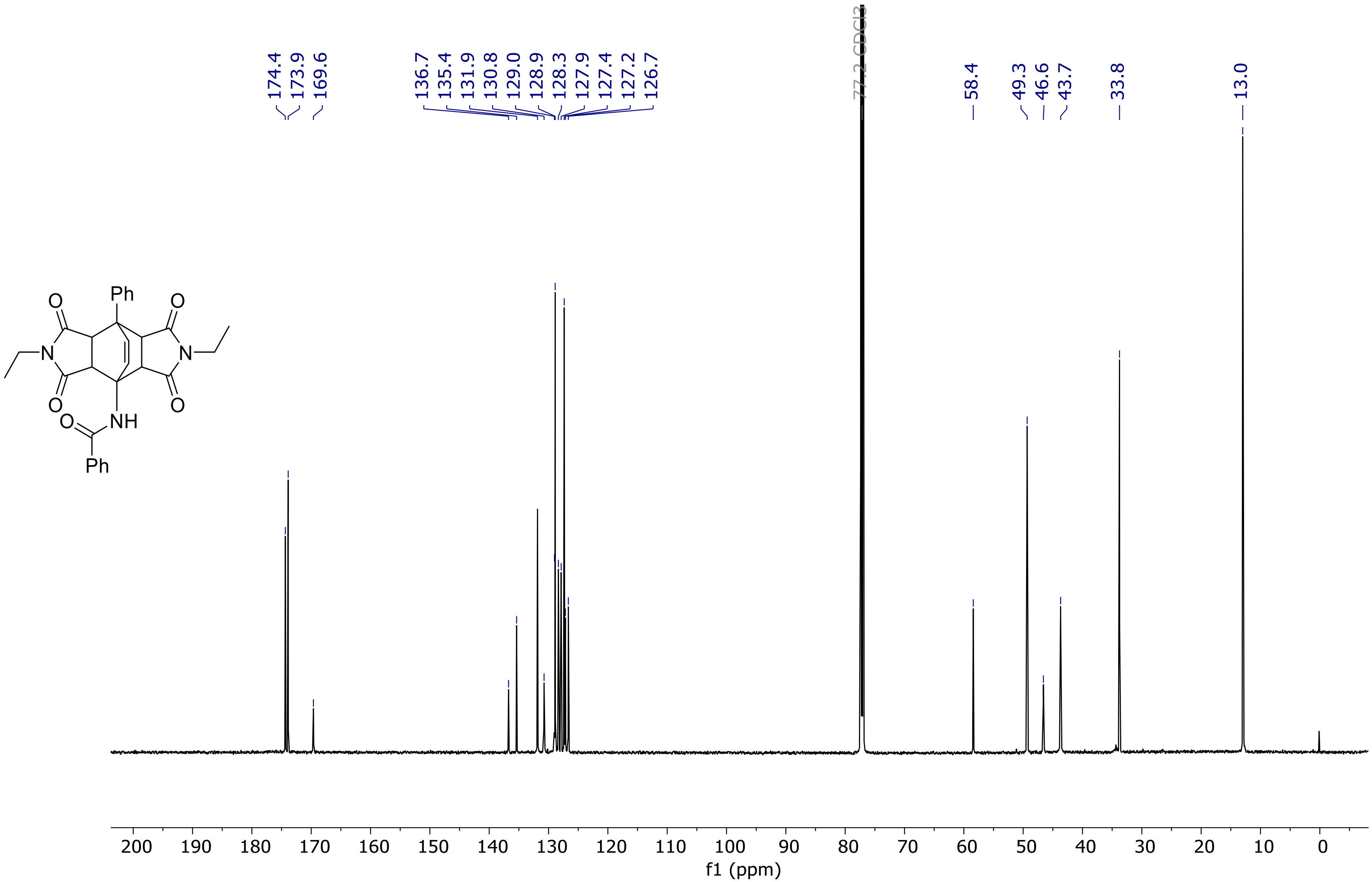

Figure S75. ^{1}H NMR Spectrum (500 MHz, $CDCl_3$) for Compound **4**

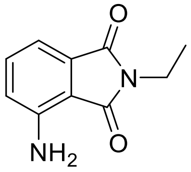

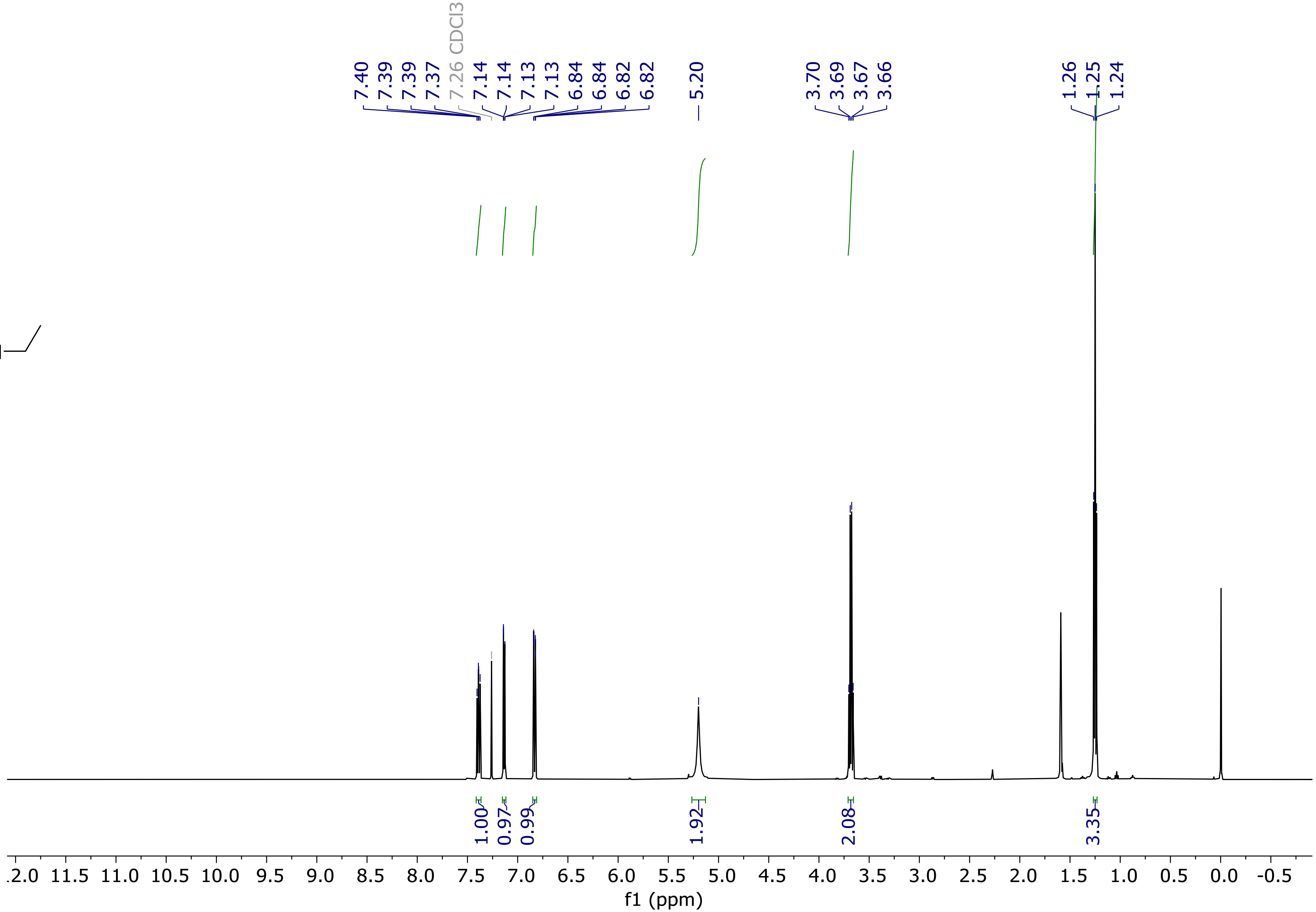

Figure S76. $^{13}C\{^{1}H\}$ NMR Spectrum (125 MHz, $CDCl_3$) for Compound **4**

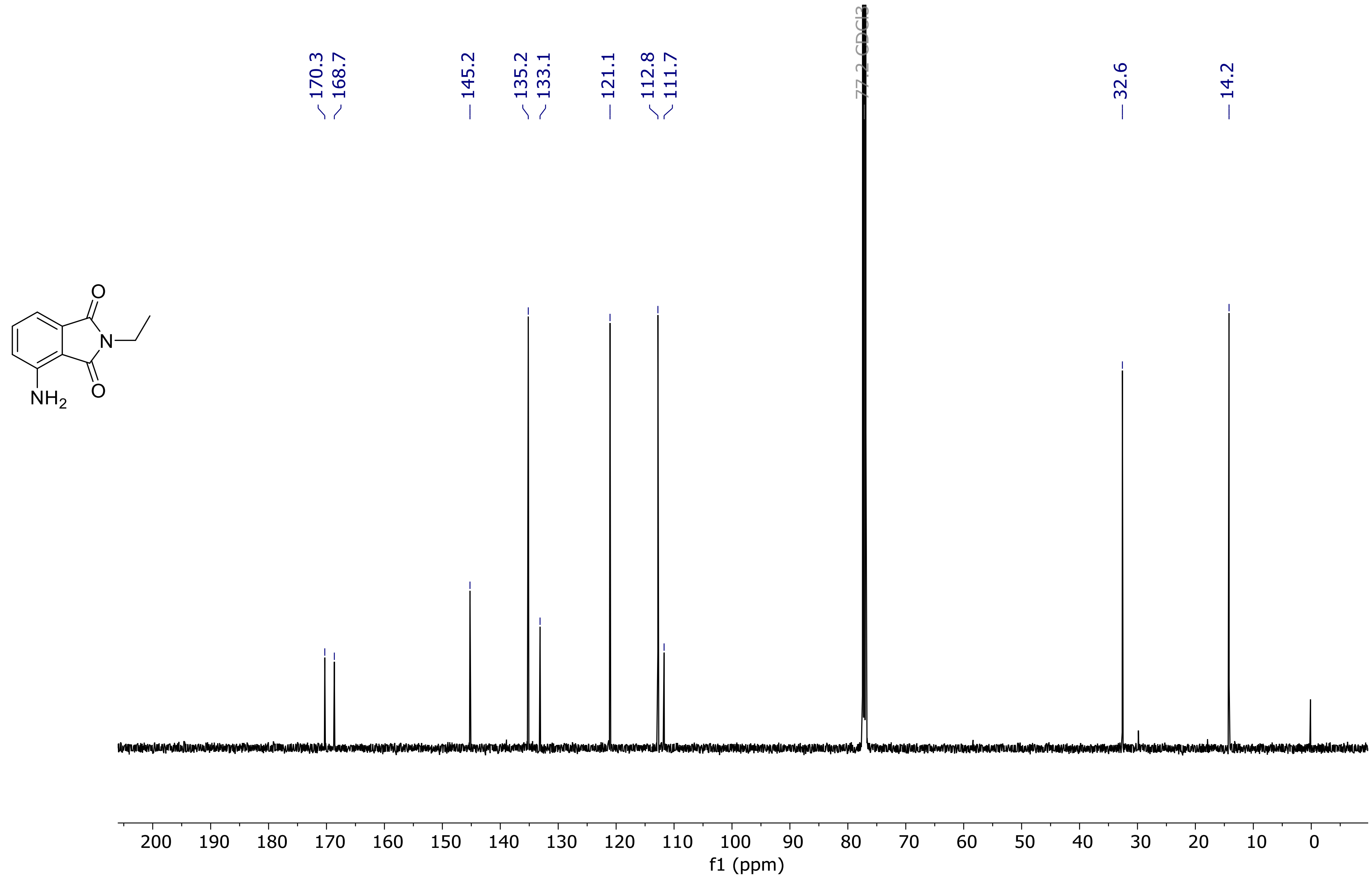

Figure S77. ^{1}H NMR Spectrum (600 MHz, $CDCl_3$) for Compound **5**

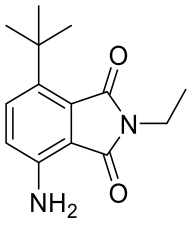

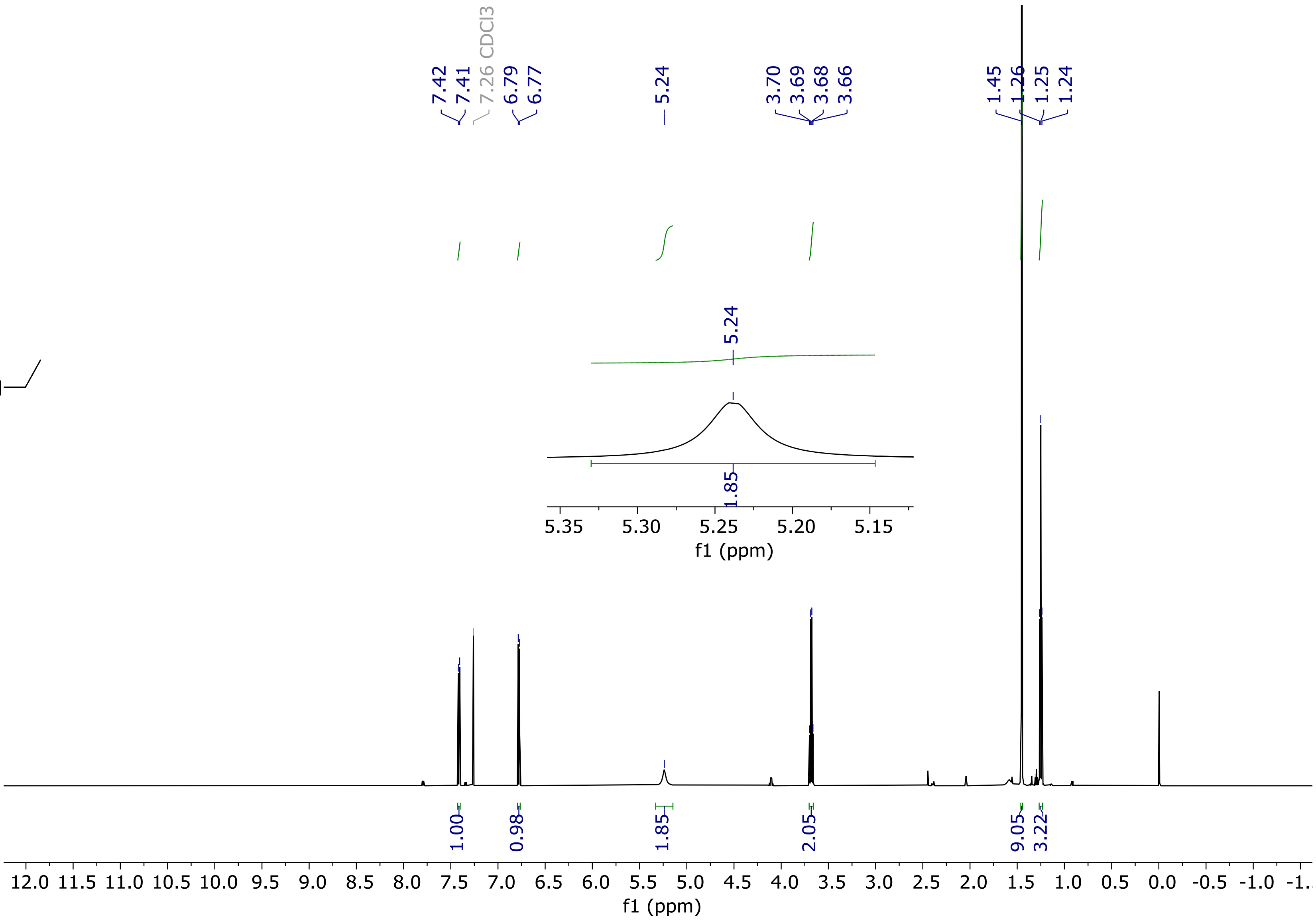

Figure S78. $^{13}C\{^{1}H\}$ NMR Spectrum (150 MHz, $CDCl_3$) for Compound **5**

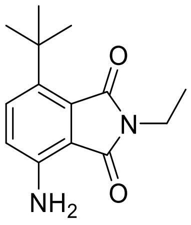

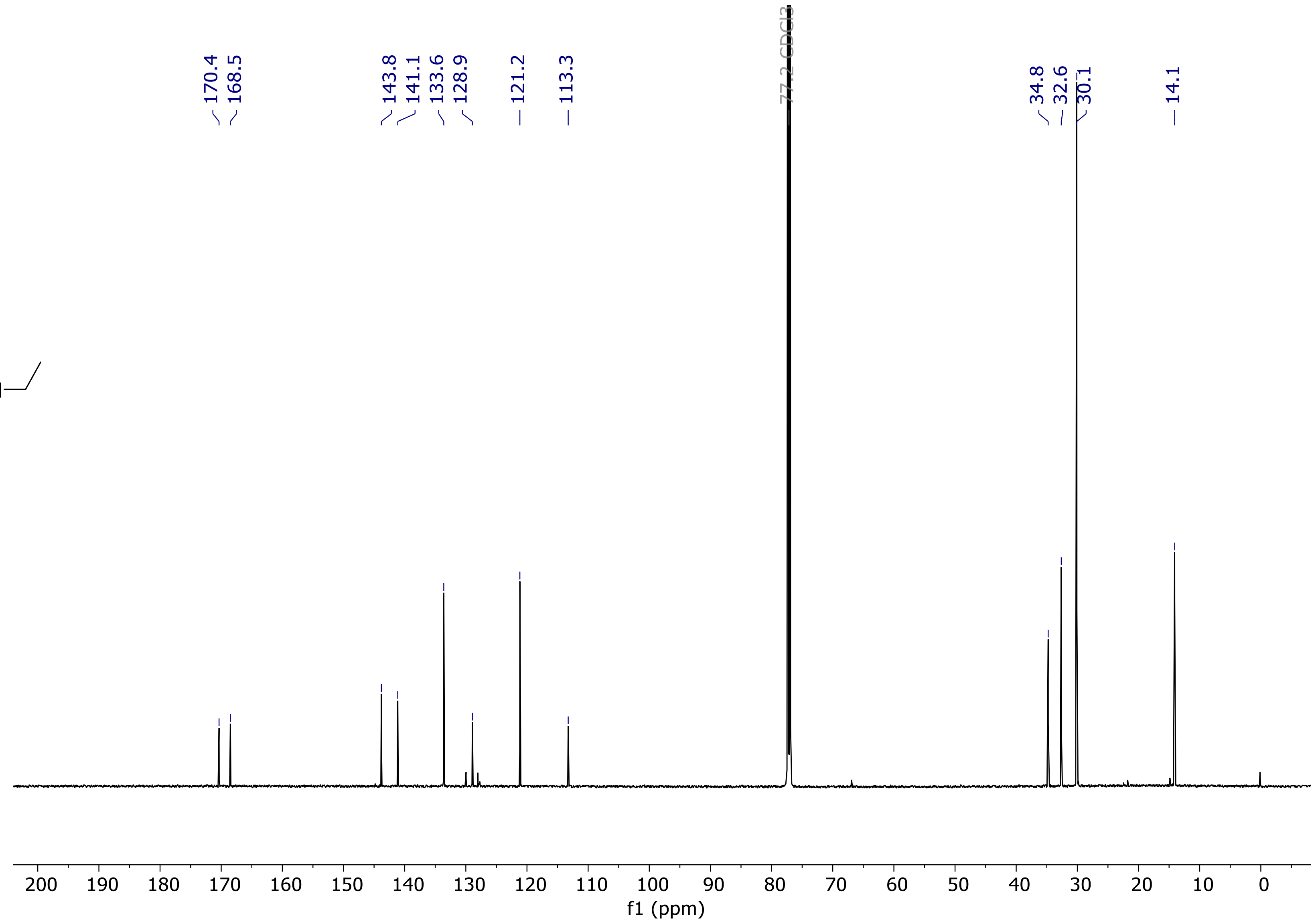

Figure S79. ^{1}H NMR Spectrum (600 MHz, DMSO-d$_6$) for Compound **6**

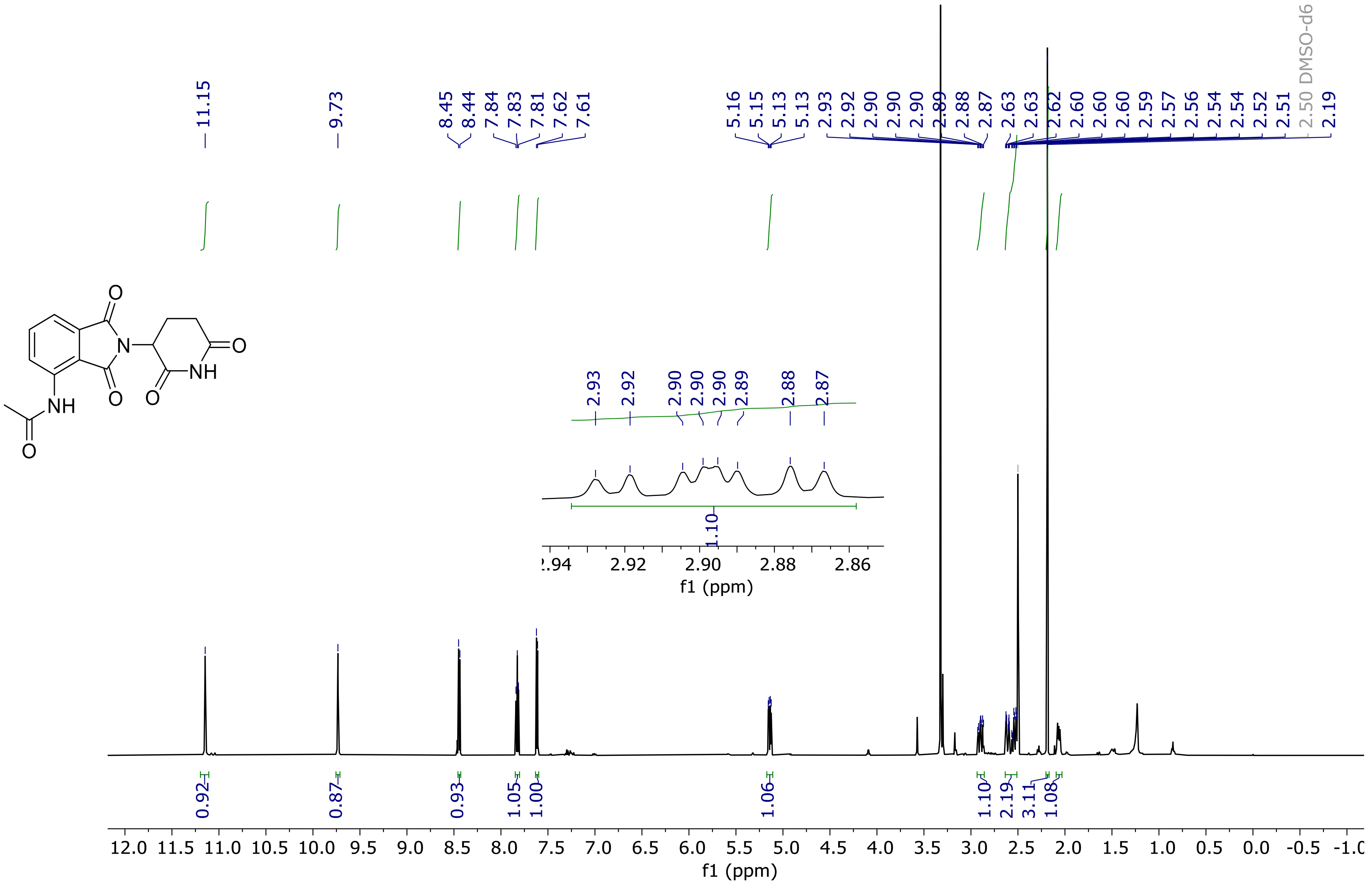

Figure S80. $^{13}C\{^{1}H\}$ NMR Spectrum (150 MHz, DMSO-d_6) for Compound **6**

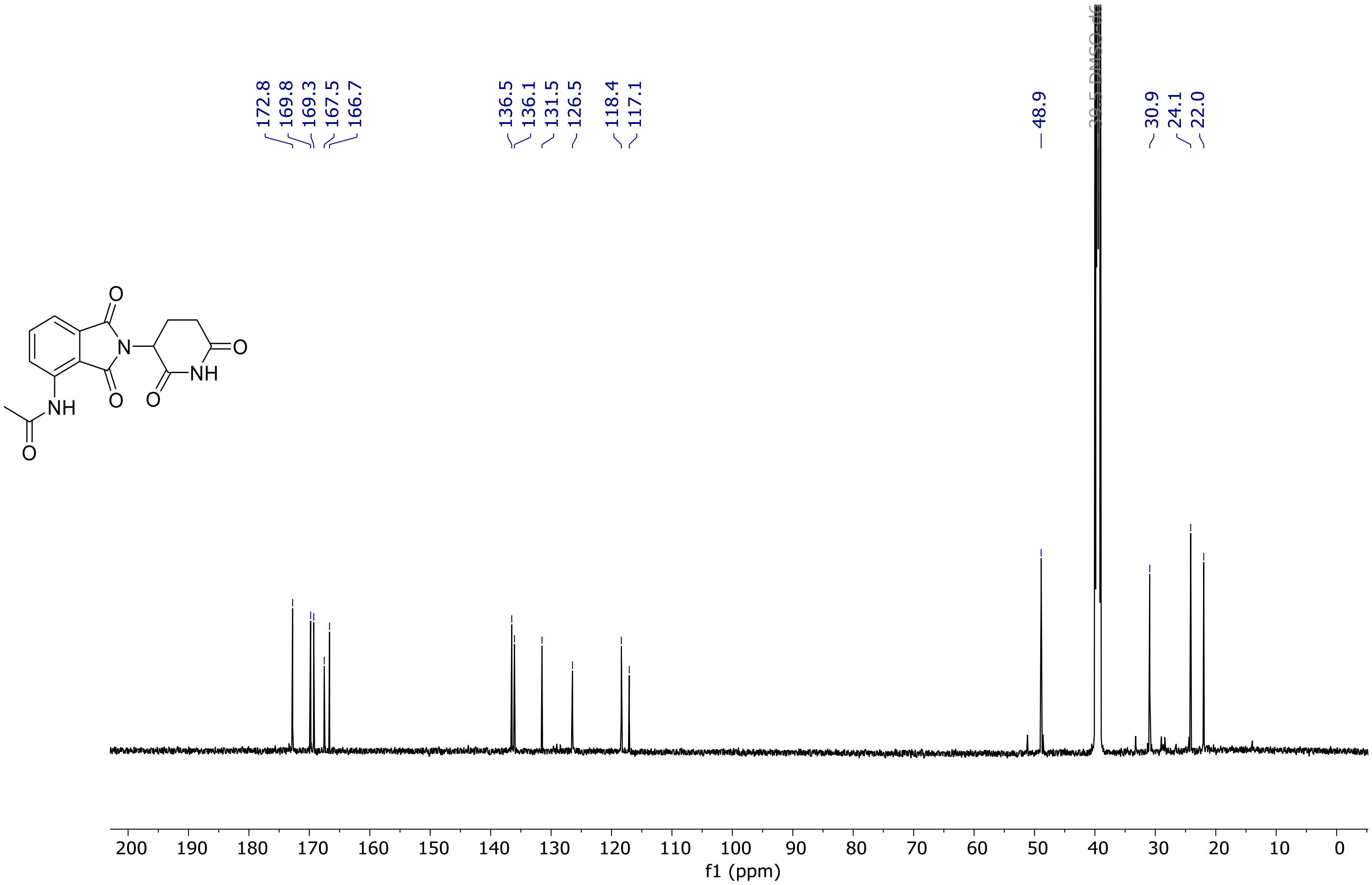

Figure S81. ^{1}H NMR Spectrum (500 MHz, DMSO-d$_6$) for Compound **7**

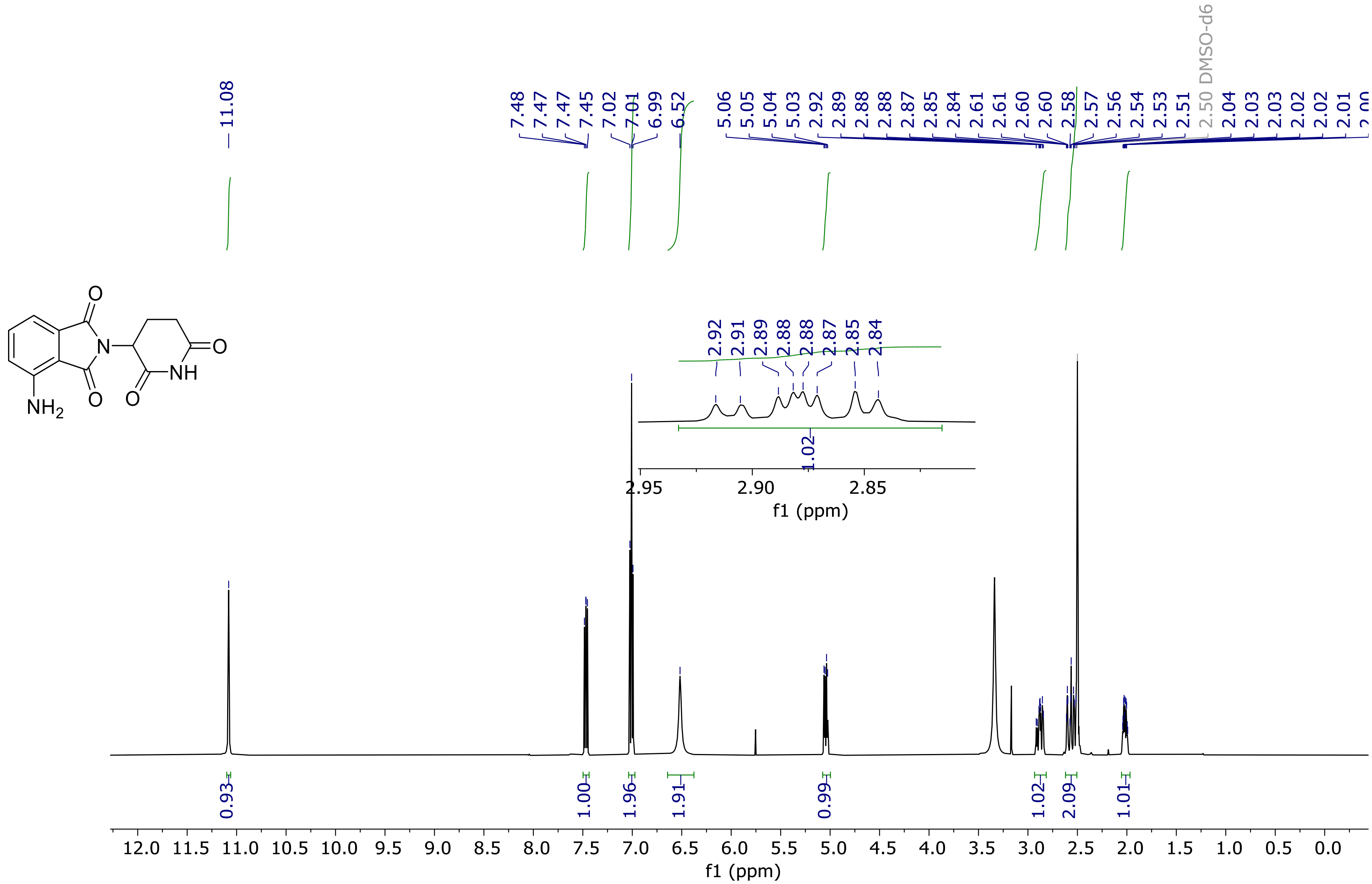

Figure S82. $^{13}C\{^{1}H\}$ NMR Spectrum (125 MHz, DMSO-d$_6$) for Compound **7**

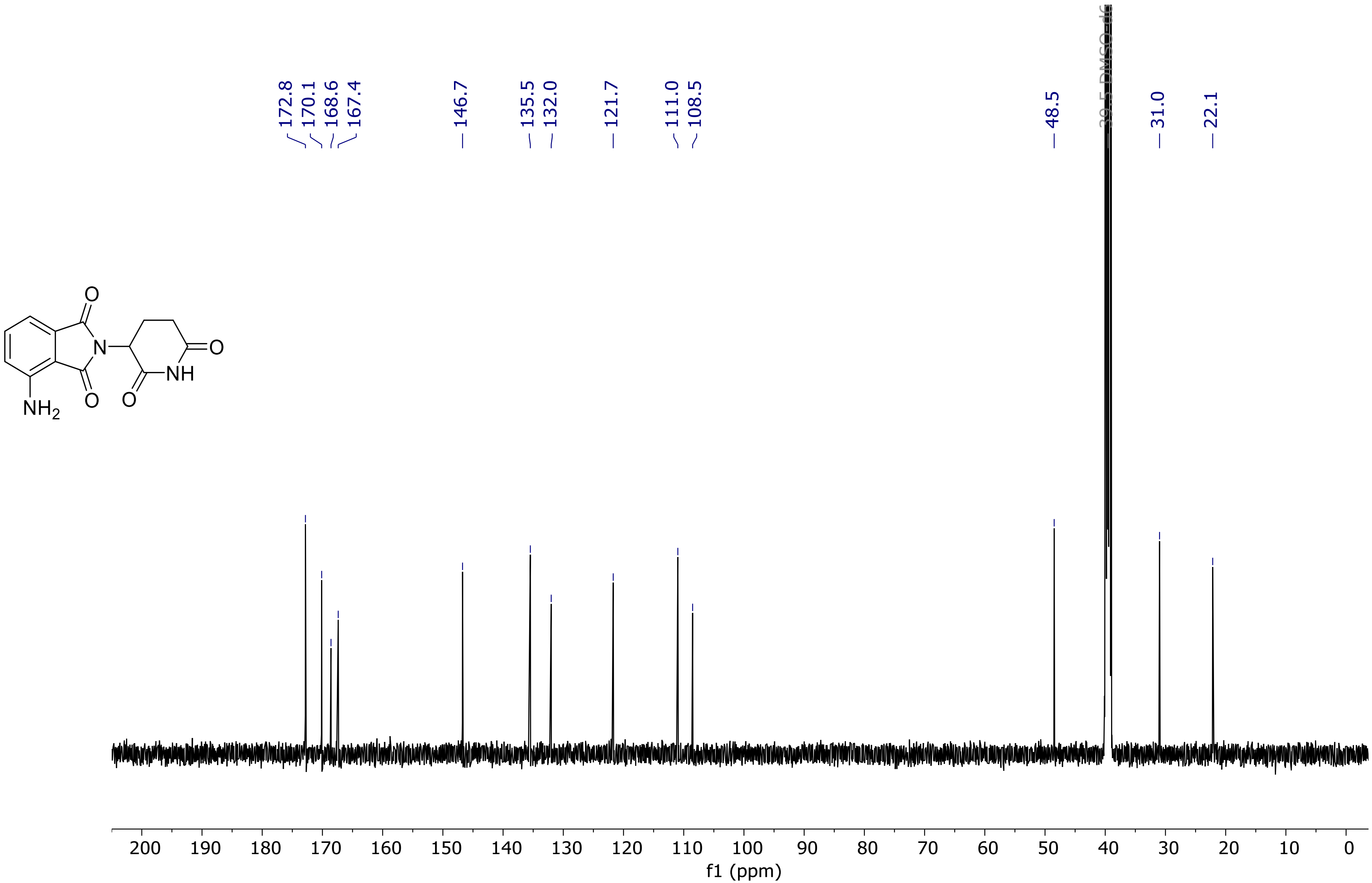

References

(1) Liashuk, O. S.; Grygorenko, O. O.; Volovenko, Y. M.; Waser, J. Photochemical [2+2] Cycloaddition of Alkynyl Boronates. *Chem. Eur. J.* **2023**, *29* (54). https://doi.org/10.1002/chem.202301650.

(2) Vol'eva, V. B.; Prokof'eva, T. I.; Belostotskaya, I. S.; Komissarova, N. L.; Gorbunov, D. B.; Kurkovskaya, L. N. Alkylation of Pyrocatechol in Tert-Butyl Alcohol–Sulfuric Acid–Benzene. *Russ. J. Org. Chem.* **2011**, *47* (9), 1310–1312. https://doi.org/10.1134/S1070428011090089.

(3) Lewis, N.; Morgan, I. A Facile and Mild Detert-Butylation Reaction. *Synth. Commun.* **1988**, *18* (15), 1783–1793. https://doi.org/10.1080/00397918808060933.

(4) Tashiro, M.; Yamato, T. Studies on Selective Preparation of Aromatic Compounds. *J. Org. Chem.,* Vol. 44, No. 17, **1979**.

(5) Kepe, V.; Kočevar, M.; Polanc, S. One-Pot Synthesis of Some 2H-Pyran-2-One Derivatives. *J. Heterocycl. Chem.* **1996**, *33* (6), 1707–1710. https://doi.org/10.1002/jhet.5570330626.

(6) Nguyen, T. M.; Sreekanth, V.; Deb, A.; Kokkonda, P.; Tiwari, P. K.; Donovan, K. A.; Shoba, V.; Chaudhary, S. K.; Mercer, J. A. M.; Lai, S.; Sadagopan, A.; Jan, M.; Fischer, E. S.; Liu, D. R.; Ebert, B. L.; Choudhary, A. Proteolysis-Targeting Chimeras with Reduced off-Targets. *Nat. Chem.* **2024**, *16* (2), 218–228. https://doi.org/10.1038/s41557-023-01379-8.

(7) Burslem, G. M.; Ottis, P.; Jaime-Figueroa, S.; Morgan, A.; Cromm, P. M.; Toure, M.; Crews, C. M. Efficient Synthesis of Immunomodulatory Drug Analogues Enables Exploration of Structure–Degradation Relationships. *ChemMedChem* **2018**, *13* (15), 1508–1512. https://doi.org/10.1002/cmdc.201800271.

(8) Rowan Scientific. *Rowan Scientific* **2025**.

(9) Anstine, D. M.; Zubatyuk, R.; Isayev, O. AIMNet2: A Neural Network Potential to Meet Your Neutral, Charged, Organic, and Elemental-Organic Needs. *Chem. Sci.* **2025**, *16* (23), 10228–10244. https://doi.org/10.1039/D4SC08572H.

(10) Zubatyuk, R.; Smith, J. S.; Leszczynski, J.; Isayev, O. Accurate and Transferable Multitask Prediction of Chemical Properties with an Atoms-in-Molecules Neural Network. *Science* **2019**.

(11) Zubatyuk, R.; Smith, J. S.; Nebgen, B. T.; Tretiak, S.; Isayev, O. Teaching a Neural Network to Attach and Detach Electrons from Molecules. *Nat. Commun.* **2021**, *12* (1). https://doi.org/10.1038/s41467-021-24904-0.

(12) Pracht, P.; Bohle, F.; Grimme, S. Automated Exploration of the Low-Energy Chemical Space with Fast Quantum Chemical Methods. *Phys. Chem. Chem. Phys.* **2020**, *22* (14), 7169–7192. https://doi.org/10.1039/C9CP06869D.

(13) Bannwarth, C.; Caldeweyher, E.; Ehlert, S.; Hansen, A.; Pracht, P.; Seibert, J.; Spicher, S.; Grimme, S. Extended Tight-Binding Quantum Chemistry Methods. *WIREs Comput. Mol. Sci.* **2021**, *11* (2), e1493. https://doi.org/10.1002/wcms.1493.

(14) Bannwarth, C.; Ehlert, S.; Grimme, S. GFN2-xTB—An Accurate and Broadly Parametrized Self-Consistent Tight-Binding Quantum Chemical Method with Multipole Electrostatics and Density-Dependent Dispersion Contributions. *J. Chem. Theory Comput.* **2019**, *15* (3), 1652–1671. https://doi.org/10.1021/acs.jctc.8b01176.

(15) Neese, F.; Wennmohs, F.; Becker, U.; Riplinger, C. The ORCA Quantum Chemistry Program Package. *J. Chem. Phys.* **2020**, *152* (22), 224108. https://doi.org/10.1063/5.0004608.

(16) Mattioli, R. R.; Santos, C. S.; de Souza, B. B.; Branco, P. D.; Bolt, R. R. A.; Raby-Buck, S. E.; Gomes Cabral, T. L.; Tormena, C. F.; Browne, D. L.; Pastre, J. C. On the Valorisation of Chitin-Derived Furans by Milling. *ChemSusChem* **2025**, *18* (3), e202401584. https://doi.org/10.1002/cssc.202401584.

(17) Santos, C. S.; Rodini Mattioli, R.; Soares Baptista, J.; da Silva, V. H.; Browne, D. L.; Pastre, J. C. Nitrogenated Aromatics from Chitin. *Green Chem.* **2023**, *25* (13), 5059–5067. https://doi.org/10.1039/D3GC00272A.

(18) Spicher, S.; Grimme, S. Single-Point Hessian Calculations for Improved Vibrational Frequencies and Rigid-Rotor-Harmonic-Oscillator Thermodynamics. *J. Chem. Theory Comput.* **2021**, *17* (3), 1701–1714. https://doi.org/10.1021/acs.jctc.0c01306.

(19) Grimme, S.; Bohle, F.; Hansen, A.; Pracht, P.; Spicher, S.; Stahn, M. Efficient Quantum Chemical Calculation of Structure Ensembles and Free Energies for Nonrigid Molecules. *J. Phys. Chem. A* **2021**, *125* (19), 4039–4054. https://doi.org/10.1021/acs.jpca.1c00971.

(20) Cabral, T. L. G.; da Silva, J. P.; Tormena, C. F.; Stein, M. Molecular Recognition and Chiral Discrimination from NMR and Multi-Scale Simulations. *Chem. Eur. J.* **2025**, *31* (25), e202404694. https://doi.org/10.1002/chem.202404694.

(21) Brauser, M.; Petzold, K.; Thiele, C. M. Investigating Interaction Dynamics of an Enantioselective Peptide-Catalyzed Acylation Reaction. *Angew. Chem. Int. Ed.* **2025**, *64* (10), e202421062. https://doi.org/10.1002/anie.202421062.

(22) Statham, B. J.; Mulks, F. F.; Bolm, C. π-Electron Donation at the Sulfoximidoyl Nitrogen Atom. *Angew. Chem. Int. Ed.* **2025**, *64* (33), e202510744. https://doi.org/10.1002/anie.202510744.

(23) Ehlert, S.; Stahn, M.; Spicher, S.; Grimme, S. Robust and Efficient Implicit Solvation Model for Fast Semiempirical Methods. *J. Chem. Theory Comput.* **2021**, *17* (7), 4250–4261. https://doi.org/10.1021/acs.jctc.1c00471.

(24) Marenich, A. V.; Cramer, C. J.; Truhlar, D. G. Universal Solvation Model Based on Solute Electron Density and on a Continuum Model of the Solvent Defined by the Bulk Dielectric

Constant and Atomic Surface Tensions. *J. Phys. Chem. B* **2009**, *113* (18), 6378–6396. https://doi.org/10.1021/jp810292n.

(25) Becke, A. D. Density-Functional Thermochemistry. III. The Role of Exact Exchange. *J. Chem. Phys.* **1993**, *98* (7), 5648–5652. https://doi.org/10.1063/1.464913.

(26) Petersson, G. A.; Bennett, A.; Tensfeldt, T. G.; Al-Laham, M. A.; Shirley, W. A.; Mantzaris, J. A. A Complete Basis Set Model Chemistry. I. The Total Energies of Closed-Shell Atoms and Hydrides of the First-Row Elements. *J. Chem. Phys.* **1988**, *89* (4), 2193–2218. https://doi.org/10.1063/1.455064.

(27) Caldeweyher, E.; Mewes, J.-M.; Ehlert, S.; Grimme, S. Extension and Evaluation of the D4 London-Dispersion Model for Periodic Systems. *Phys. Chem. Chem. Phys.* **2020**, *22* (16), 8499–8512. https://doi.org/10.1039/D0CP00502A.

(28) Perdew, J. P.; Burke, K.; Ernzerhof, M. Generalized Gradient Approximation Made Simple. *Phys. Rev. Lett.* **1996**, *77* (18), 3865–3868. https://doi.org/10.1103/PhysRevLett.77.3865.

(29) Lin, Y.-S.; Li, G.-D.; Mao, S.-P.; Chai, J.-D. Long-Range Corrected Hybrid Density Functionals with Improved Dispersion Corrections. *J. Chem. Theory Comput.* **2013**, *9* (1), 263–272. https://doi.org/10.1021/ct300715s.

(30) Schäfer, A.; Huber, C.; Ahlrichs, R. Fully Optimized Contracted Gaussian Basis Sets of Triple Zeta Valence Quality for Atoms Li to Kr. *J. Chem. Phys.* **1994**, *100* (8), 5829–5835. https://doi.org/10.1063/1.467146.

(31) Barone, V.; Cossi, M. Quantum Calculation of Molecular Energies and Energy Gradients in Solution by a Conductor Solvent Model. *J. Phys. Chem. A* **1998**, *102* (11), 1995–2001. https://doi.org/10.1021/jp9716997

(32) Ishida, K.; Morokuma, K.; Komornicki, A. The Intrinsic Reaction Coordinate. An *Ab Initio* Calculation for HNC → HCN and H^- + CH_4 → CH_4 + H^-. *J. Chem. Phys.* **1977**, *66* (5), 2153–2156. https://doi.org/10.1063/1.434152
